# Research on recognition of slippery road surface and collision warning system based on deep learning

**DOI:** 10.1371/journal.pone.0310858

**Published:** 2024-11-11

**Authors:** Huiqi Du, Lei Wang, Mingjiang Cai

**Affiliations:** 1 Continuing Education and Training Centre, Tianjin Sino-German University of Applied Sciences, Tianjin, China; 2 School of Automobile & Rail Transportation, Tianjin Sino-German University of Applied Sciences, Tianjin, China; 3 School of Automobile and Transportation, Tianjin University of Technology and Education, Tianjin, China; Southwest Jiaotong University, CHINA

## Abstract

Aiming at the problems of slow detection speed, large prediction error and weak environmental adaptability of current vehicle collision warning system, this paper proposes a recognition method of slippery road surface and collision warning system based on deep learning. Firstly, this paper uses the on-board camera to monitor the environment and road conditions in front of the vehicle in real time, and a residual network model FS-ResNet50 is proposed, which integrated SE attention mechanism and multi-level feature information based on the traditional ResNet50 model. The FS-ResNet50 model is used to identify the slippery states of the current road, such as wet and snowy. Secondly, the yolov5 algorithm is used to detect the position of the vehicle in front, and a driving safety distance model with adaptive traffic environment characteristics is established based on different road conditions and driving conditions, and an early warning area that dynamically changed with the speed and the road slippery states is generated. Finally, according to the relationship between the warning area and the position of the vehicle, the possible collision is predicted and timely warned. Experimental results show that the method proposed in this paper improves the overall warning accuracy by 6.72% and reduces the warning false alarm rate for oncoming traffic on both sides by 16.67% compared with the traditional collision warning system. It can ensure safe driving, especially in bad weather conditions and has a high application value.

## 1. Introduction

With the continuous development of transportation industry, road traffic safety has become a concern problem, and the road slippery states are an important factor affecting driving safety. For example, when driving on rainy days, the water on the road will bring safety hazards such as prolonged braking distance and tire slip to the driver, especially in the forward collision accident, due to the driver’s misjudgment of the distance between the vehicles.

Causing serious casualties and property losses [1]. In order to solve this problem, more and more research and engineering practice focus on the development of vehicle front collision warning system. Advanced driving assistance system can assist drivers to perform correct operation to a certain extent, thus improving driving safety and reducing the occurrence of traffic accidents [2, 3].

Many domestic and international scholars have conducted research in the field of forward collision warning system, and the main approaches can be divided into Lidar-based, millimeter wave radar-based and deep learning-based collision warning research. Lidar-based and millimeter-wave radar-based technologies rely heavily on Lidar and millimeter-wave radar, however, these sensors are not good at recognizing static objects, and the equipment is expensive and cannot visualize road information. In comparison, machine vision-based collision warning has a series of advantages such as low cost and the ability to visualize road information, which is favored by a wide range of manufacturers [4, 5].Therefore, the recognition of slippery road surface and collision warning system based on deep learning can directly solve the pain points, improve some defects of the previous vehicle forward collision warning algorithm, improve the accuracy of early warning, reduce the false alarm rate, and improve the driver’s trust in the early warning system.

## 2. Related work

At present, the method of road condition recognition is mainly based on machine vision. The current mainstream machine vision recognition methods are mainly divided into image processing algorithms and deep learning based methods. The image processing algorithm mainly uses OpenCV technology and support vector machine (SVM) to extract image features. Chen et al. [6] extracted pavement texture features by grey co-occurrence matrix, and then classified 5 common road surfaces by SVM. The method based on deep learning mainly extracts the image feature information through the neural network. Gin [7] designed a new residual unit, the Ref-Block, and built a lightweight network, RefNet, which was accelerated by the Tensor RT inference machine to classify road surfaces. Chen et al. [8] used adversarial neural networks to first remove the road surface shadow, and then used residual networks to classify the road surfaces. Yang et al.[9] firstly designed an adaptive correction algorithm based on two-dimensional gamma function to correct and process the photo data with uneven illumination, and then ResNet18 model was used for the road surface recognition. In summary, although the existing recognition methods can recognize the road surface, they are easy to be interfered by other irrelevant information, and the generalization performance is not high. To solve these problems, this paper proposes a recognition method of slippery road surface based on improved ResNet50 network.

There is a wide variety of research on machine vision-based collision warning systems. Cai ChuangXin constructed a safety distance model by conducting a detailed study of lane line detection, however, this method can only be applied in roads with distinct lane lines and is not suitable for unstructured driving scenarios [10]. He Yong judged whether the overlap between the left and right vehicles in front and the lane line exceeds 50%, combined with the vehicle’s turn signal to determine the cut-in of the vehicle in front, but this method requires high accuracy for the detection algorithm and does not make good detection when encountering extreme weather such as rainy and snowy [11]. Hiraoka and Takada proposed the concept of "collision avoidance reduction" parameter to predict the probability of collision with the vehicle in front, but this concept is based on the premise that the vehicle is driving at a constant speed, not in line with the actual road driving situation [12]. ShenHaiyang proposed to delineate different safe driving zones according to different vehicle speeds, the method does not consider the friction coefficient between vehicle tires and the ground under different road conditions, which has certain limitations [13]. In summary, machine vision-based vehicle collision warning technology currently has problems such as slow detection speed, large prediction error in the warning area, and environment weak adaptability, which lead to drivers’ distrust of the warning system.

In view of the problems of the above collision warning algorithm, this paper proposes a collision warning system based on the recognition of slippery road surfaces. The main idea of the algorithm is to build a safety distance model with adaptive traffic environment characteristics, which can considering the influence of forward obstacle types, motion states and road conditions on forward collision, thereby generating a warning area that changes dynamically with the speed of the self vehicle and the slippery states of road surface. Finally, according to the relationship between the area of the warning and the location of the vehicle, timely warning is provided for possible collision situations.

## 3. Recognition of slippery road surface

### 3.1 FS-ResNet50 model

In the process of road driving, road conditions can be broadly divided into: dry road, wet road and snowy road, etc. The traditional method of acquiring road status mainly relies on the meteorological department to detect and obtain data sets of various weather elements, identify the current weather, and then transmit the road status of the current car driving section to the driver through the broadcast, network and other ways. Subsequently, the recognition method of road surface is mainly realized through image recognition, that is, the image is preprocessed by image enhancement, denoising and color conversion, and then the features in the image, such as brightness, fullness and degree, are extracted to complete the recognition of different road surfaces. However, this method is easily affected by lighting and complex traffic scenes. Compared with traditional image recognition, convolutional neural network can extract more abundant image features with higher recognition accuracy [14, 15]. Therefore, ResNet50 network model in convolutional neural network will be used in this paper to identify road surfaces.

The ResNet50 model is a commonly used architecture in deep residual networks, which effectively addresses the common problem of gradient vanishing in the training of deep neural networks by introducing residual connections (skip connections) [16]. The FS-ResNet50 model proposed in this paper is based on the ResNet50 model and further enhances the model’s detection speed and accuracy by introducing new mechanisms and strategies.

The traditional ResNet-50 model employs a 7×7 convolutional layer in its first layer, which can lead to increased computational burden and parameter count, thereby affecting the efficiency of model training and inference. The proposed FS-ResNet50 model replaces the 7×7 convolutional layer in the first layer of the original ResNet50 model with three consecutive 3×3 convolutional kernels. This approach maintains the receptive field while adding more nonlinear transformations and network depth, enabling the network to learn more complex features. Additionally, smaller convolutional kernels effectively reduce model parameters, thereby decreasing model computational complexity and the risk of overfitting.

**Fig 1:**
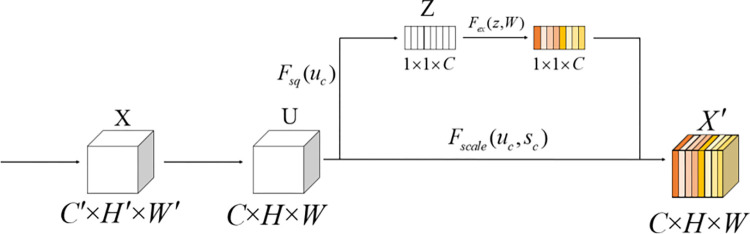
Schematic diagram of the SE attention mechanism module.

The attention mechanism is a technique used to enhance the performance of convolutional neural networks. It enables the network to focus more on critical feature channels by adaptively learning the weights of each channel. Therefore, FS-ResNet50 introduces the SE (Squeeze-and-Excitation) attention mechanism at the end of each residual block in the original model. The SE attention mechanism reshapes the feature channels through two steps: squeeze and excitation, enhancing the model’s ability to recognize important regions in images. The structure of the SE attention mechanism is shown in Fig 1.

**Fig 2:**
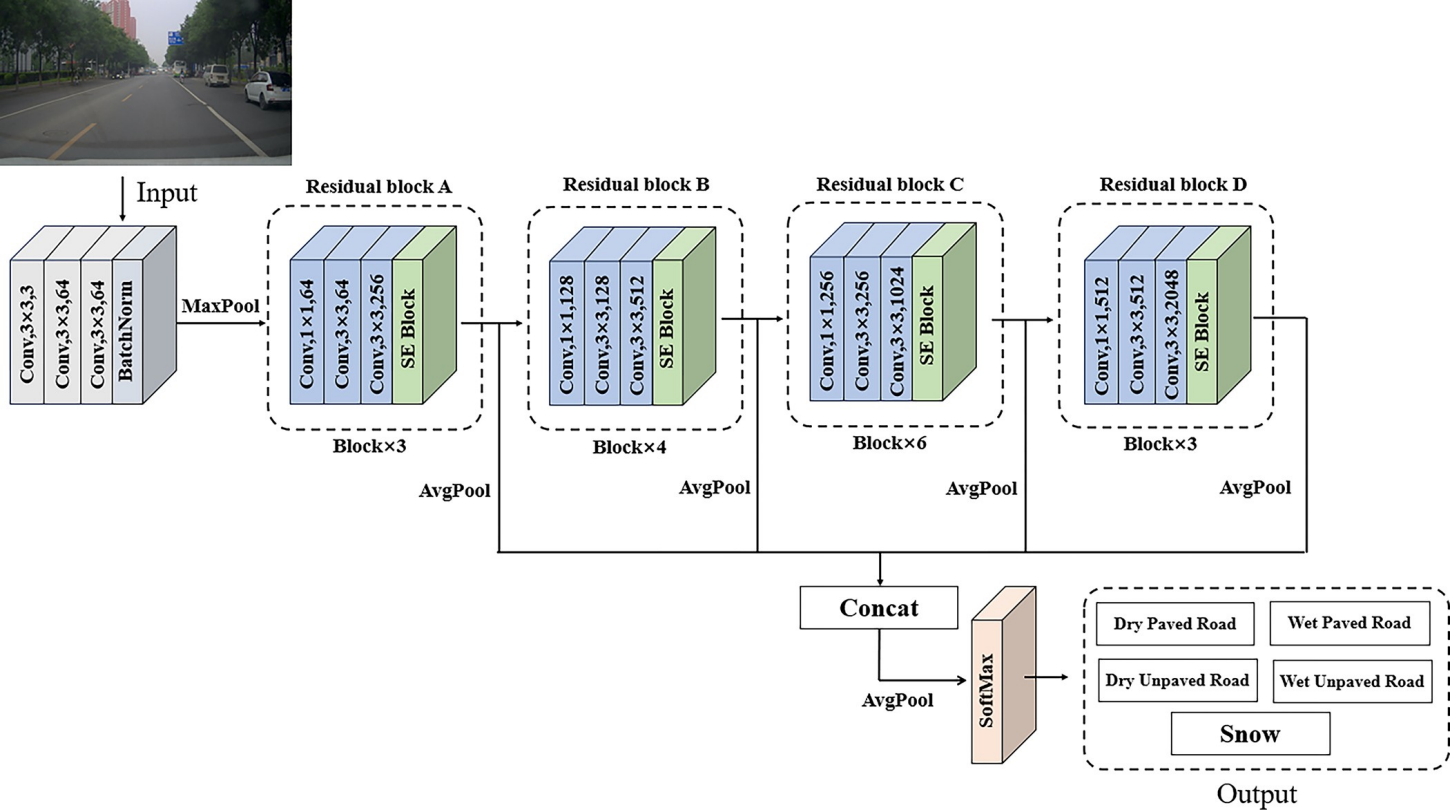
The structure of the FS-ResNet50 model.

In road surface conditions recognition tasks, environmental factors such as uneven or extreme lighting conditions can increase the difficulty of recognition. To improve the recognition rate of road surface conditions, the FS-ResNet50 model enhances the model’s ability to capture global features by applying global average pooling to the output of each residual block. Subsequently, these four globally averaged pooled features are fused to achieve cross-level feature integration. Through feature fusion, features from different network levels complement and enhance each other, thereby improving the model’s recognition accuracy of road surface conditions in complex traffic scenes and enhancing the model’s generalization performance. The structure of the FS-ResNet50 model is shown in Fig 2.

### 3.2 Experimental analysis

After building the network model, we started image acquisition, and the road surface images under different weather conditions were collected by the acquisition equipment, and the size of the images collected was 1980×1020. For images with insufficient data, this paper selected road images that were consistent with this experiment from KITTI dataset, BDD100K dataset and Oxford RobotCardataset [17–19]. In our experiments, we utilized high-performance GPUs to accelerate model training. Regarding training data volume, we used a total of 6,380 images to train the model, including 4,700 images captured on-site and 1,680 images sourced from open-source datasets.

**Fig 3:**
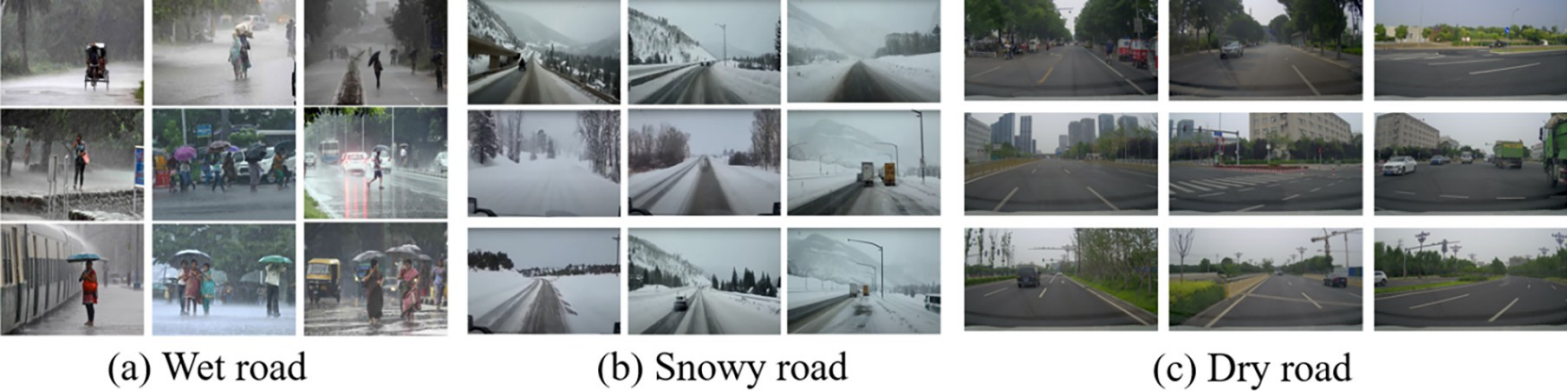
Road surface section data diagram.

All collected images were collected together to form an experimental data set (see S1 Dataset), labeled and summarized one by one, and resulting classification results were roughly classified into three major categories: dry, wet and snowy, as in Fig 3 and then trained for recognition using the FS-ResNet50 network model. The specific parameter settings and environment configuration of this research training were shown in Table 1.

**Table 1:** Experimental environment configuration and parameter setting.

Category	Model	Category	Value
CPU	Intel(R) Core(TM) i7-9750H	Input size (pixel×pixel)	224×224
GPU	NVIDIA GeForce GTX 1660 Ti	Learning rate	0.001
Internal storage	16G	Batch size	16
Deep Learning Framework	PyTorch 1.10.2	Iterations	100

**Fig 4:**
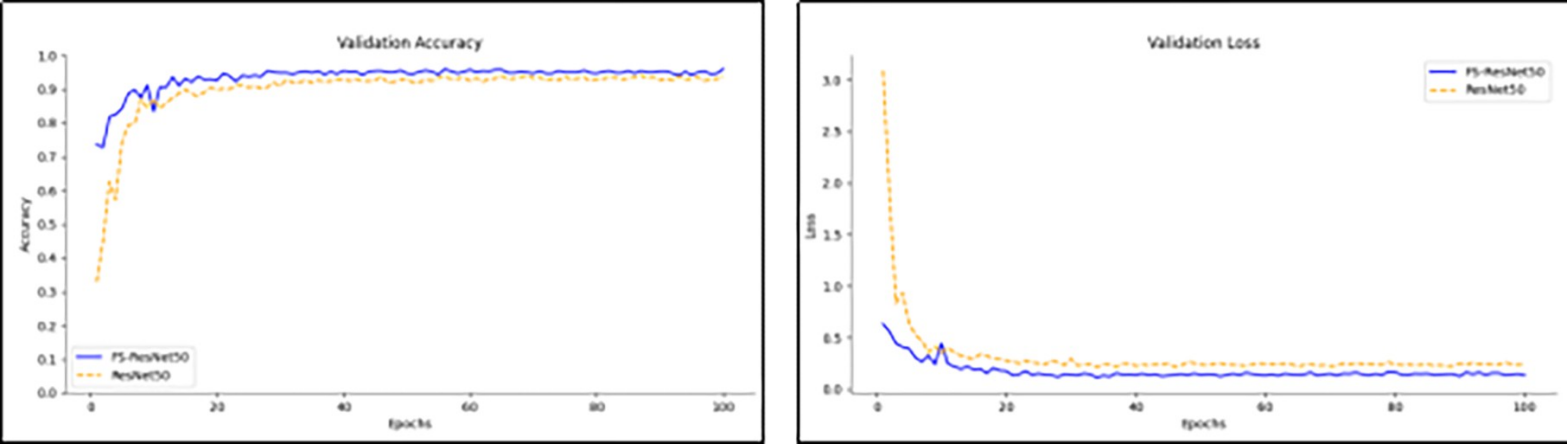
Iteration curves of accuracy and loss values.

This paper compares the accuracy and loss values of the ResNet50 network and the FS-ResNet50 model tested on the test set. The comparison results are shown in Fig 4. As can be seen from the figure, the initial recognition accuracy of the FS-ResNet50 model is 73.55%, and the highest accuracy is 95.87%; the initial recognition accuracy of the ResNet50 model is 32.87%, and the highest accuracy is 93.73%. The initial loss value of the FS-ResNet50 model is 0.63, and the lowest loss value is 0.136; the initial loss value of the ResNet50 model is 3.09, and the lowest loss value is 0.241. Additionally, the FS-ResNet50 model has a smaller fluctuation range and a faster descent speed in loss values compared to the ResNet50 model.

In order to further verify the superiority of FS-ResNet50 model, The performance of the FS-ResNet50 model was compared with the ResNet50, ResNext, MobileViTv3, ResNet101, DenseNet and EfficientNet models commonly used in the field of image classification. The experimental results were shown in Table 2.

**Table 2:** Same model recognition accuracy.

Model	Evaluation index			
	Training set accuracy (%)	Verification set accuracy (%)	Average processing time of a single image (s)	Floating-point arithmetic
ResNet50	97.17	92.13	0.243	3.89×10^9^
ResNext	97.64	92.73	0.301	4.82×10^9^
MoblieViTv3	96.96	91.34	0.284	2.54×10^9^
ResNet101	97.10	91.81	0.457	5.02×10^9^
DenseNet	97.89	92.84	0.451	5.31×10^9^
EfficientNet	97.62	93.47	0.456	5.36×10^9^
FS-ResNet50	98.31	95.87	0.275	4.17×10^9^

As can be seen from the Table 3, the average processing time of a single image of FS-RESnet50 model is similar to that of other models. However, the training set accuracy is respectively improved by 1.14, 0.67, 1.35, 0.121, 0.42 and 0.69 percentage points compared to ResNet50, ResNext, MoblieViTv3, ResNet101, DenseNet and EfficientNet. The accuracy of verification set is respectively improved by 3.74, 3.1processing4, 4.53, 4.06, 3.03 and 2.40 percentage points. The network models of ResNext, ResNet101, DenseNet and EfficientNet are more complex, with larger parameters and larger floating-point computation, while the models of ResNet50 and MoblieViTv3 have lower floating-point computation. However, the validation set accuracy of FS-ResNet50 model is much higher than that of ResNet50 and MoblieViTv3. In this paper, the recognition accuracy of the image takes precedence over the detection speed, so the FS-ResNet50 model has obvious advantages. Through comparison, it can be concluded that the improvement strategy and optimization mode of FS-ResNet50 model can obtain better recognition results. Other models performed poorly due to redundant parameters, simple design, or inadequate capture of local details.

**Table 3:** Comparative test results of vehicle inspection.

Algorithm	Average accuracy	FPS
Haar feature algorithm	90.03%	5.30
R-CNN	97.35%	10.04
YOLOV5	96.31%	30.50

**Fig 5:**
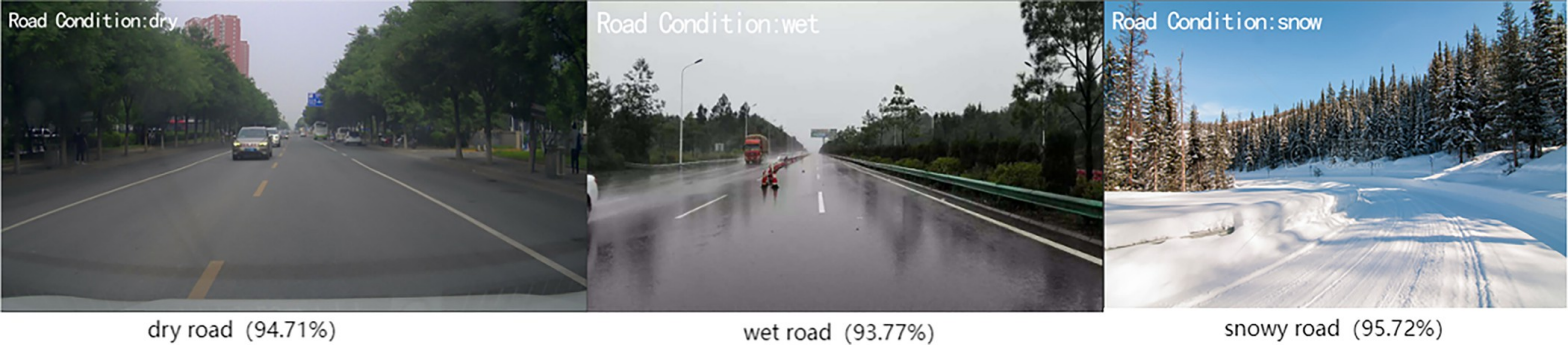
Results of recognition of slippery states of road surface.

To test the recognition effect of the model in actual traffic scenes, this paper collected images of different road surfaces through online collection and offline real-life photography and used the FS-ResNet50 model for recognition. The effect of road surface recognition is shown in Fig 5, and the values in brackets represent the recognition accuracy of the model in this paper for the current pavement type. As can be seen from the Fig 5, this method can effectively recognize the conditions of various road surfaces in actual driving environments.

## 4. Vehicle collision warning strategy

### 4.1. Vehicle detection

Within the last decade, target detection algorithms have become more and more numerous as they continue to iterate. However in the final analysis they can be divided into two main categories: two-stage detectors and one-stage detectors. The one-stage detector outputs localization and classification at the same time, much faster than the two-stage detector. Considering that the traffic information around the vehicle during the driving process are constantly changing, the early warning system have high requirements for the real-time recognition of the target object, so the method used in this paper is the yolov5 algorithm in the one-stage detector. Yolov5 algorithm detects images faster than Haar feature algorithm and R-CNN algorithm [20]. In order to test the effectiveness of the algorithms, this paper obtained a part of the video captured by the on-board camera for experiments, and compared the average accuracy and the frames per second (FPS) of the video detected by the three algorithms. The experimental results are shown in Table 3. As can be seen from the table, in terms of frame rate, the detected video frame rate of R-CNN is much lower than the normal video frame rate of 25–30 frames/s, and the frame rate of Haar feature algorithm is only 10.04 frames/s, while the frame rate of yolov5 algorithm keeps the same as the original video frame rate. In terms of accuracy, yolov5 is more accurate than the Haar feature algorithm in terms of average target detection accuracy and is similar to the accuracy of the R-CNN algorithm. The above proves that yolov5 can better meet the accuracy and real-time requirements of forward collision warning system.

### 4.2 Improved model for safe driving distance

The delineation of travel danger zones is an important issue in collision warning. Accurate delineation of driving early warning areas can help drivers anticipate potentially dangerous situations in advance and take appropriate safety measures, thereby reducing the probability of traffic accidents. The traditional method of traffic Early warning zone classification mainly relies on manual observation and empirical judgment, which is subjective, easily influenced by individual experience and subjective consciousness, and cannot meet the needs of large-scale road networks [21].Therefore, in order to construct an objective road collision warning area model, a "safe distance" needs to be set to avoid collisions.

In this paper, we use the real-time estimation of road friction coefficient proposed by Tang Hongdu et al. [22] to change the safe distance between vehicles. This algorithm considers the influence of the characteristics of different driving styles, the road slippery state and the front and rear vehicles speed on the safe distance, so as to establish a driving safety distance model with adaptive traffic environment characteristics, as shown inEquation1:

$$\begin{array}{l} D=\frac{V_{f}^{2}}{2a_{m}j}+V_{f}(T_{d}+T_{f}+T_{m}\rho )-\frac{V_{L}^{2}}{2a_{L}}\\ j=\left\{ \begin{array}{l} f(\varphi_{\min})\;\varphi <\varphi_{\min}\\ f(\varphi_{\min})+\frac{f(\varphi_{\max})-f(\varphi_{\min})}{\varphi_{\max}-\varphi_{\min}}(\varphi -\varphi_{\min})\;\varphi_{\min}<\varphi <\varphi_{\max}\\ f(\varphi_{\max})\;\varphi_{\max}<\varphi \end{array}\right. \end{array}$$ (1)

Where *V*_*f*_ represents the speed of the self vehicle and *V*_*L*_ is the speed of the front vehicle. *T*_*d*_ represents the driver’s reaction time, *T*_*f*_ is the driver’s judgment time, *T*_*m*_ indicates the driver’s time to take braking action, *ρ* is the driving characteristics weight, according to different driving styles of drivers to adjust the value, generally take the value of 0.25 ~ 1.00, because this paper focuses on the impact of road conditions and vehicle speed on the safe distance, so the value of *ρ* will be 1.00. *a*_*m*_ and *a*_*L*_ are respectively the acceleration of this self vehicle and the front vehicle. j represents the influence of the road condition on the safe distance, and its value is mainly determined by the friction coefficient *φ*,there into, *φ*_max_ is the friction coefficient of the normal road conditions, *φ*_min_ is the friction coefficient of the worst road conditions, such as the icing road, etc. According to the relevant research results, when driving in the normal road environment, the value of *f*(*φ*_max_) is 1, when driving in the snow and ice road environment, the value of *f*(*φ*_*m*in_) is 0.3.Referring to the results of the other reference [23], the values of *a*_*m*_ and *a*_*L*_ are tentatively set as 6m/s2, *T*_*d*_ = 0.2s, *T*_*f*_ = 0.1s, *T*_*m*_ = 2s, *V*_*f*_ and *V*_*L*_ are the speed of the self and front vehicles, in order to increase the safety vehicle distance, Vf = Vl is assumed when calculating the safety distance. The safety distance of each vehicle speed under different road conditions can be obtained from Eq 1, and the results are shown in Table 4.

**Table 4:** Relationship between vehicle speed and safety distance under various road conditions.

Speed(km/h)	20	25	30	35	40	45	50	55	60
Road conditions									
Dry	12.8m	16.0m	19.2m	22.4m	25.6m	28.7m	31.9m	35.1m	38.3m
Wet	14.8m	19.1m	23.7m	28.5m	33.6m	38.9m	44.4m	50.3m	56.3m
Snow	18.8m	25.3m	32.7m	40.7m	49.6m	59.1m	69.5m	80.5m	92.3m

**Fig 6:**
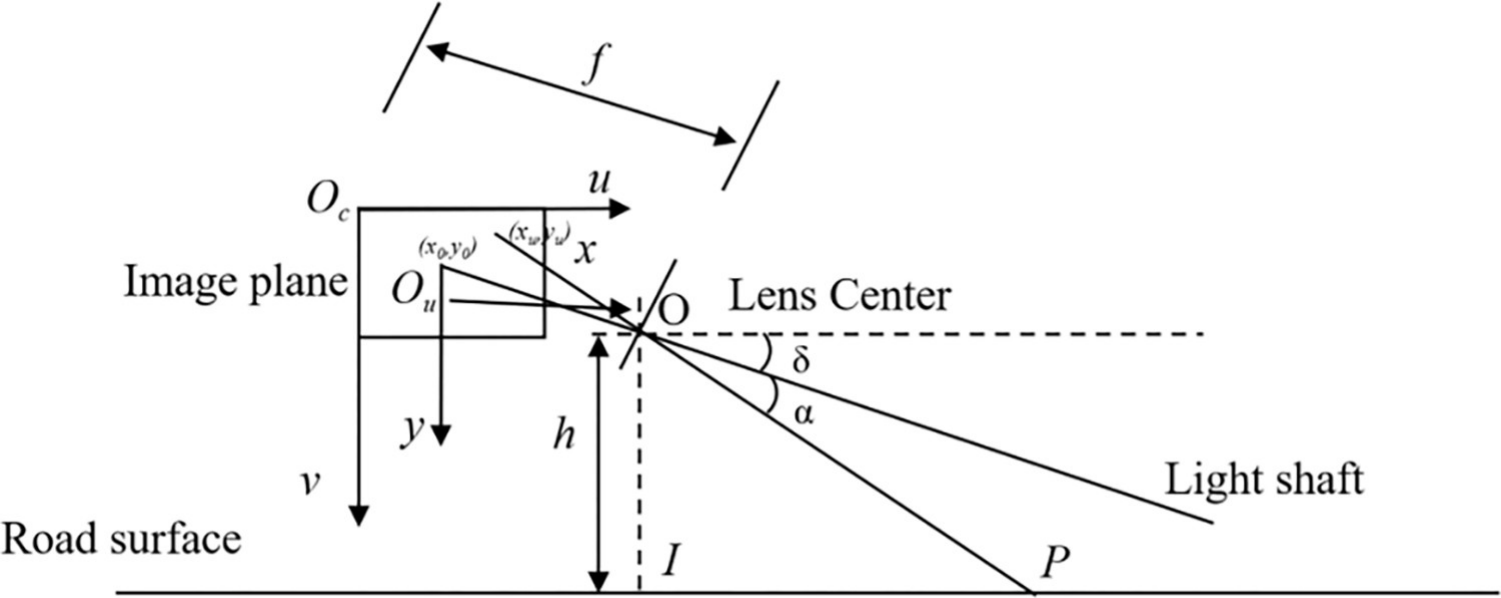
Image conversion schematic.

The actual safety distance is obtained according to Eq 1. To plot the corresponding danger area on the camera, the actual distance needs to be converted into a pixel distance on the image. In this paper, a monocular camera is used to acquire image data, and its ranging principle is the small-aperture imaging principle. In this paper, we use geometric relational projections to derive the pixel distances of images. As shown in Fig 6, set the camera pitch angle is *δ*, the optical axis and into the camera image coordinate system intersects the image physical coordinate system plane origin *O*_*u*_. *O*_*c*_ is the pixel coordinate system origin, O point is the camera lens position, the height between the camera lens and the ground is OI, IP is the safety distance, P point projection to the camera image coordinate system coordinates of (*x*_*u*_, *y*_*u*_), PP’ and the angle of the optical axis is *α*, the camera focal length is f.

According to the geometric relationship in the figure, it is obtained that:

$$\left\{ \begin{array}{l} IP=OI*\tan∠POI\\ ∠POI=\frac{\pi }{2}-\alpha -\delta \\ \tan\alpha =\frac{y_{u}}{f}\\ y_{u}=(v-v_{0})*d_{y} \end{array}\right.$$ (2)

where *v*_0_ represents the coordinates of the image physical coordinate system corresponding to the image pixel coordinate system, and *d*_*y*_ is the unit pixel distance in the y-axis direction on the camera’s light-sensing chip. The relationship between the vertical coordinate v and IP in the image pixel coordinate system can be seen from Eq 2 as follows:

$$v=v_{0}+\frac{f}{d_{y}}*\tan[\mathrm{arccot}(\frac{IP}{h})-\sigma ]$$ (3)

After obtaining the vertical coordinates, the next step is to obtain the horizontal coordinates in the pixel coordinate system. Let the width of the vehicle be *W*, then the safe distance width is extended to *W+d*, where d is the coefficient of following the actual road conditions change. According to the similar triangle principle of the monocular camera, it is known that the horizontal coordinates u_1_, u_2_ in the pixel coordinate system are calculated as follows:

$$\left\{ \begin{array}{l} u_{1}=u_{center}-\frac{(W+d)*f}{2IP}\\ u_{2}=u_{center}+\frac{(W+d)*f}{2IP} \end{array}\right.$$ (4)

where *u*_*center*_ represents the horizontal coordinate corresponding to the center of the screen in the image pixel coordinates.

### 4.3 Vehicle collision warning strategy

It is assumed that in the world coordinate system, the camera position coincides with the symmetry axis of the vehicle, and in the image coordinate system, the bottom of the target detection frame is exactly in contact with the ground. Then set the coordinates of the lower left corner of the early warning area as (u_1_, *v*_1_) and the coordinates of the lower right corner as (u_2_, *v*_2_). u_1_, *v*_1_ and u_2_, *v*_2_ coordinates can be derived from Eq 3 and Eq 4.

**Fig 7:**
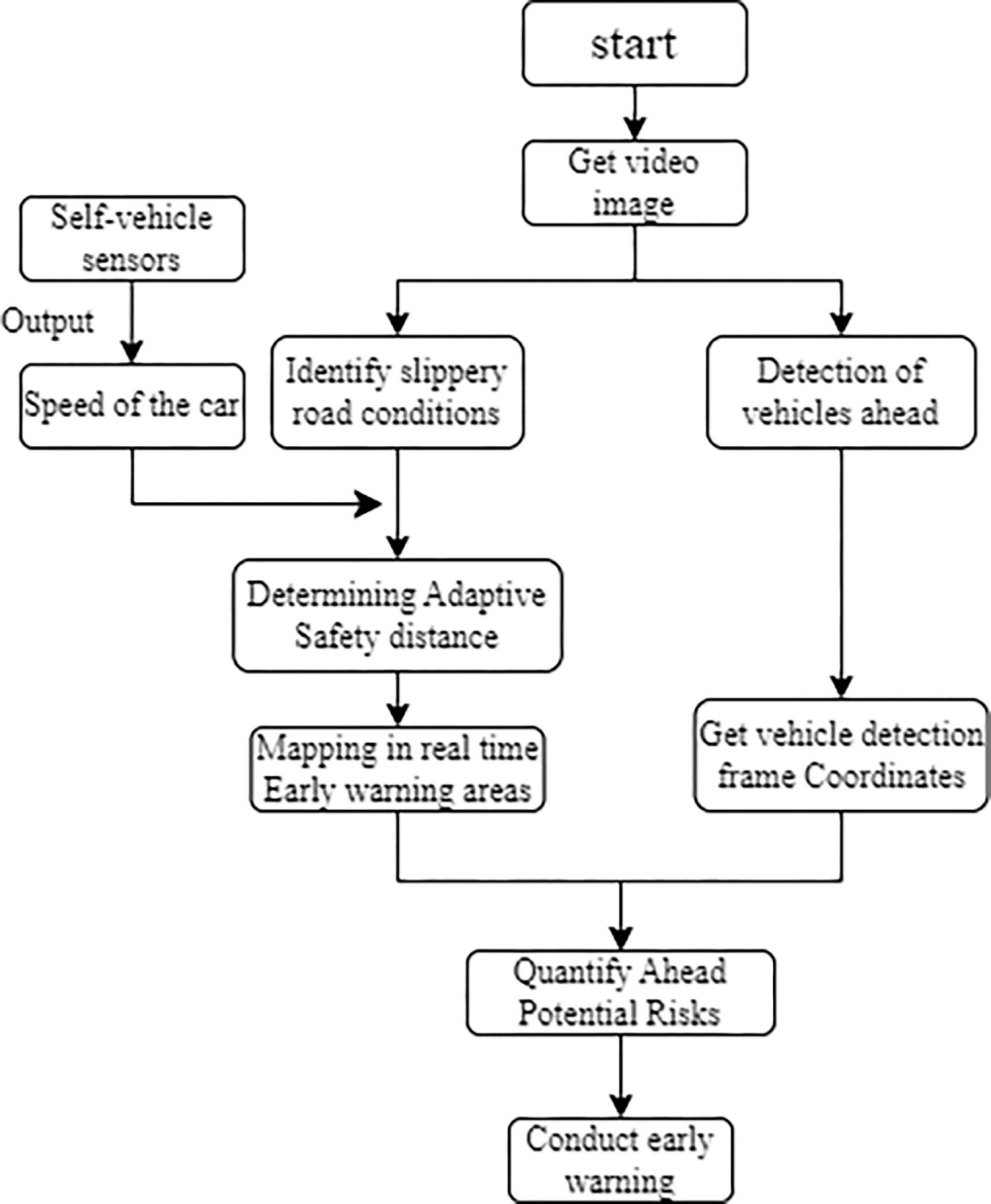
Flow chart of warning strategy.

In this paper, we predict a warning area in front of the vehicle that changes dynamically with the speed of the vehicle and the slippery states of road obtained from image detection. The early warning strategy is divided into several situations that drivers may encounter, such as vehicles in front are too close, vehicles is coming from the left and vehicles is coming from the right. The specific warning method is to get the coordinates of the lower left and lower right corners of the vehicle detection frame and determine whether it is in the warning area. If the coordinates of the lower left corner and the coordinates of the lower right corner are both within the warning area, it is considered that the vehicle ahead is too close; if the coordinates of the lower left corner are not within the warning area and the coordinates of the lower right corner are within the warning area, it is considered that the vehicle ahead is coming from the right side; if the coordinates of the lower right corner are not within the warning area and the coordinates of the lower left corner are within the warning area, it is considered that the vehicle ahead is coming from the left side. The specific warning process algorithm is shown in Fig 7.

## 5. Results

**Fig 8:**
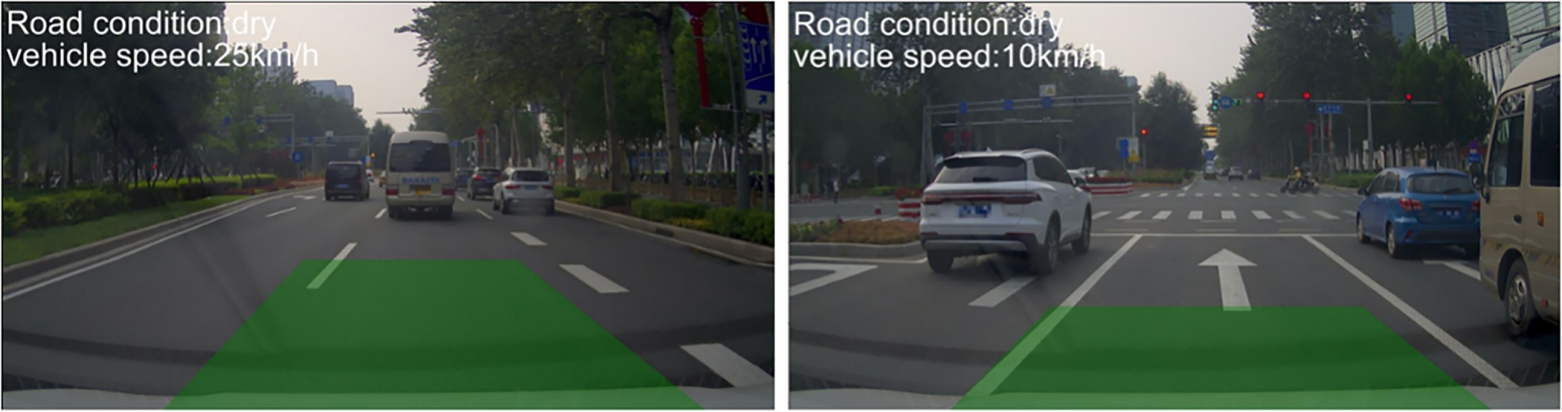
Schematic diagram of danger zones at speeds of 25km/h (Left) and 10km/h (Right).

**Fig 9:**
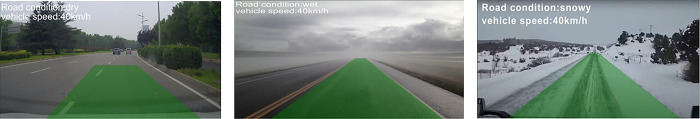
Schematic diagram of dangerous areas for different road surface conditions.

To verify the effectiveness of the adaptive safe distance model proposed in this paper, the algorithm was tested under different road conditions, and the experimental results were shown in Figs 8 and 9. As shown in Fig 8, when the current vehicle speed is 25 km/h and the road surface is dry, the minimum safe warning distance is 16 meters; when the vehicle speed decreases to 10 km/h, the minimum safe warning distance is updated to 2.8 meters. Fig 9 shows that at the same vehicle speed (both 40 km/h), when the road surface is dry, the length of the dangerous area is 25.6 meters; when the road surface is wet after rain, the length of the dangerous area is 33.6 meters; and when the road surface is snowy, the length of the dangerous area is 49.6 meters. It can be seen that the algorithm is capable of constructing an adaptive safe distance for collision warning under different vehicle speeds and road conditions.

**Fig 10:**
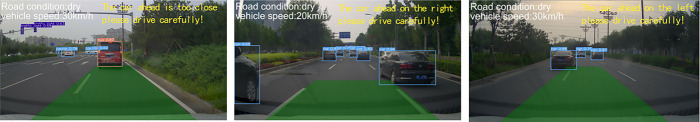
Schematic Diagram of Warning Effects: (Left) represents too close distance to the preceding vehicle, (Middle) represents an approaching vehicle from the right, and (Right) represents an approaching vehicle from the left.

Based on the aforementioned experiments, warning experiments for potential collisions of vehicles on actual roads were conducted according to the positional relationship between the warning areas and the vehicles. The experimental results are shown in Fig 10. The three images below respectively show the warning effects for situations where the vehicle distance is too close, there is an approaching vehicle from the right ahead, and there is an approaching vehicle from the left ahead.

The principle of traditional forward collision warning systems mainly relied on sensor data to estimate the distance and relative speed between the host vehicle and the obstacle ahead. Based on the obtained distance and speed, the system calculated the time to collision (TTC) between the vehicle and the obstacle ahead, and compared the calculated value with a preset threshold to issue warnings [24–26]. To further verify the warning effectiveness of the algorithm proposed in this paper, driving videos from different road sections and surface conditions were selected for experiments and the results of the warning effectiveness were shown in Table 5 compared with traditional warning algorithms. The experimental results indicate that the accuracy rate of traditional warnings is 87.31%, with a false alarm rate of 12.69%; while the accuracy rate of the warning system proposed in this paper is 94.03%, with a false alarm rate of 5.97%. The overall accuracy rate has improved by 6.72%, and the false alarm rate for predicting approaching vehicles from both sides has decreased by 16.67% compared to traditional warnings.

**Table 5:** Comparative effect of different vehicle early warning systems.

	Type of warning	Too close distance to the preceding vehicle	An approaching vehicle from the left	An approaching vehicle from the right
	True value	86	23	25
**traditional collision warning systems**	False warning	4	6	7
	False warning	82	17	18
**collision warning systems based on road condition recognition**	False warning	3	2	3
	False warning	83	21	22

## 6. Conclusions

This paper realizes vehicle collision warning based on the recognition of slippery road surface and vehicle detection algorithm, which solves the problems of weak environmental adaptability and low detection accuracy of the current research algorithm to a certain extent. In this paper the on-board camera is used to monitor the environment and road conditions in front of the vehicle in real time, and a residual network model FS-ResNet50 is proposed, which integrates SE attention mechanism and multi-level feature information based on the traditional ResNet50 model. The FS-ResNet50 model is used to identify the slippery states of the current road, such as dry and wet. Secondly, the yolov5 algorithm is used to detect the position of the vehicle in front, and a driving safety distance model with adaptive traffic environment characteristics is established based on different road environments and driving conditions, and an early warning area that dynamically changed with the speed and the slippery states of the current road is generated. Finally, according to the relationship between the warning area and the position of the vehicle, the possible collision is predicted and timely warned. The experimental results show that the algorithm in this paper can effectively generate an early warning area that dynamically changes with the speed of the self-vehicle and the slippery states of the road, which has high application value. In terms of early warning, this algorithm is more accurate than the traditional collision warning algorithm, and the overall warning accuracy was improved by 6.72%, and the warning false alarm rate for oncoming traffic on both sides was reduced by 16.67%.

In conclusion, this algorithm can provide better safety for drivers and can play an important role in reducing the occurrence rate of traffic accidents and protecting the lives of drivers. These future research directions include further considering the variation of friction coefficient between tires and the road surface under different conditions, and developing an algorithm based on the parallel characteristics of lane lines to adapt to different camera installation locations and driving environments.

## Supporting information

S1 Dataset Experimental data set. All collected images were collected together, labeled and summarized one by one, and resulting classification results were roughly classified into three major categories: dry, wet and snowy.
(ZIP)
