# Supplementary figures and images for: Research on recognition of slippery road surface and collision warning system based on deep learning (part 2 of 2)
Source: PLoS One. 2024 Nov 11;19(11):e0310858. doi: 10.1371/journal.pone.0310858 (PMC11554202; doi:10.1371/journal.pone.0310858)

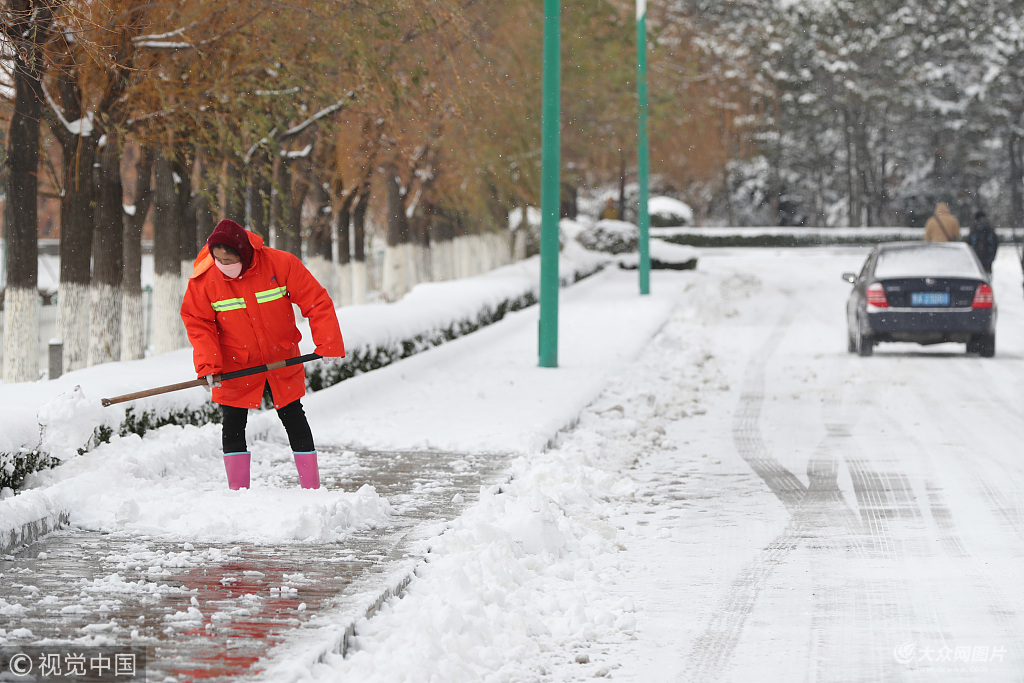

Supplement: S1 Dataset — All collected images were collected together, labeled and summarized one by one, and resulting classification results were roughly classified into three major categories: dry, wet and snowy. (ZIP) [file pone.0310858.s001.zip › weather1_data/snow_road/1331.jpg]

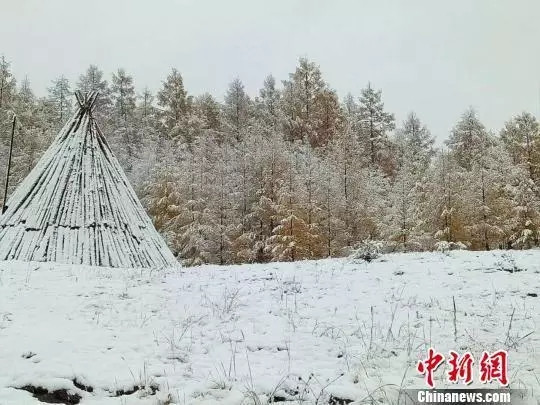

Supplement: S1 Dataset — All collected images were collected together, labeled and summarized one by one, and resulting classification results were roughly classified into three major categories: dry, wet and snowy. (ZIP) [file pone.0310858.s001.zip › weather1_data/snow_road/1332.jpg]

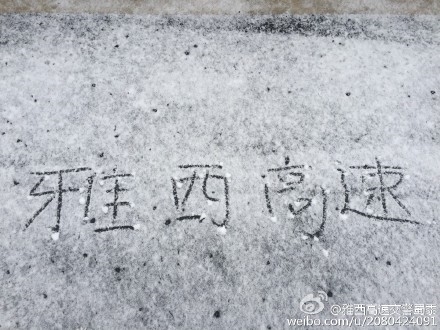

Supplement: S1 Dataset — All collected images were collected together, labeled and summarized one by one, and resulting classification results were roughly classified into three major categories: dry, wet and snowy. (ZIP) [file pone.0310858.s001.zip › weather1_data/snow_road/1338.jpg]

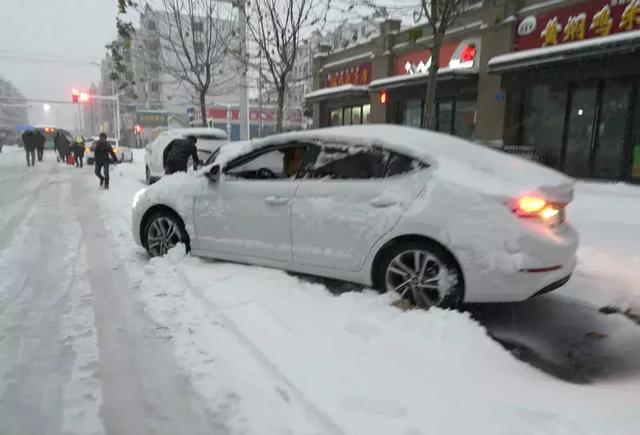

Supplement: S1 Dataset — All collected images were collected together, labeled and summarized one by one, and resulting classification results were roughly classified into three major categories: dry, wet and snowy. (ZIP) [file pone.0310858.s001.zip › weather1_data/snow_road/1441.jpg]

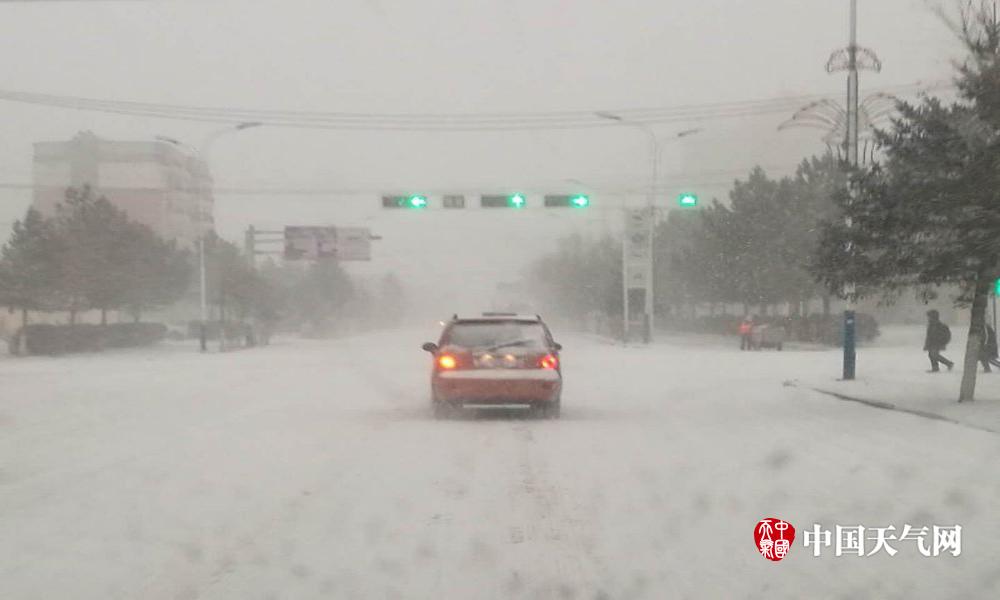

Supplement: S1 Dataset — All collected images were collected together, labeled and summarized one by one, and resulting classification results were roughly classified into three major categories: dry, wet and snowy. (ZIP) [file pone.0310858.s001.zip › weather1_data/snow_road/1444.jpg]

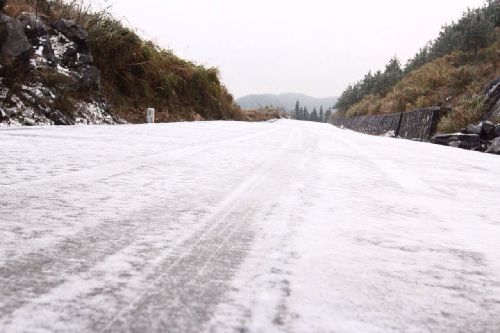

Supplement: S1 Dataset — All collected images were collected together, labeled and summarized one by one, and resulting classification results were roughly classified into three major categories: dry, wet and snowy. (ZIP) [file pone.0310858.s001.zip › weather1_data/snow_road/1445.jpg]

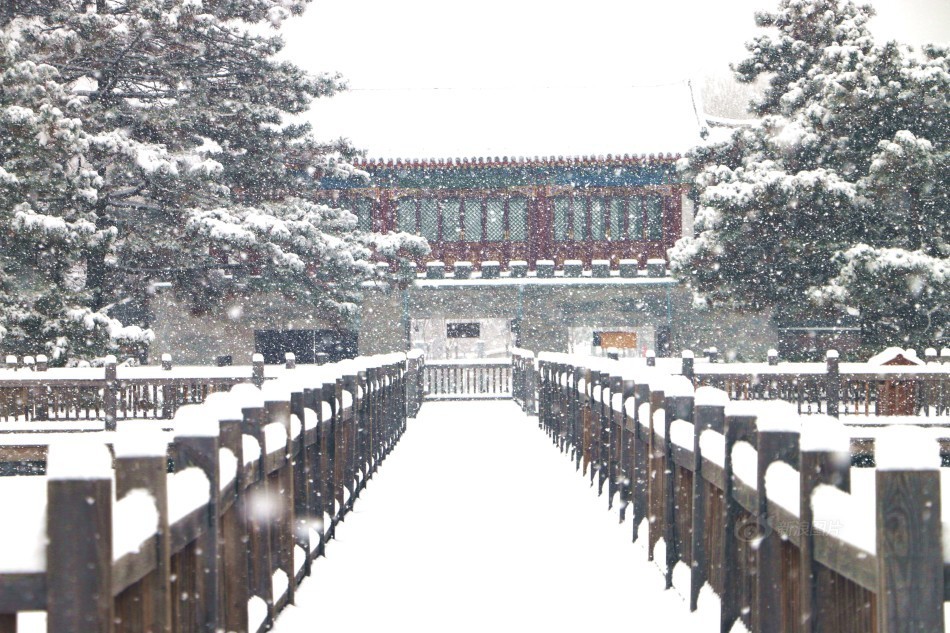

Supplement: S1 Dataset — All collected images were collected together, labeled and summarized one by one, and resulting classification results were roughly classified into three major categories: dry, wet and snowy. (ZIP) [file pone.0310858.s001.zip › weather1_data/snow_road/1446.jpg]

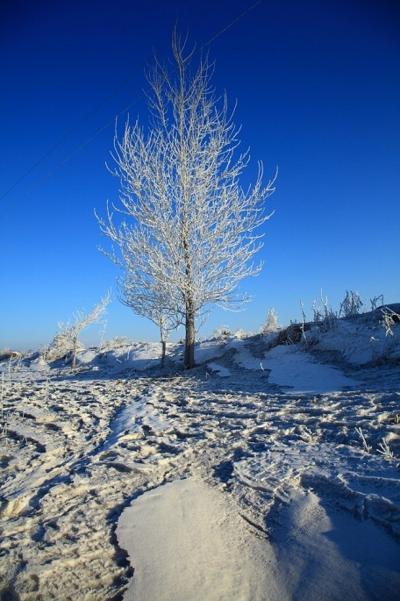

Supplement: S1 Dataset — All collected images were collected together, labeled and summarized one by one, and resulting classification results were roughly classified into three major categories: dry, wet and snowy. (ZIP) [file pone.0310858.s001.zip › weather1_data/snow_road/snow_00002.jpg]

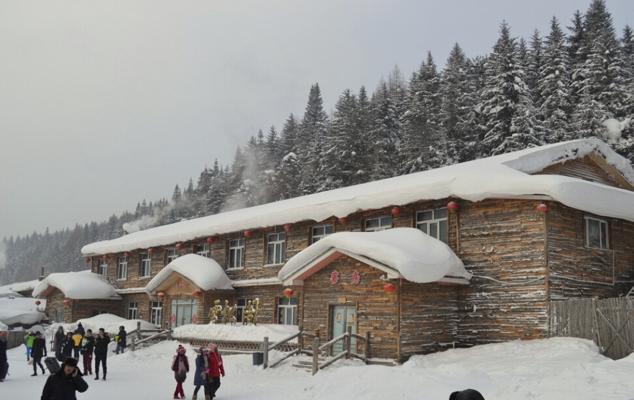

Supplement: S1 Dataset — All collected images were collected together, labeled and summarized one by one, and resulting classification results were roughly classified into three major categories: dry, wet and snowy. (ZIP) [file pone.0310858.s001.zip › weather1_data/snow_road/snow_00012.jpg]

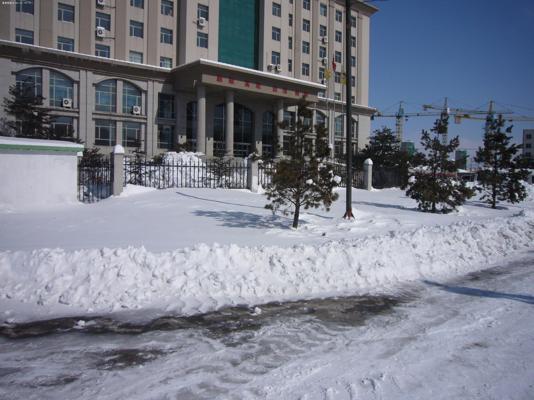

Supplement: S1 Dataset — All collected images were collected together, labeled and summarized one by one, and resulting classification results were roughly classified into three major categories: dry, wet and snowy. (ZIP) [file pone.0310858.s001.zip › weather1_data/snow_road/snow_00017.jpg]

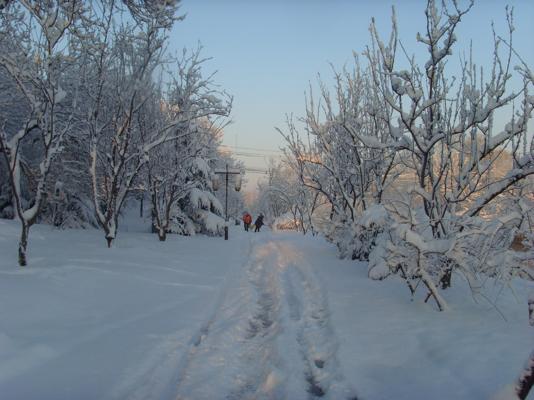

Supplement: S1 Dataset — All collected images were collected together, labeled and summarized one by one, and resulting classification results were roughly classified into three major categories: dry, wet and snowy. (ZIP) [file pone.0310858.s001.zip › weather1_data/snow_road/snow_00032.jpg]

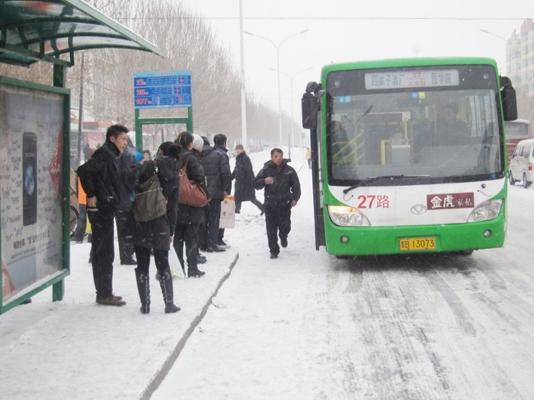

Supplement: S1 Dataset — All collected images were collected together, labeled and summarized one by one, and resulting classification results were roughly classified into three major categories: dry, wet and snowy. (ZIP) [file pone.0310858.s001.zip › weather1_data/snow_road/snow_00053.jpg]

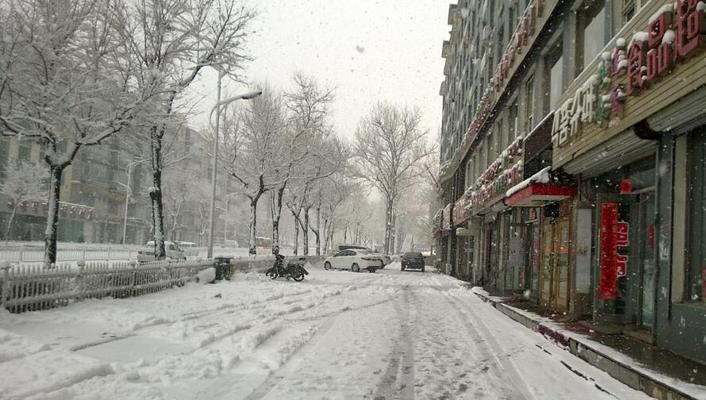

Supplement: S1 Dataset — All collected images were collected together, labeled and summarized one by one, and resulting classification results were roughly classified into three major categories: dry, wet and snowy. (ZIP) [file pone.0310858.s001.zip › weather1_data/snow_road/snow_00056.jpg]

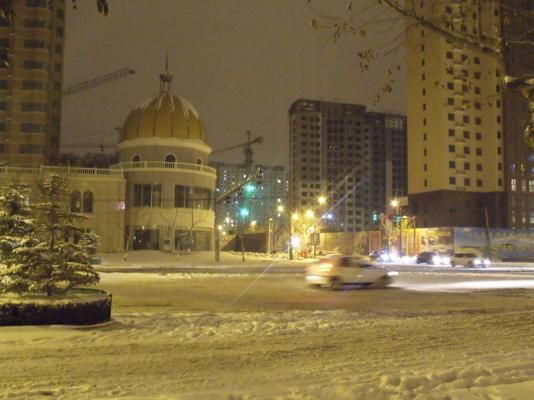

Supplement: S1 Dataset — All collected images were collected together, labeled and summarized one by one, and resulting classification results were roughly classified into three major categories: dry, wet and snowy. (ZIP) [file pone.0310858.s001.zip › weather1_data/snow_road/snow_00096.jpg]

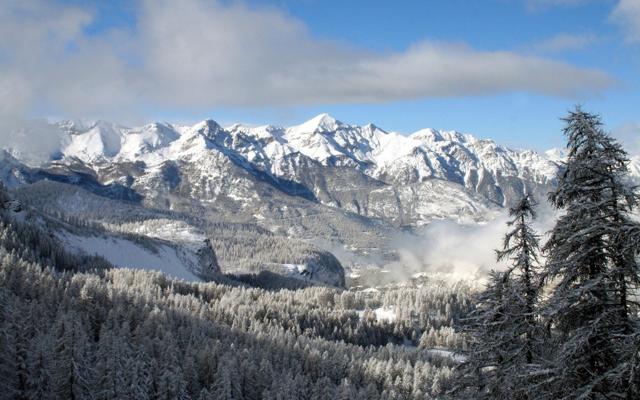

Supplement: S1 Dataset — All collected images were collected together, labeled and summarized one by one, and resulting classification results were roughly classified into three major categories: dry, wet and snowy. (ZIP) [file pone.0310858.s001.zip › weather1_data/snow_road/snow_00122.jpg]

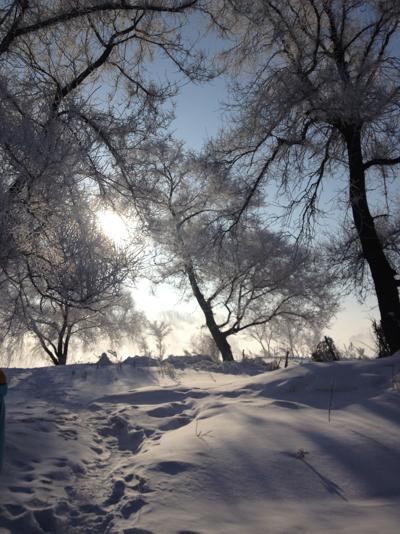

Supplement: S1 Dataset — All collected images were collected together, labeled and summarized one by one, and resulting classification results were roughly classified into three major categories: dry, wet and snowy. (ZIP) [file pone.0310858.s001.zip › weather1_data/snow_road/snow_00126.jpg]

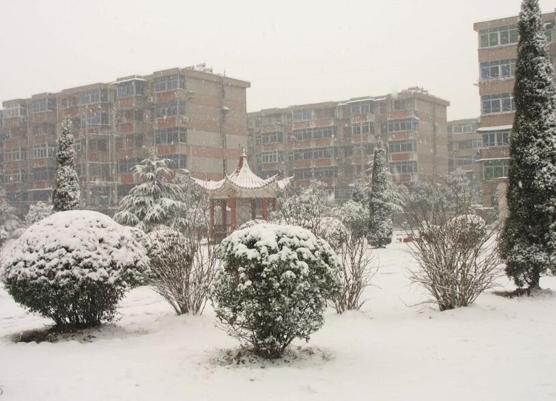

Supplement: S1 Dataset — All collected images were collected together, labeled and summarized one by one, and resulting classification results were roughly classified into three major categories: dry, wet and snowy. (ZIP) [file pone.0310858.s001.zip › weather1_data/snow_road/snow_00143.jpg]

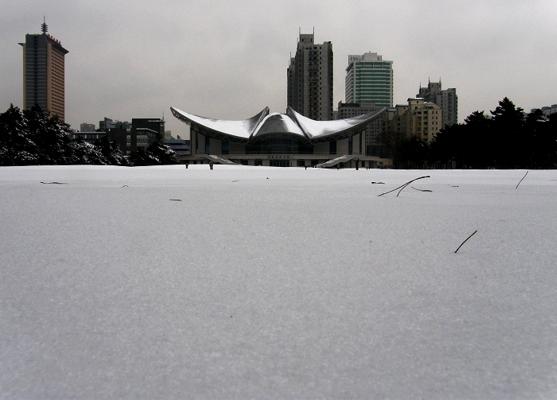

Supplement: S1 Dataset — All collected images were collected together, labeled and summarized one by one, and resulting classification results were roughly classified into three major categories: dry, wet and snowy. (ZIP) [file pone.0310858.s001.zip › weather1_data/snow_road/snow_00148.jpg]

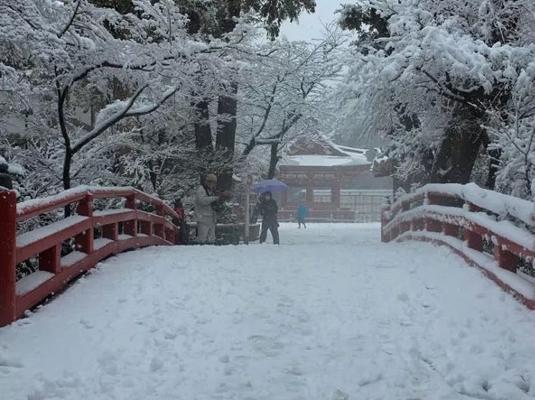

Supplement: S1 Dataset — All collected images were collected together, labeled and summarized one by one, and resulting classification results were roughly classified into three major categories: dry, wet and snowy. (ZIP) [file pone.0310858.s001.zip › weather1_data/snow_road/snow_00154.jpg]

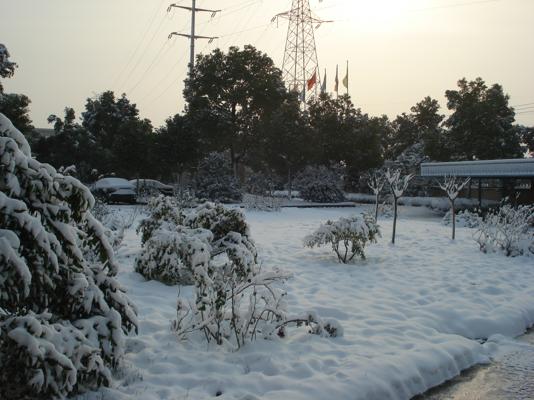

Supplement: S1 Dataset — All collected images were collected together, labeled and summarized one by one, and resulting classification results were roughly classified into three major categories: dry, wet and snowy. (ZIP) [file pone.0310858.s001.zip › weather1_data/snow_road/snow_00164.jpg]

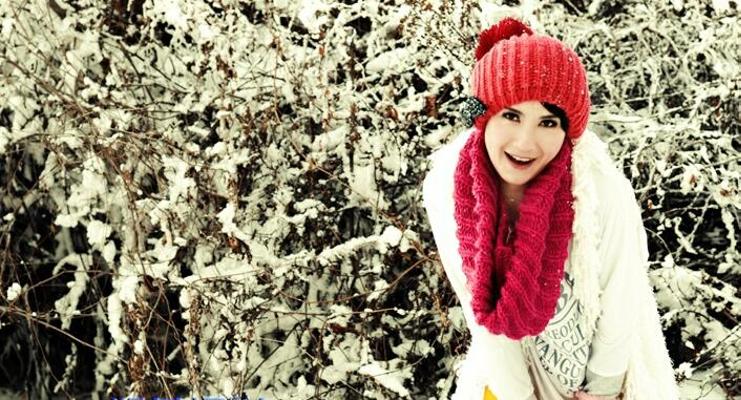

Supplement: S1 Dataset — All collected images were collected together, labeled and summarized one by one, and resulting classification results were roughly classified into three major categories: dry, wet and snowy. (ZIP) [file pone.0310858.s001.zip › weather1_data/snow_road/snow_00166.jpg]

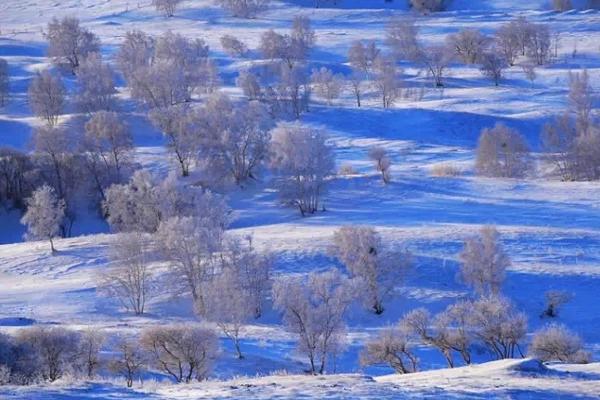

Supplement: S1 Dataset — All collected images were collected together, labeled and summarized one by one, and resulting classification results were roughly classified into three major categories: dry, wet and snowy. (ZIP) [file pone.0310858.s001.zip › weather1_data/snow_road/snow_00167.jpg]

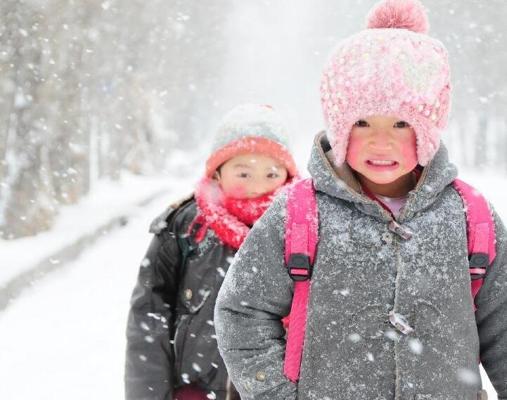

Supplement: S1 Dataset — All collected images were collected together, labeled and summarized one by one, and resulting classification results were roughly classified into three major categories: dry, wet and snowy. (ZIP) [file pone.0310858.s001.zip › weather1_data/snow_road/snow_00257.jpg]

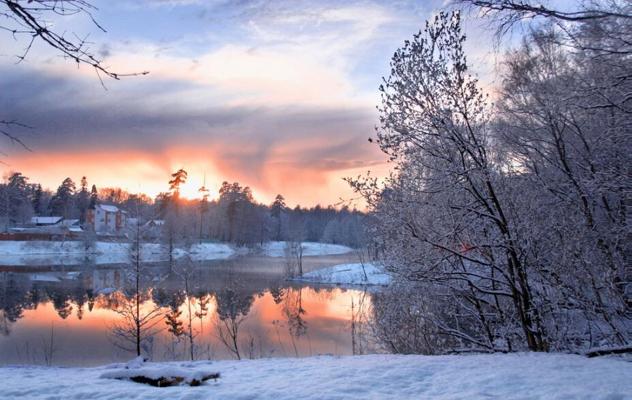

Supplement: S1 Dataset — All collected images were collected together, labeled and summarized one by one, and resulting classification results were roughly classified into three major categories: dry, wet and snowy. (ZIP) [file pone.0310858.s001.zip › weather1_data/snow_road/snow_00259.jpg]

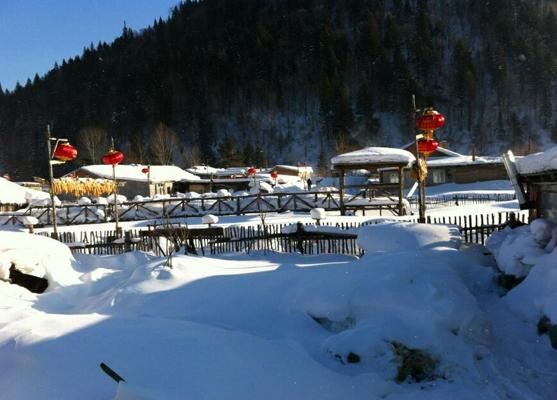

Supplement: S1 Dataset — All collected images were collected together, labeled and summarized one by one, and resulting classification results were roughly classified into three major categories: dry, wet and snowy. (ZIP) [file pone.0310858.s001.zip › weather1_data/snow_road/snow_00274.jpg]

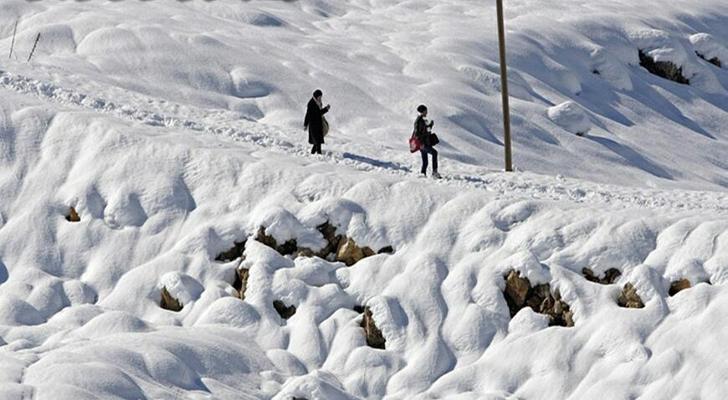

Supplement: S1 Dataset — All collected images were collected together, labeled and summarized one by one, and resulting classification results were roughly classified into three major categories: dry, wet and snowy. (ZIP) [file pone.0310858.s001.zip › weather1_data/snow_road/snow_00277.jpg]

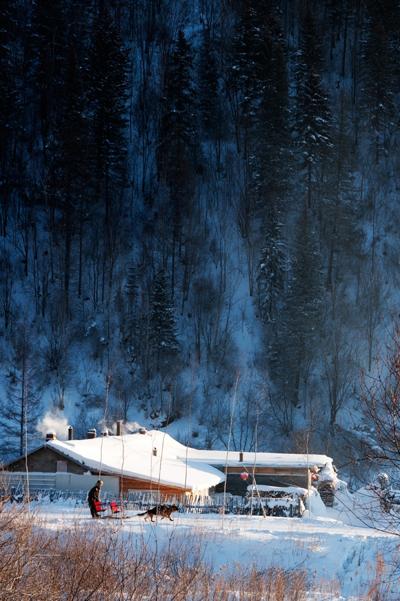

Supplement: S1 Dataset — All collected images were collected together, labeled and summarized one by one, and resulting classification results were roughly classified into three major categories: dry, wet and snowy. (ZIP) [file pone.0310858.s001.zip › weather1_data/snow_road/snow_00278.jpg]

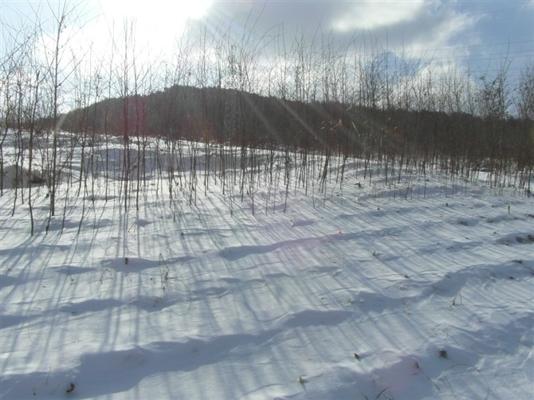

Supplement: S1 Dataset — All collected images were collected together, labeled and summarized one by one, and resulting classification results were roughly classified into three major categories: dry, wet and snowy. (ZIP) [file pone.0310858.s001.zip › weather1_data/snow_road/snow_00283.jpg]

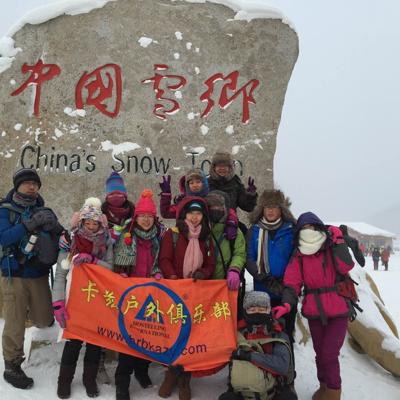

Supplement: S1 Dataset — All collected images were collected together, labeled and summarized one by one, and resulting classification results were roughly classified into three major categories: dry, wet and snowy. (ZIP) [file pone.0310858.s001.zip › weather1_data/snow_road/snow_00288.jpg]

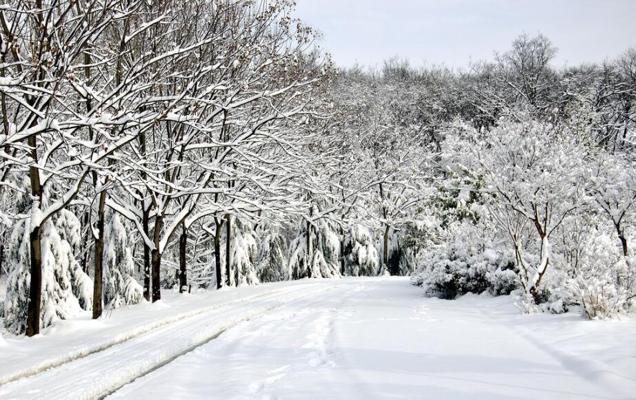

Supplement: S1 Dataset — All collected images were collected together, labeled and summarized one by one, and resulting classification results were roughly classified into three major categories: dry, wet and snowy. (ZIP) [file pone.0310858.s001.zip › weather1_data/snow_road/snow_00382.jpg]

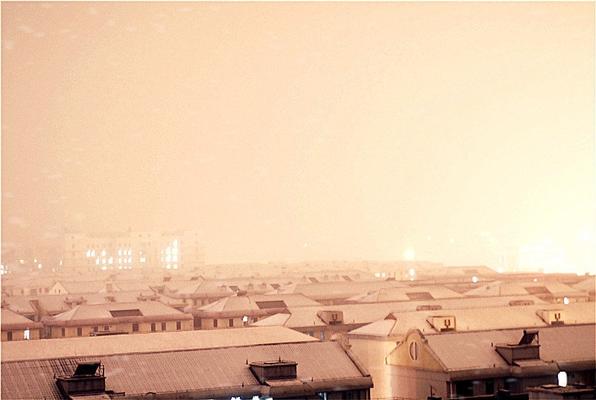

Supplement: S1 Dataset — All collected images were collected together, labeled and summarized one by one, and resulting classification results were roughly classified into three major categories: dry, wet and snowy. (ZIP) [file pone.0310858.s001.zip › weather1_data/snow_road/snow_00383.jpg]

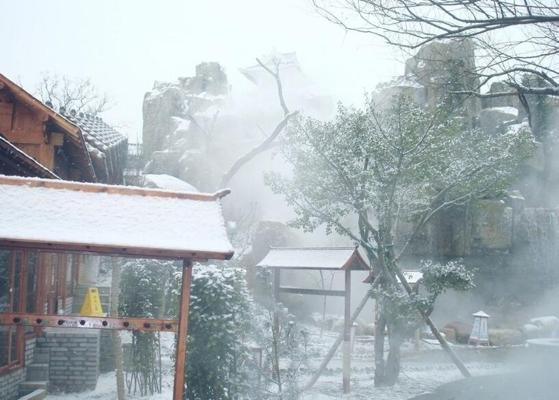

Supplement: S1 Dataset — All collected images were collected together, labeled and summarized one by one, and resulting classification results were roughly classified into three major categories: dry, wet and snowy. (ZIP) [file pone.0310858.s001.zip › weather1_data/snow_road/snow_00396.jpg]

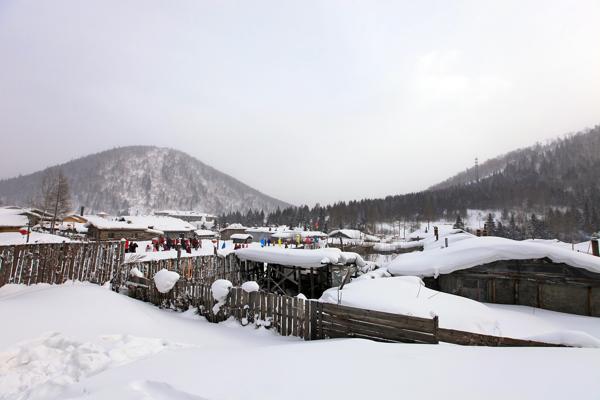

Supplement: S1 Dataset — All collected images were collected together, labeled and summarized one by one, and resulting classification results were roughly classified into three major categories: dry, wet and snowy. (ZIP) [file pone.0310858.s001.zip › weather1_data/snow_road/snow_00397.jpg]

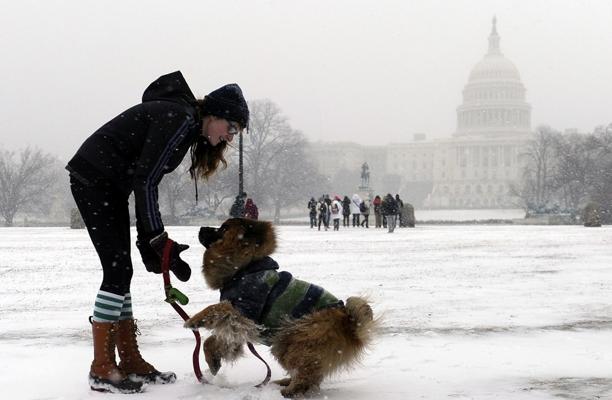

Supplement: S1 Dataset — All collected images were collected together, labeled and summarized one by one, and resulting classification results were roughly classified into three major categories: dry, wet and snowy. (ZIP) [file pone.0310858.s001.zip › weather1_data/snow_road/snow_00409.jpg]

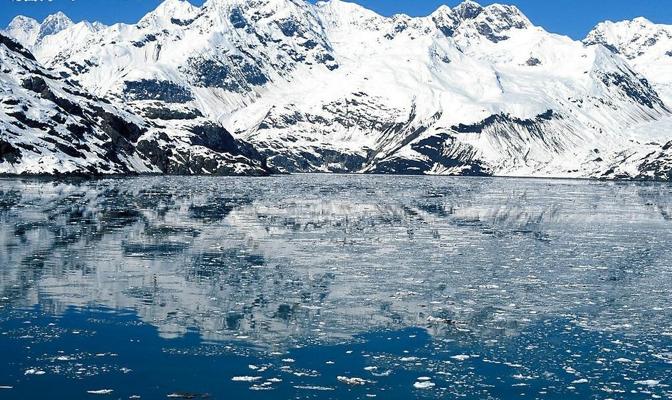

Supplement: S1 Dataset — All collected images were collected together, labeled and summarized one by one, and resulting classification results were roughly classified into three major categories: dry, wet and snowy. (ZIP) [file pone.0310858.s001.zip › weather1_data/snow_road/snow_00412.jpg]

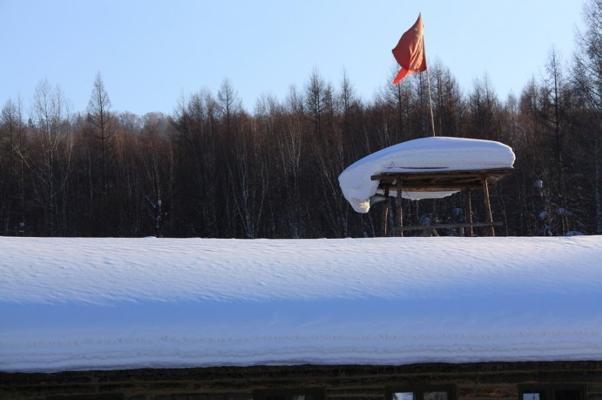

Supplement: S1 Dataset — All collected images were collected together, labeled and summarized one by one, and resulting classification results were roughly classified into three major categories: dry, wet and snowy. (ZIP) [file pone.0310858.s001.zip › weather1_data/snow_road/snow_00414.jpg]

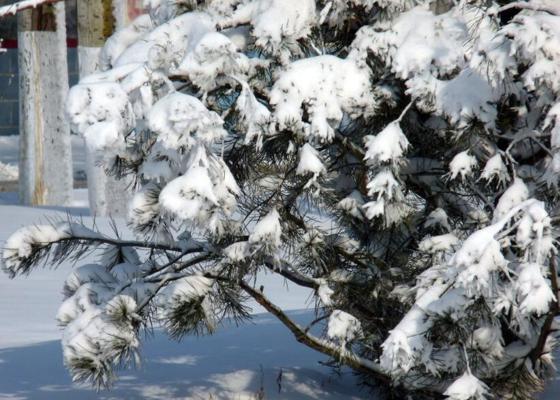

Supplement: S1 Dataset — All collected images were collected together, labeled and summarized one by one, and resulting classification results were roughly classified into three major categories: dry, wet and snowy. (ZIP) [file pone.0310858.s001.zip › weather1_data/snow_road/snow_00415.jpg]

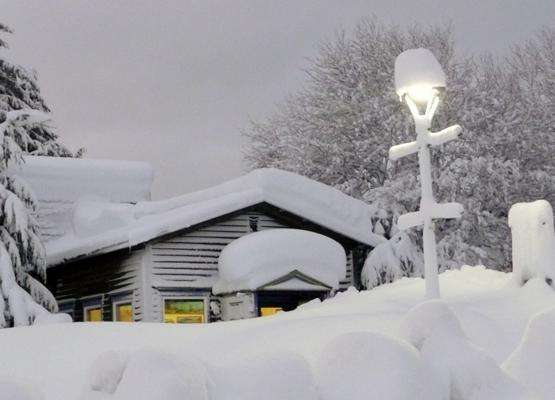

Supplement: S1 Dataset — All collected images were collected together, labeled and summarized one by one, and resulting classification results were roughly classified into three major categories: dry, wet and snowy. (ZIP) [file pone.0310858.s001.zip › weather1_data/snow_road/snow_00416.jpg]

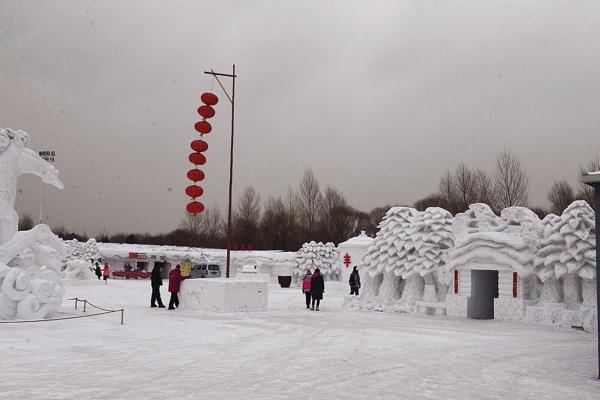

Supplement: S1 Dataset — All collected images were collected together, labeled and summarized one by one, and resulting classification results were roughly classified into three major categories: dry, wet and snowy. (ZIP) [file pone.0310858.s001.zip › weather1_data/snow_road/snow_00419.jpg]

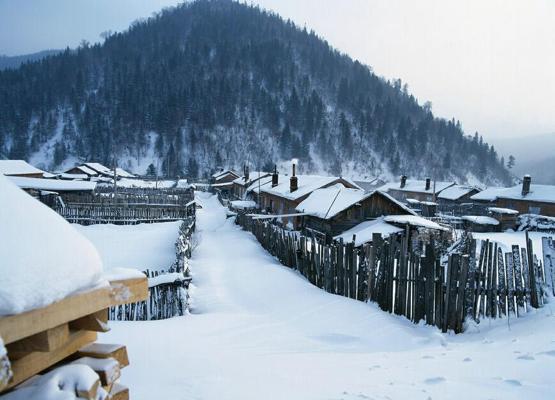

Supplement: S1 Dataset — All collected images were collected together, labeled and summarized one by one, and resulting classification results were roughly classified into three major categories: dry, wet and snowy. (ZIP) [file pone.0310858.s001.zip › weather1_data/snow_road/snow_00420.jpg]

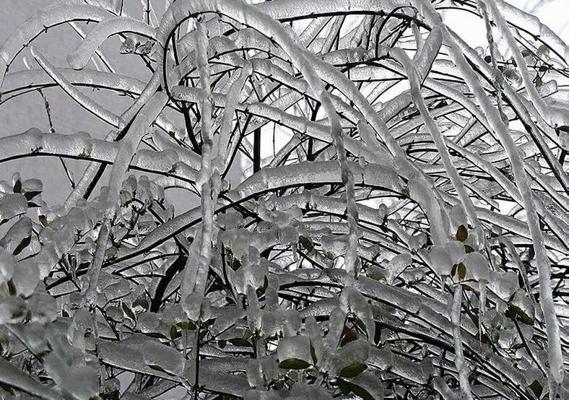

Supplement: S1 Dataset — All collected images were collected together, labeled and summarized one by one, and resulting classification results were roughly classified into three major categories: dry, wet and snowy. (ZIP) [file pone.0310858.s001.zip › weather1_data/snow_road/snow_00422.jpg]

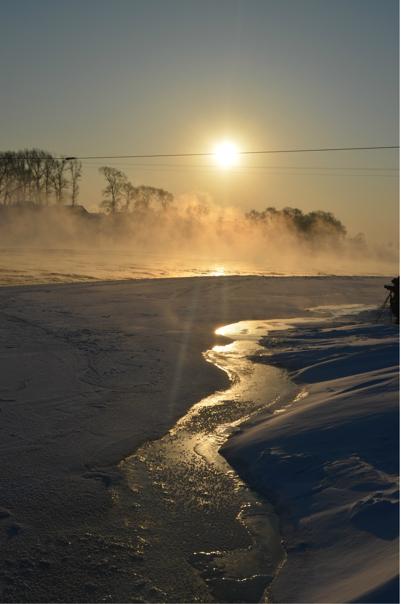

Supplement: S1 Dataset — All collected images were collected together, labeled and summarized one by one, and resulting classification results were roughly classified into three major categories: dry, wet and snowy. (ZIP) [file pone.0310858.s001.zip › weather1_data/snow_road/snow_00424.jpg]

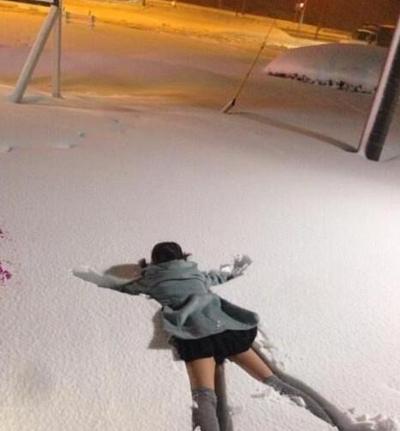

Supplement: S1 Dataset — All collected images were collected together, labeled and summarized one by one, and resulting classification results were roughly classified into three major categories: dry, wet and snowy. (ZIP) [file pone.0310858.s001.zip › weather1_data/snow_road/snow_00430.jpg]

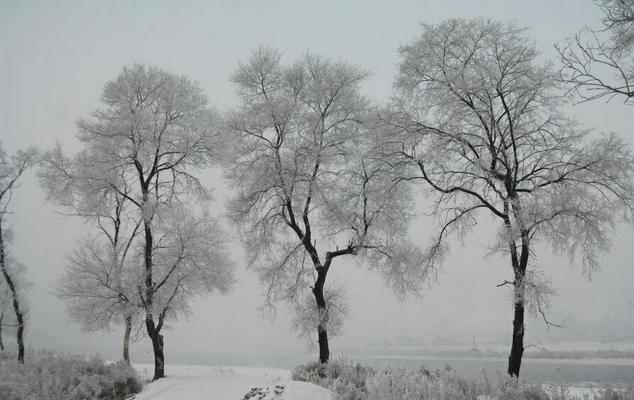

Supplement: S1 Dataset — All collected images were collected together, labeled and summarized one by one, and resulting classification results were roughly classified into three major categories: dry, wet and snowy. (ZIP) [file pone.0310858.s001.zip › weather1_data/snow_road/snow_00431.jpg]

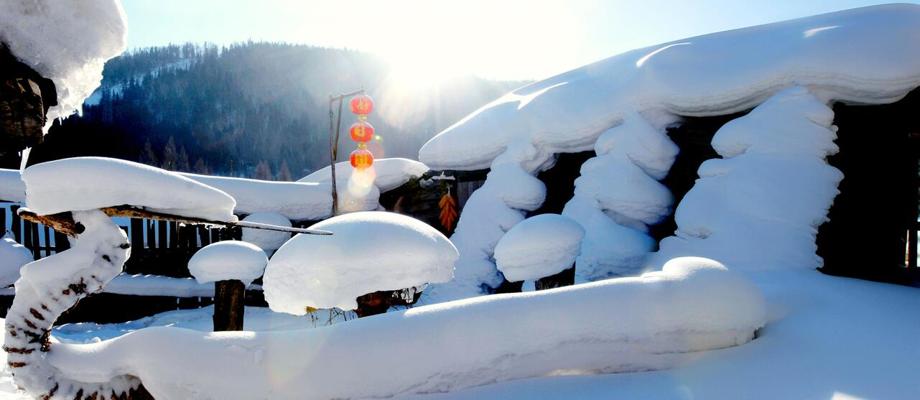

Supplement: S1 Dataset — All collected images were collected together, labeled and summarized one by one, and resulting classification results were roughly classified into three major categories: dry, wet and snowy. (ZIP) [file pone.0310858.s001.zip › weather1_data/snow_road/snow_00435.jpg]

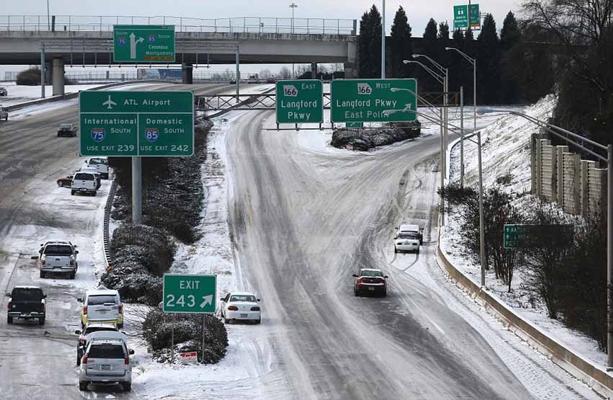

Supplement: S1 Dataset — All collected images were collected together, labeled and summarized one by one, and resulting classification results were roughly classified into three major categories: dry, wet and snowy. (ZIP) [file pone.0310858.s001.zip › weather1_data/snow_road/snow_00437.jpg]

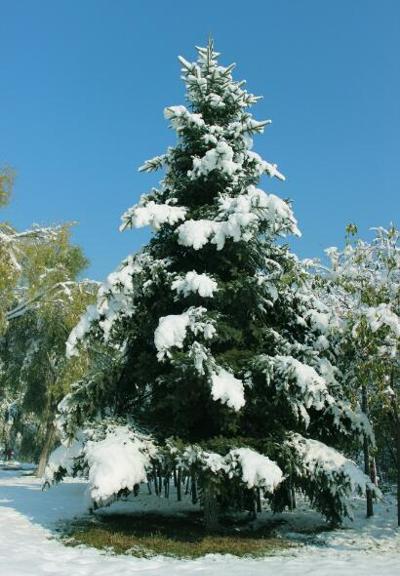

Supplement: S1 Dataset — All collected images were collected together, labeled and summarized one by one, and resulting classification results were roughly classified into three major categories: dry, wet and snowy. (ZIP) [file pone.0310858.s001.zip › weather1_data/snow_road/snow_00445.jpg]

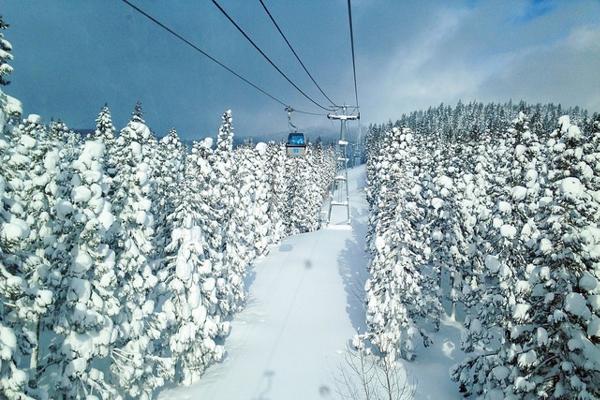

Supplement: S1 Dataset — All collected images were collected together, labeled and summarized one by one, and resulting classification results were roughly classified into three major categories: dry, wet and snowy. (ZIP) [file pone.0310858.s001.zip › weather1_data/snow_road/snow_00448.jpg]

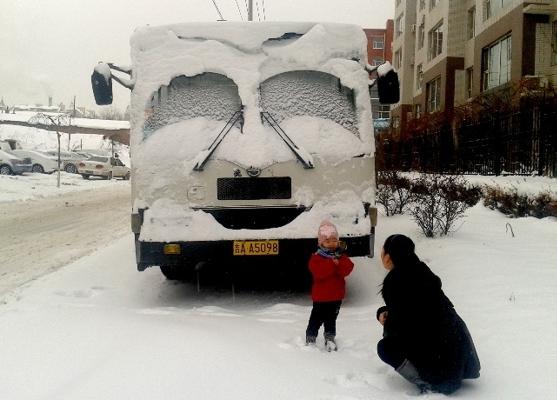

Supplement: S1 Dataset — All collected images were collected together, labeled and summarized one by one, and resulting classification results were roughly classified into three major categories: dry, wet and snowy. (ZIP) [file pone.0310858.s001.zip › weather1_data/snow_road/snow_00460.jpg]

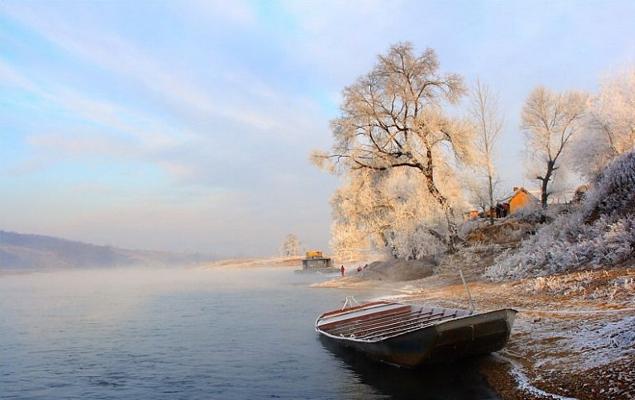

Supplement: S1 Dataset — All collected images were collected together, labeled and summarized one by one, and resulting classification results were roughly classified into three major categories: dry, wet and snowy. (ZIP) [file pone.0310858.s001.zip › weather1_data/snow_road/snow_00623.jpg]

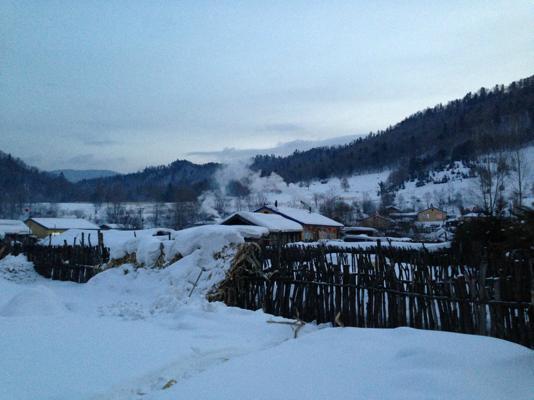

Supplement: S1 Dataset — All collected images were collected together, labeled and summarized one by one, and resulting classification results were roughly classified into three major categories: dry, wet and snowy. (ZIP) [file pone.0310858.s001.zip › weather1_data/snow_road/snow_00624.jpg]

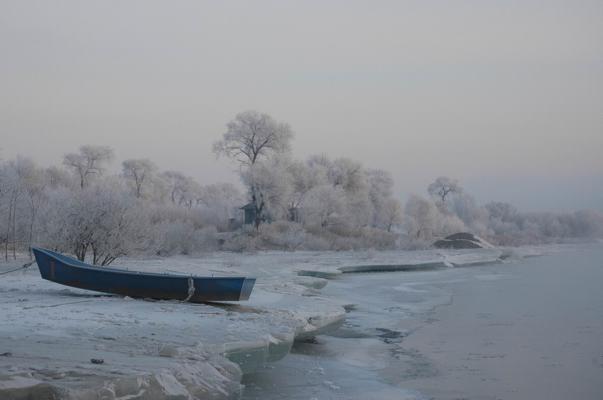

Supplement: S1 Dataset — All collected images were collected together, labeled and summarized one by one, and resulting classification results were roughly classified into three major categories: dry, wet and snowy. (ZIP) [file pone.0310858.s001.zip › weather1_data/snow_road/snow_00626.jpg]

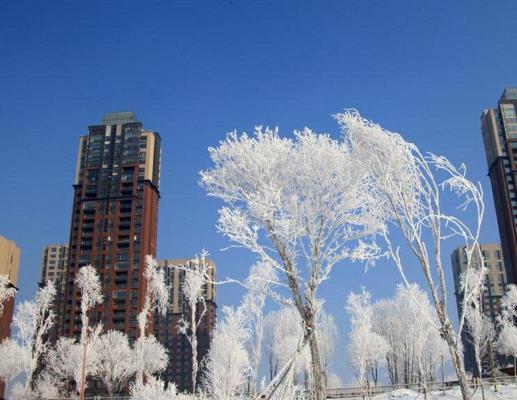

Supplement: S1 Dataset — All collected images were collected together, labeled and summarized one by one, and resulting classification results were roughly classified into three major categories: dry, wet and snowy. (ZIP) [file pone.0310858.s001.zip › weather1_data/snow_road/snow_00635.jpg]

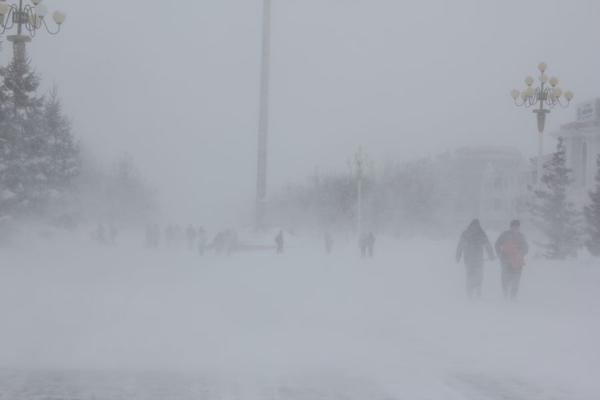

Supplement: S1 Dataset — All collected images were collected together, labeled and summarized one by one, and resulting classification results were roughly classified into three major categories: dry, wet and snowy. (ZIP) [file pone.0310858.s001.zip › weather1_data/snow_road/snow_00638.jpg]

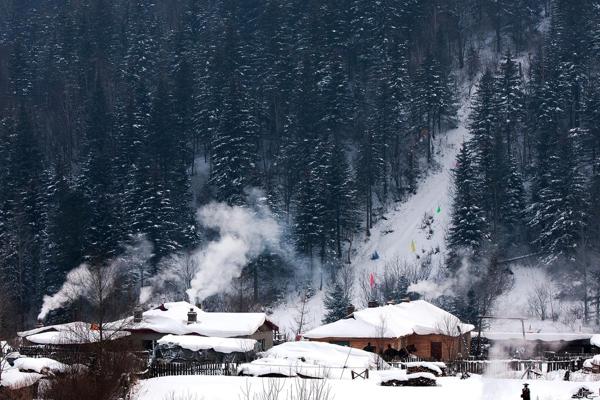

Supplement: S1 Dataset — All collected images were collected together, labeled and summarized one by one, and resulting classification results were roughly classified into three major categories: dry, wet and snowy. (ZIP) [file pone.0310858.s001.zip › weather1_data/snow_road/snow_00640.jpg]

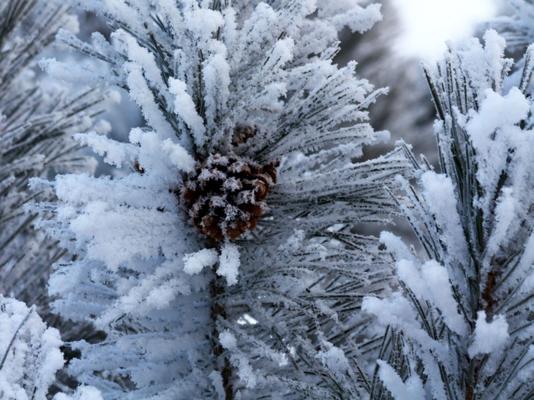

Supplement: S1 Dataset — All collected images were collected together, labeled and summarized one by one, and resulting classification results were roughly classified into three major categories: dry, wet and snowy. (ZIP) [file pone.0310858.s001.zip › weather1_data/snow_road/snow_00642.jpg]

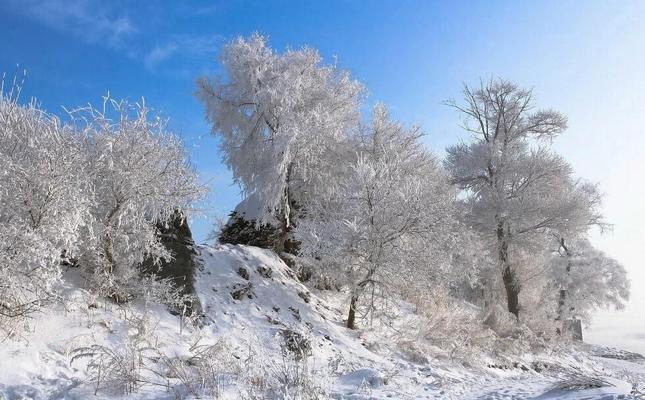

Supplement: S1 Dataset — All collected images were collected together, labeled and summarized one by one, and resulting classification results were roughly classified into three major categories: dry, wet and snowy. (ZIP) [file pone.0310858.s001.zip › weather1_data/snow_road/snow_00646.jpg]

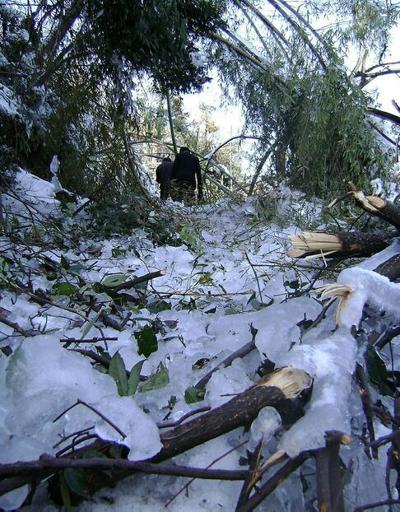

Supplement: S1 Dataset — All collected images were collected together, labeled and summarized one by one, and resulting classification results were roughly classified into three major categories: dry, wet and snowy. (ZIP) [file pone.0310858.s001.zip › weather1_data/snow_road/snow_00648.jpg]

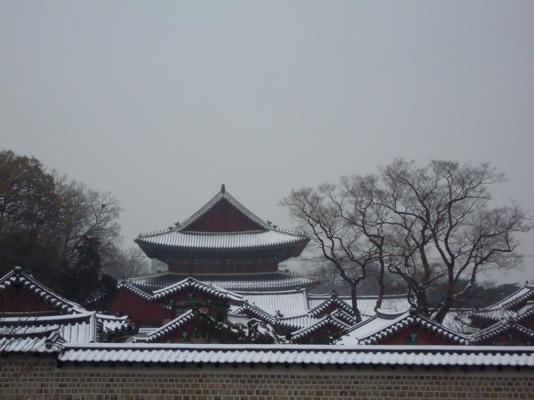

Supplement: S1 Dataset — All collected images were collected together, labeled and summarized one by one, and resulting classification results were roughly classified into three major categories: dry, wet and snowy. (ZIP) [file pone.0310858.s001.zip › weather1_data/snow_road/snow_00672.jpg]

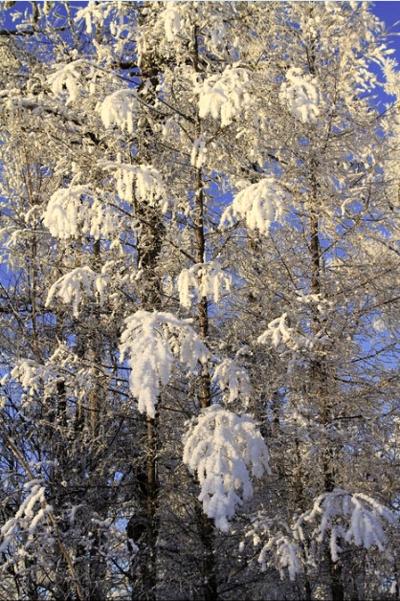

Supplement: S1 Dataset — All collected images were collected together, labeled and summarized one by one, and resulting classification results were roughly classified into three major categories: dry, wet and snowy. (ZIP) [file pone.0310858.s001.zip › weather1_data/snow_road/snow_00674.jpg]

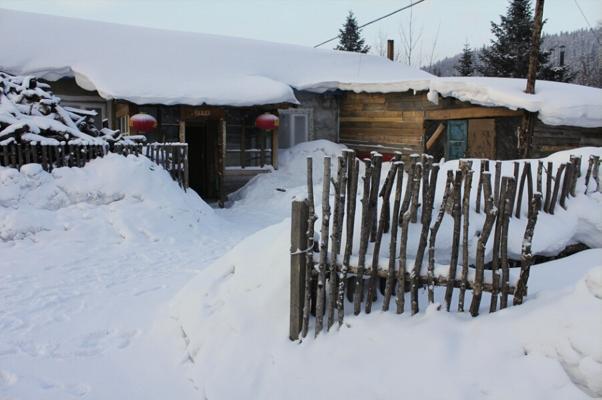

Supplement: S1 Dataset — All collected images were collected together, labeled and summarized one by one, and resulting classification results were roughly classified into three major categories: dry, wet and snowy. (ZIP) [file pone.0310858.s001.zip › weather1_data/snow_road/snow_00675.jpg]

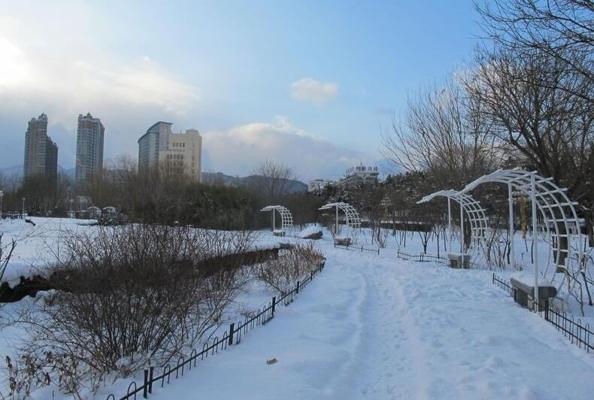

Supplement: S1 Dataset — All collected images were collected together, labeled and summarized one by one, and resulting classification results were roughly classified into three major categories: dry, wet and snowy. (ZIP) [file pone.0310858.s001.zip › weather1_data/snow_road/snow_00685.jpg]

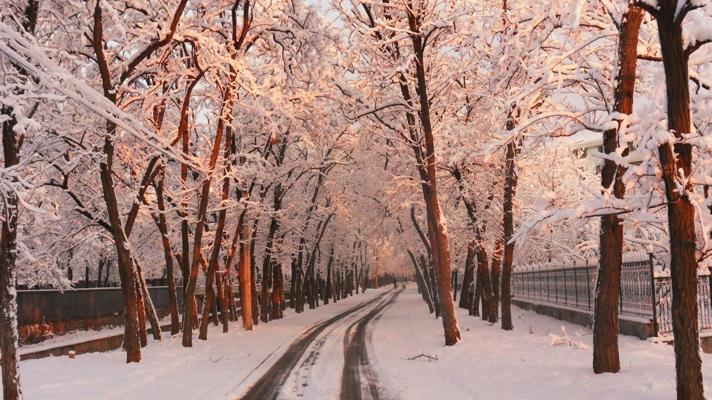

Supplement: S1 Dataset — All collected images were collected together, labeled and summarized one by one, and resulting classification results were roughly classified into three major categories: dry, wet and snowy. (ZIP) [file pone.0310858.s001.zip › weather1_data/snow_road/snow_00687.jpg]

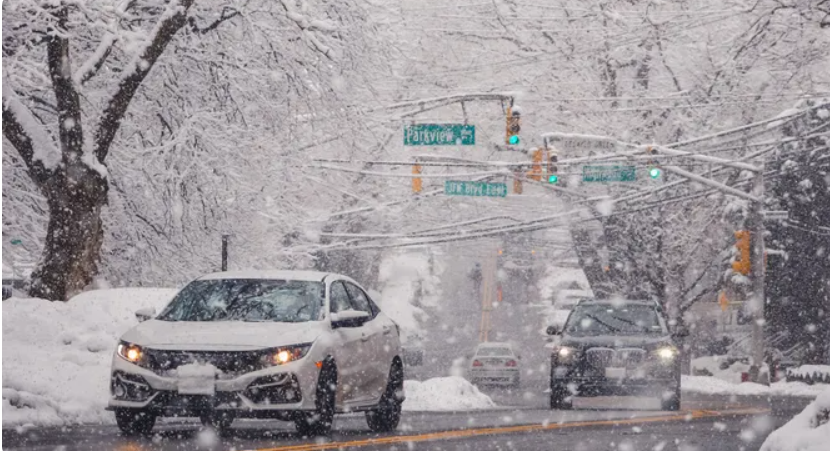

Supplement: S1 Dataset — All collected images were collected together, labeled and summarized one by one, and resulting classification results were roughly classified into three major categories: dry, wet and snowy. (ZIP) [file pone.0310858.s001.zip › weather1_data/snow_road/微信截图_20230906115150.png]

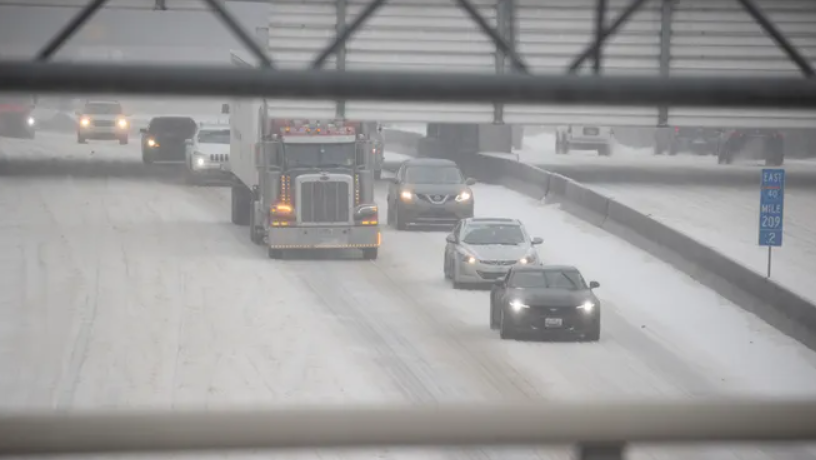

Supplement: S1 Dataset — All collected images were collected together, labeled and summarized one by one, and resulting classification results were roughly classified into three major categories: dry, wet and snowy. (ZIP) [file pone.0310858.s001.zip › weather1_data/snow_road/微信截图_20230906115158.png]

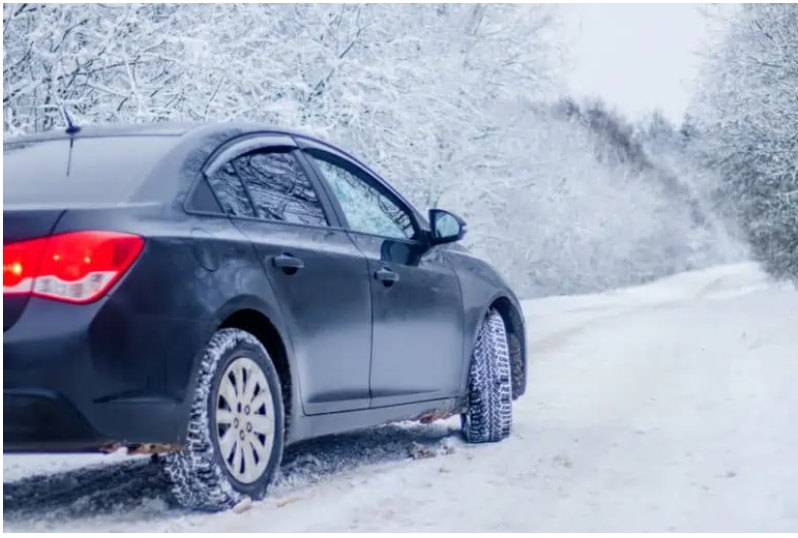

Supplement: S1 Dataset — All collected images were collected together, labeled and summarized one by one, and resulting classification results were roughly classified into three major categories: dry, wet and snowy. (ZIP) [file pone.0310858.s001.zip › weather1_data/snow_road/微信截图_20230906115909.png]

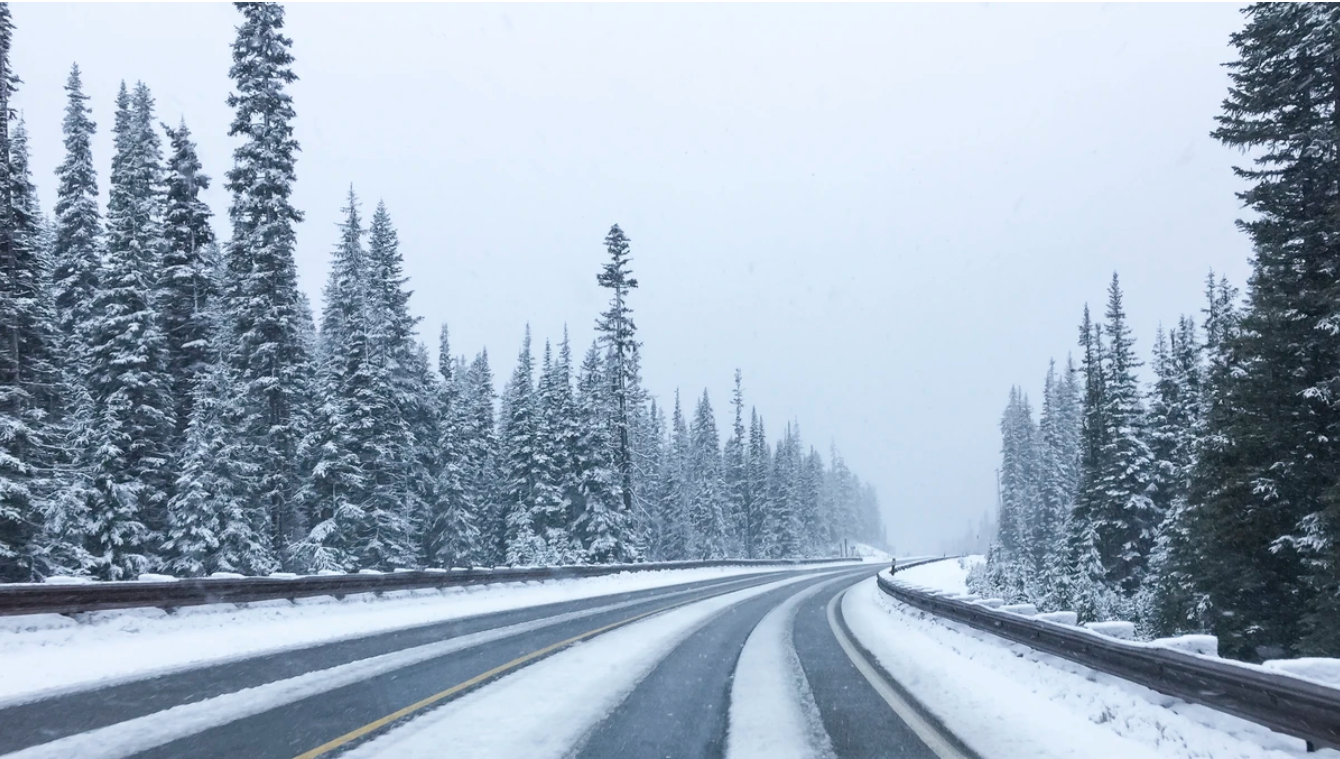

Supplement: S1 Dataset — All collected images were collected together, labeled and summarized one by one, and resulting classification results were roughly classified into three major categories: dry, wet and snowy. (ZIP) [file pone.0310858.s001.zip › weather1_data/snow_road/微信截图_20230906123857.png]

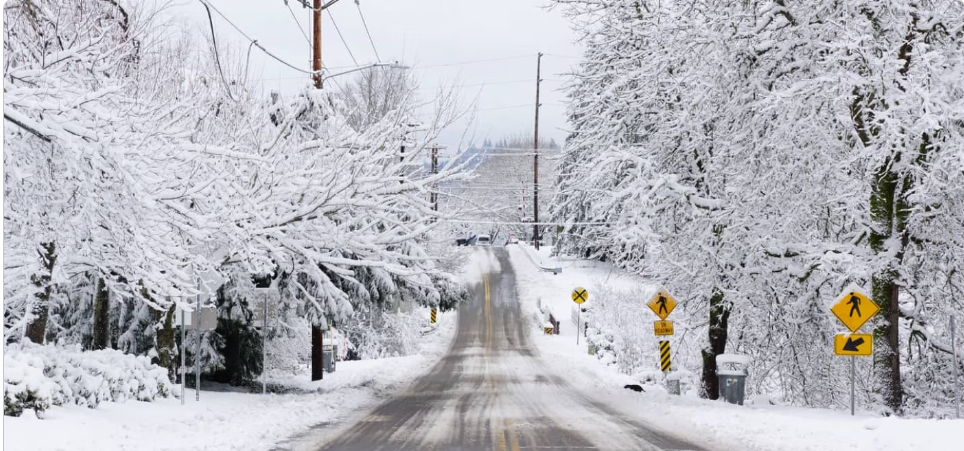

Supplement: S1 Dataset — All collected images were collected together, labeled and summarized one by one, and resulting classification results were roughly classified into three major categories: dry, wet and snowy. (ZIP) [file pone.0310858.s001.zip › weather1_data/snow_road/微信截图_20230906124144.png]

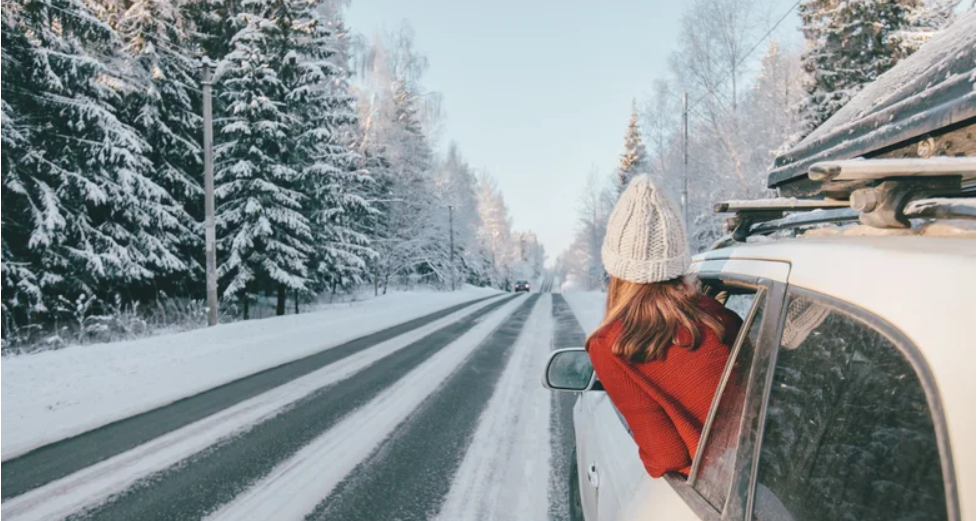

Supplement: S1 Dataset — All collected images were collected together, labeled and summarized one by one, and resulting classification results were roughly classified into three major categories: dry, wet and snowy. (ZIP) [file pone.0310858.s001.zip › weather1_data/snow_road/微信截图_20230906124610.png]

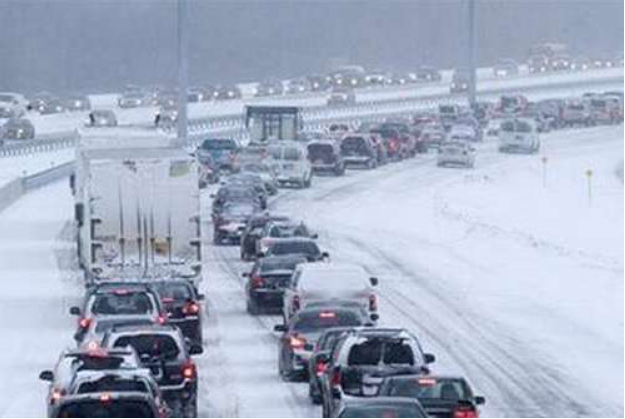

Supplement: S1 Dataset — All collected images were collected together, labeled and summarized one by one, and resulting classification results were roughly classified into three major categories: dry, wet and snowy. (ZIP) [file pone.0310858.s001.zip › weather1_data/snow_road/微信截图_20230906125416.png]

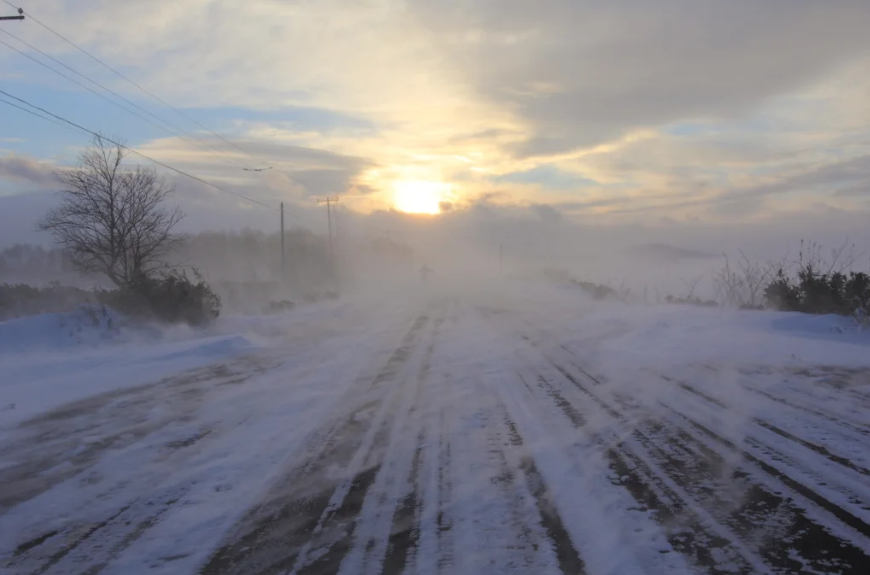

Supplement: S1 Dataset — All collected images were collected together, labeled and summarized one by one, and resulting classification results were roughly classified into three major categories: dry, wet and snowy. (ZIP) [file pone.0310858.s001.zip › weather1_data/snow_road/微信截图_20230906141722.png]

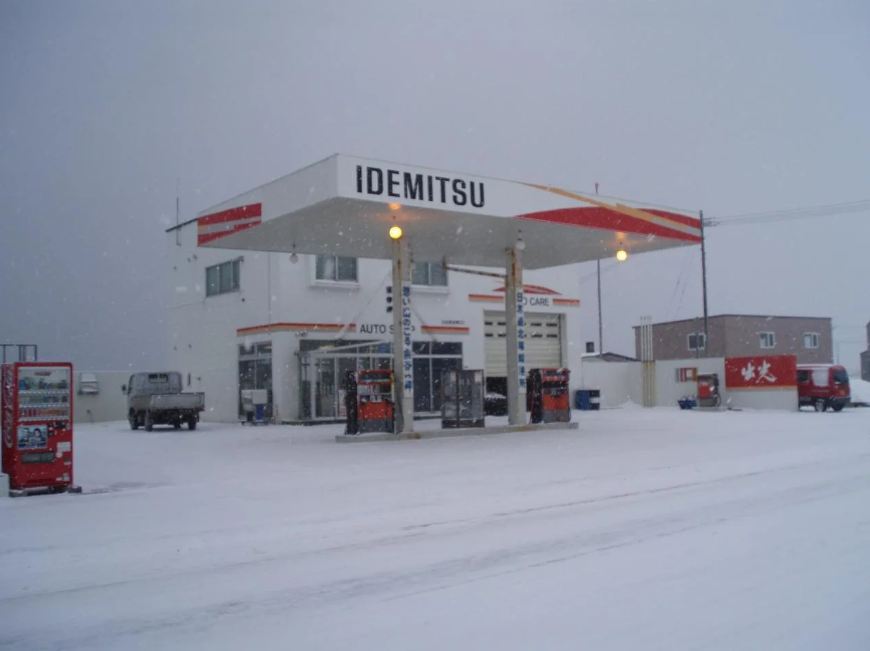

Supplement: S1 Dataset — All collected images were collected together, labeled and summarized one by one, and resulting classification results were roughly classified into three major categories: dry, wet and snowy. (ZIP) [file pone.0310858.s001.zip › weather1_data/snow_road/微信截图_20230906141730.png]

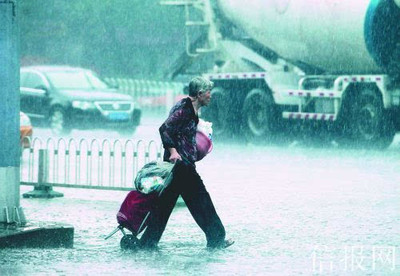

Supplement: S1 Dataset — All collected images were collected together, labeled and summarized one by one, and resulting classification results were roughly classified into three major categories: dry, wet and snowy. (ZIP) [file pone.0310858.s001.zip › weather1_data/wet_road/1048.jpg]

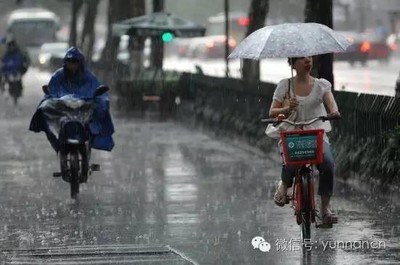

Supplement: S1 Dataset — All collected images were collected together, labeled and summarized one by one, and resulting classification results were roughly classified into three major categories: dry, wet and snowy. (ZIP) [file pone.0310858.s001.zip › weather1_data/wet_road/1053.jpg]

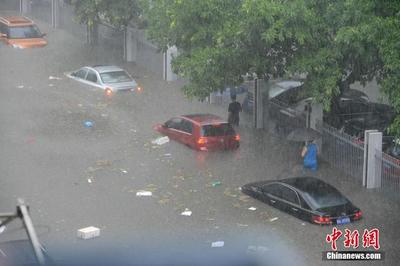

Supplement: S1 Dataset — All collected images were collected together, labeled and summarized one by one, and resulting classification results were roughly classified into three major categories: dry, wet and snowy. (ZIP) [file pone.0310858.s001.zip › weather1_data/wet_road/1056.jpg]

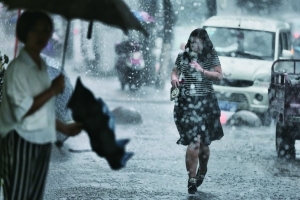

Supplement: S1 Dataset — All collected images were collected together, labeled and summarized one by one, and resulting classification results were roughly classified into three major categories: dry, wet and snowy. (ZIP) [file pone.0310858.s001.zip › weather1_data/wet_road/1140.jpg]

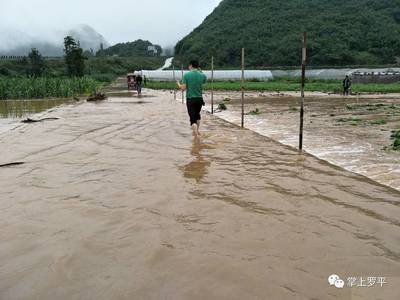

Supplement: S1 Dataset — All collected images were collected together, labeled and summarized one by one, and resulting classification results were roughly classified into three major categories: dry, wet and snowy. (ZIP) [file pone.0310858.s001.zip › weather1_data/wet_road/1157.jpg]

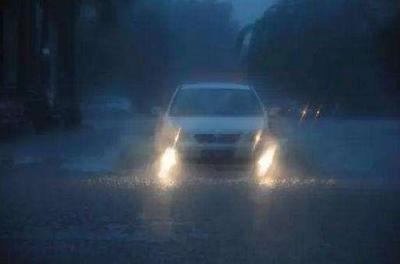

Supplement: S1 Dataset — All collected images were collected together, labeled and summarized one by one, and resulting classification results were roughly classified into three major categories: dry, wet and snowy. (ZIP) [file pone.0310858.s001.zip › weather1_data/wet_road/1161.jpg]

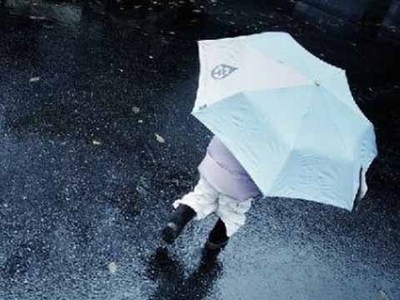

Supplement: S1 Dataset — All collected images were collected together, labeled and summarized one by one, and resulting classification results were roughly classified into three major categories: dry, wet and snowy. (ZIP) [file pone.0310858.s001.zip › weather1_data/wet_road/1168.jpg]

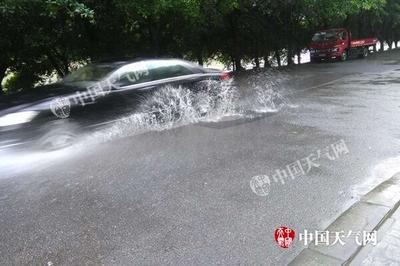

Supplement: S1 Dataset — All collected images were collected together, labeled and summarized one by one, and resulting classification results were roughly classified into three major categories: dry, wet and snowy. (ZIP) [file pone.0310858.s001.zip › weather1_data/wet_road/132.jpg]

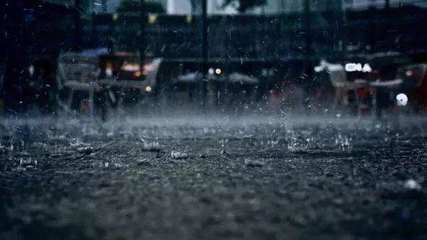

Supplement: S1 Dataset — All collected images were collected together, labeled and summarized one by one, and resulting classification results were roughly classified into three major categories: dry, wet and snowy. (ZIP) [file pone.0310858.s001.zip › weather1_data/wet_road/14.jpg]

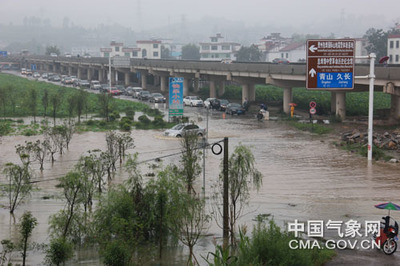

Supplement: S1 Dataset — All collected images were collected together, labeled and summarized one by one, and resulting classification results were roughly classified into three major categories: dry, wet and snowy. (ZIP) [file pone.0310858.s001.zip › weather1_data/wet_road/1566.jpg]

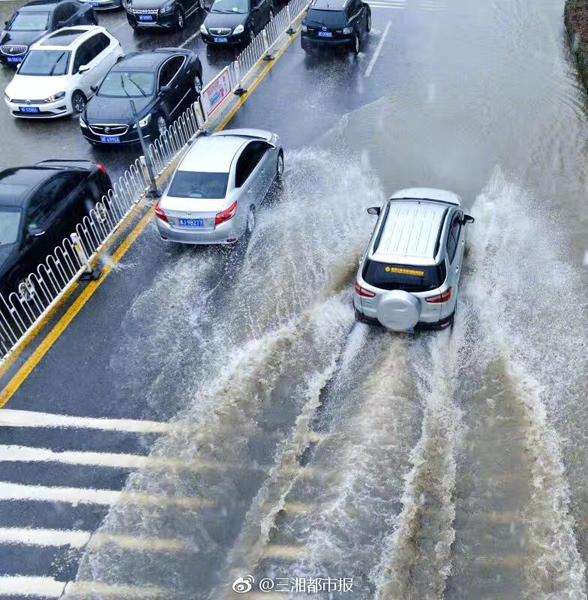

Supplement: S1 Dataset — All collected images were collected together, labeled and summarized one by one, and resulting classification results were roughly classified into three major categories: dry, wet and snowy. (ZIP) [file pone.0310858.s001.zip › weather1_data/wet_road/1568.jpg]

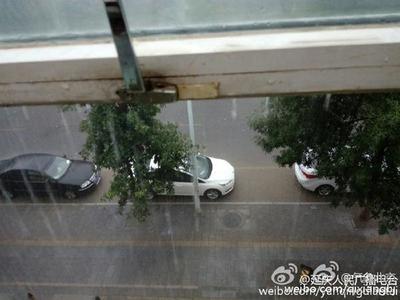

Supplement: S1 Dataset — All collected images were collected together, labeled and summarized one by one, and resulting classification results were roughly classified into three major categories: dry, wet and snowy. (ZIP) [file pone.0310858.s001.zip › weather1_data/wet_road/1581.jpg]

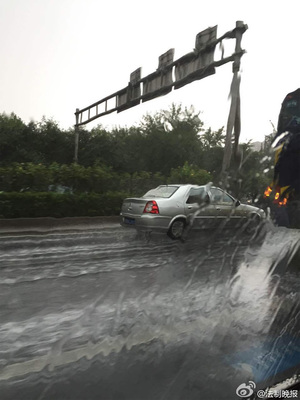

Supplement: S1 Dataset — All collected images were collected together, labeled and summarized one by one, and resulting classification results were roughly classified into three major categories: dry, wet and snowy. (ZIP) [file pone.0310858.s001.zip › weather1_data/wet_road/1585.jpg]

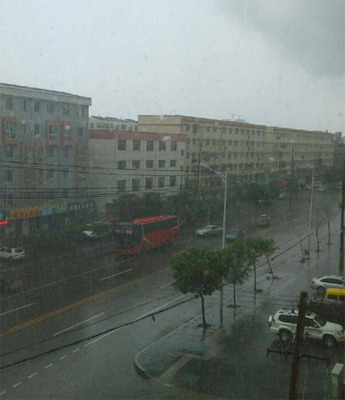

Supplement: S1 Dataset — All collected images were collected together, labeled and summarized one by one, and resulting classification results were roughly classified into three major categories: dry, wet and snowy. (ZIP) [file pone.0310858.s001.zip › weather1_data/wet_road/1587.jpg]

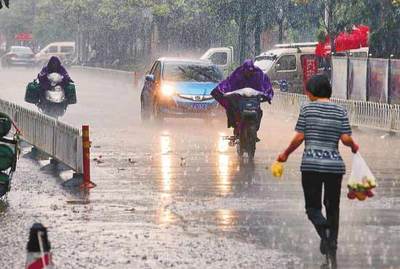

Supplement: S1 Dataset — All collected images were collected together, labeled and summarized one by one, and resulting classification results were roughly classified into three major categories: dry, wet and snowy. (ZIP) [file pone.0310858.s001.zip › weather1_data/wet_road/1591.jpg]

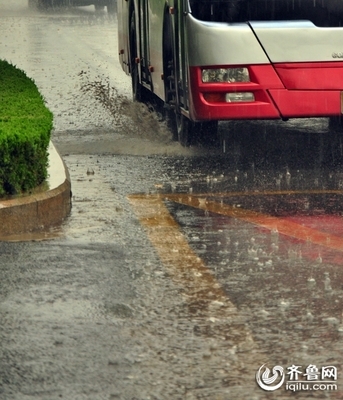

Supplement: S1 Dataset — All collected images were collected together, labeled and summarized one by one, and resulting classification results were roughly classified into three major categories: dry, wet and snowy. (ZIP) [file pone.0310858.s001.zip › weather1_data/wet_road/1801.jpg]

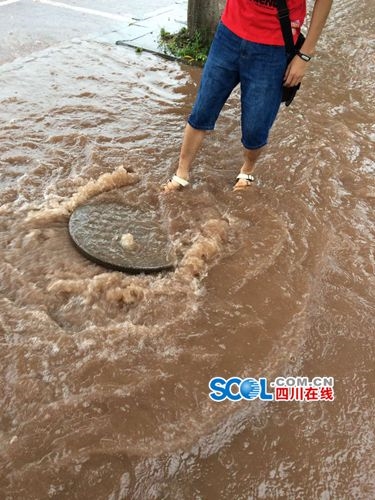

Supplement: S1 Dataset — All collected images were collected together, labeled and summarized one by one, and resulting classification results were roughly classified into three major categories: dry, wet and snowy. (ZIP) [file pone.0310858.s001.zip › weather1_data/wet_road/1815.jpg]

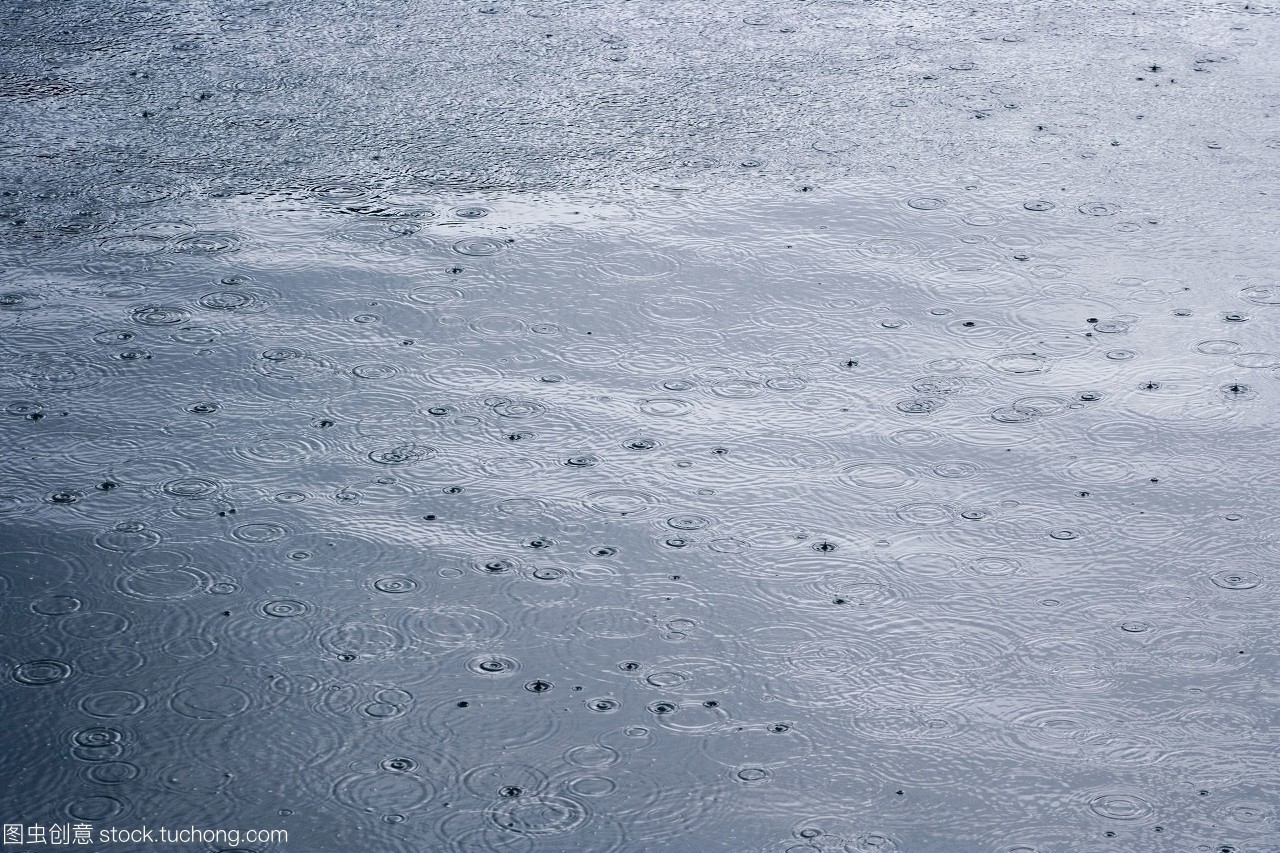

Supplement: S1 Dataset — All collected images were collected together, labeled and summarized one by one, and resulting classification results were roughly classified into three major categories: dry, wet and snowy. (ZIP) [file pone.0310858.s001.zip › weather1_data/wet_road/1821.jpg]

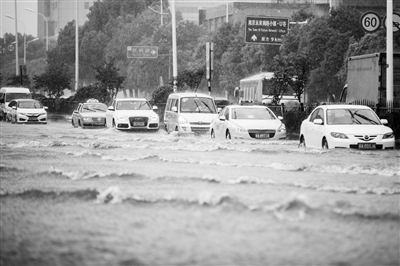

Supplement: S1 Dataset — All collected images were collected together, labeled and summarized one by one, and resulting classification results were roughly classified into three major categories: dry, wet and snowy. (ZIP) [file pone.0310858.s001.zip › weather1_data/wet_road/1823.jpg]

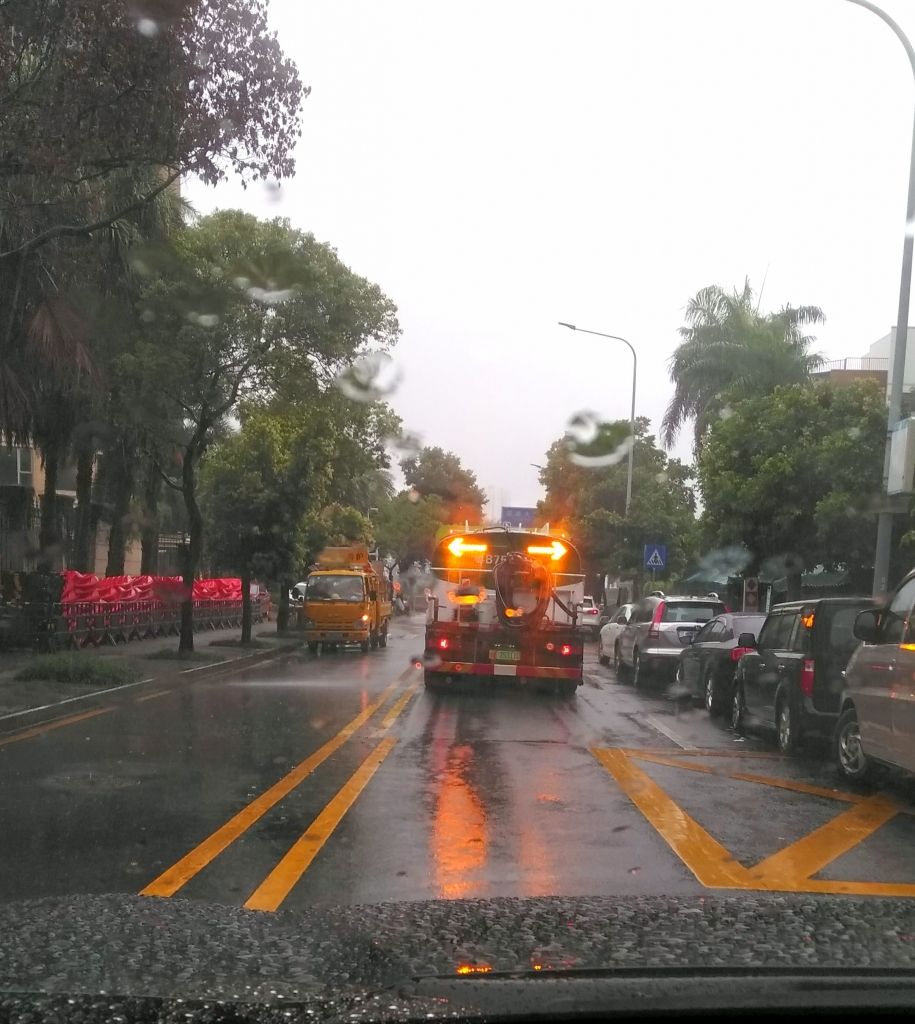

Supplement: S1 Dataset — All collected images were collected together, labeled and summarized one by one, and resulting classification results were roughly classified into three major categories: dry, wet and snowy. (ZIP) [file pone.0310858.s001.zip › weather1_data/wet_road/6a3af880a4e64e29bdb7ca920aea21fa.jpg]

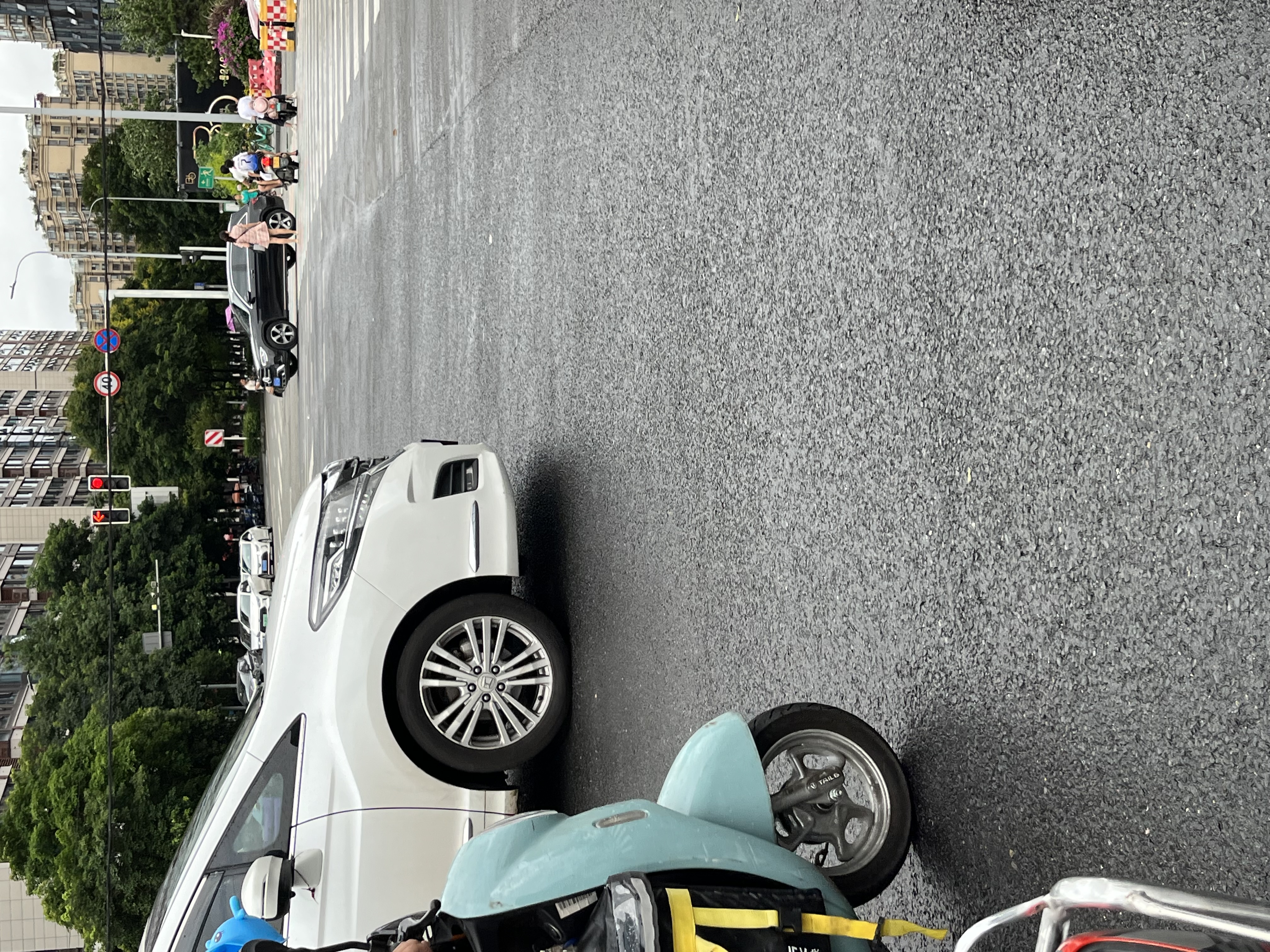

Supplement: S1 Dataset — All collected images were collected together, labeled and summarized one by one, and resulting classification results were roughly classified into three major categories: dry, wet and snowy. (ZIP) [file pone.0310858.s001.zip › weather1_data/wet_road/IMG_2240.jpg]

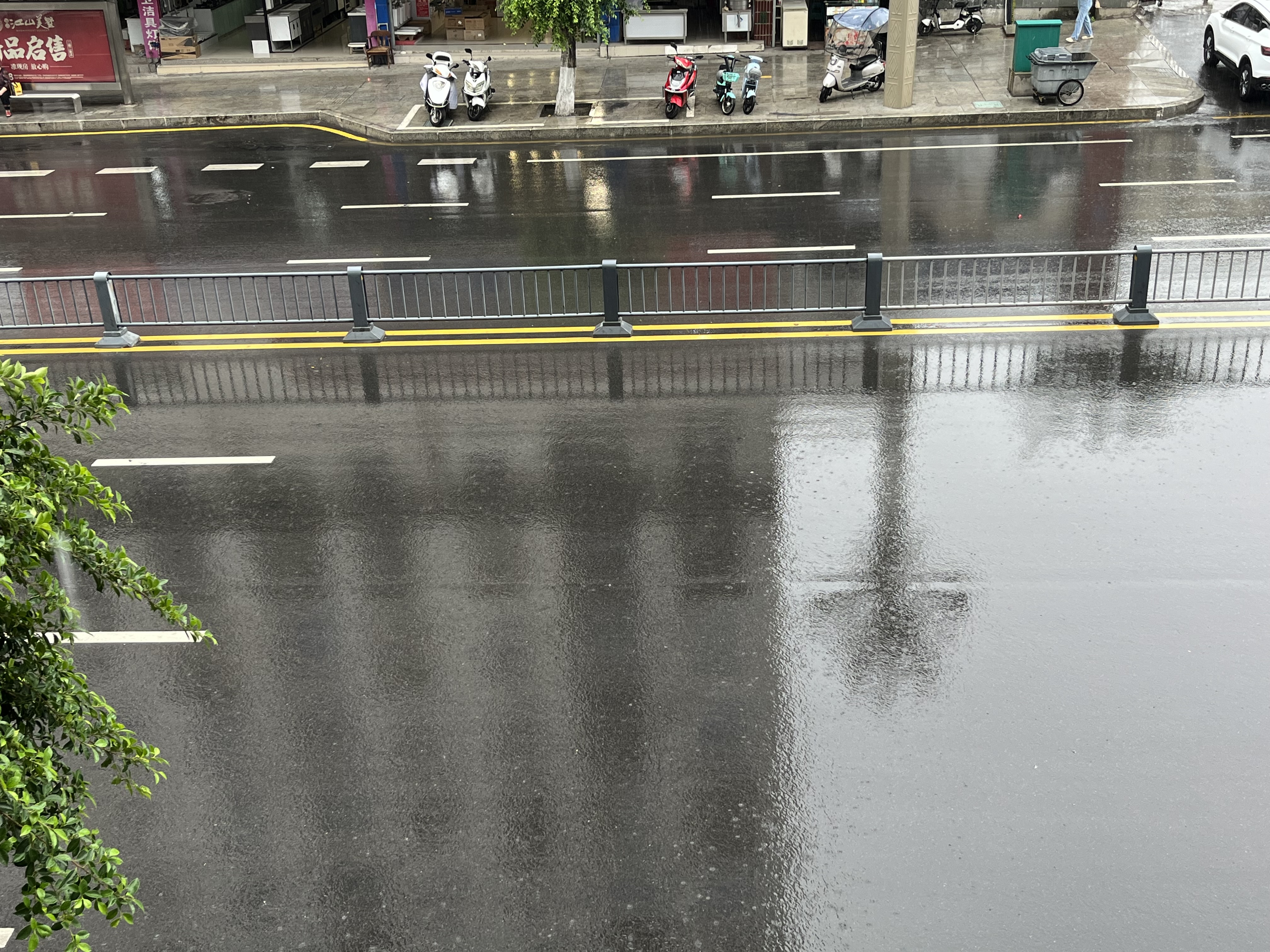

Supplement: S1 Dataset — All collected images were collected together, labeled and summarized one by one, and resulting classification results were roughly classified into three major categories: dry, wet and snowy. (ZIP) [file pone.0310858.s001.zip › weather1_data/wet_road/IMG_2312.jpg]

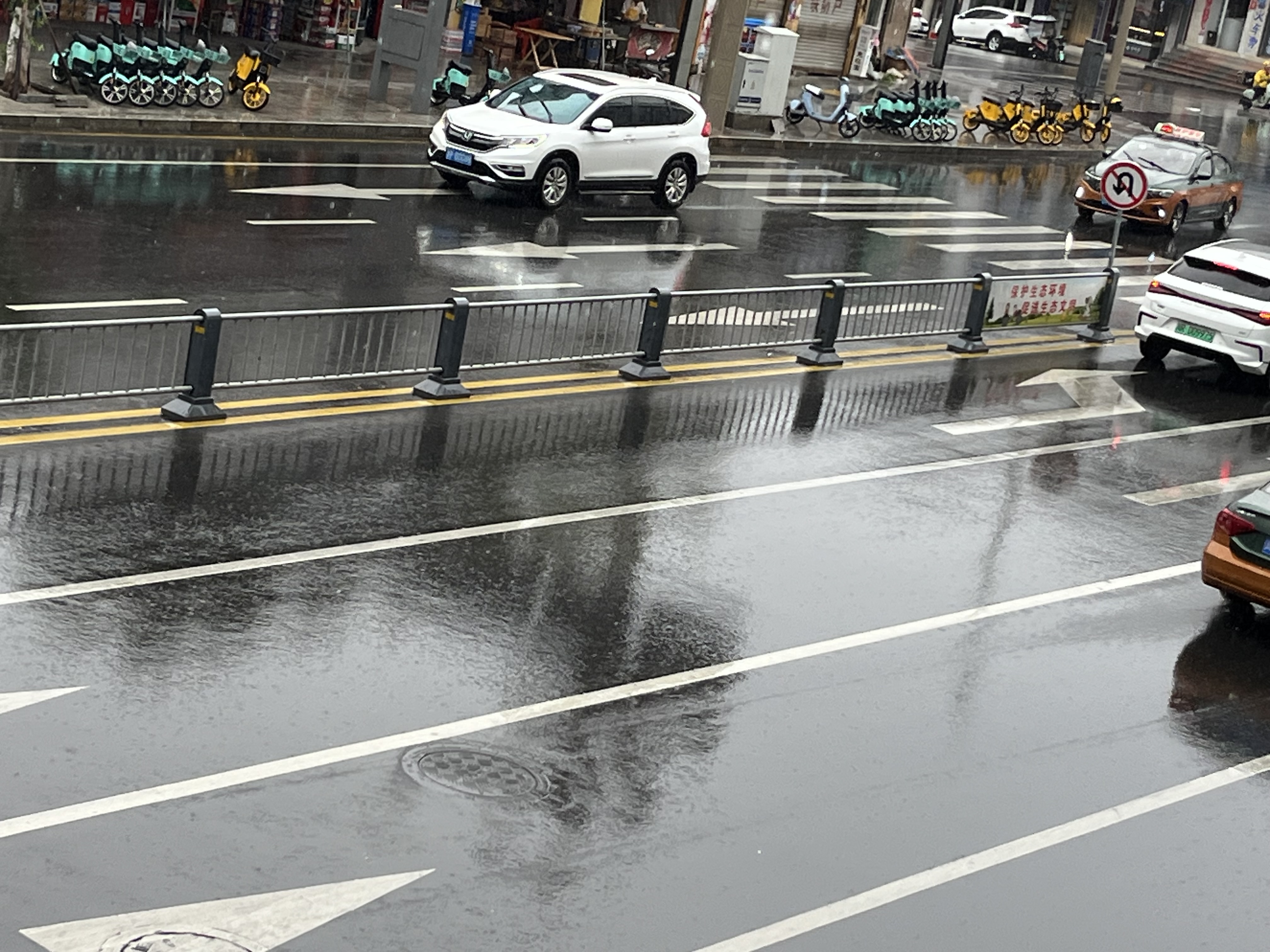

Supplement: S1 Dataset — All collected images were collected together, labeled and summarized one by one, and resulting classification results were roughly classified into three major categories: dry, wet and snowy. (ZIP) [file pone.0310858.s001.zip › weather1_data/wet_road/IMG_2313.jpg]

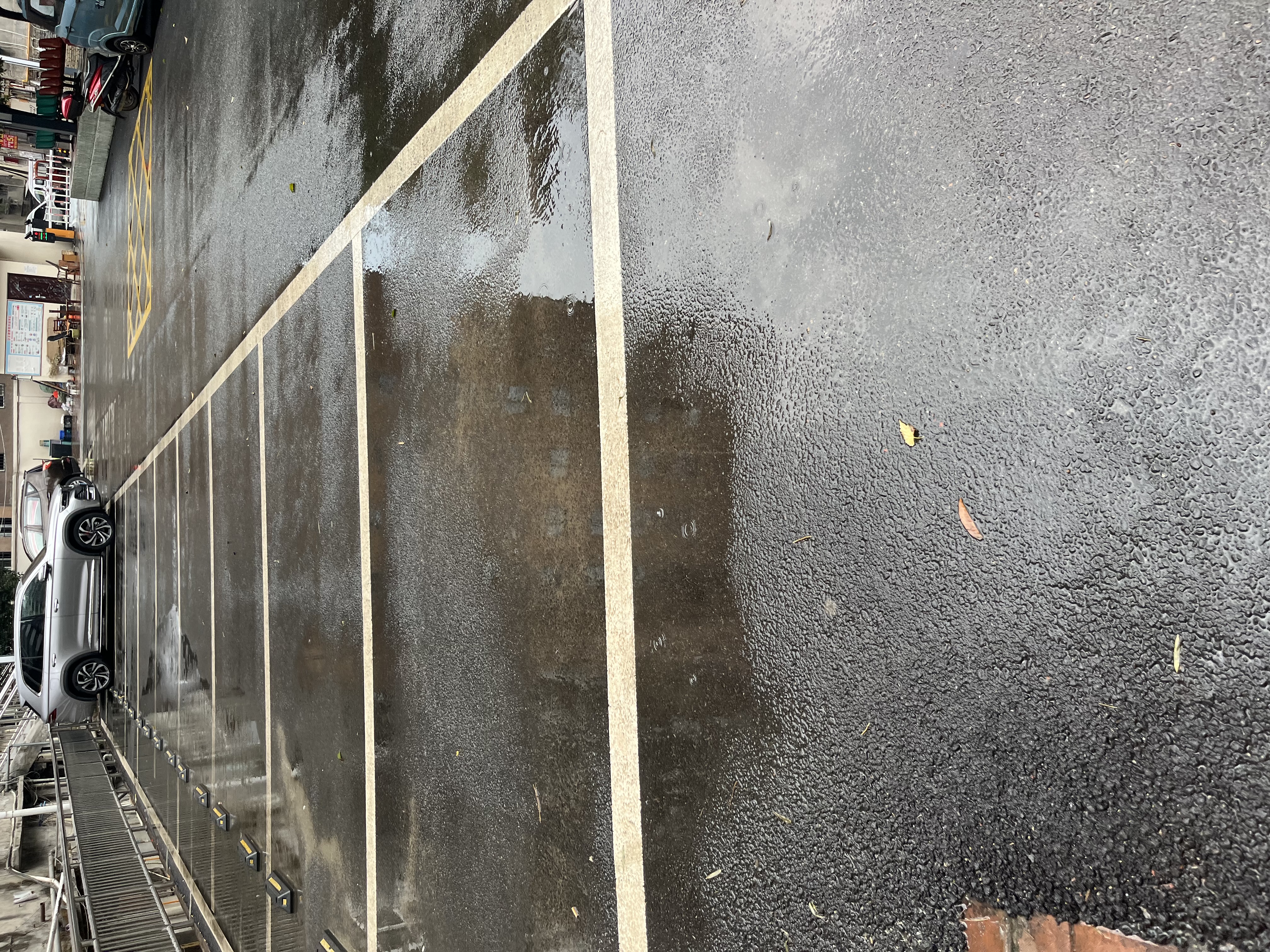

Supplement: S1 Dataset — All collected images were collected together, labeled and summarized one by one, and resulting classification results were roughly classified into three major categories: dry, wet and snowy. (ZIP) [file pone.0310858.s001.zip › weather1_data/wet_road/IMG_2319.jpg]

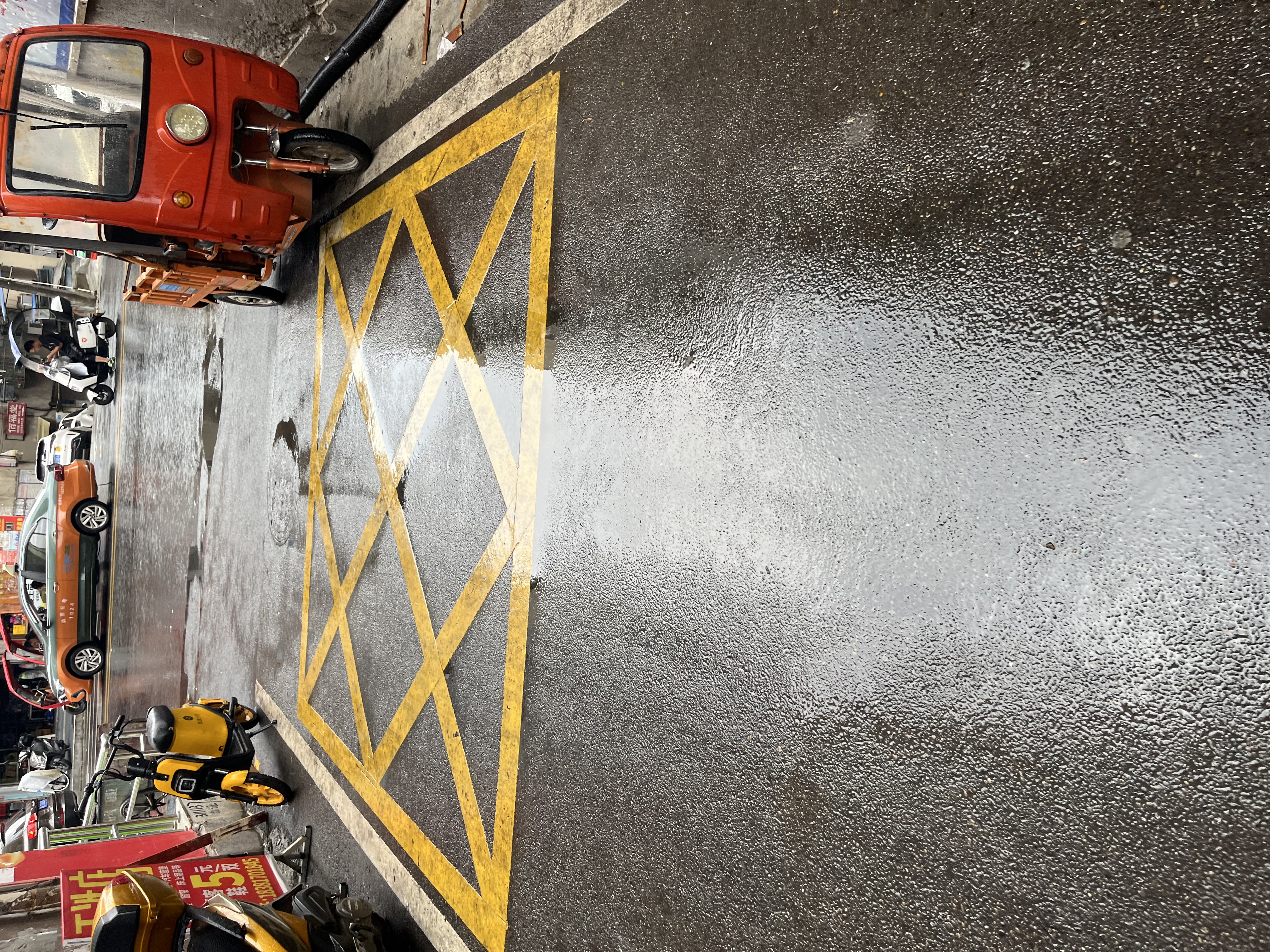

Supplement: S1 Dataset — All collected images were collected together, labeled and summarized one by one, and resulting classification results were roughly classified into three major categories: dry, wet and snowy. (ZIP) [file pone.0310858.s001.zip › weather1_data/wet_road/IMG_2325.jpg]

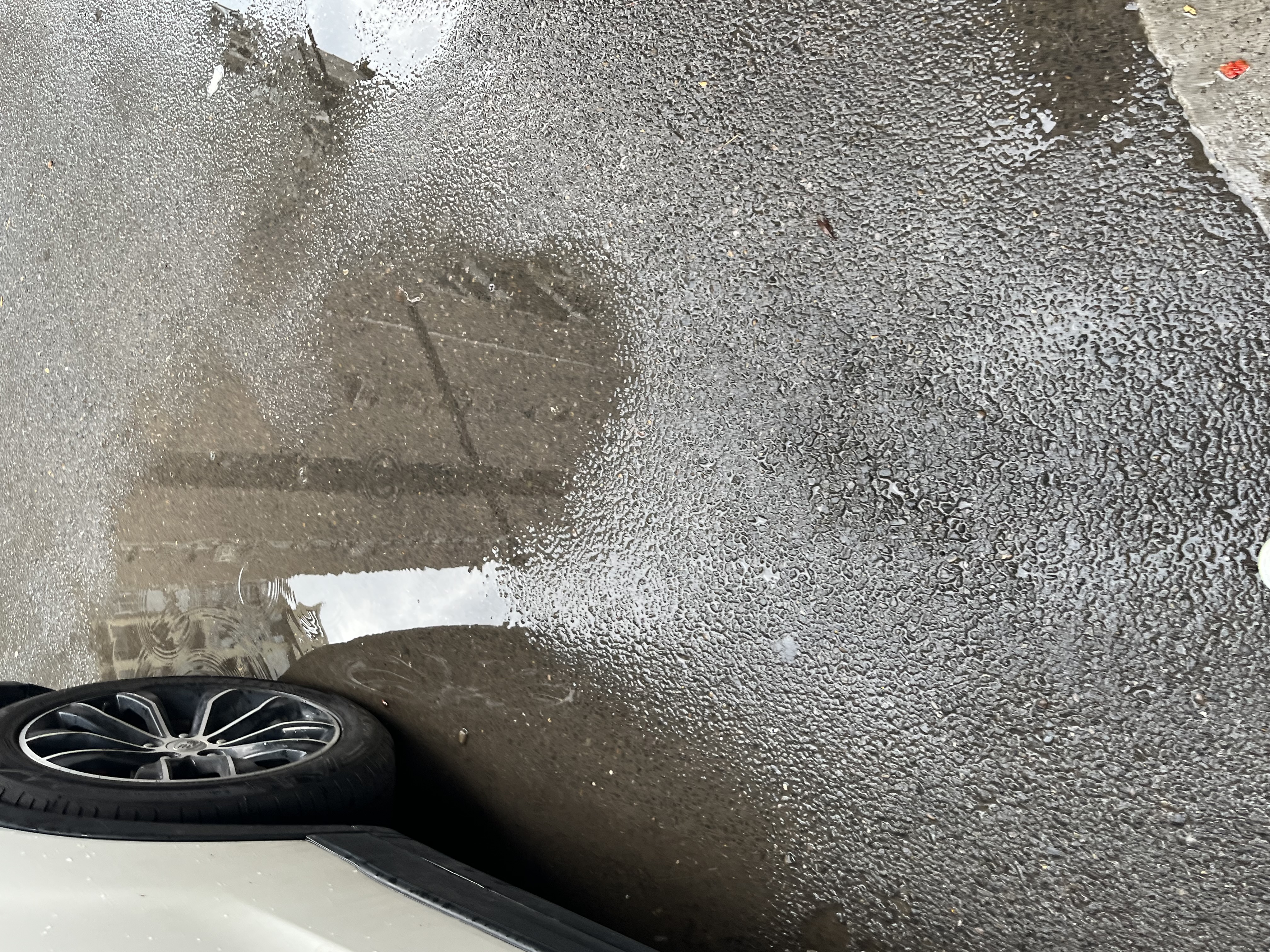

Supplement: S1 Dataset — All collected images were collected together, labeled and summarized one by one, and resulting classification results were roughly classified into three major categories: dry, wet and snowy. (ZIP) [file pone.0310858.s001.zip › weather1_data/wet_road/IMG_2331.jpg]

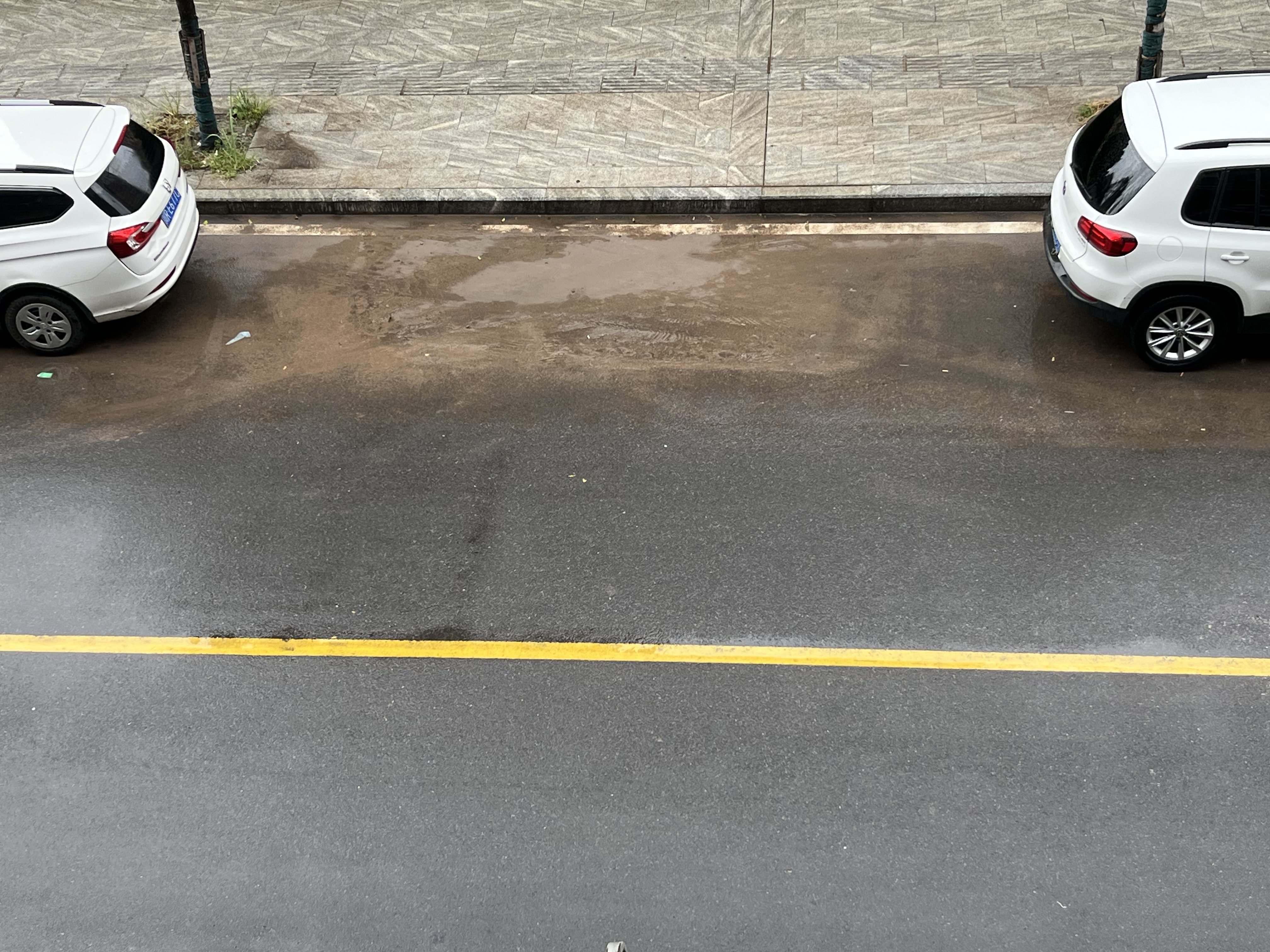

Supplement: S1 Dataset — All collected images were collected together, labeled and summarized one by one, and resulting classification results were roughly classified into three major categories: dry, wet and snowy. (ZIP) [file pone.0310858.s001.zip › weather1_data/wet_road/IMG_2334.jpg]

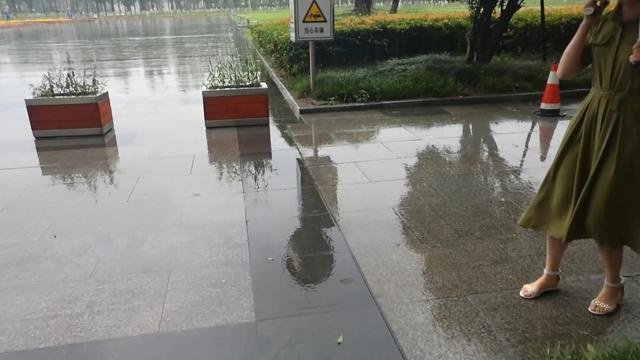

Supplement: S1 Dataset — All collected images were collected together, labeled and summarized one by one, and resulting classification results were roughly classified into three major categories: dry, wet and snowy. (ZIP) [file pone.0310858.s001.zip › weather1_data/wet_road/RainSight0.jpg]
