# Supplementary figures and images for: Research on recognition of slippery road surface and collision warning system based on deep learning (part 1 of 2)
Source: PLoS One. 2024 Nov 11;19(11):e0310858. doi: 10.1371/journal.pone.0310858 (PMC11554202; doi:10.1371/journal.pone.0310858)

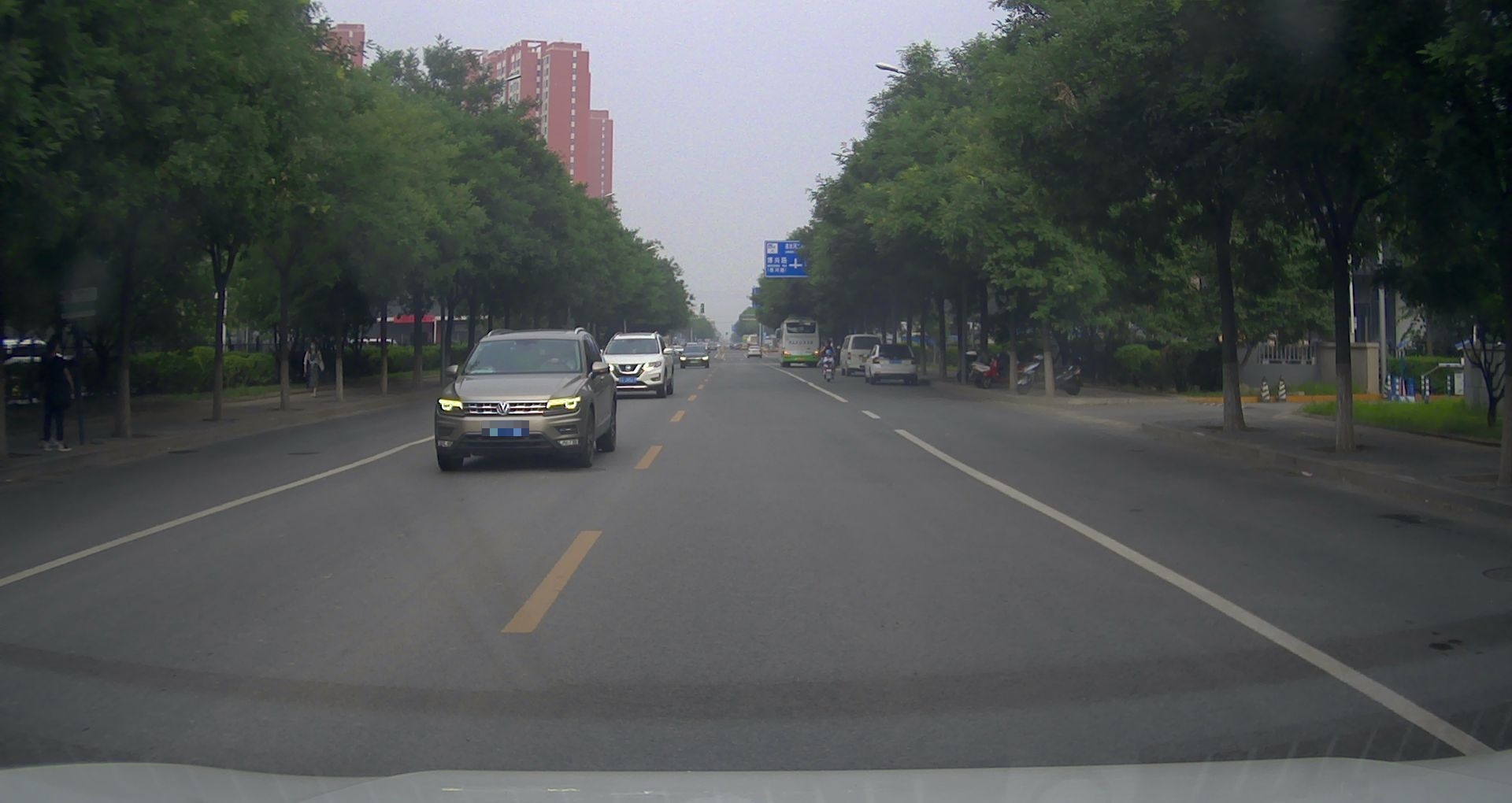

Supplement: S1 Dataset — All collected images were collected together, labeled and summarized one by one, and resulting classification results were roughly classified into three major categories: dry, wet and snowy. (ZIP) [file pone.0310858.s001.zip › weather1_data/dry_road/1627260258054.jpg]

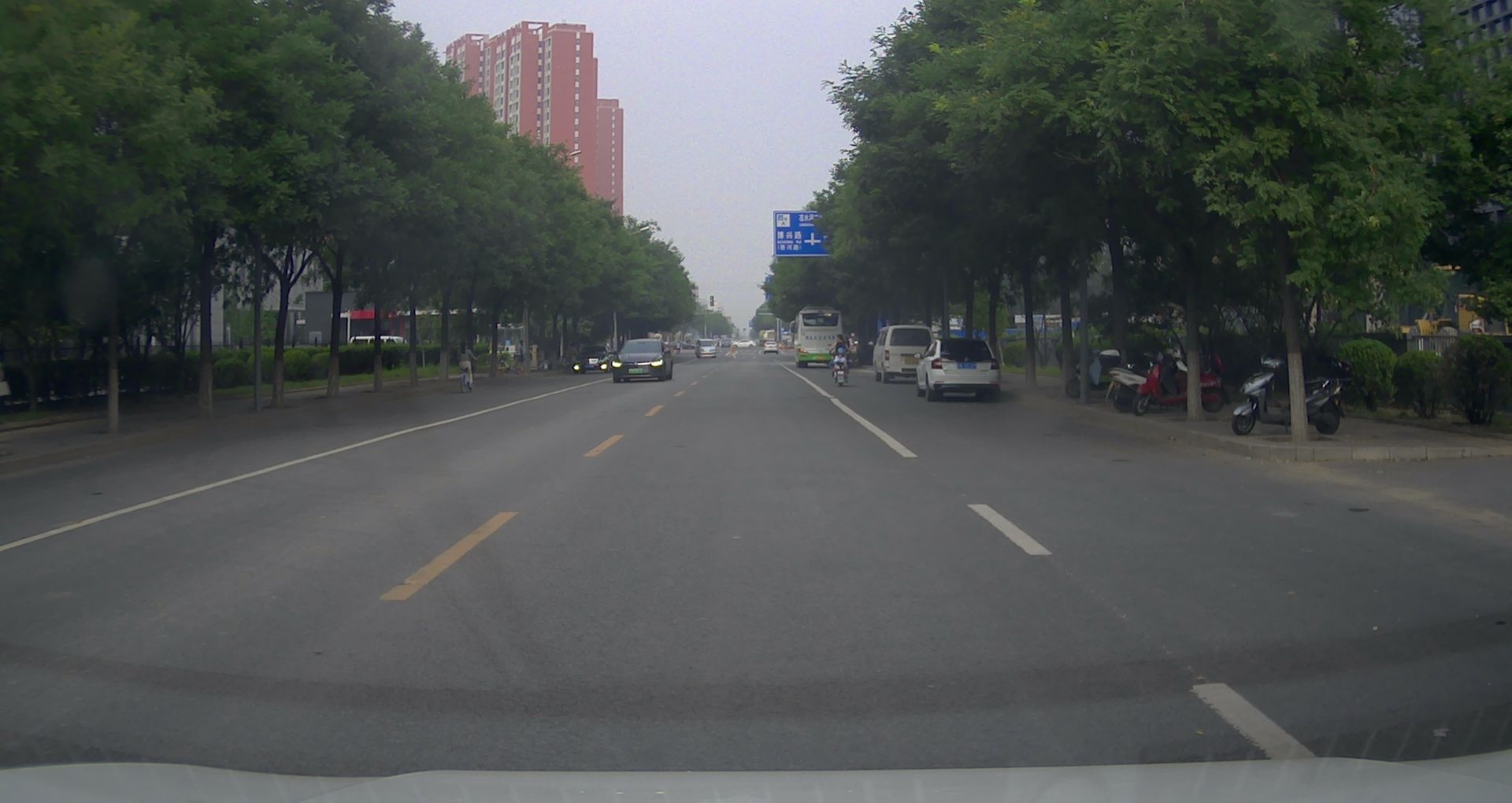

Supplement: S1 Dataset — All collected images were collected together, labeled and summarized one by one, and resulting classification results were roughly classified into three major categories: dry, wet and snowy. (ZIP) [file pone.0310858.s001.zip › weather1_data/dry_road/1627260259796.jpg]

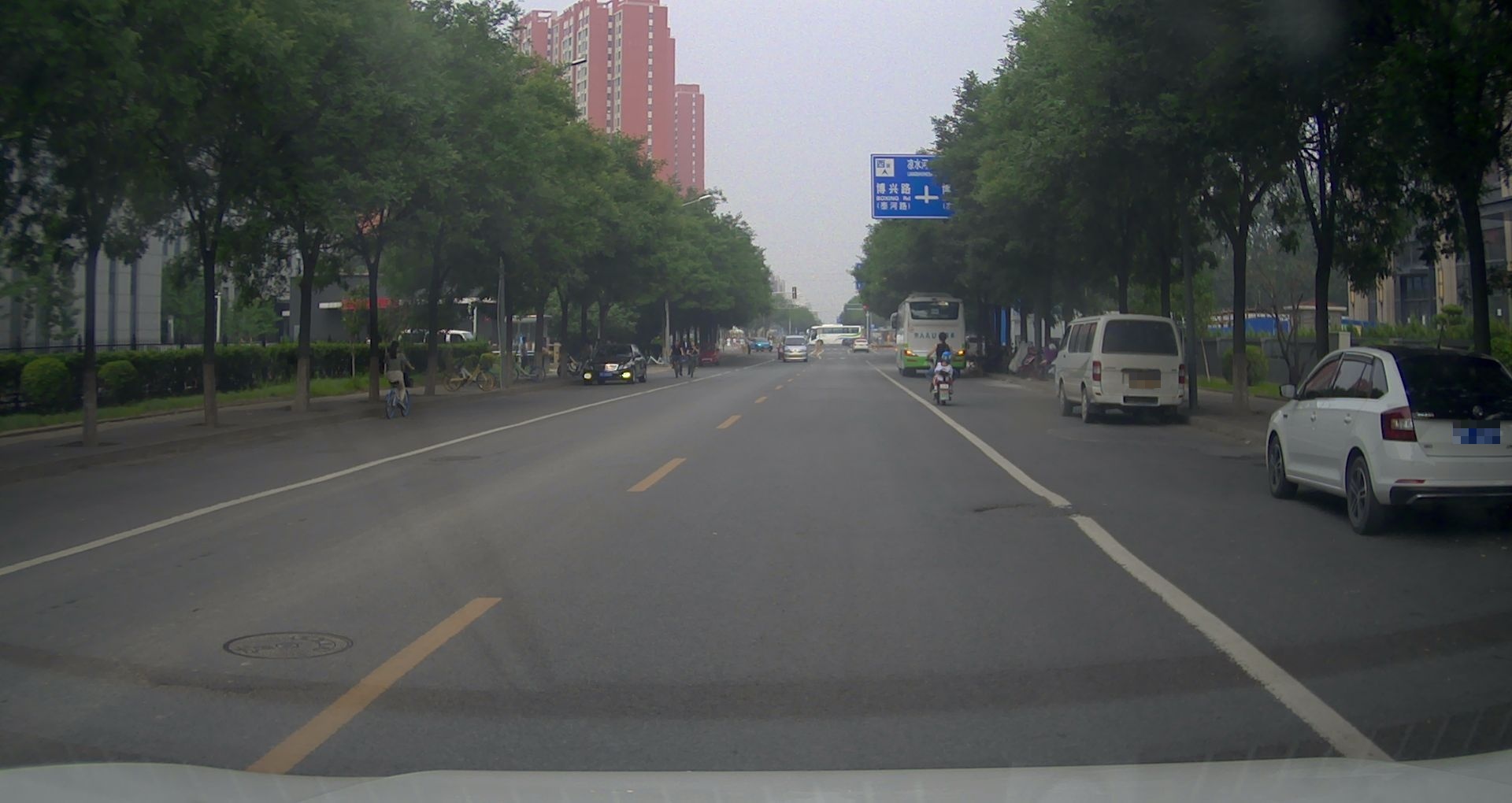

Supplement: S1 Dataset — All collected images were collected together, labeled and summarized one by one, and resulting classification results were roughly classified into three major categories: dry, wet and snowy. (ZIP) [file pone.0310858.s001.zip › weather1_data/dry_road/1627260261960.jpg]

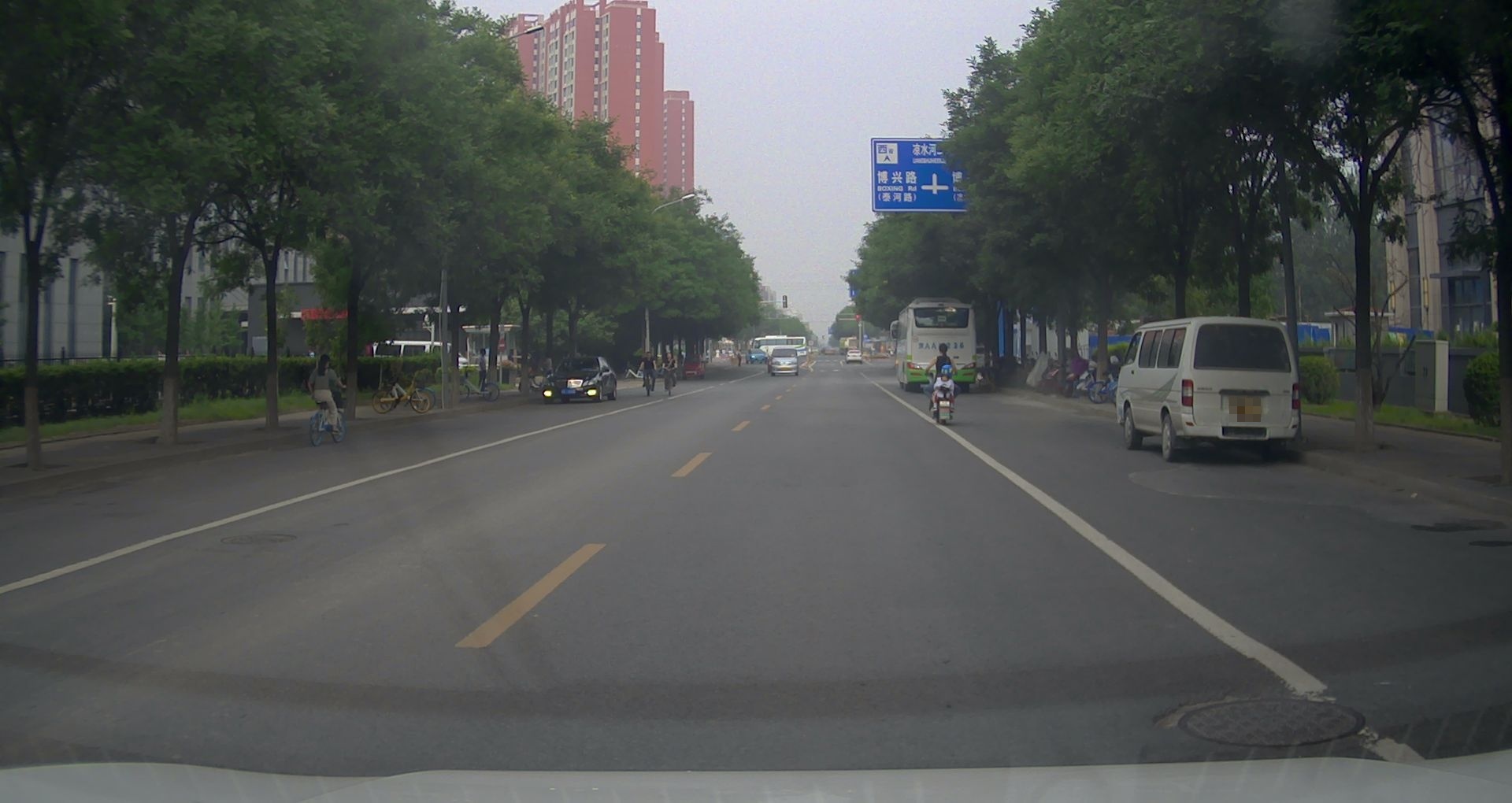

Supplement: S1 Dataset — All collected images were collected together, labeled and summarized one by one, and resulting classification results were roughly classified into three major categories: dry, wet and snowy. (ZIP) [file pone.0310858.s001.zip › weather1_data/dry_road/1627260262603.jpg]

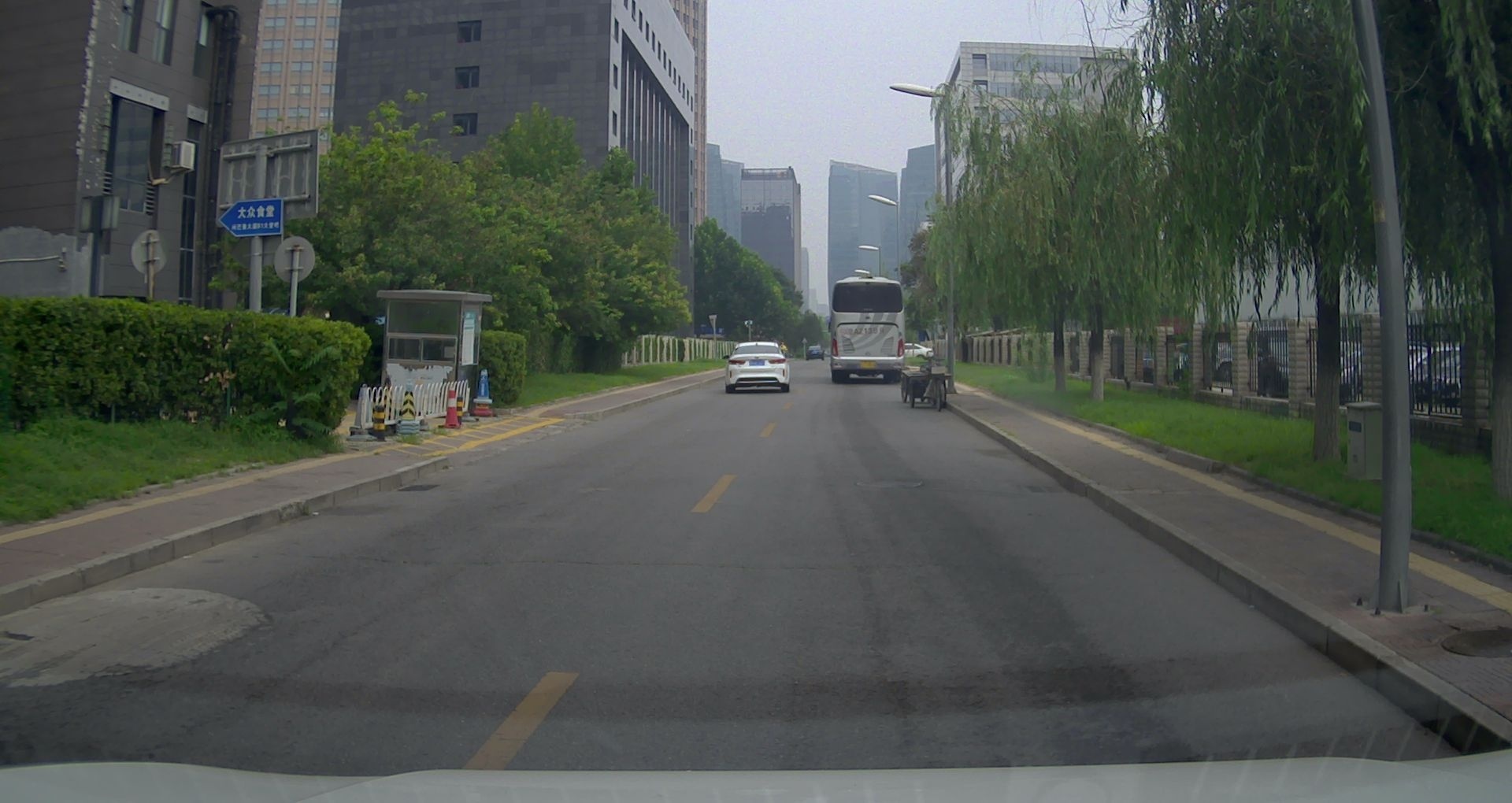

Supplement: S1 Dataset — All collected images were collected together, labeled and summarized one by one, and resulting classification results were roughly classified into three major categories: dry, wet and snowy. (ZIP) [file pone.0310858.s001.zip › weather1_data/dry_road/1627264638256.jpg]

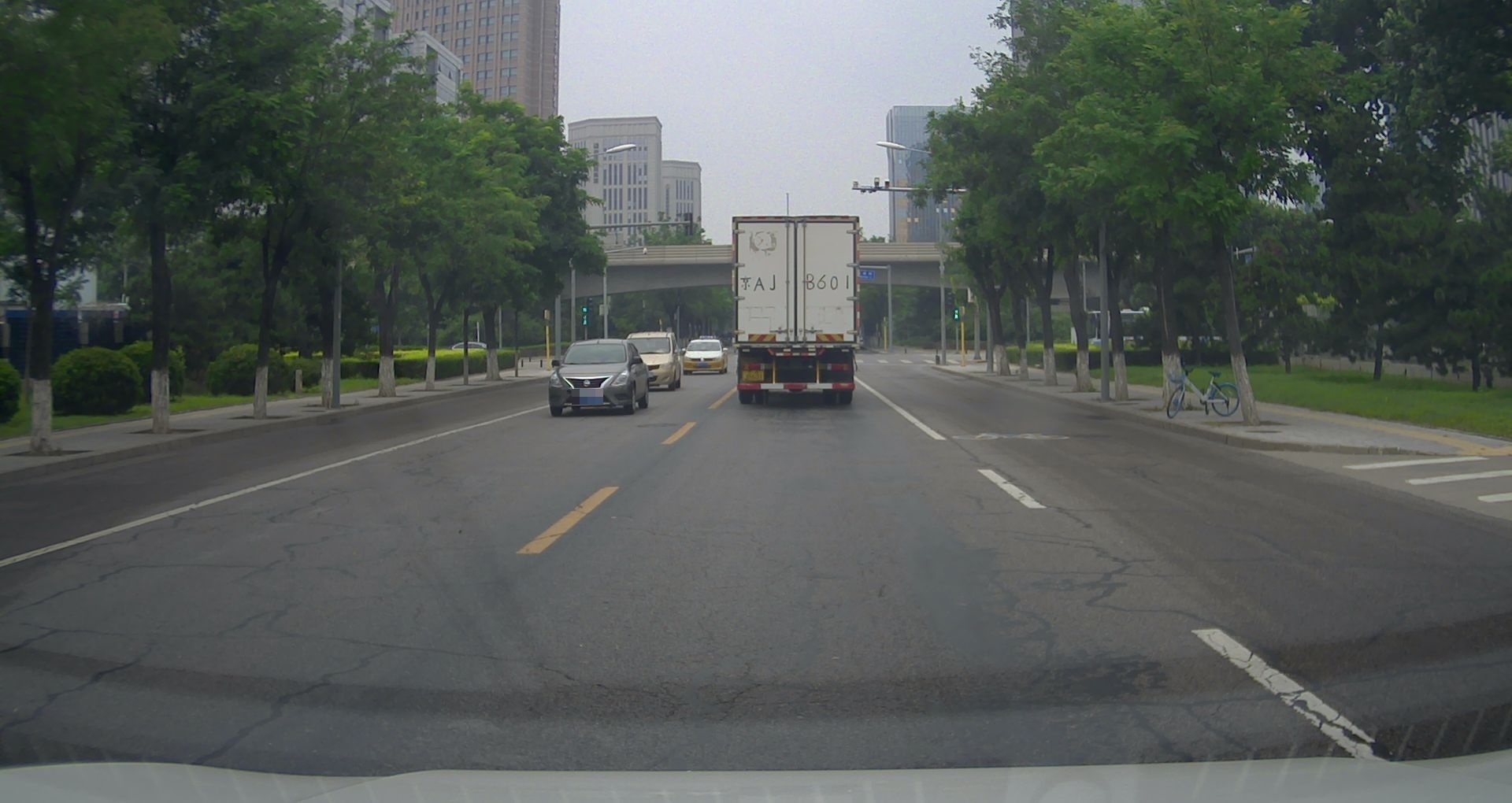

Supplement: S1 Dataset — All collected images were collected together, labeled and summarized one by one, and resulting classification results were roughly classified into three major categories: dry, wet and snowy. (ZIP) [file pone.0310858.s001.zip › weather1_data/dry_road/1627264980259.jpg]

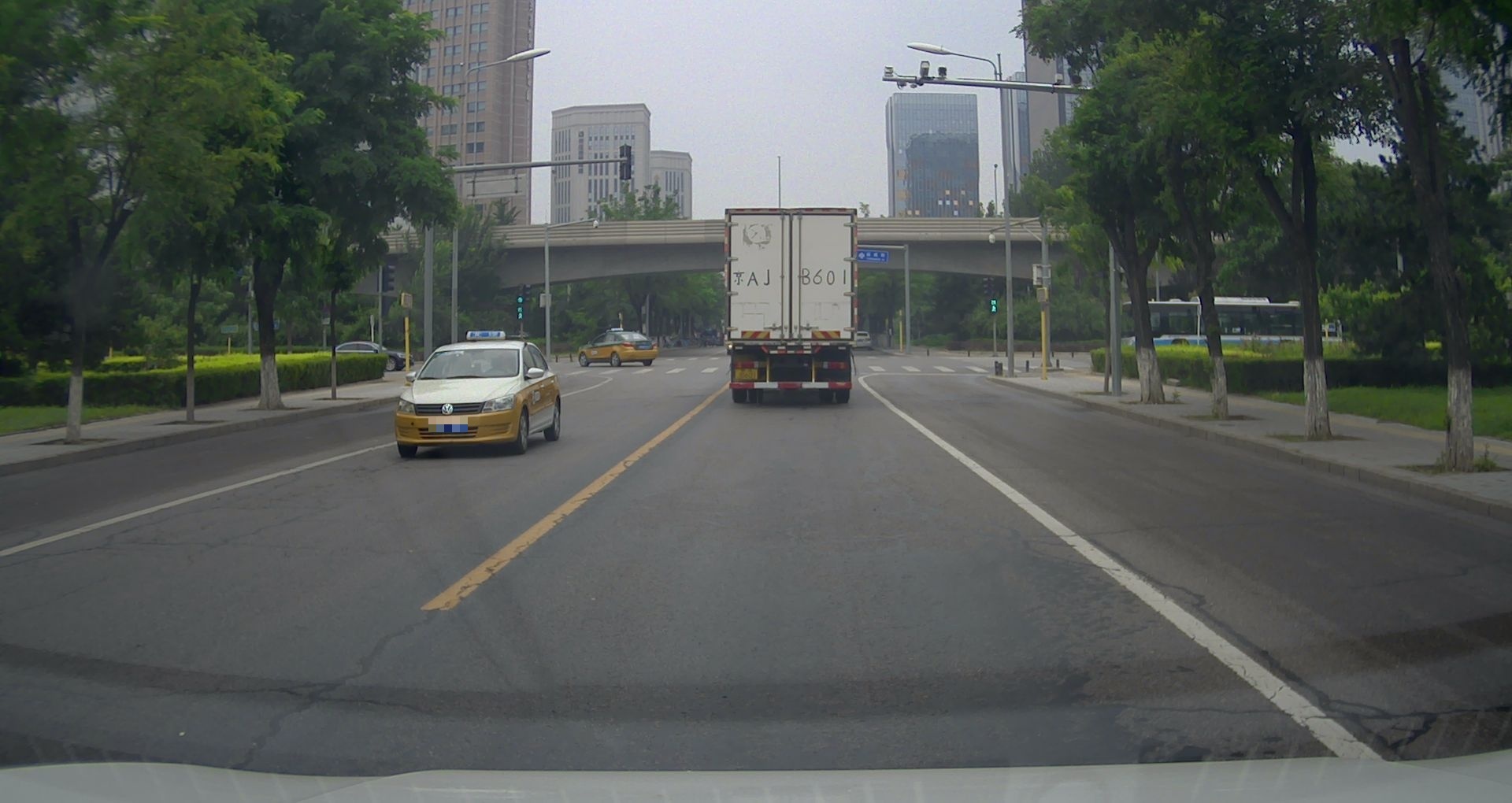

Supplement: S1 Dataset — All collected images were collected together, labeled and summarized one by one, and resulting classification results were roughly classified into three major categories: dry, wet and snowy. (ZIP) [file pone.0310858.s001.zip › weather1_data/dry_road/1627264982403.jpg]

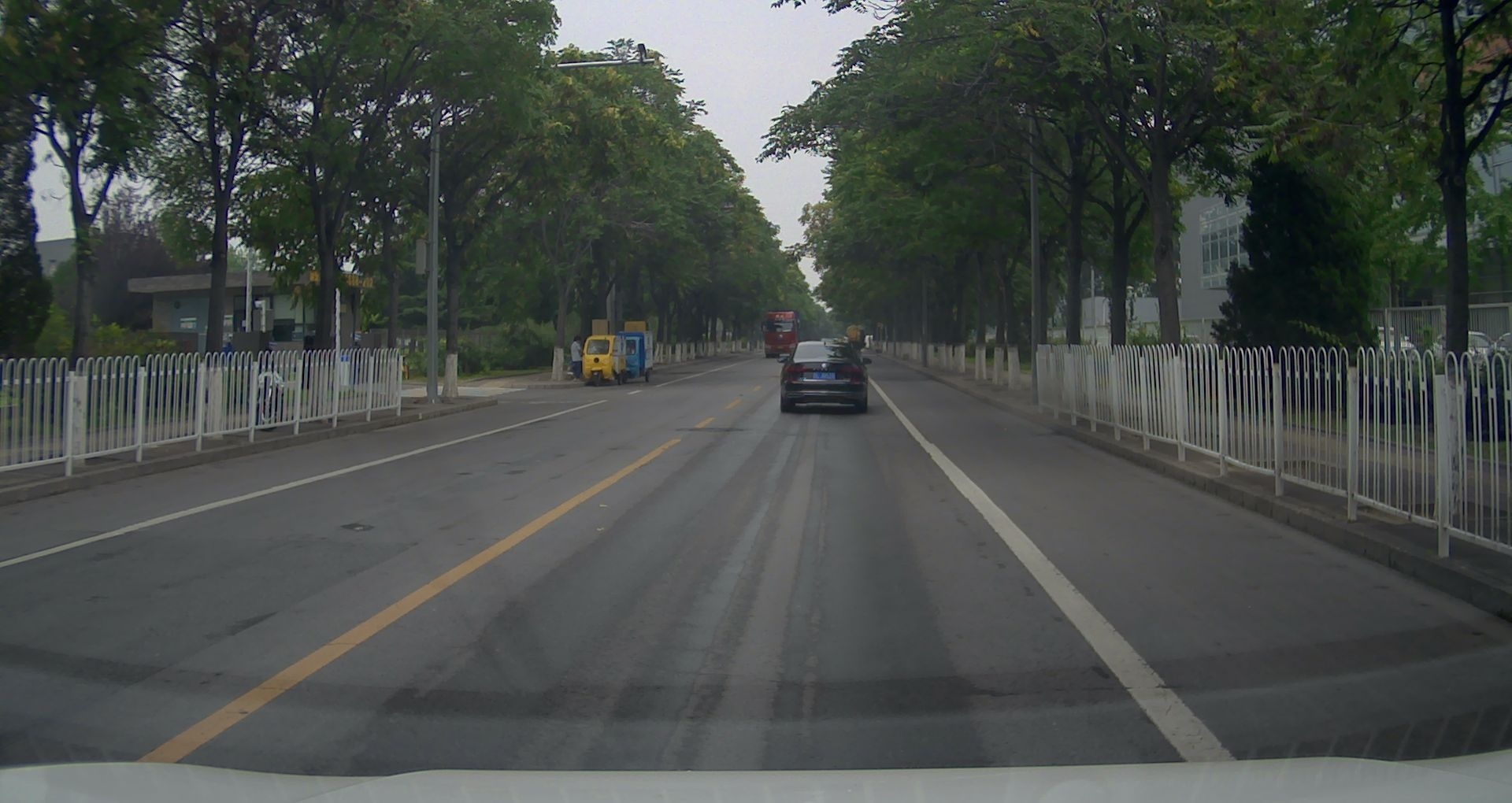

Supplement: S1 Dataset — All collected images were collected together, labeled and summarized one by one, and resulting classification results were roughly classified into three major categories: dry, wet and snowy. (ZIP) [file pone.0310858.s001.zip › weather1_data/dry_road/1627265529309.jpg]

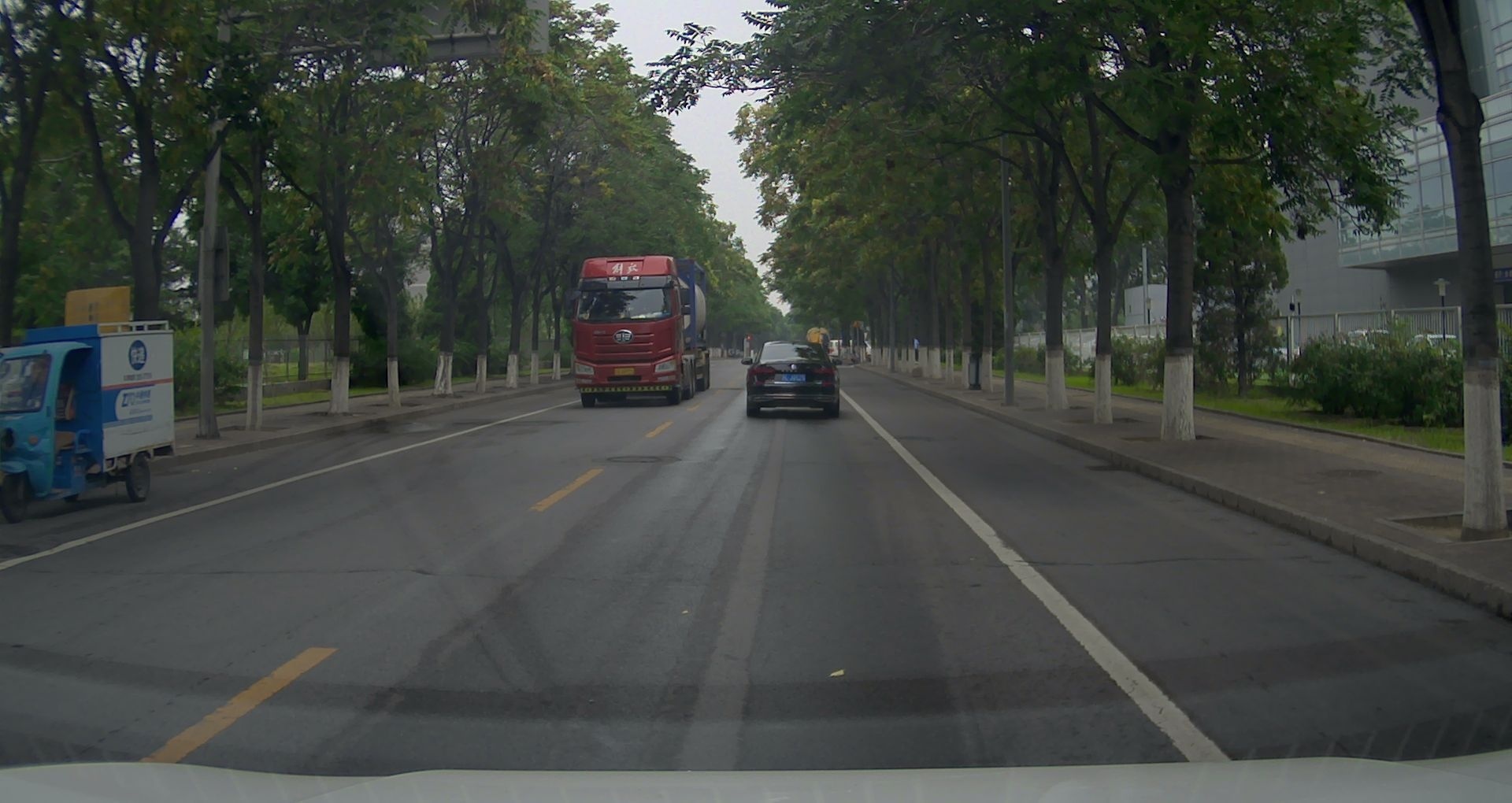

Supplement: S1 Dataset — All collected images were collected together, labeled and summarized one by one, and resulting classification results were roughly classified into three major categories: dry, wet and snowy. (ZIP) [file pone.0310858.s001.zip › weather1_data/dry_road/1627265533437.jpg]

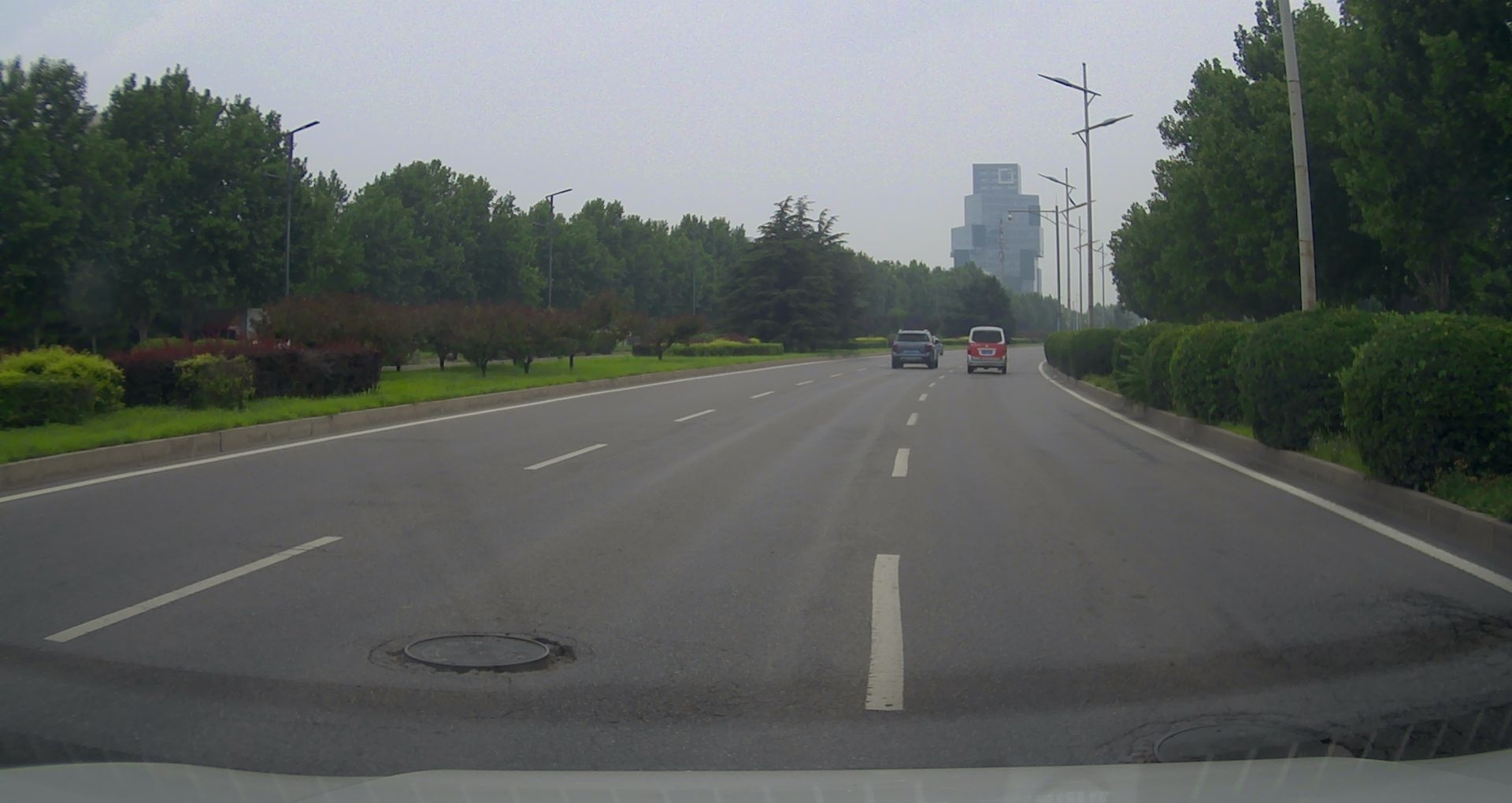

Supplement: S1 Dataset — All collected images were collected together, labeled and summarized one by one, and resulting classification results were roughly classified into three major categories: dry, wet and snowy. (ZIP) [file pone.0310858.s001.zip › weather1_data/dry_road/1627266215163.jpg]

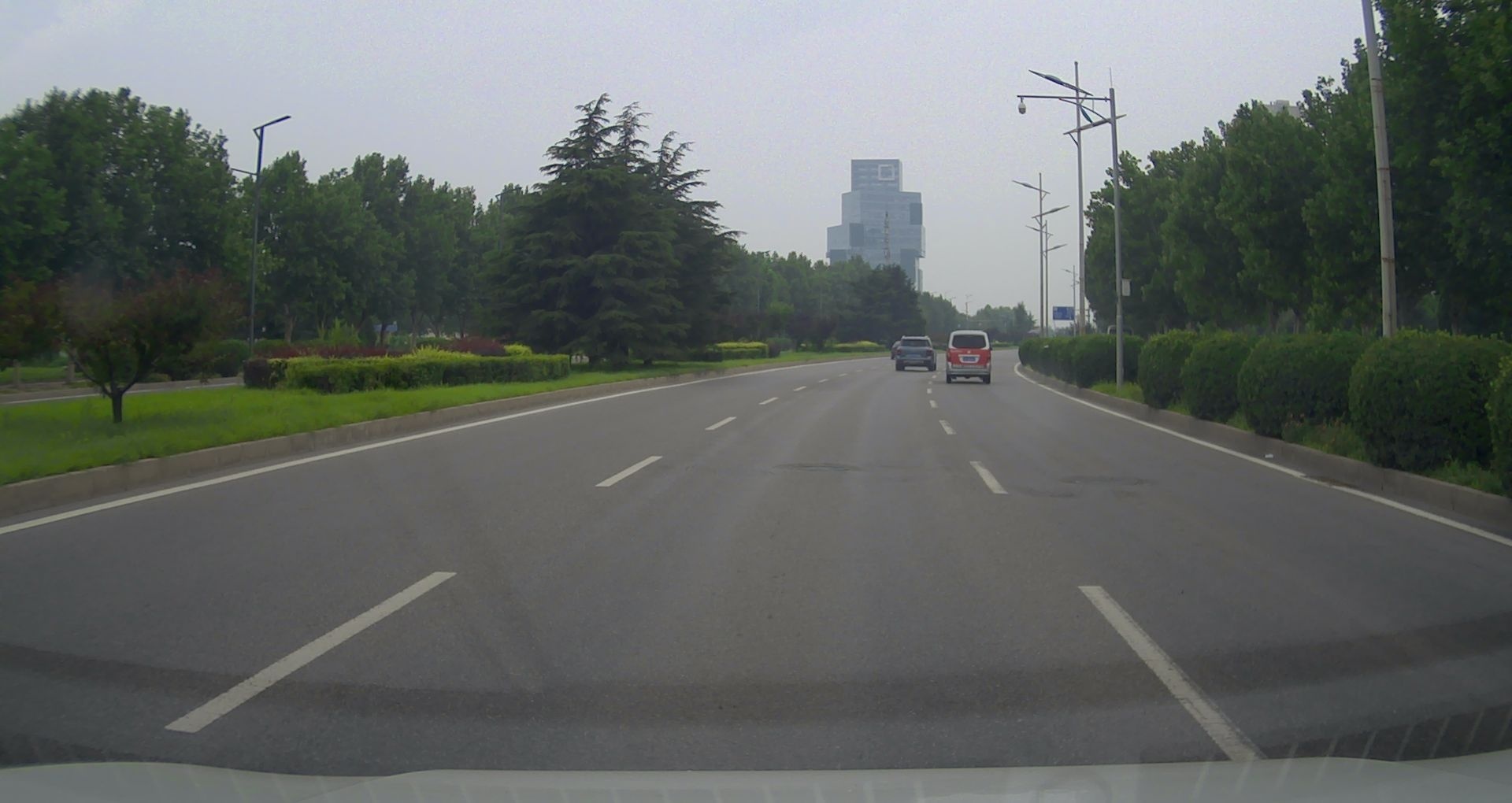

Supplement: S1 Dataset — All collected images were collected together, labeled and summarized one by one, and resulting classification results were roughly classified into three major categories: dry, wet and snowy. (ZIP) [file pone.0310858.s001.zip › weather1_data/dry_road/1627266217325.jpg]

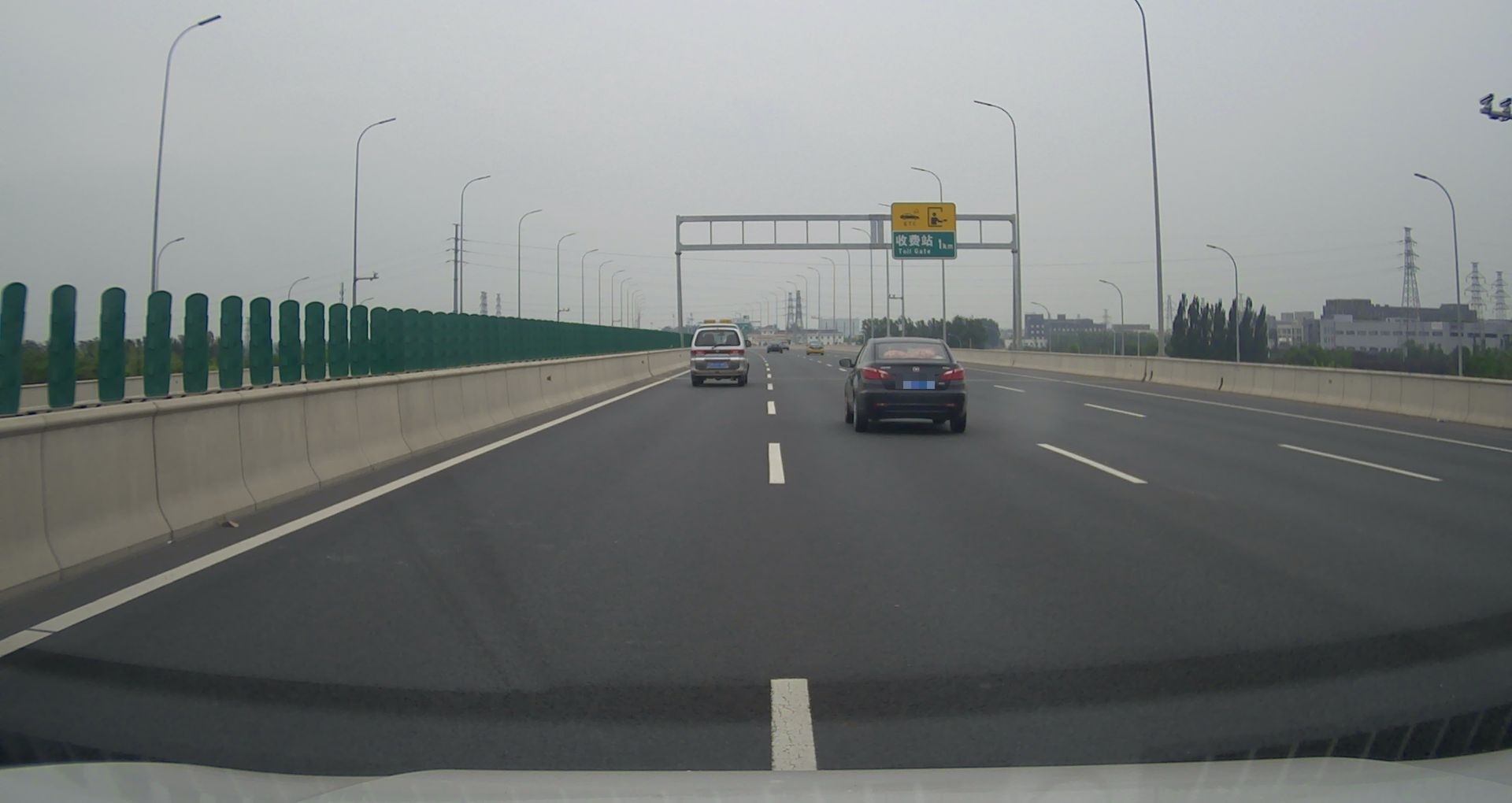

Supplement: S1 Dataset — All collected images were collected together, labeled and summarized one by one, and resulting classification results were roughly classified into three major categories: dry, wet and snowy. (ZIP) [file pone.0310858.s001.zip › weather1_data/dry_road/1627268277043.jpg]

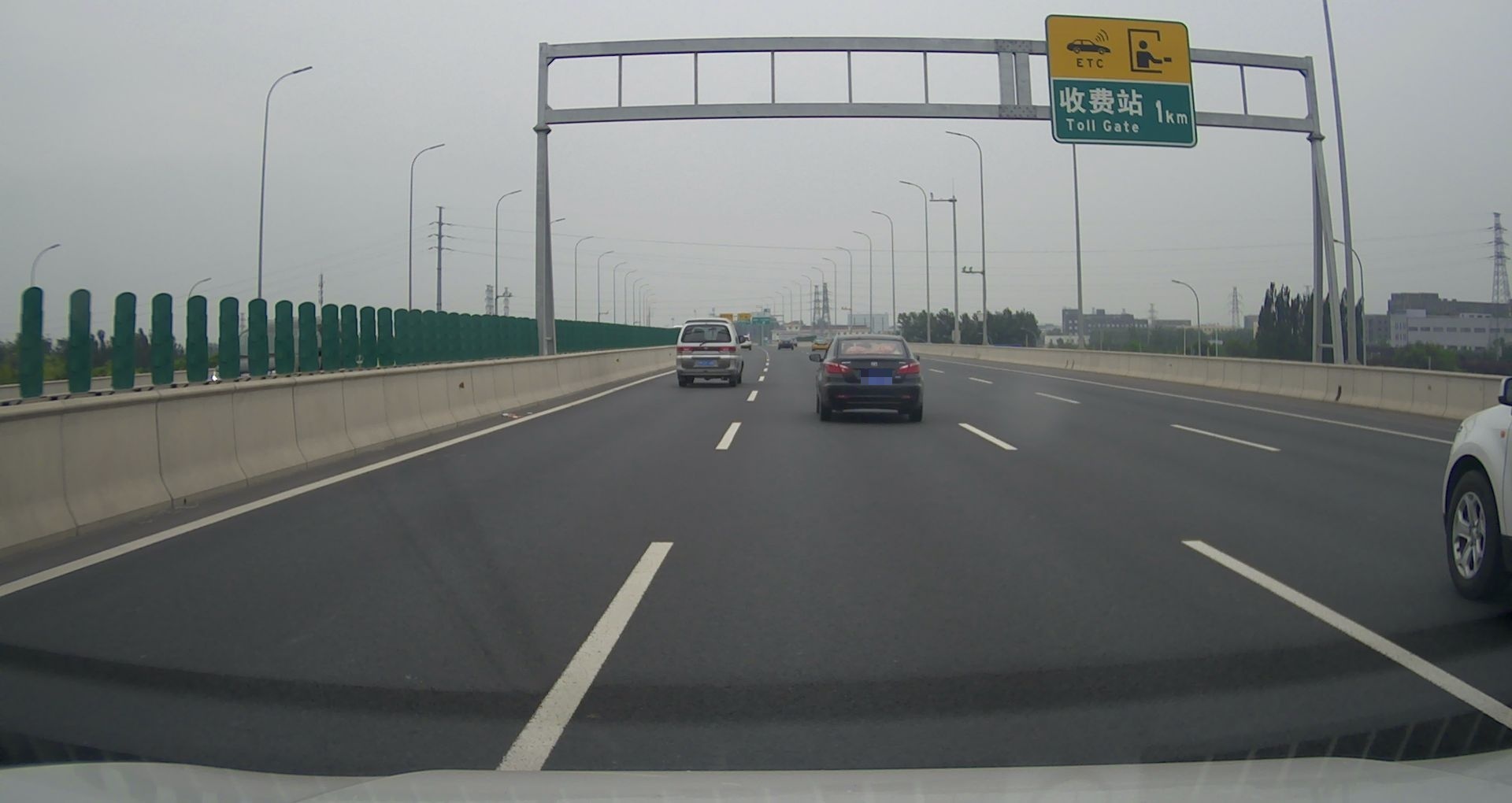

Supplement: S1 Dataset — All collected images were collected together, labeled and summarized one by one, and resulting classification results were roughly classified into three major categories: dry, wet and snowy. (ZIP) [file pone.0310858.s001.zip › weather1_data/dry_road/1627268279118.jpg]

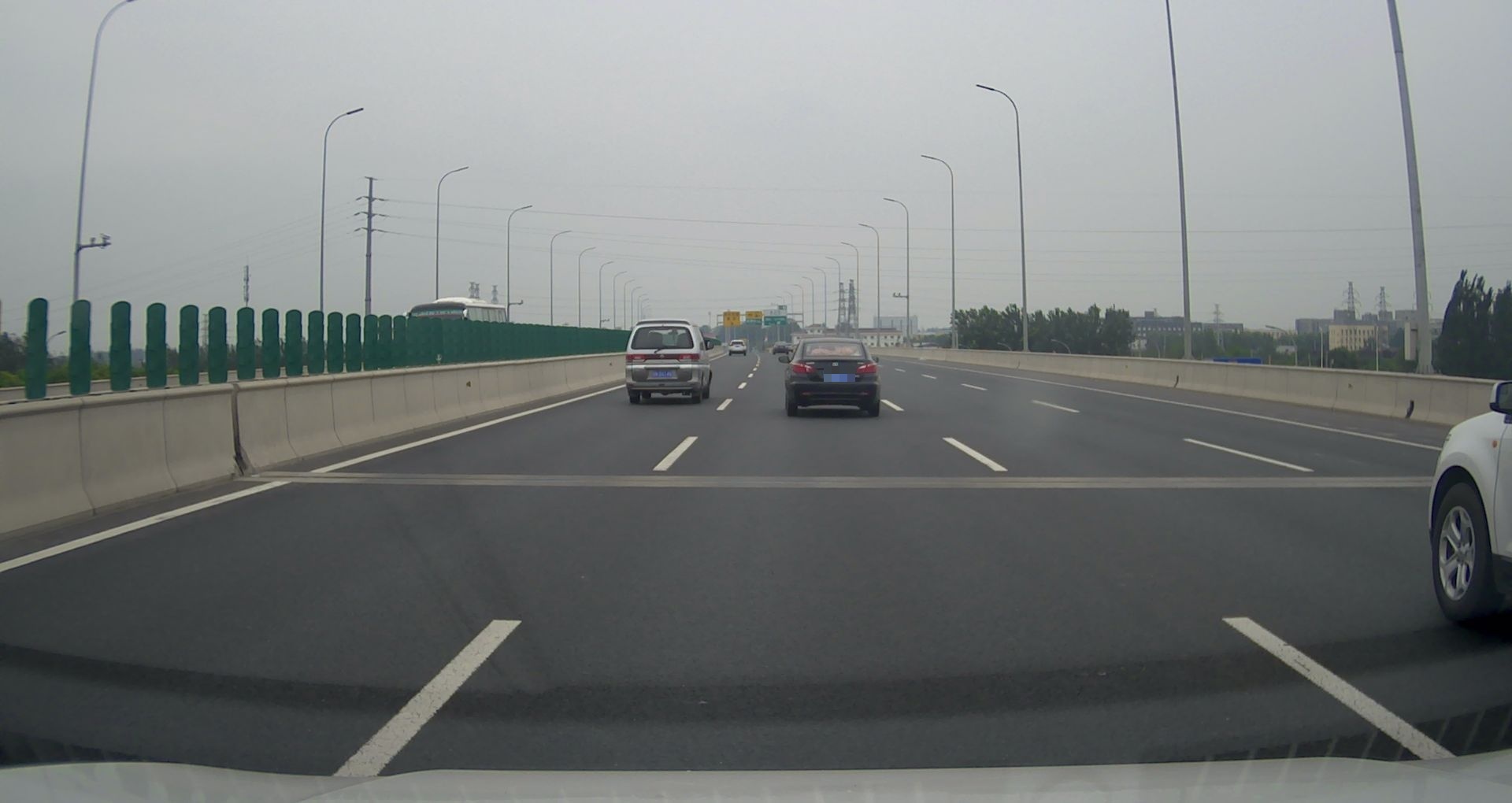

Supplement: S1 Dataset — All collected images were collected together, labeled and summarized one by one, and resulting classification results were roughly classified into three major categories: dry, wet and snowy. (ZIP) [file pone.0310858.s001.zip › weather1_data/dry_road/1627268282623.jpg]

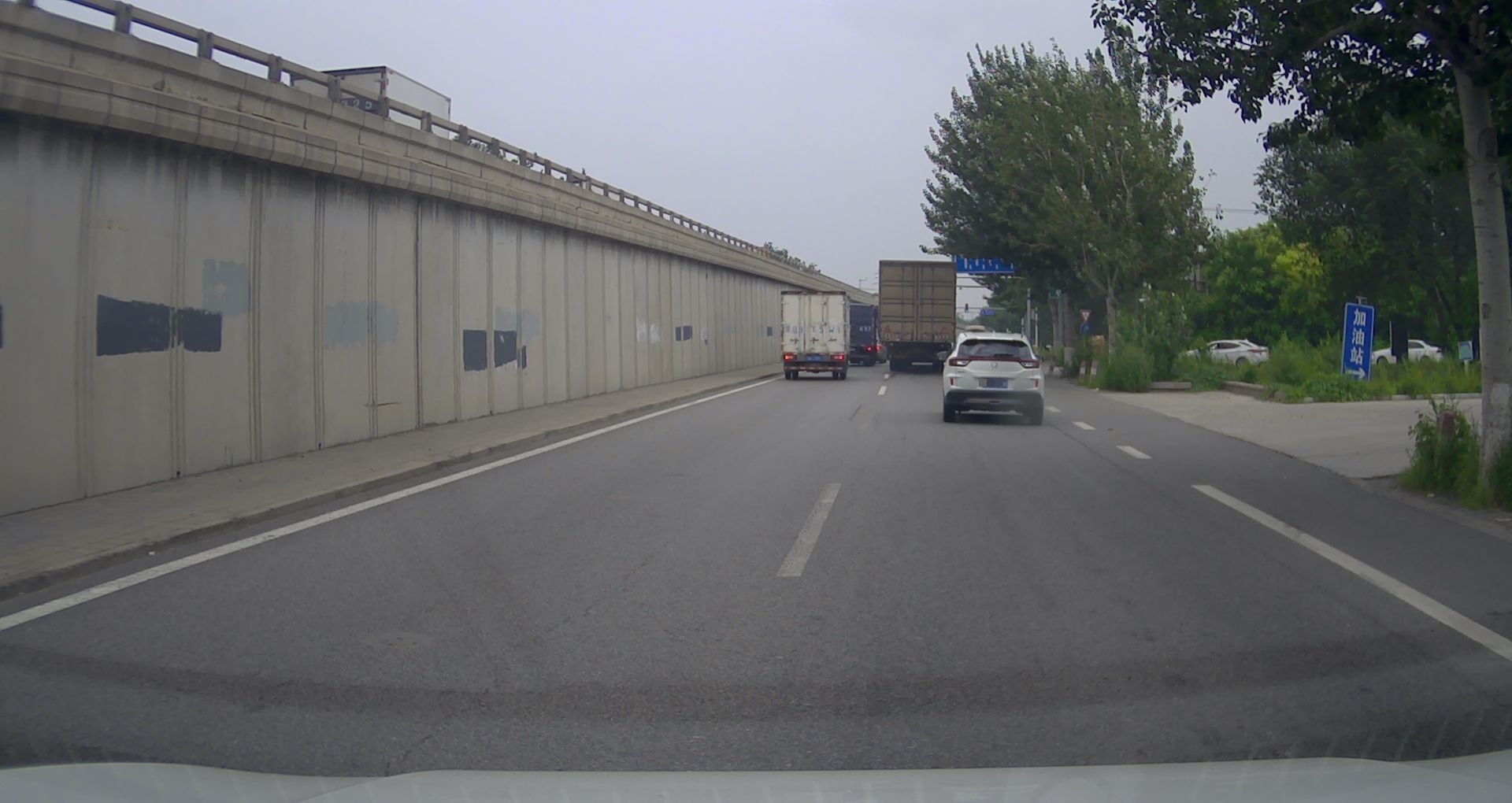

Supplement: S1 Dataset — All collected images were collected together, labeled and summarized one by one, and resulting classification results were roughly classified into three major categories: dry, wet and snowy. (ZIP) [file pone.0310858.s001.zip › weather1_data/dry_road/1627269262431.jpg]

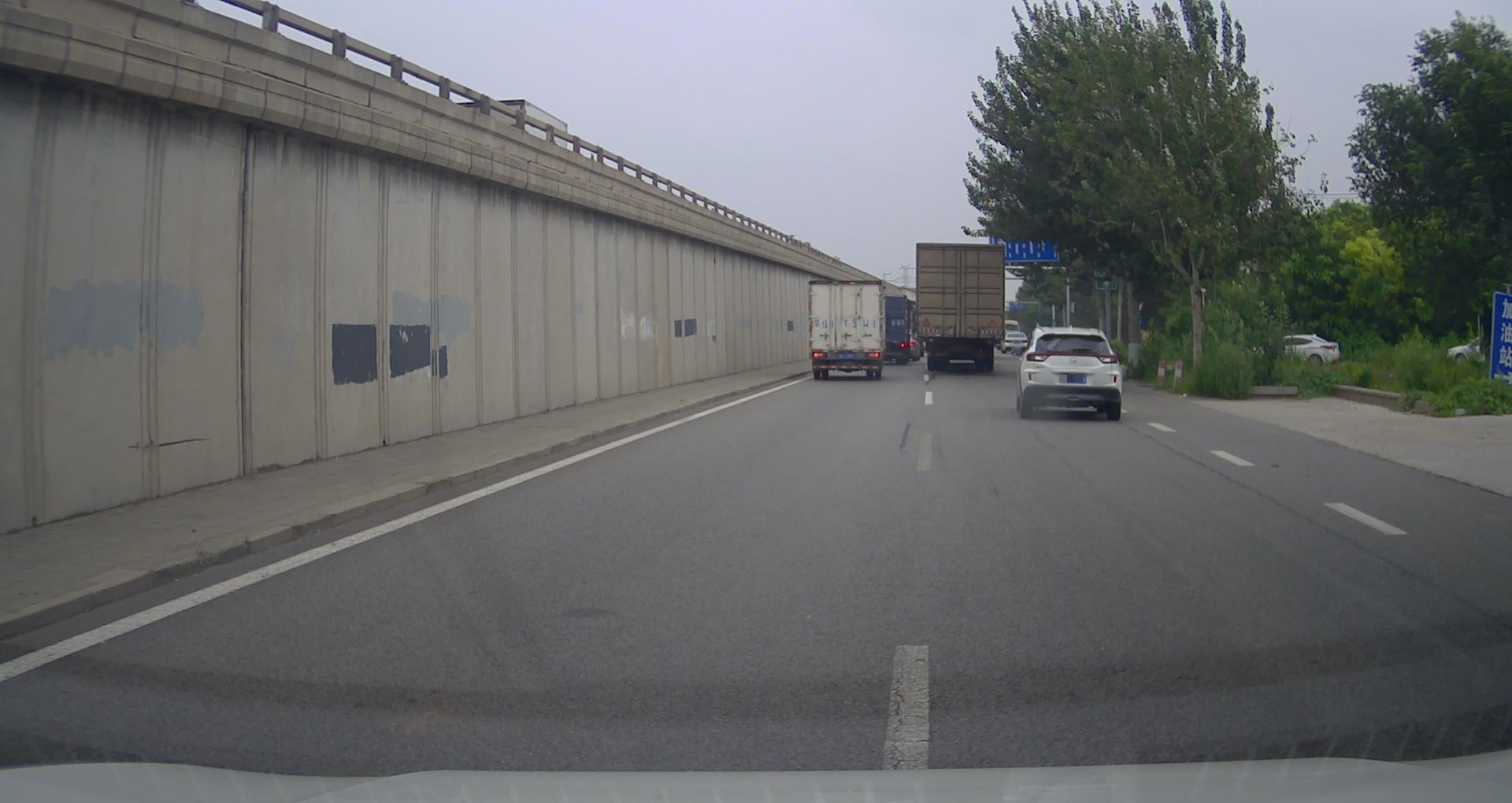

Supplement: S1 Dataset — All collected images were collected together, labeled and summarized one by one, and resulting classification results were roughly classified into three major categories: dry, wet and snowy. (ZIP) [file pone.0310858.s001.zip › weather1_data/dry_road/1627269263114.jpg]

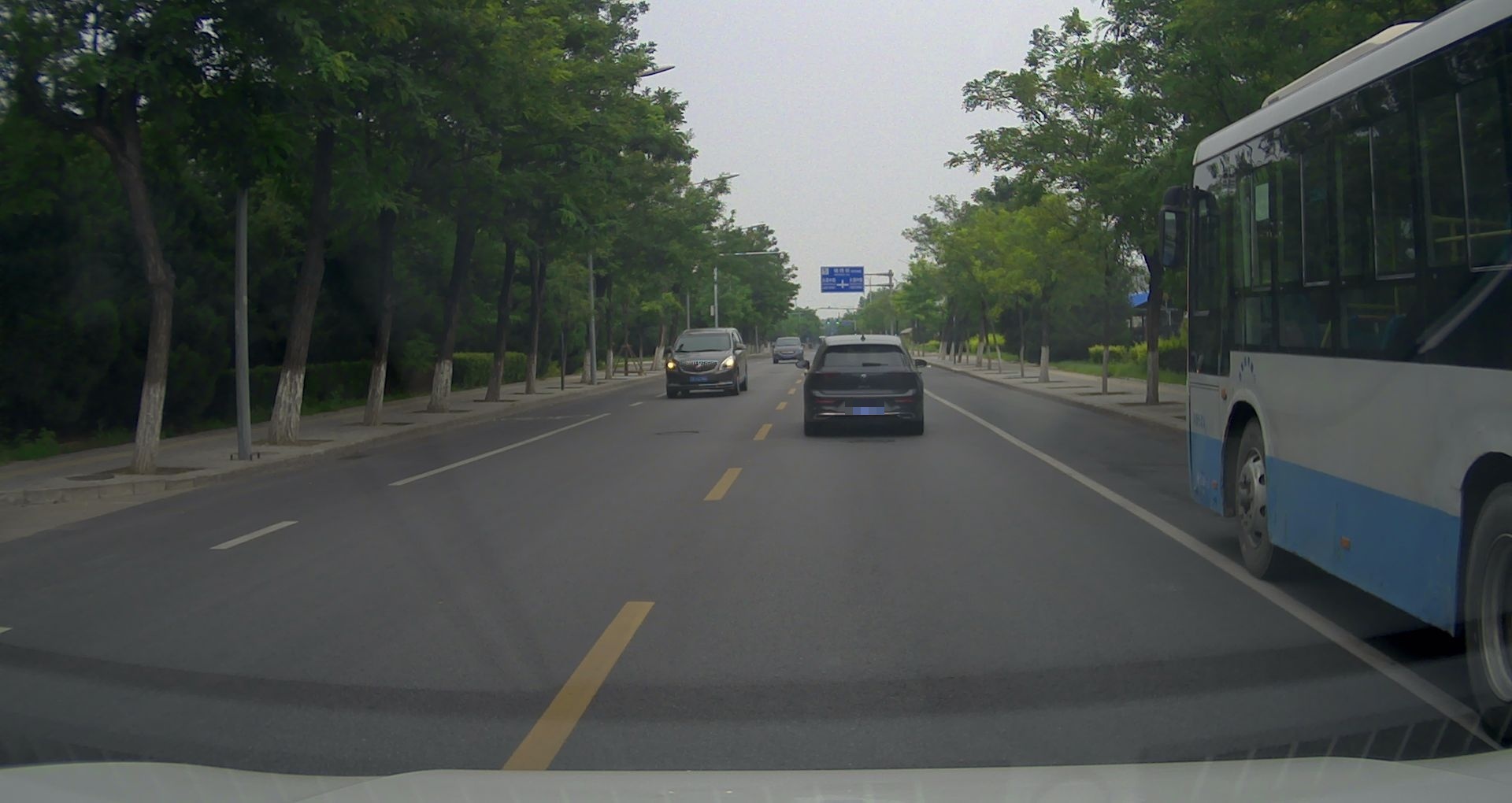

Supplement: S1 Dataset — All collected images were collected together, labeled and summarized one by one, and resulting classification results were roughly classified into three major categories: dry, wet and snowy. (ZIP) [file pone.0310858.s001.zip › weather1_data/dry_road/1627276179075.jpg]

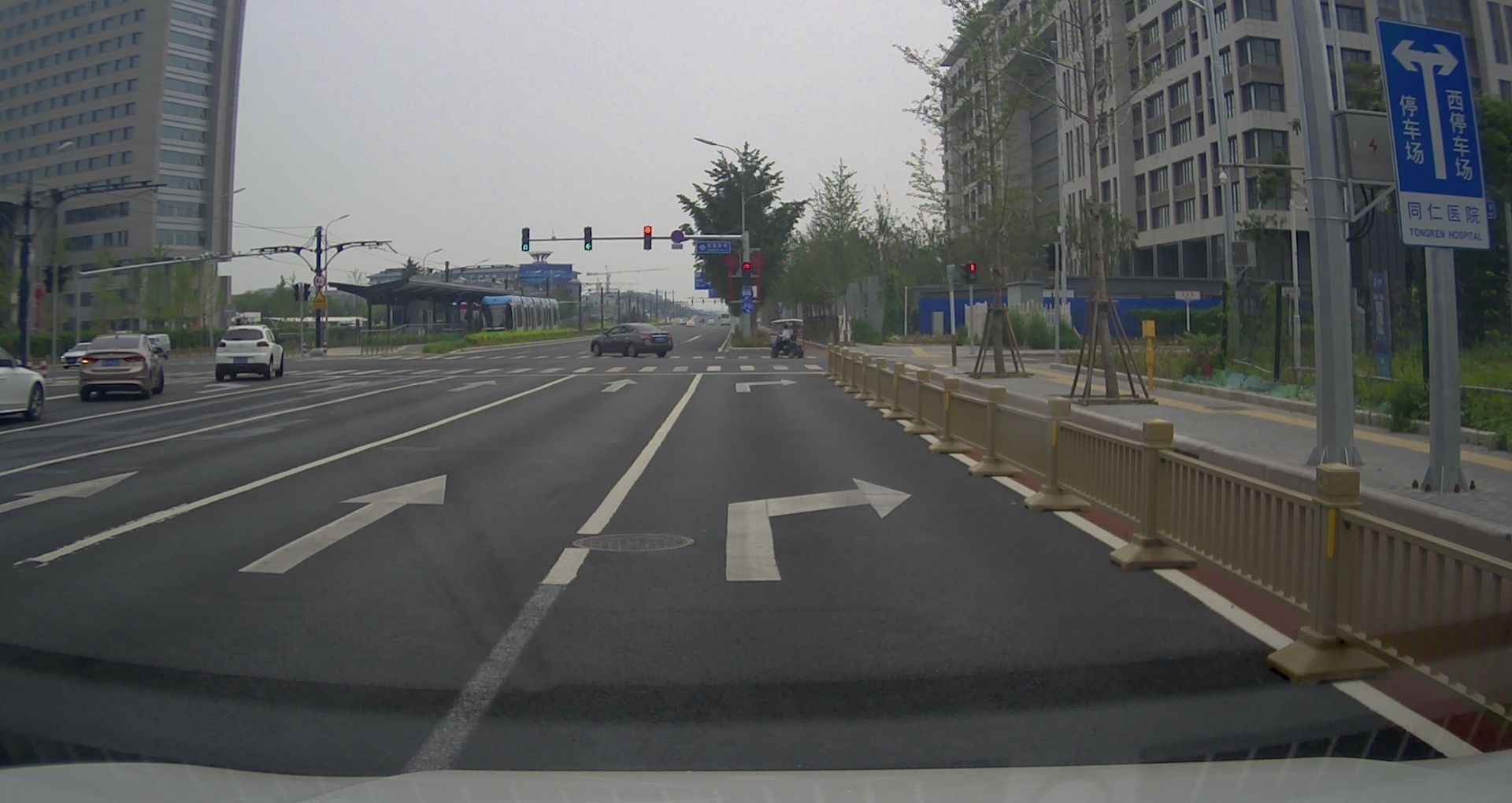

Supplement: S1 Dataset — All collected images were collected together, labeled and summarized one by one, and resulting classification results were roughly classified into three major categories: dry, wet and snowy. (ZIP) [file pone.0310858.s001.zip › weather1_data/dry_road/1627281663833.jpg]

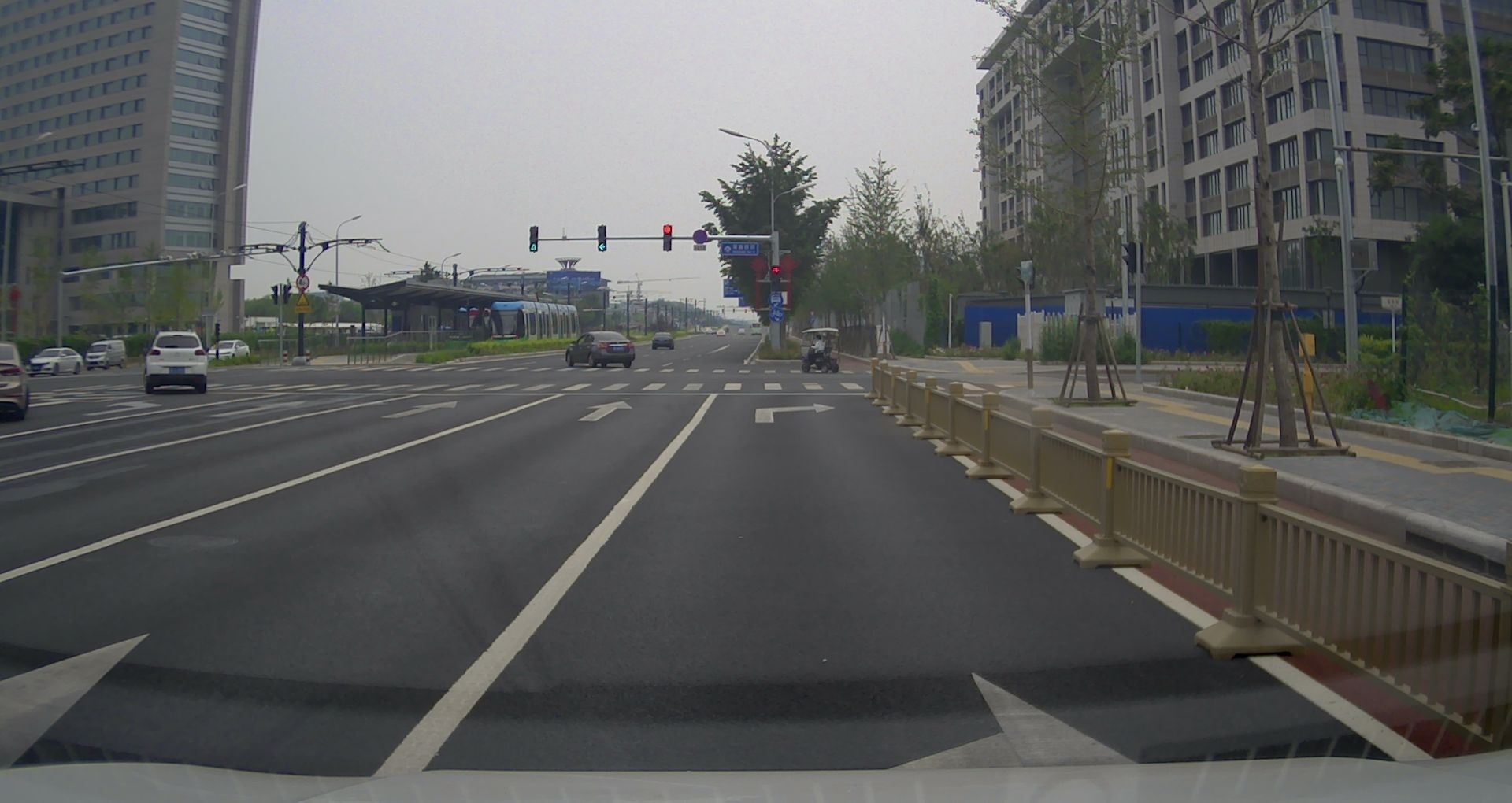

Supplement: S1 Dataset — All collected images were collected together, labeled and summarized one by one, and resulting classification results were roughly classified into three major categories: dry, wet and snowy. (ZIP) [file pone.0310858.s001.zip › weather1_data/dry_road/1627281664530.jpg]

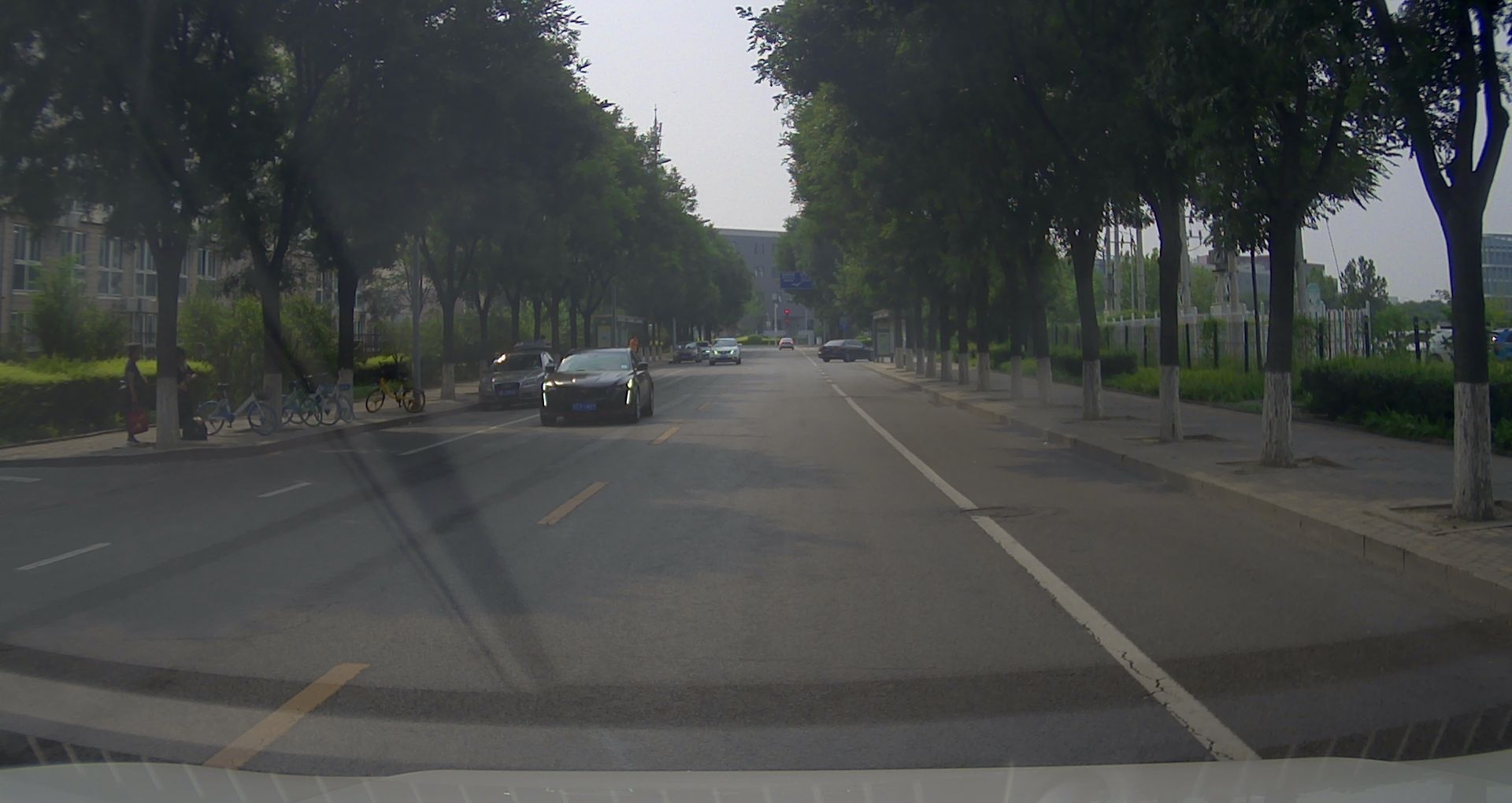

Supplement: S1 Dataset — All collected images were collected together, labeled and summarized one by one, and resulting classification results were roughly classified into three major categories: dry, wet and snowy. (ZIP) [file pone.0310858.s001.zip › weather1_data/dry_road/1627285067187.jpg]

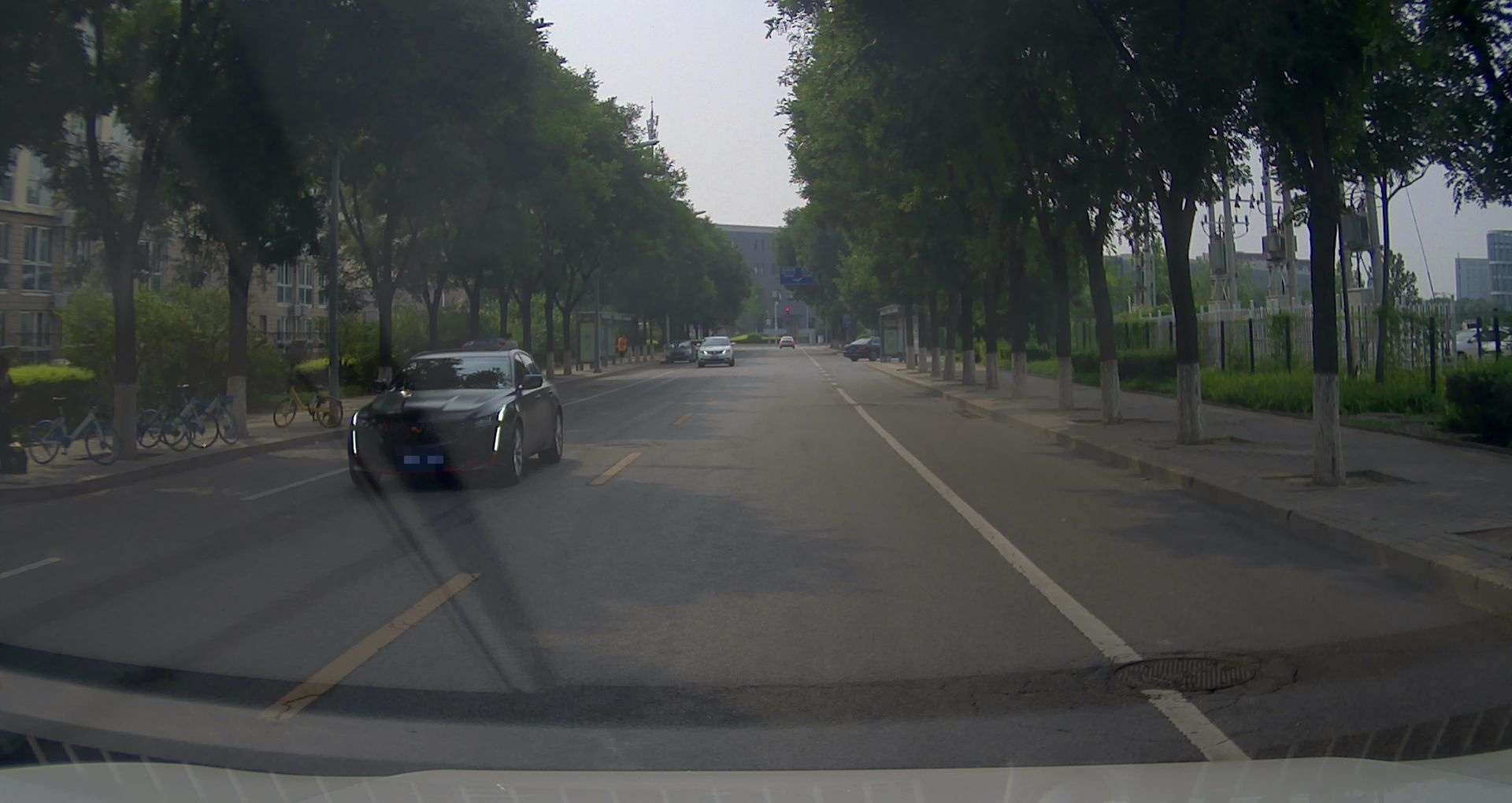

Supplement: S1 Dataset — All collected images were collected together, labeled and summarized one by one, and resulting classification results were roughly classified into three major categories: dry, wet and snowy. (ZIP) [file pone.0310858.s001.zip › weather1_data/dry_road/1627285067661.jpg]

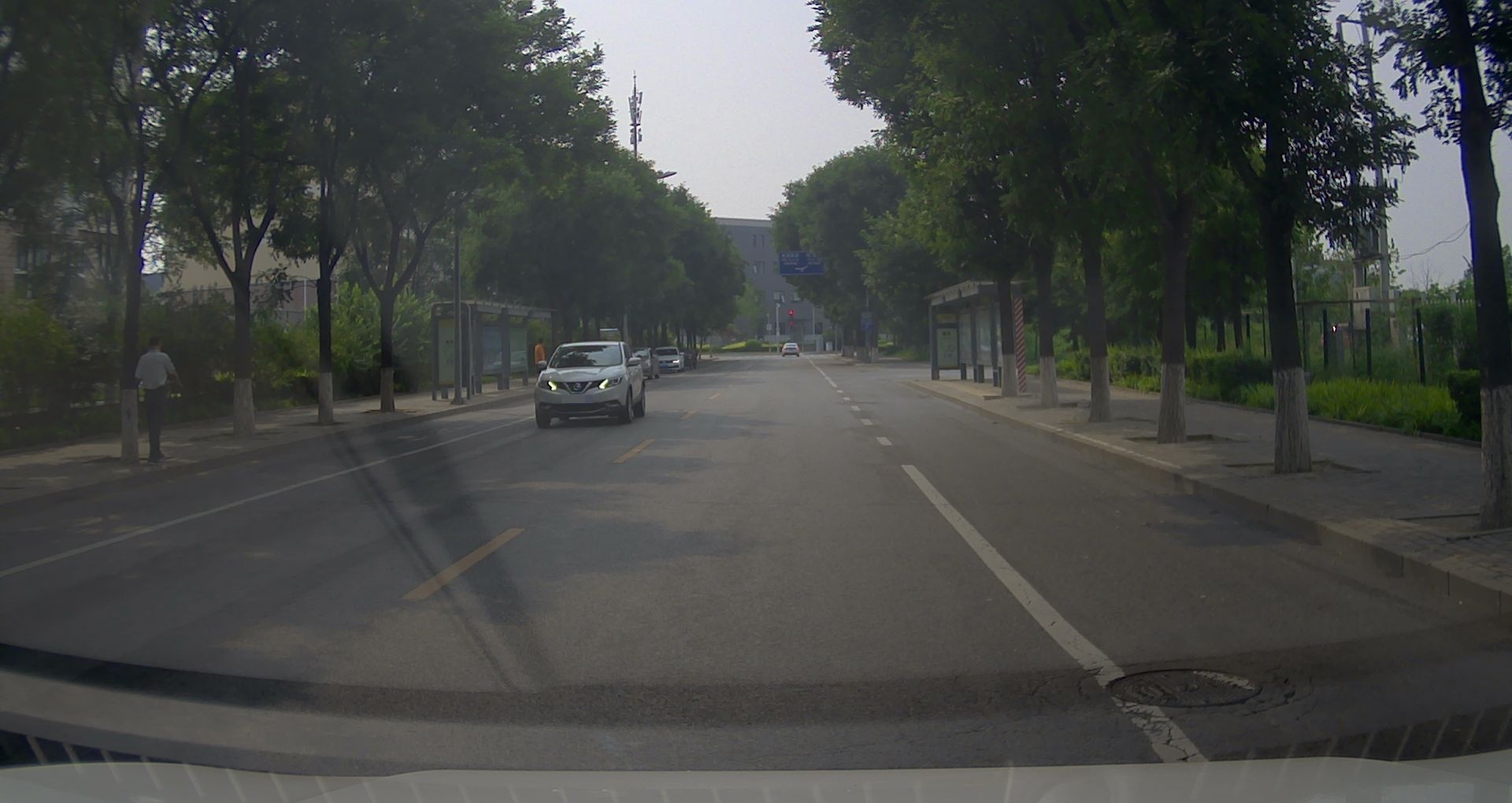

Supplement: S1 Dataset — All collected images were collected together, labeled and summarized one by one, and resulting classification results were roughly classified into three major categories: dry, wet and snowy. (ZIP) [file pone.0310858.s001.zip › weather1_data/dry_road/1627285069766.jpg]

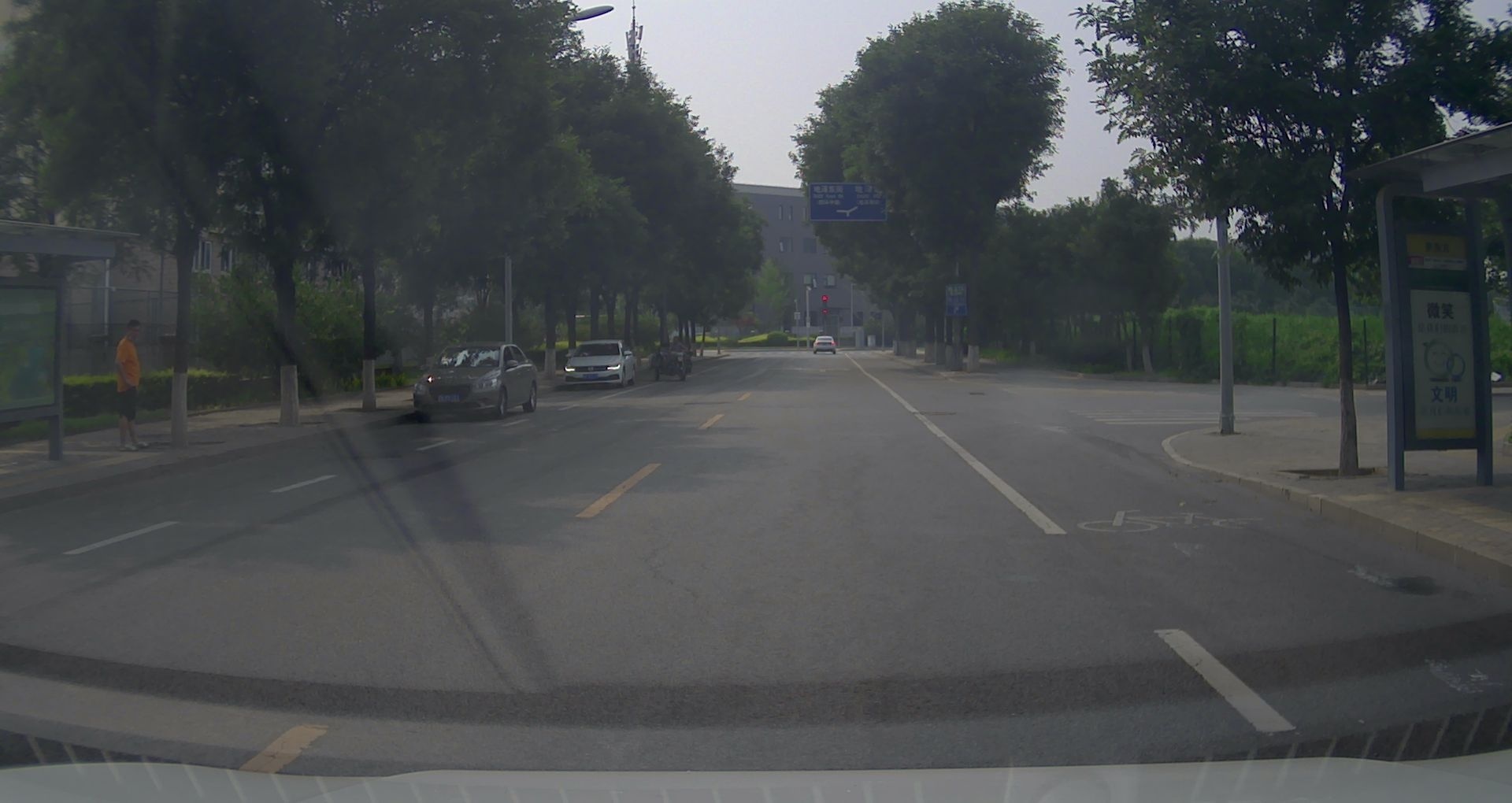

Supplement: S1 Dataset — All collected images were collected together, labeled and summarized one by one, and resulting classification results were roughly classified into three major categories: dry, wet and snowy. (ZIP) [file pone.0310858.s001.zip › weather1_data/dry_road/1627285072542.jpg]

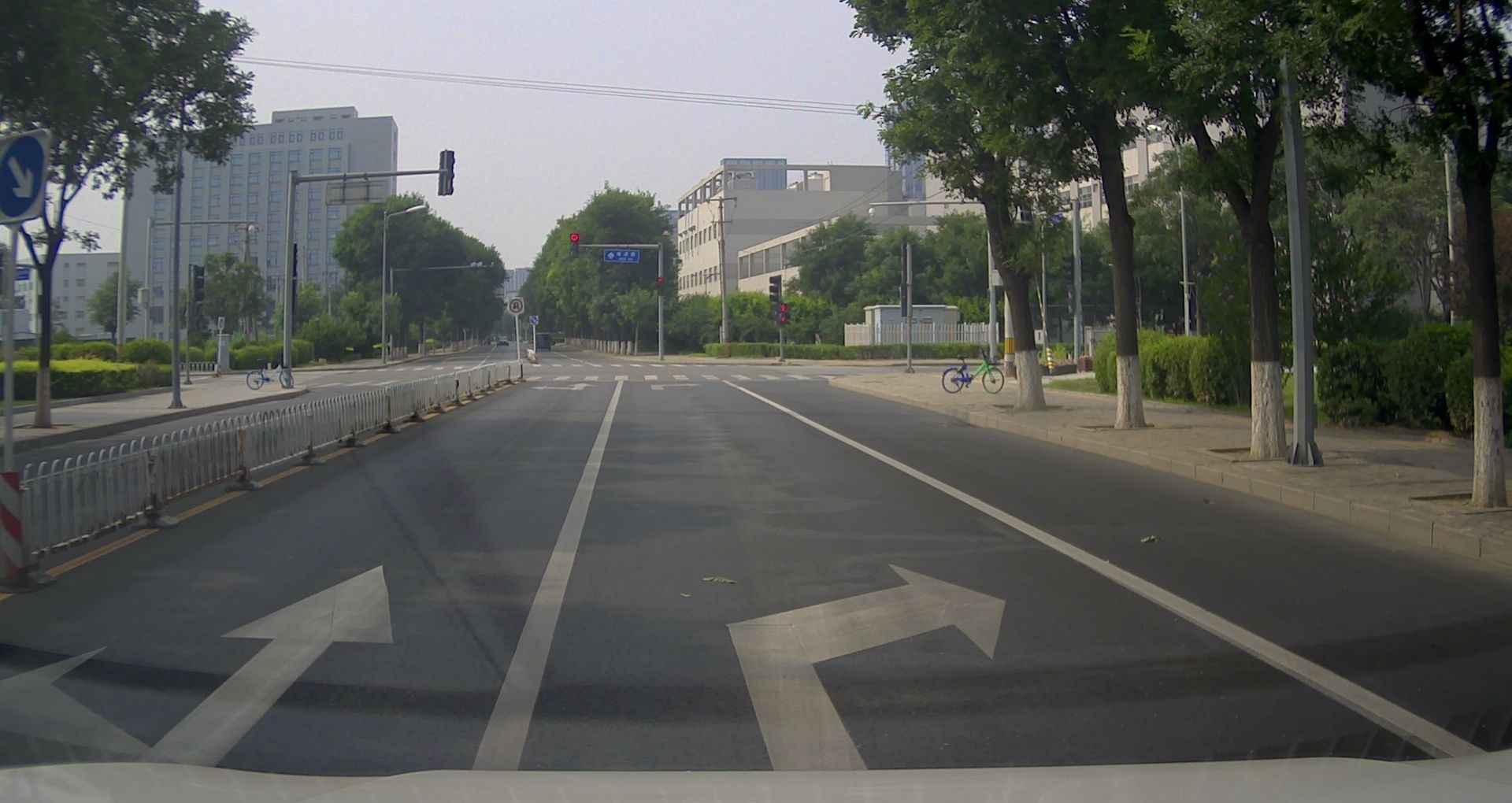

Supplement: S1 Dataset — All collected images were collected together, labeled and summarized one by one, and resulting classification results were roughly classified into three major categories: dry, wet and snowy. (ZIP) [file pone.0310858.s001.zip › weather1_data/dry_road/1627285114371.jpg]

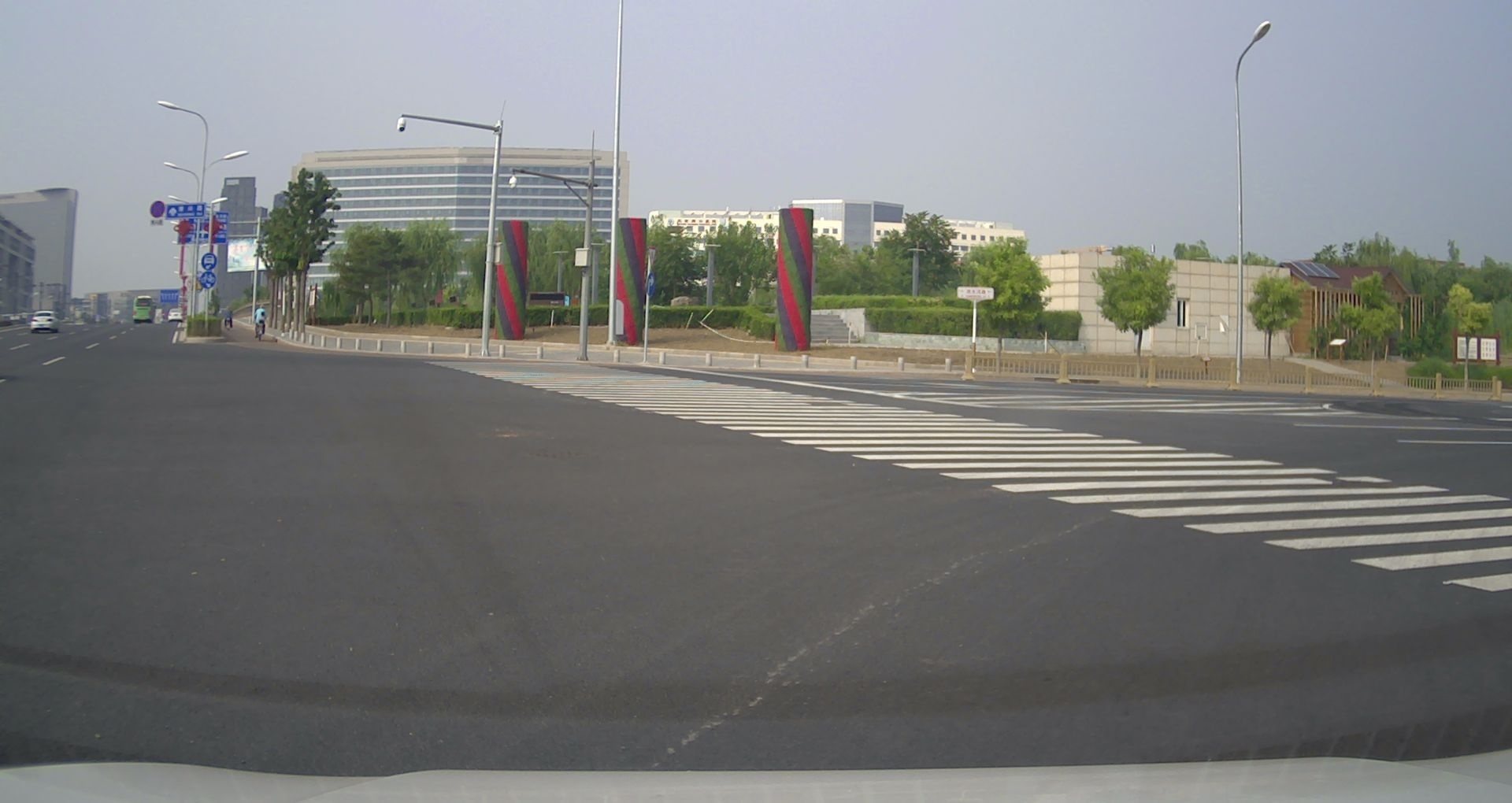

Supplement: S1 Dataset — All collected images were collected together, labeled and summarized one by one, and resulting classification results were roughly classified into three major categories: dry, wet and snowy. (ZIP) [file pone.0310858.s001.zip › weather1_data/dry_road/1627285596937.jpg]

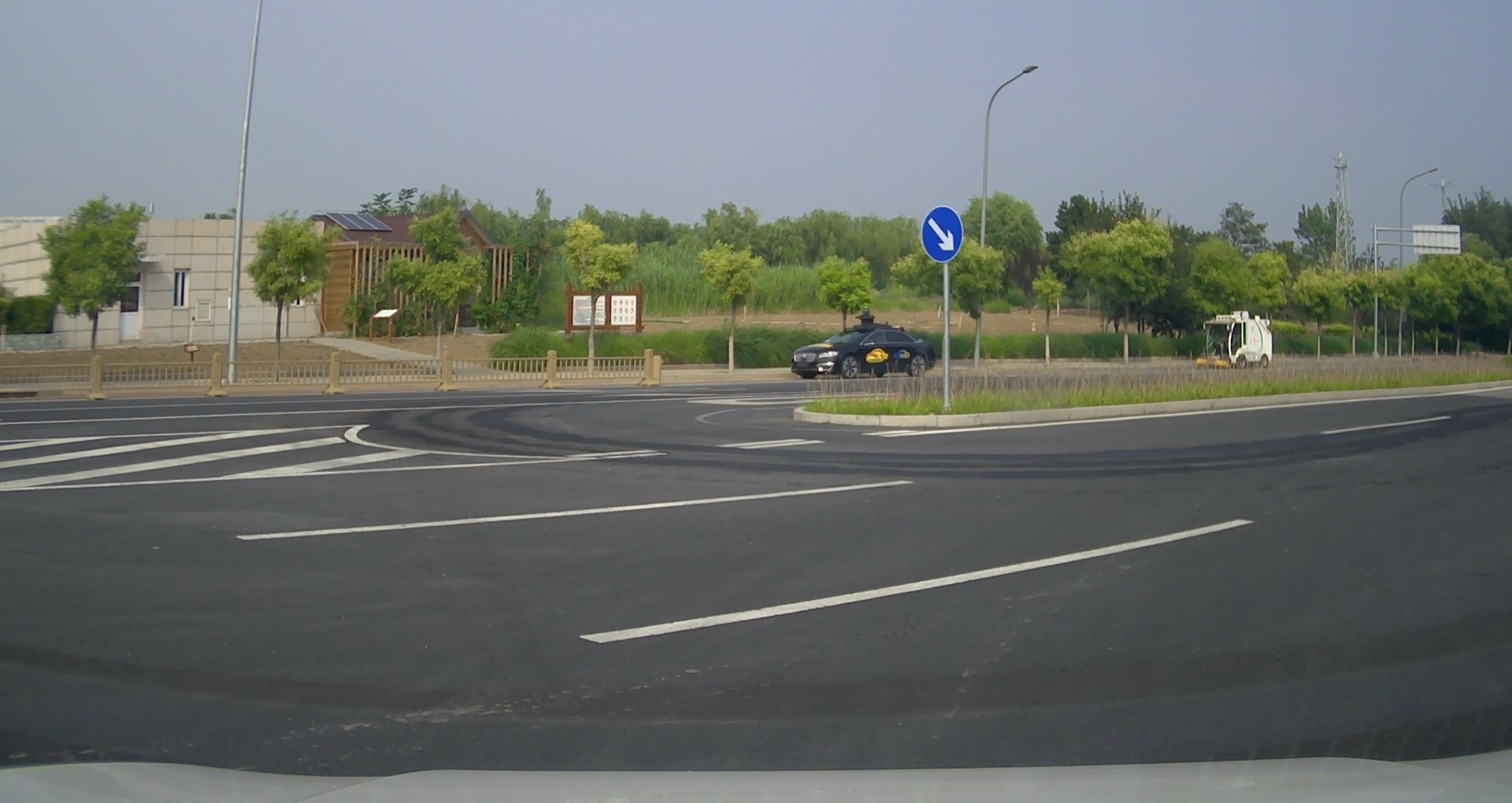

Supplement: S1 Dataset — All collected images were collected together, labeled and summarized one by one, and resulting classification results were roughly classified into three major categories: dry, wet and snowy. (ZIP) [file pone.0310858.s001.zip › weather1_data/dry_road/1627285599085.jpg]

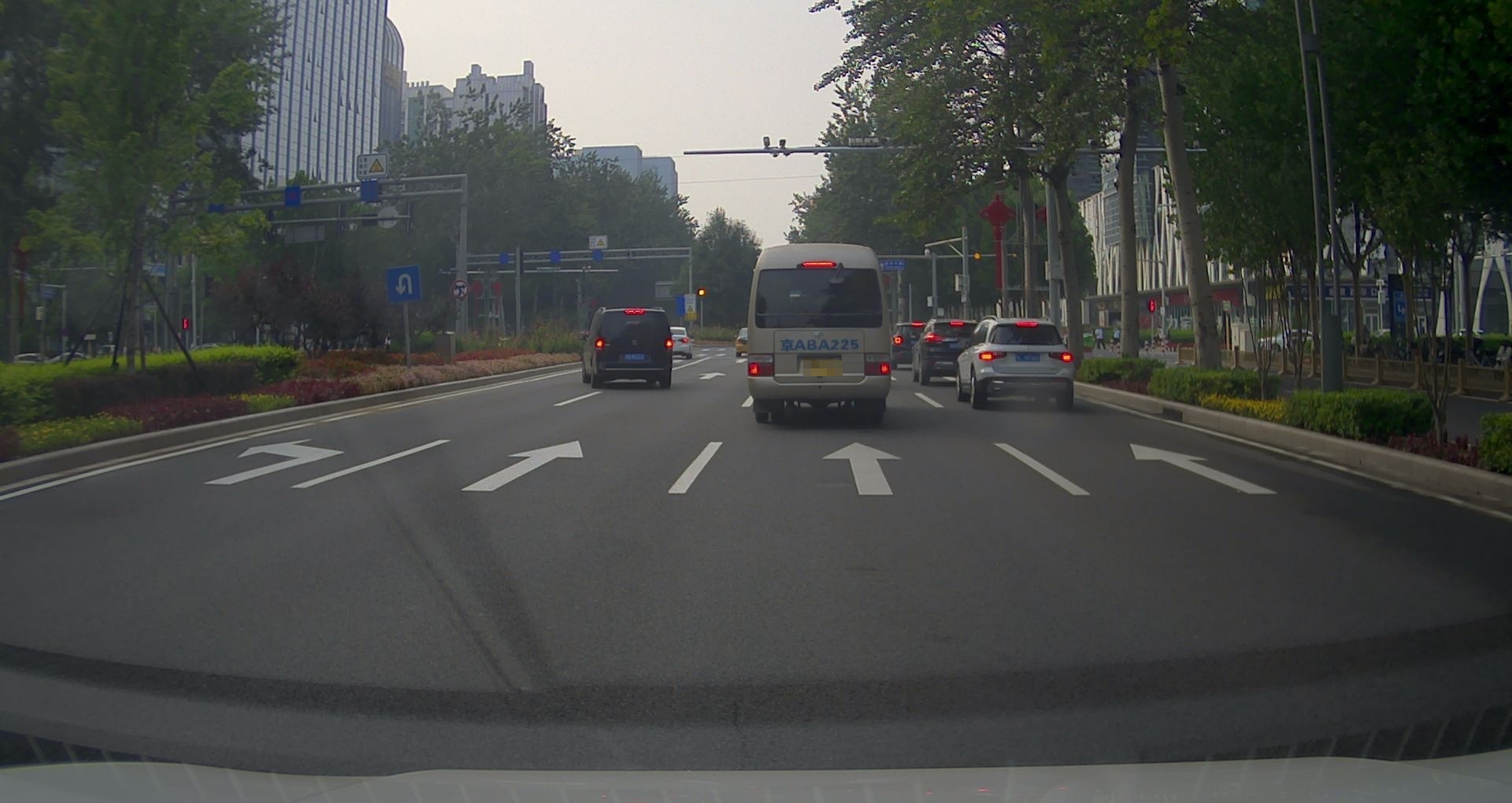

Supplement: S1 Dataset — All collected images were collected together, labeled and summarized one by one, and resulting classification results were roughly classified into three major categories: dry, wet and snowy. (ZIP) [file pone.0310858.s001.zip › weather1_data/dry_road/1627288294759.jpg]

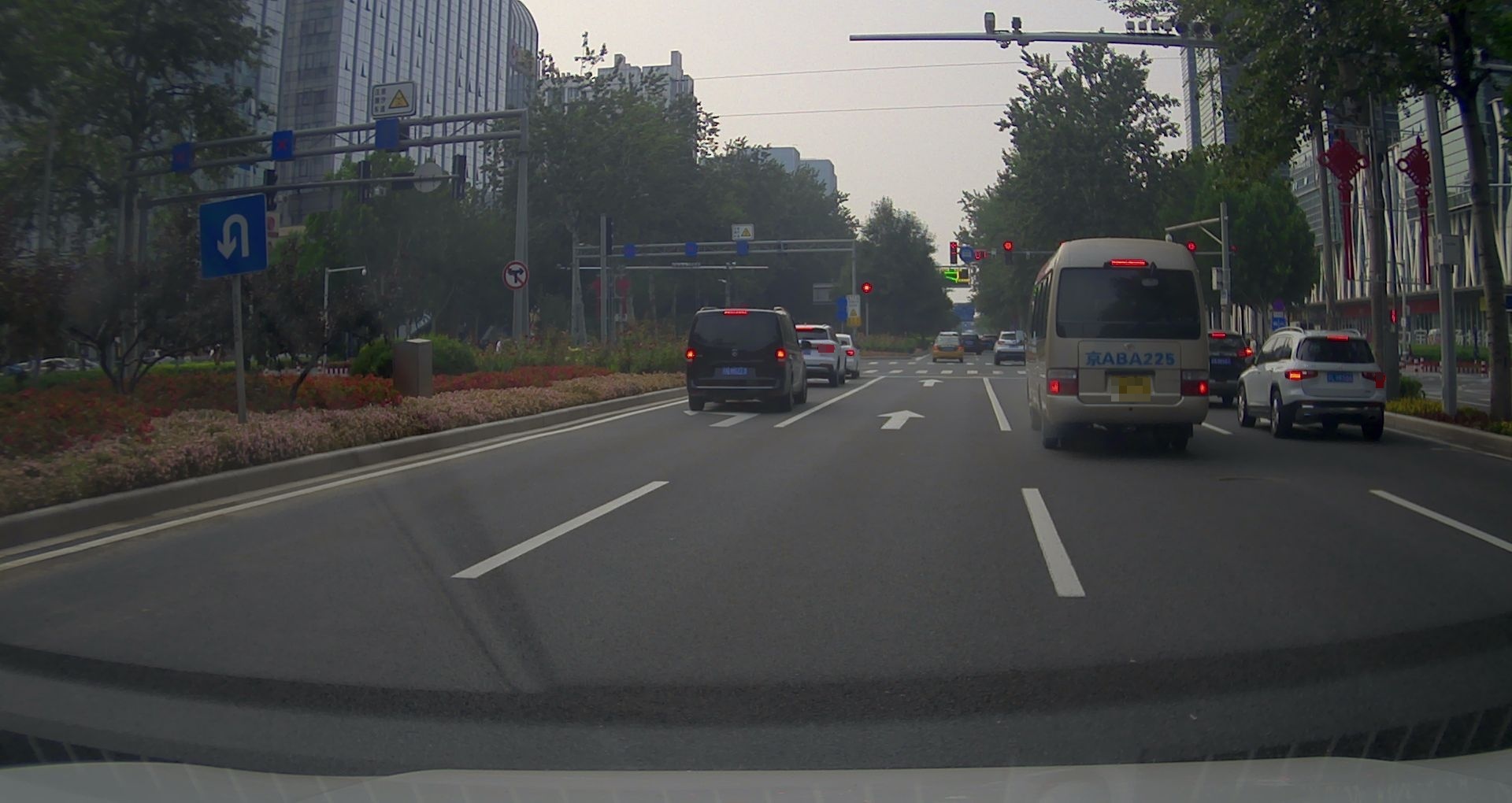

Supplement: S1 Dataset — All collected images were collected together, labeled and summarized one by one, and resulting classification results were roughly classified into three major categories: dry, wet and snowy. (ZIP) [file pone.0310858.s001.zip › weather1_data/dry_road/1627288296935.jpg]

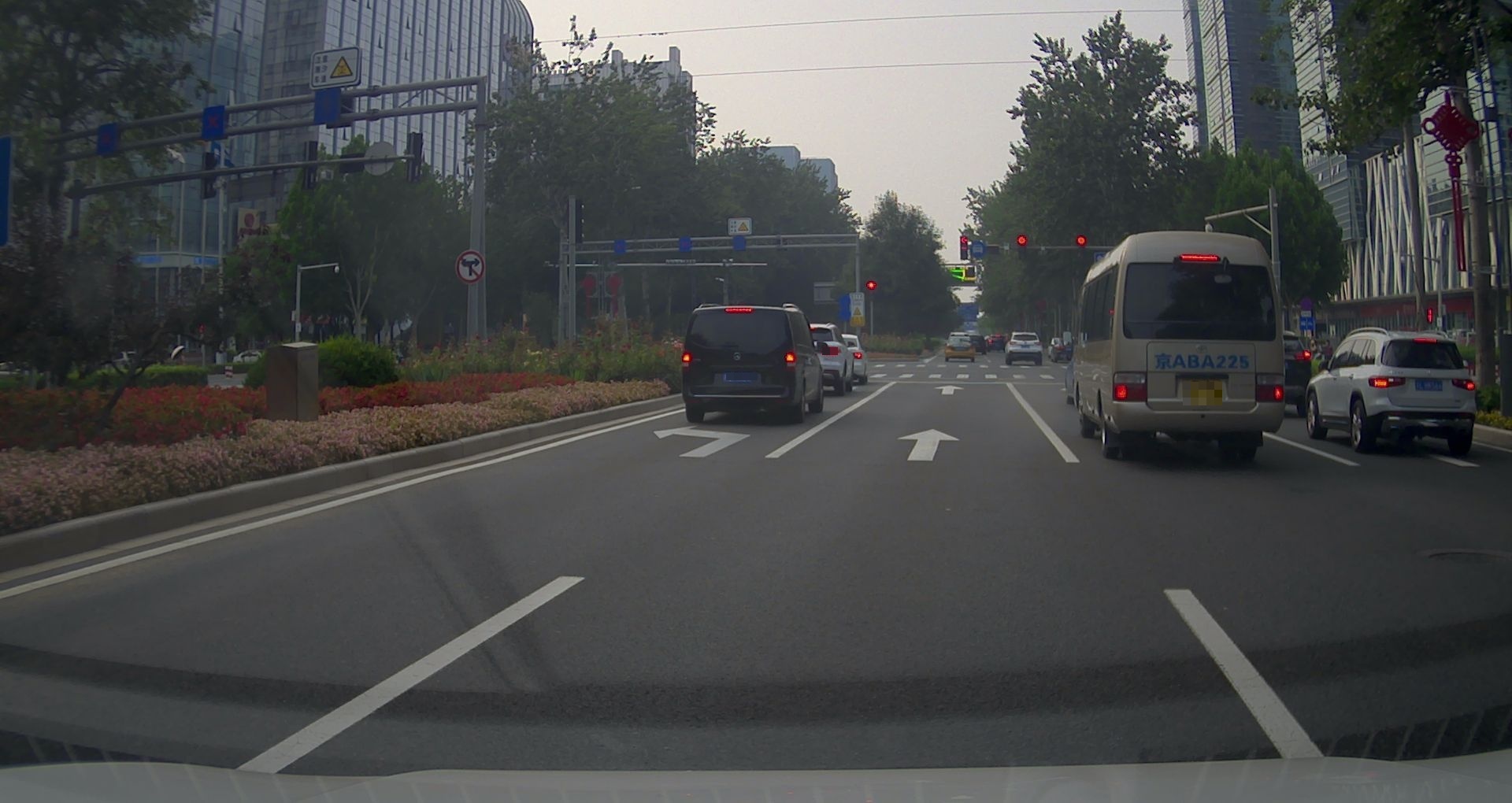

Supplement: S1 Dataset — All collected images were collected together, labeled and summarized one by one, and resulting classification results were roughly classified into three major categories: dry, wet and snowy. (ZIP) [file pone.0310858.s001.zip › weather1_data/dry_road/1627288297615.jpg]

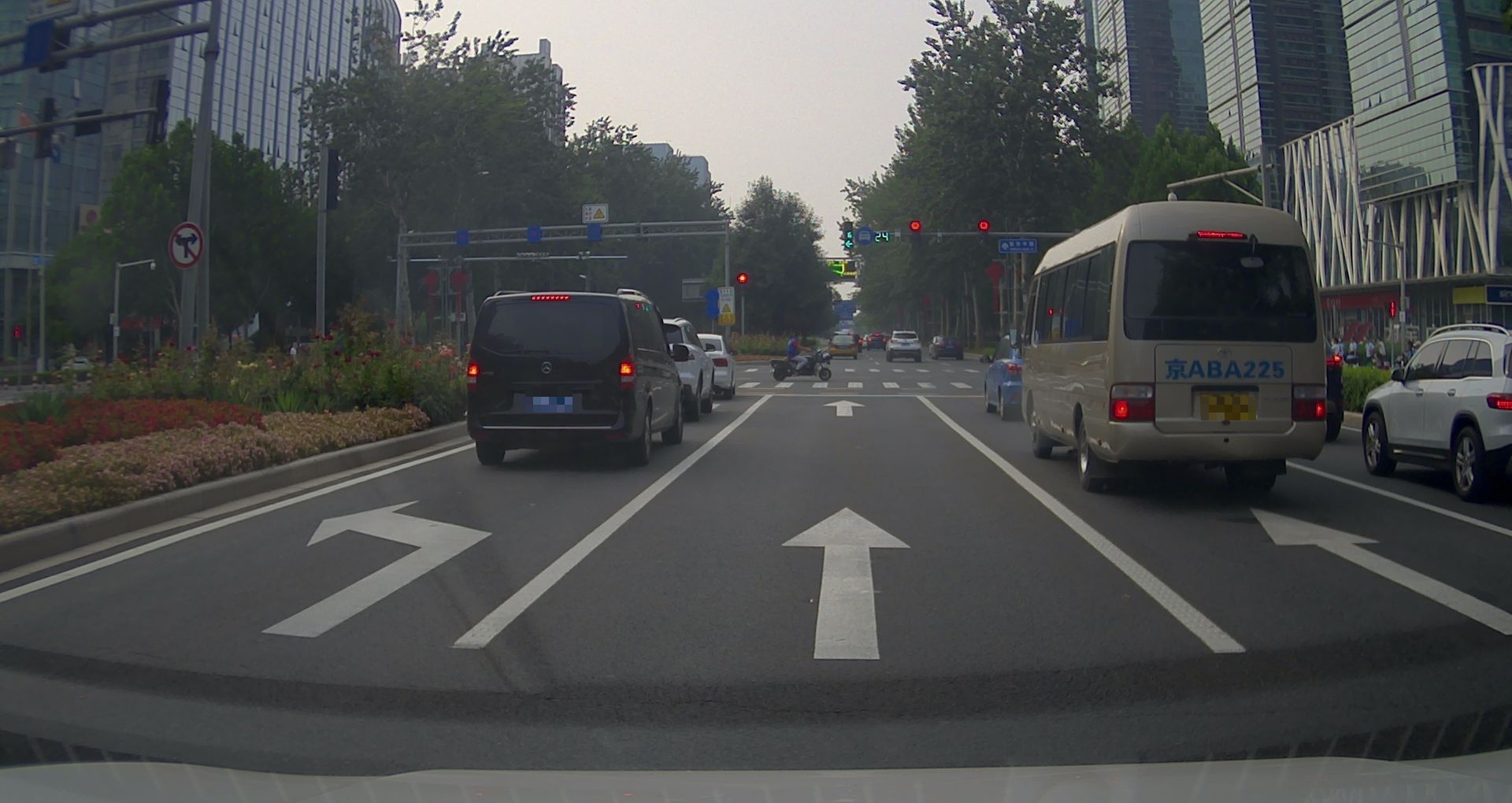

Supplement: S1 Dataset — All collected images were collected together, labeled and summarized one by one, and resulting classification results were roughly classified into three major categories: dry, wet and snowy. (ZIP) [file pone.0310858.s001.zip › weather1_data/dry_road/1627288299007.jpg]

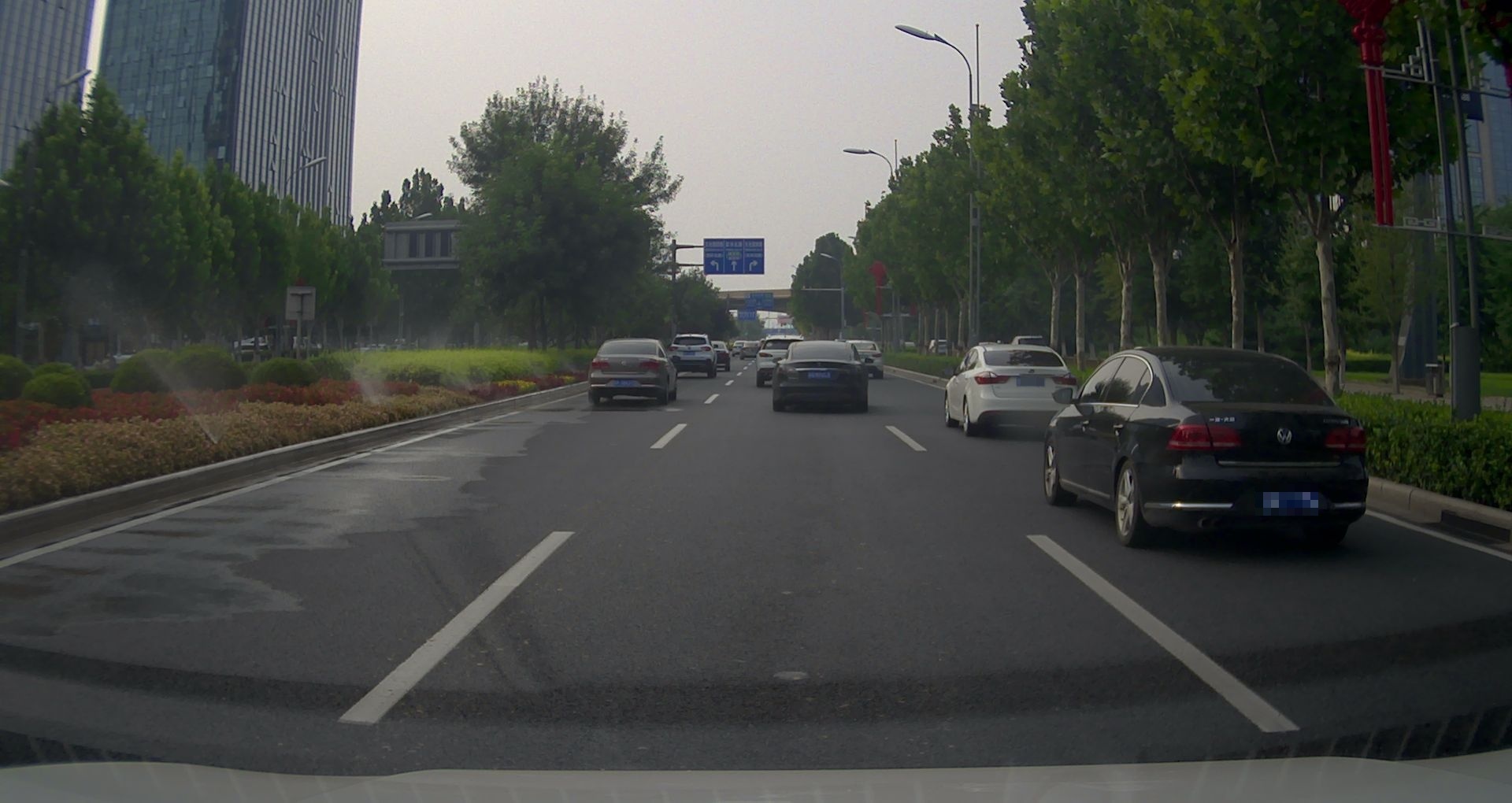

Supplement: S1 Dataset — All collected images were collected together, labeled and summarized one by one, and resulting classification results were roughly classified into three major categories: dry, wet and snowy. (ZIP) [file pone.0310858.s001.zip › weather1_data/dry_road/1627288449125.jpg]

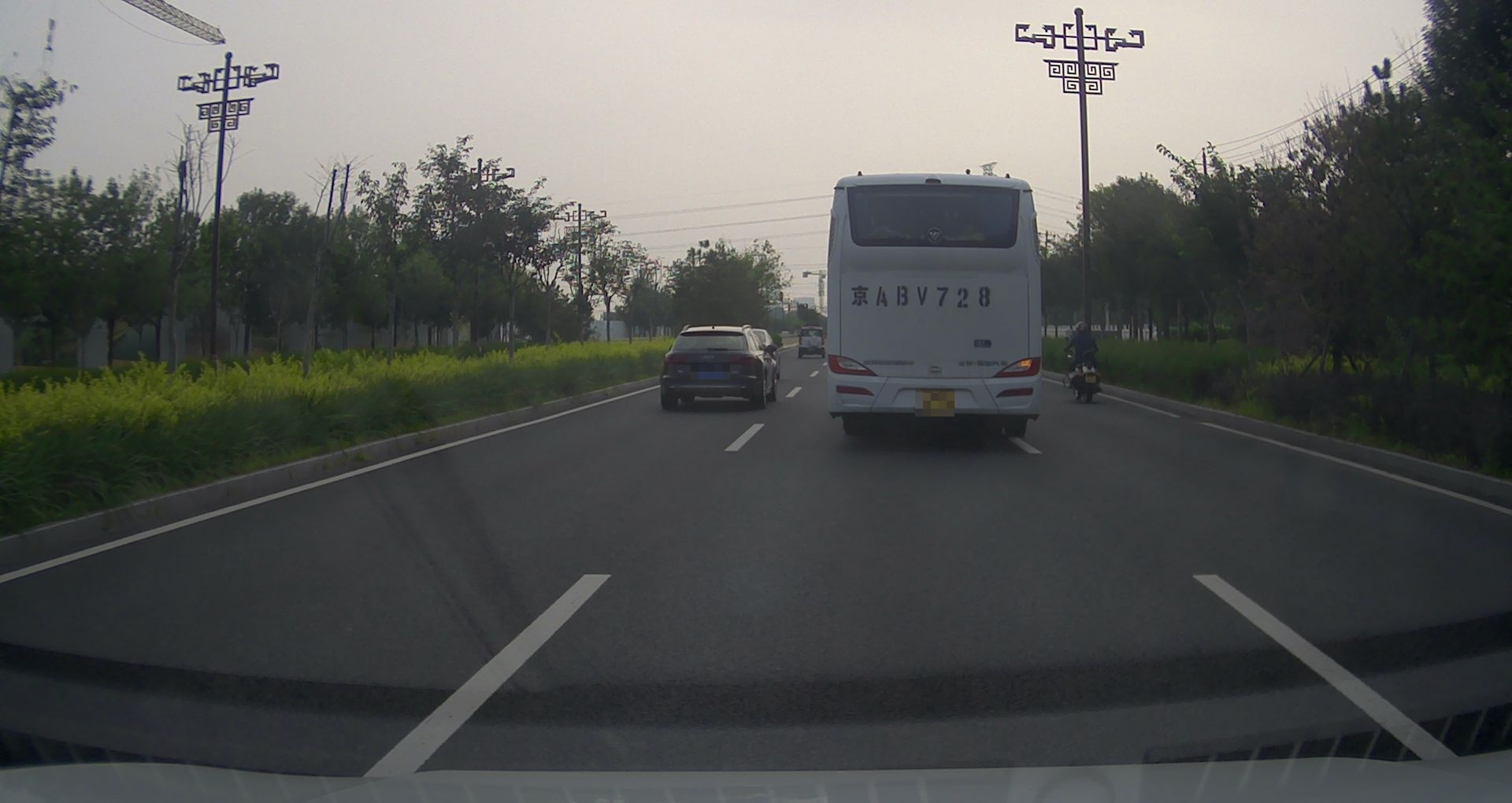

Supplement: S1 Dataset — All collected images were collected together, labeled and summarized one by one, and resulting classification results were roughly classified into three major categories: dry, wet and snowy. (ZIP) [file pone.0310858.s001.zip › weather1_data/dry_road/1628123766582.jpg]

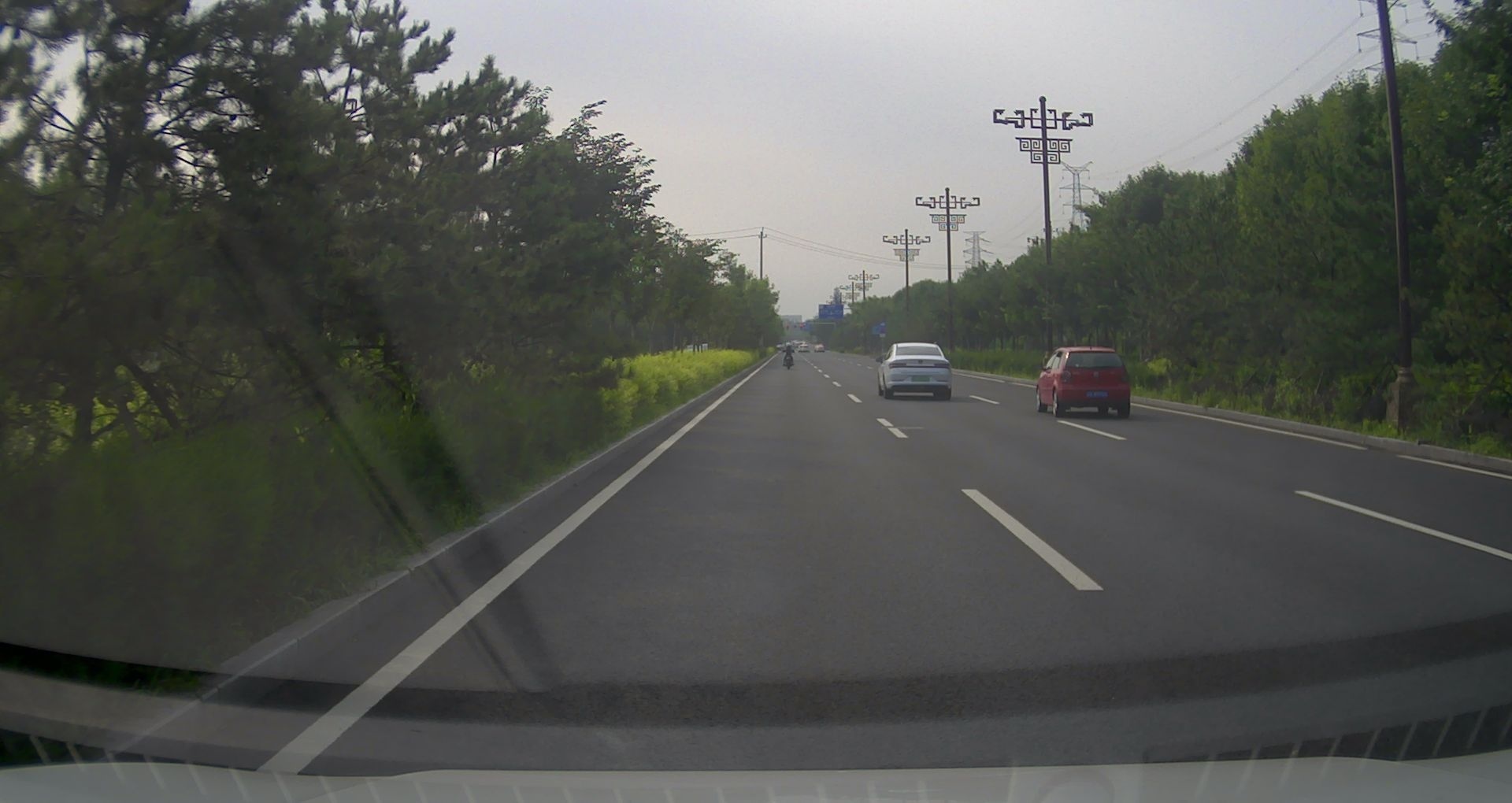

Supplement: S1 Dataset — All collected images were collected together, labeled and summarized one by one, and resulting classification results were roughly classified into three major categories: dry, wet and snowy. (ZIP) [file pone.0310858.s001.zip › weather1_data/dry_road/1628123881064.jpg]

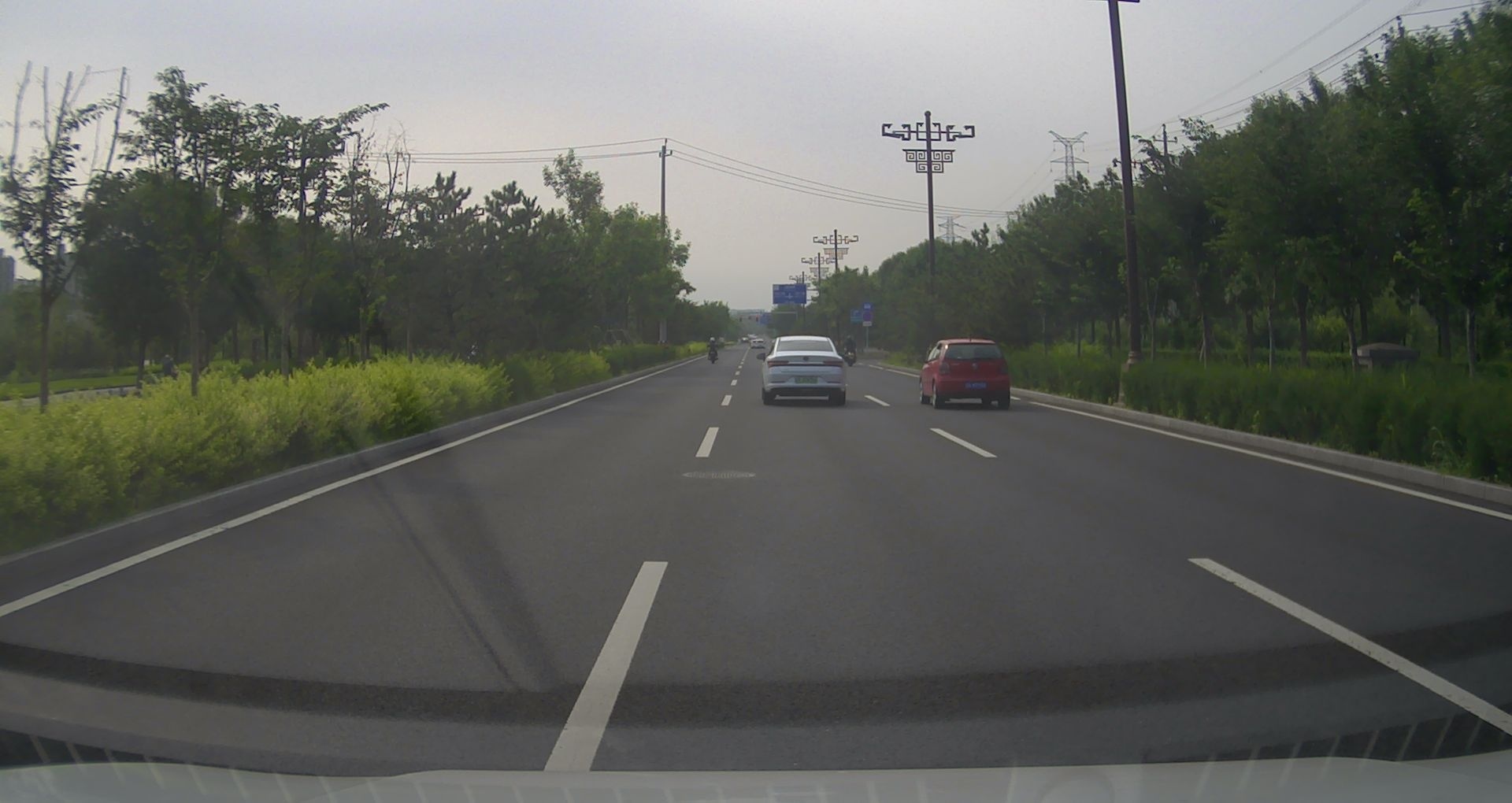

Supplement: S1 Dataset — All collected images were collected together, labeled and summarized one by one, and resulting classification results were roughly classified into three major categories: dry, wet and snowy. (ZIP) [file pone.0310858.s001.zip › weather1_data/dry_road/1628123884568.jpg]

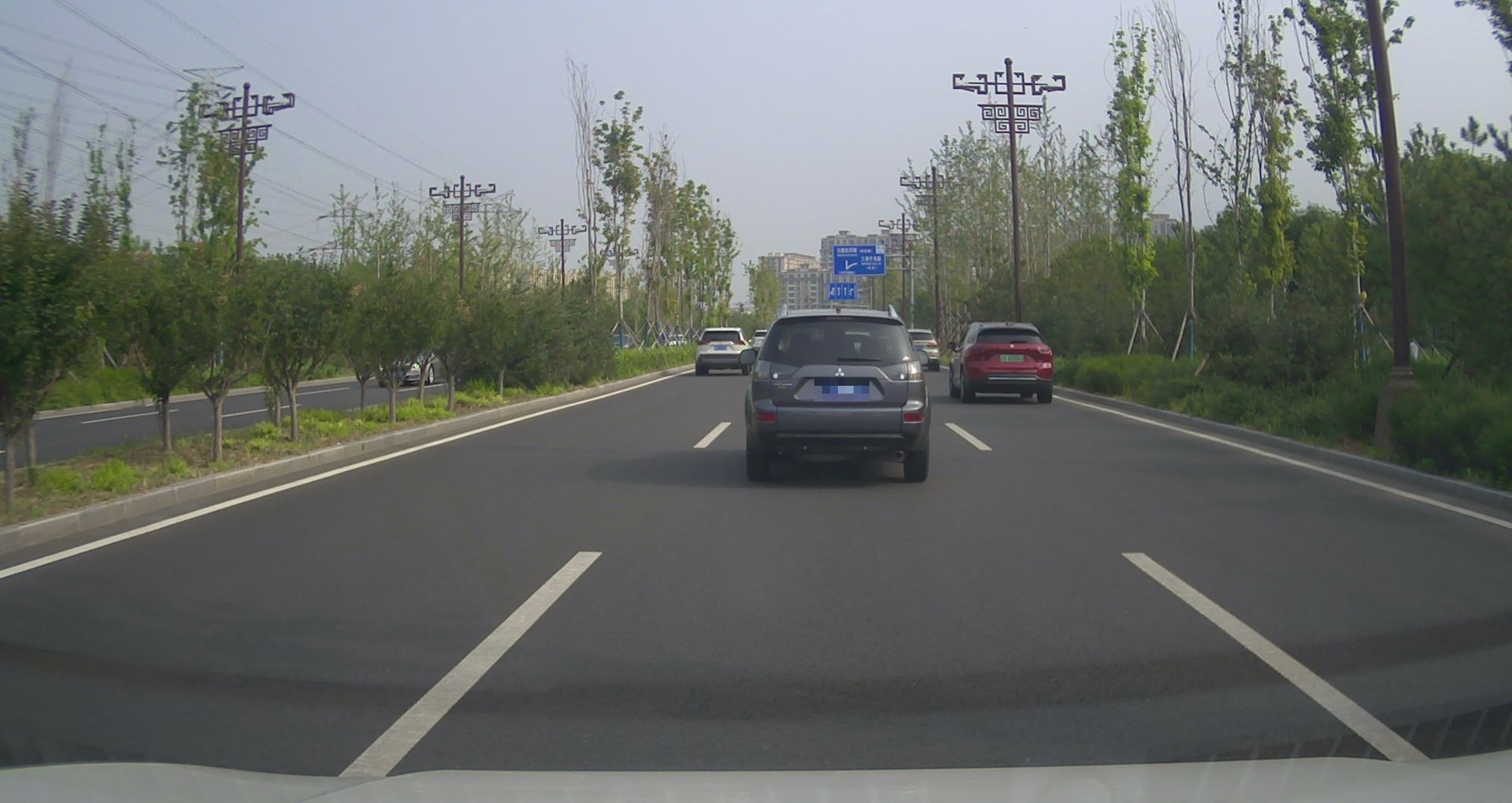

Supplement: S1 Dataset — All collected images were collected together, labeled and summarized one by one, and resulting classification results were roughly classified into three major categories: dry, wet and snowy. (ZIP) [file pone.0310858.s001.zip › weather1_data/dry_road/1628124276814.jpg]

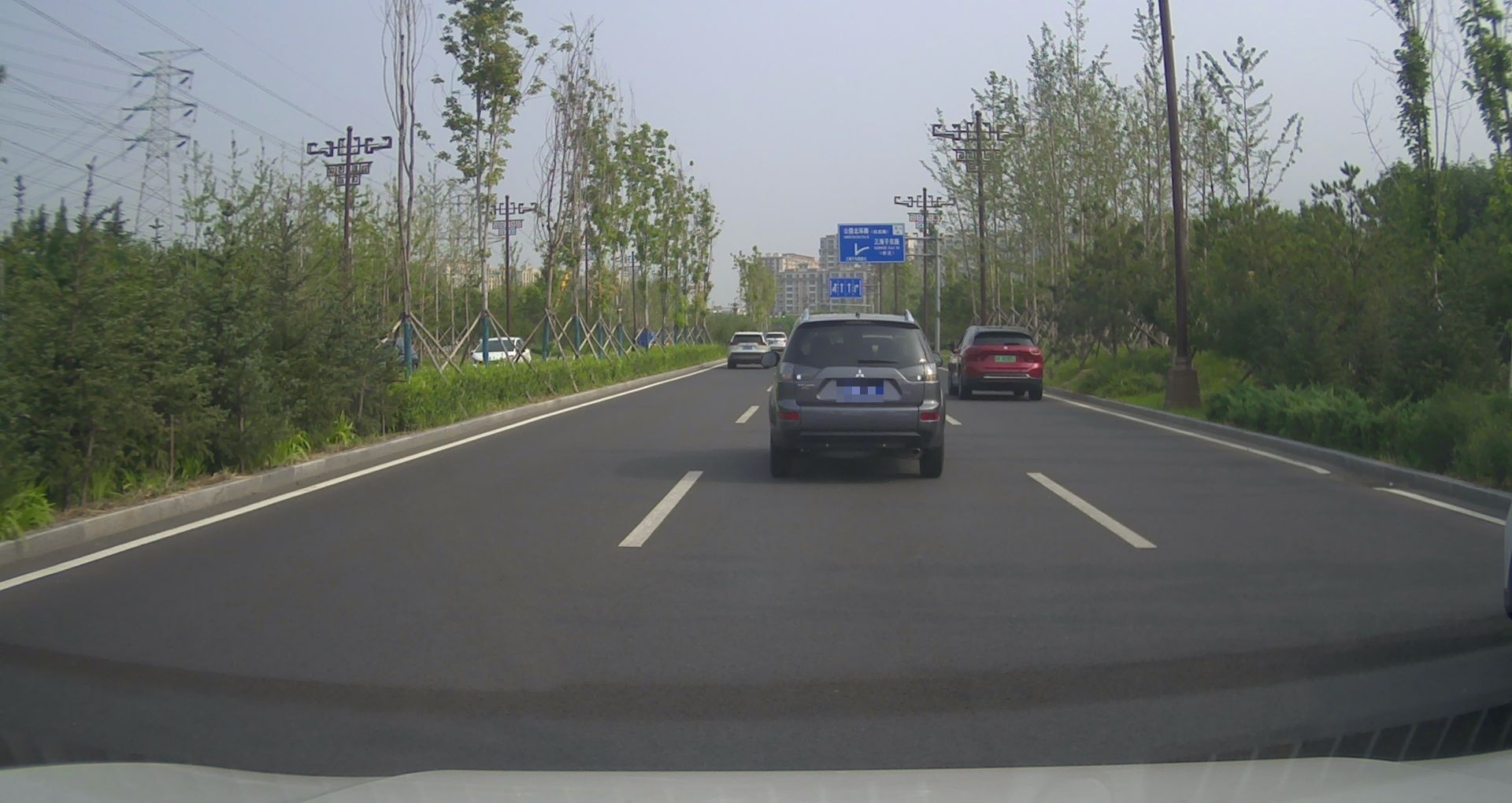

Supplement: S1 Dataset — All collected images were collected together, labeled and summarized one by one, and resulting classification results were roughly classified into three major categories: dry, wet and snowy. (ZIP) [file pone.0310858.s001.zip › weather1_data/dry_road/1628124278917.jpg]

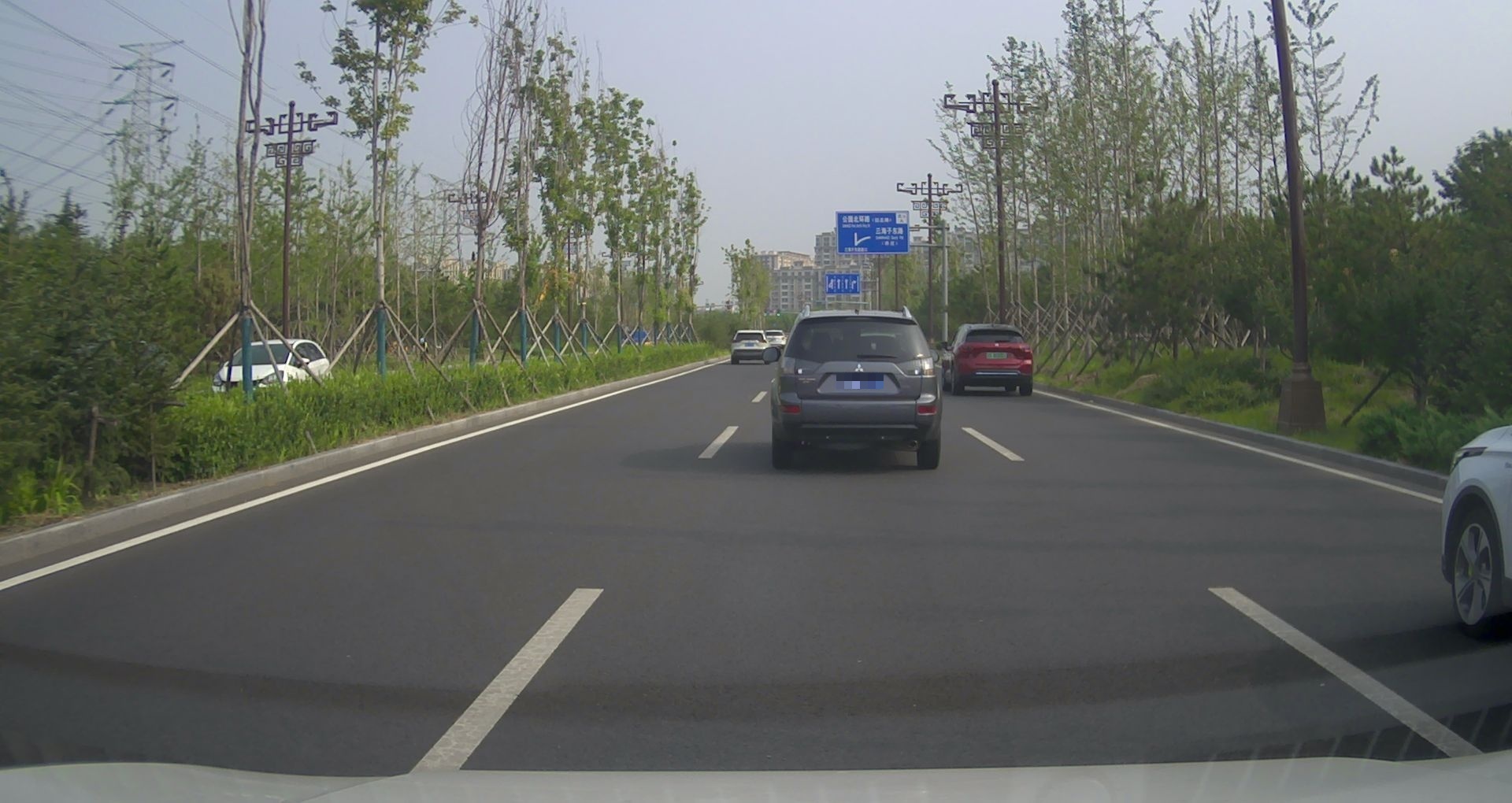

Supplement: S1 Dataset — All collected images were collected together, labeled and summarized one by one, and resulting classification results were roughly classified into three major categories: dry, wet and snowy. (ZIP) [file pone.0310858.s001.zip › weather1_data/dry_road/1628124279599.jpg]

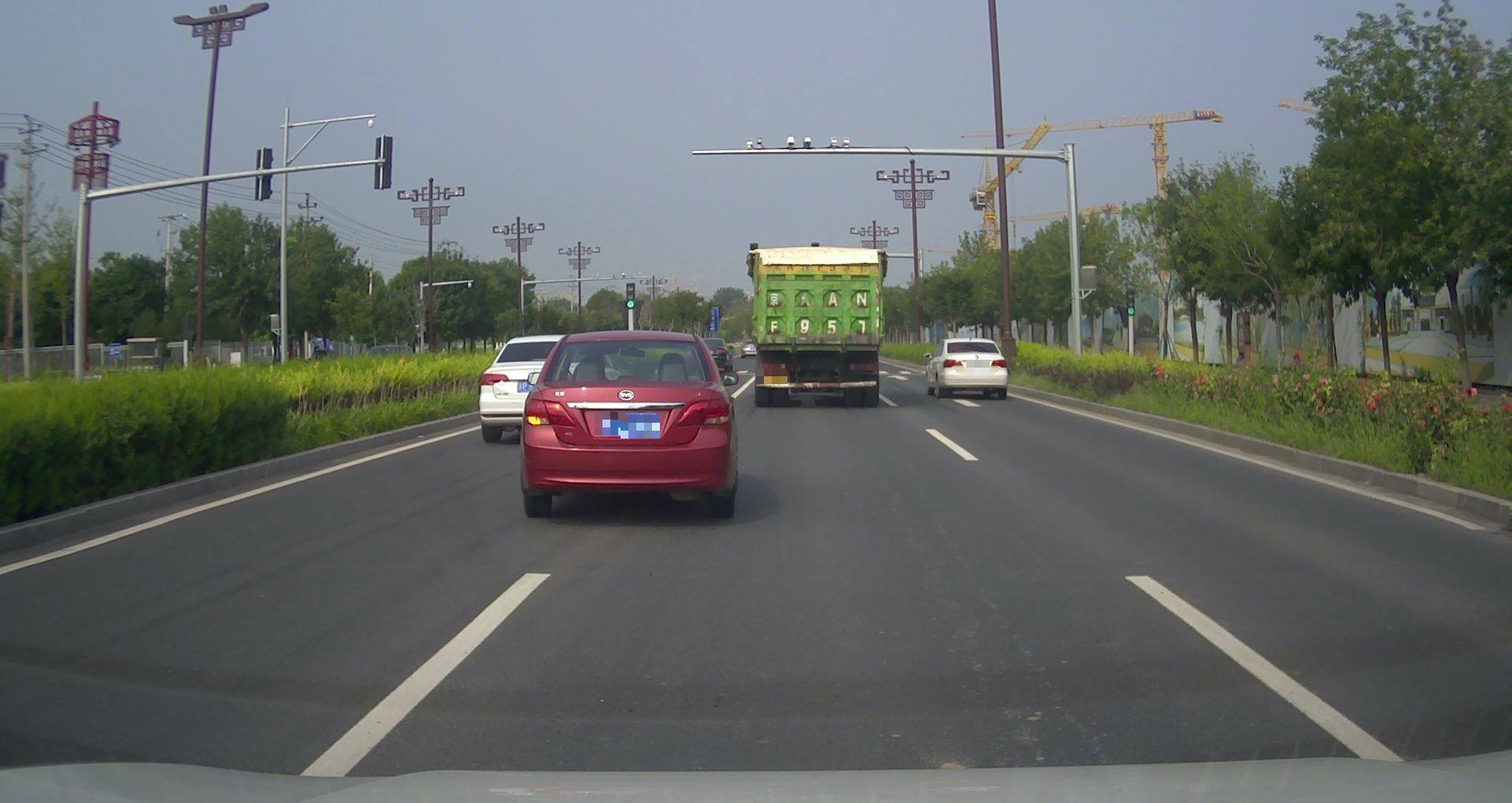

Supplement: S1 Dataset — All collected images were collected together, labeled and summarized one by one, and resulting classification results were roughly classified into three major categories: dry, wet and snowy. (ZIP) [file pone.0310858.s001.zip › weather1_data/dry_road/1628124995994.jpg]

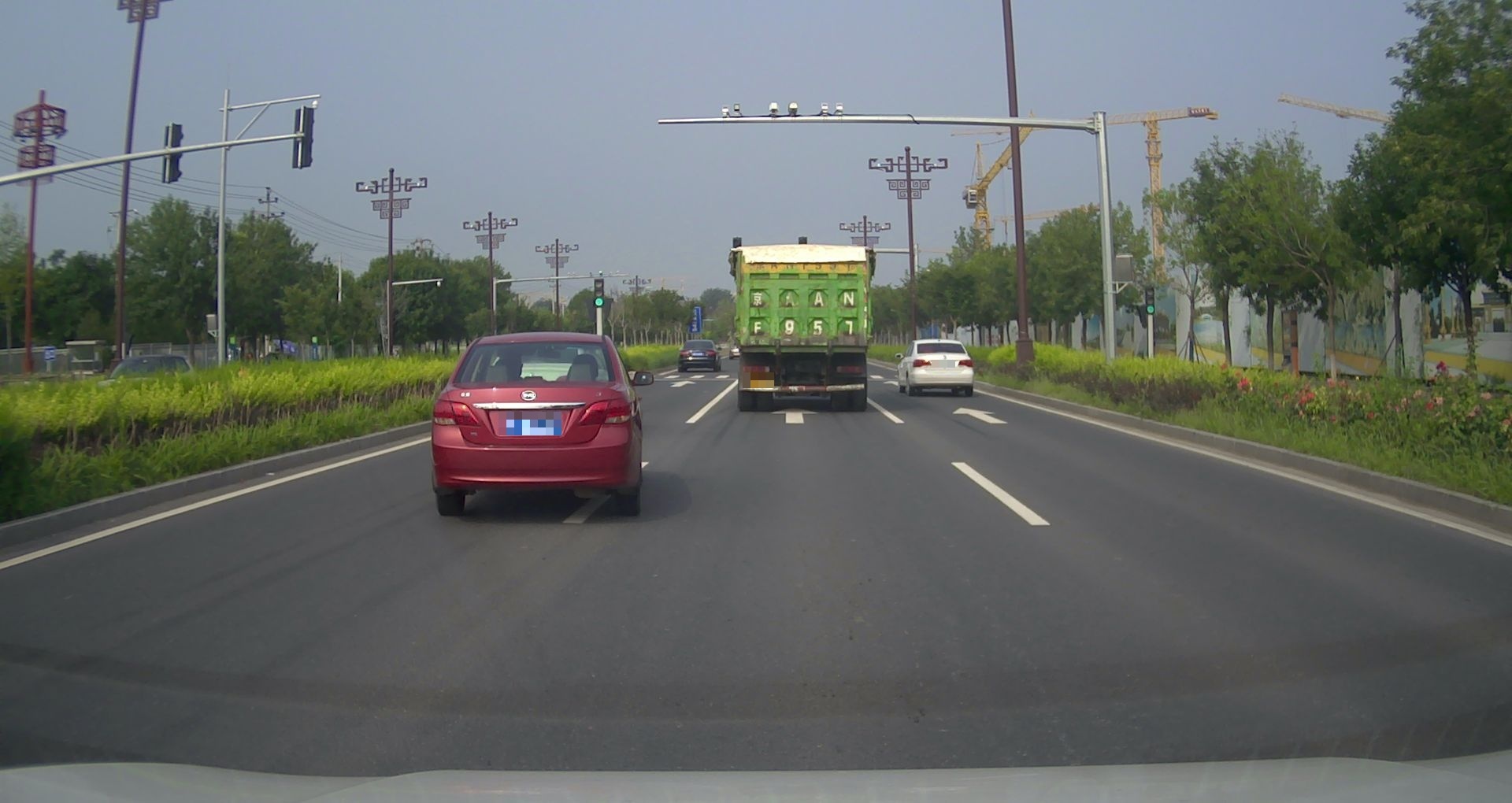

Supplement: S1 Dataset — All collected images were collected together, labeled and summarized one by one, and resulting classification results were roughly classified into three major categories: dry, wet and snowy. (ZIP) [file pone.0310858.s001.zip › weather1_data/dry_road/1628124996708.jpg]

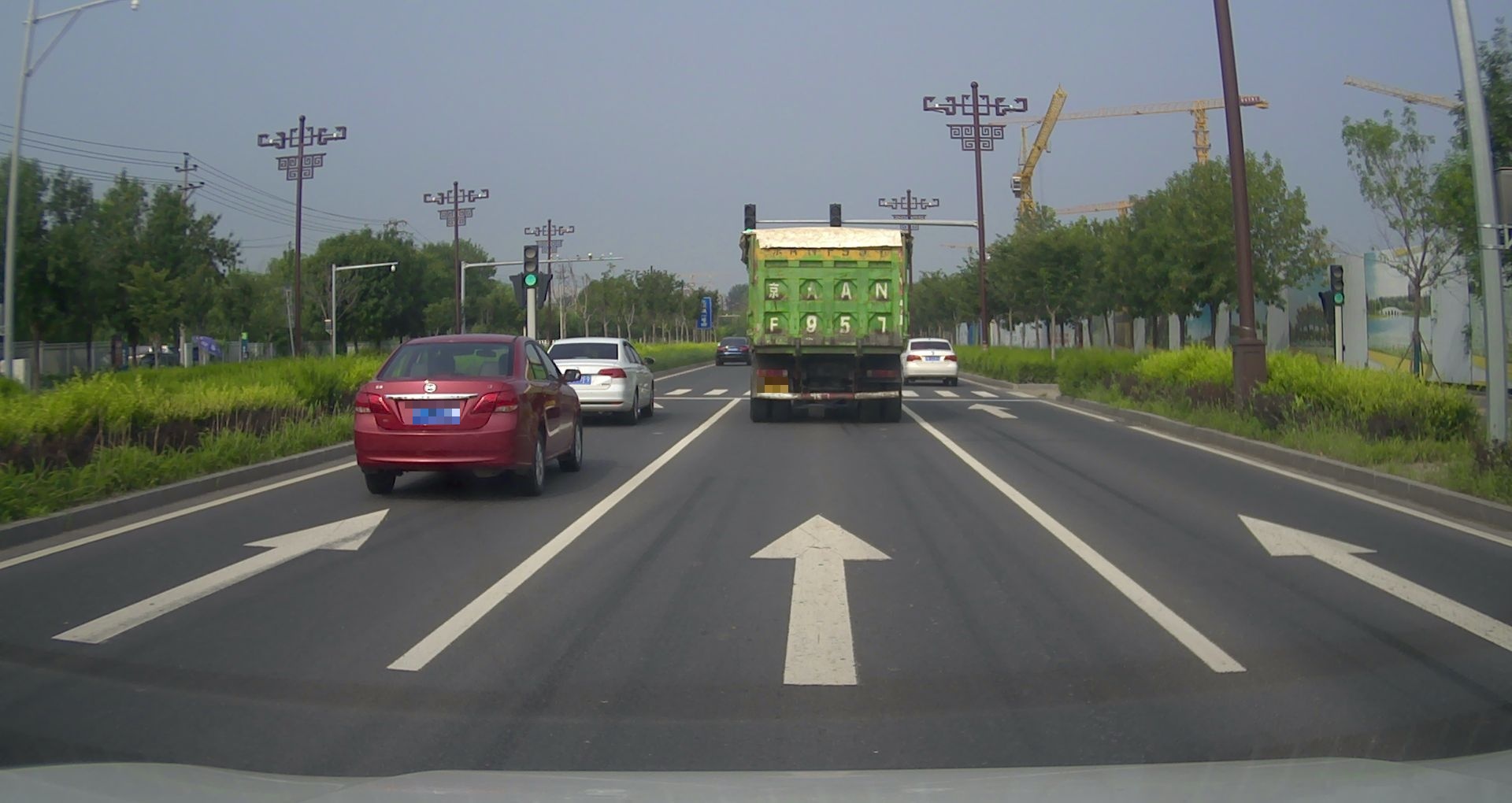

Supplement: S1 Dataset — All collected images were collected together, labeled and summarized one by one, and resulting classification results were roughly classified into three major categories: dry, wet and snowy. (ZIP) [file pone.0310858.s001.zip › weather1_data/dry_road/1628124998781.jpg]

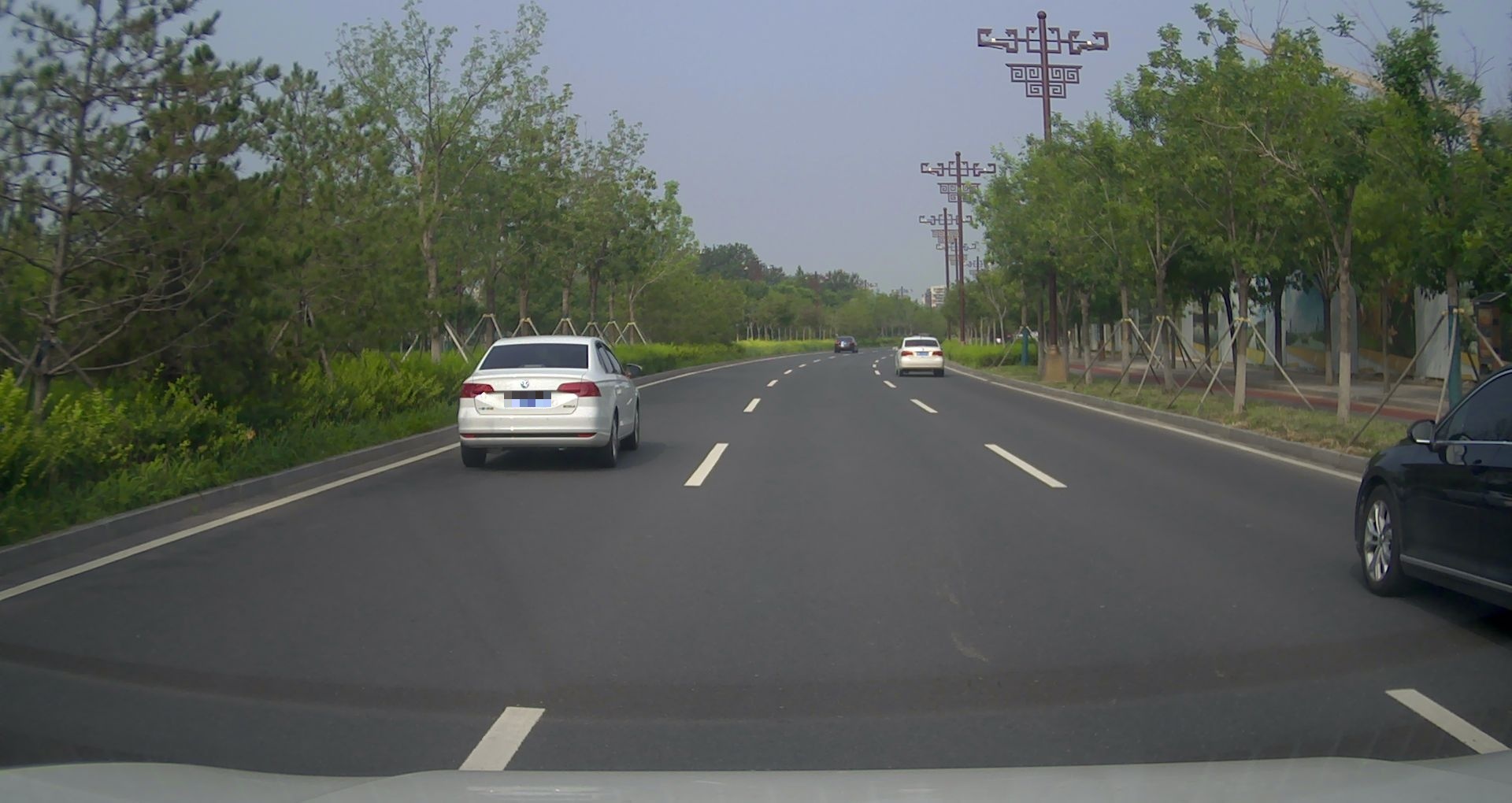

Supplement: S1 Dataset — All collected images were collected together, labeled and summarized one by one, and resulting classification results were roughly classified into three major categories: dry, wet and snowy. (ZIP) [file pone.0310858.s001.zip › weather1_data/dry_road/1628125030663.jpg]

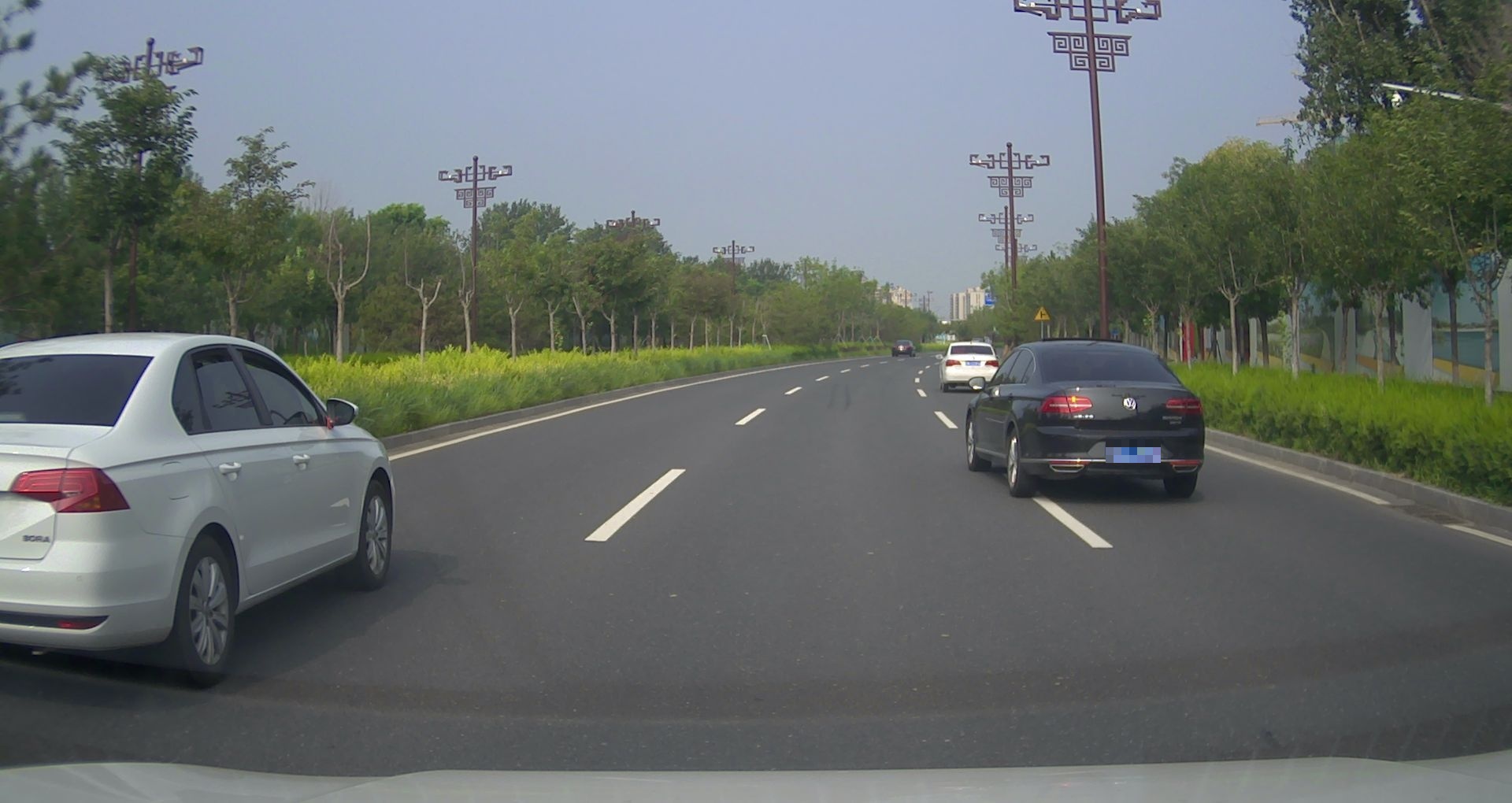

Supplement: S1 Dataset — All collected images were collected together, labeled and summarized one by one, and resulting classification results were roughly classified into three major categories: dry, wet and snowy. (ZIP) [file pone.0310858.s001.zip › weather1_data/dry_road/1628125035460.jpg]

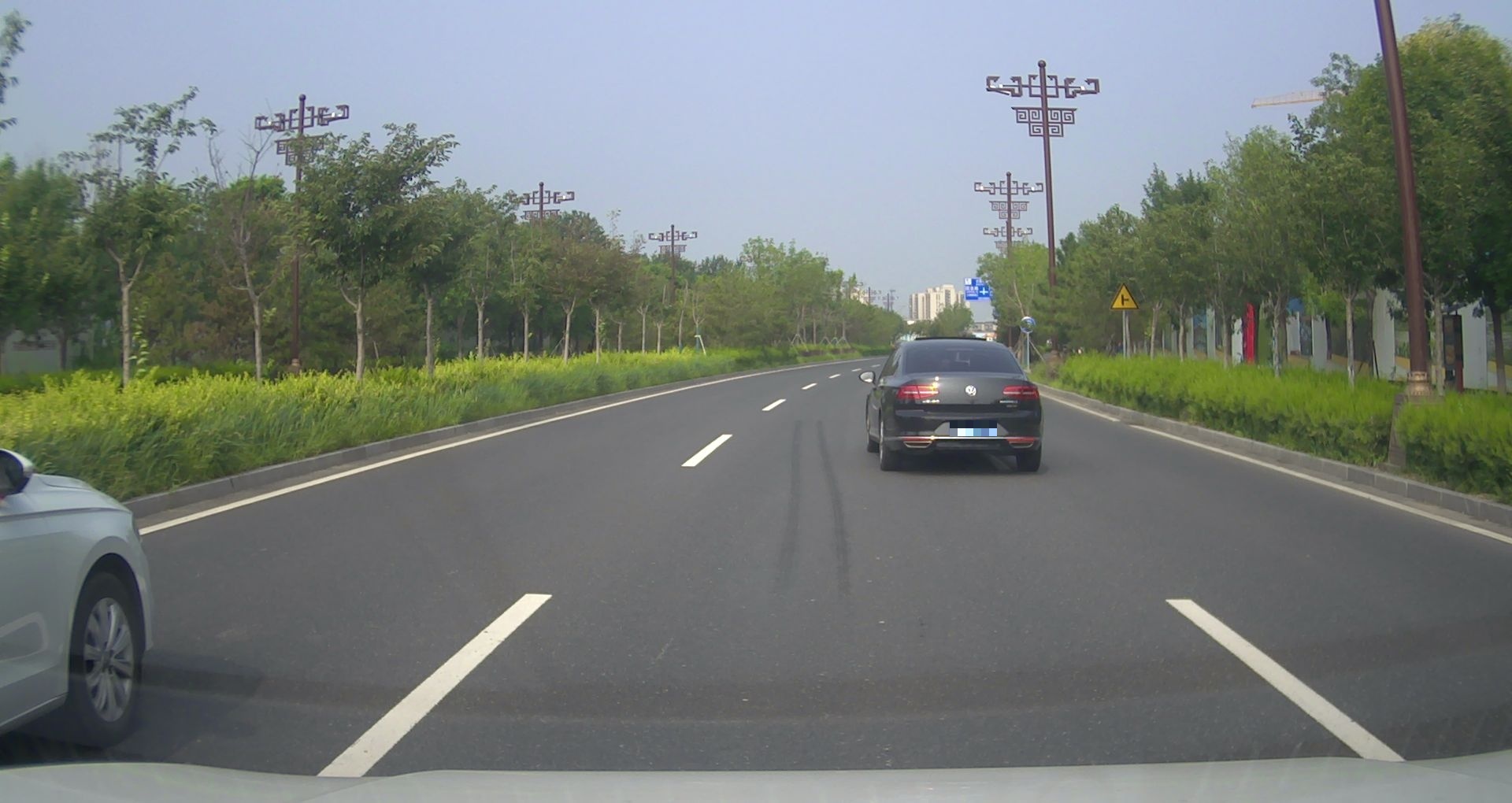

Supplement: S1 Dataset — All collected images were collected together, labeled and summarized one by one, and resulting classification results were roughly classified into three major categories: dry, wet and snowy. (ZIP) [file pone.0310858.s001.zip › weather1_data/dry_road/1628125036869.jpg]

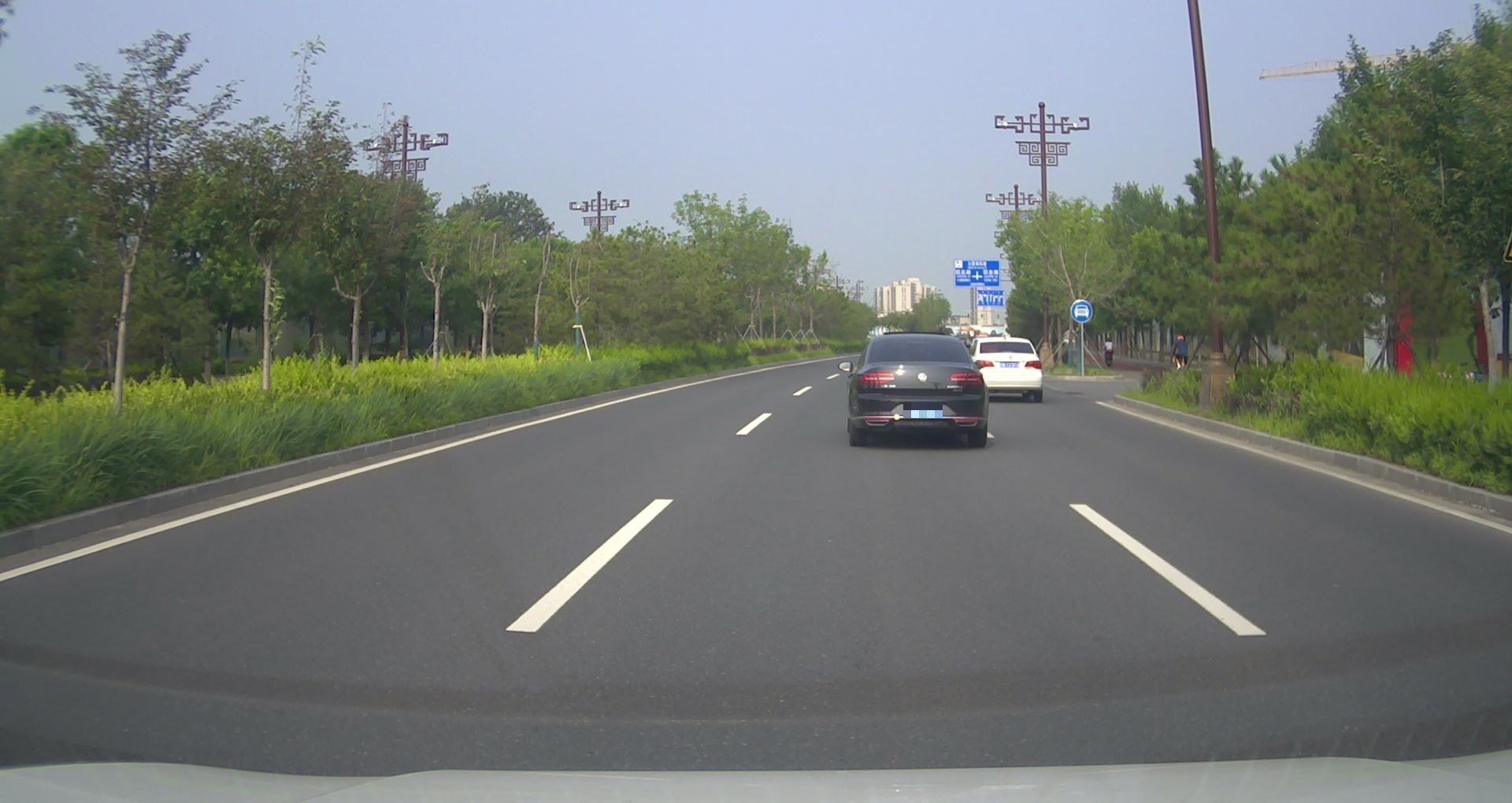

Supplement: S1 Dataset — All collected images were collected together, labeled and summarized one by one, and resulting classification results were roughly classified into three major categories: dry, wet and snowy. (ZIP) [file pone.0310858.s001.zip › weather1_data/dry_road/1628125038332.jpg]

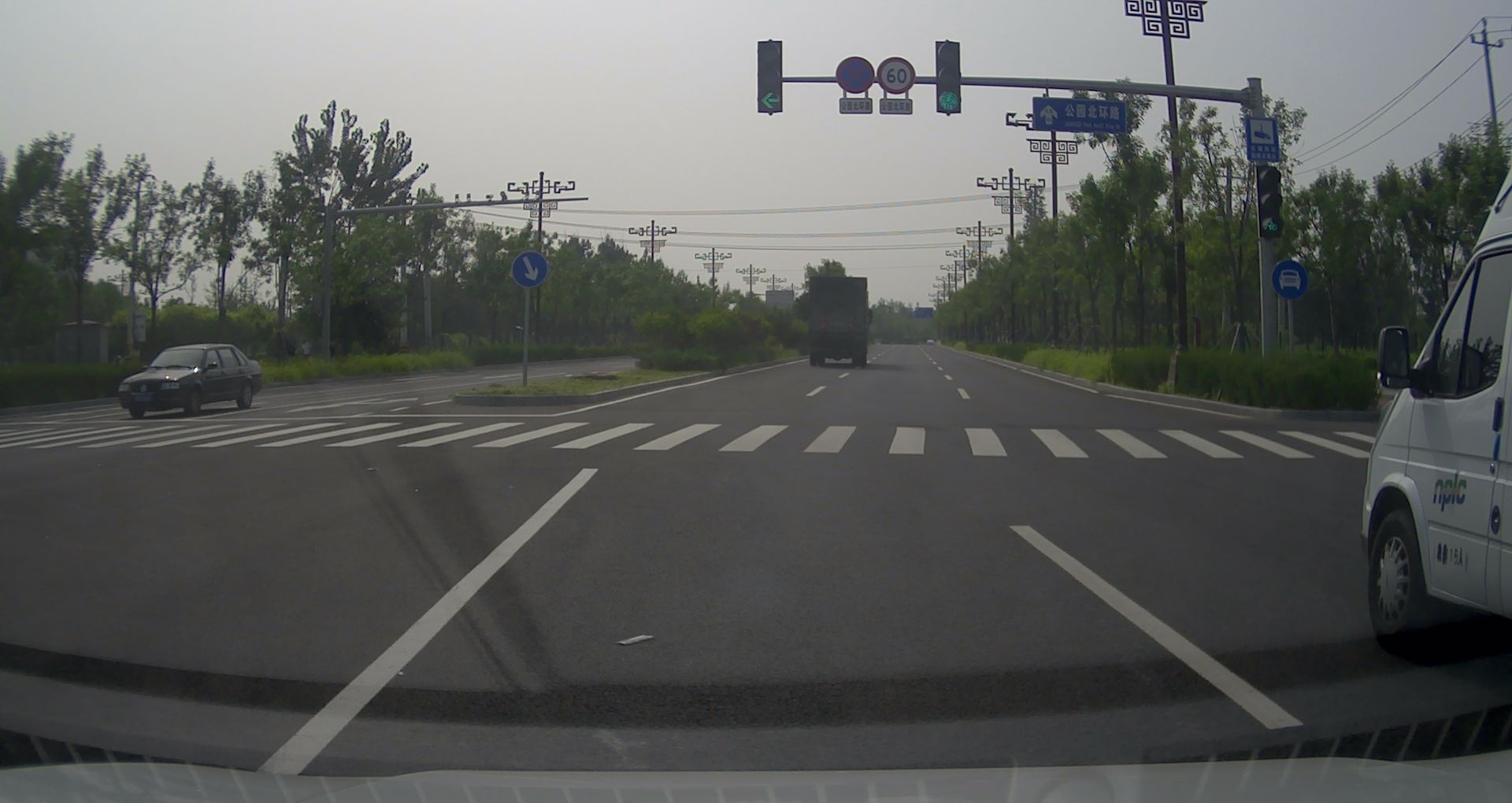

Supplement: S1 Dataset — All collected images were collected together, labeled and summarized one by one, and resulting classification results were roughly classified into three major categories: dry, wet and snowy. (ZIP) [file pone.0310858.s001.zip › weather1_data/dry_road/1628126084256.jpg]

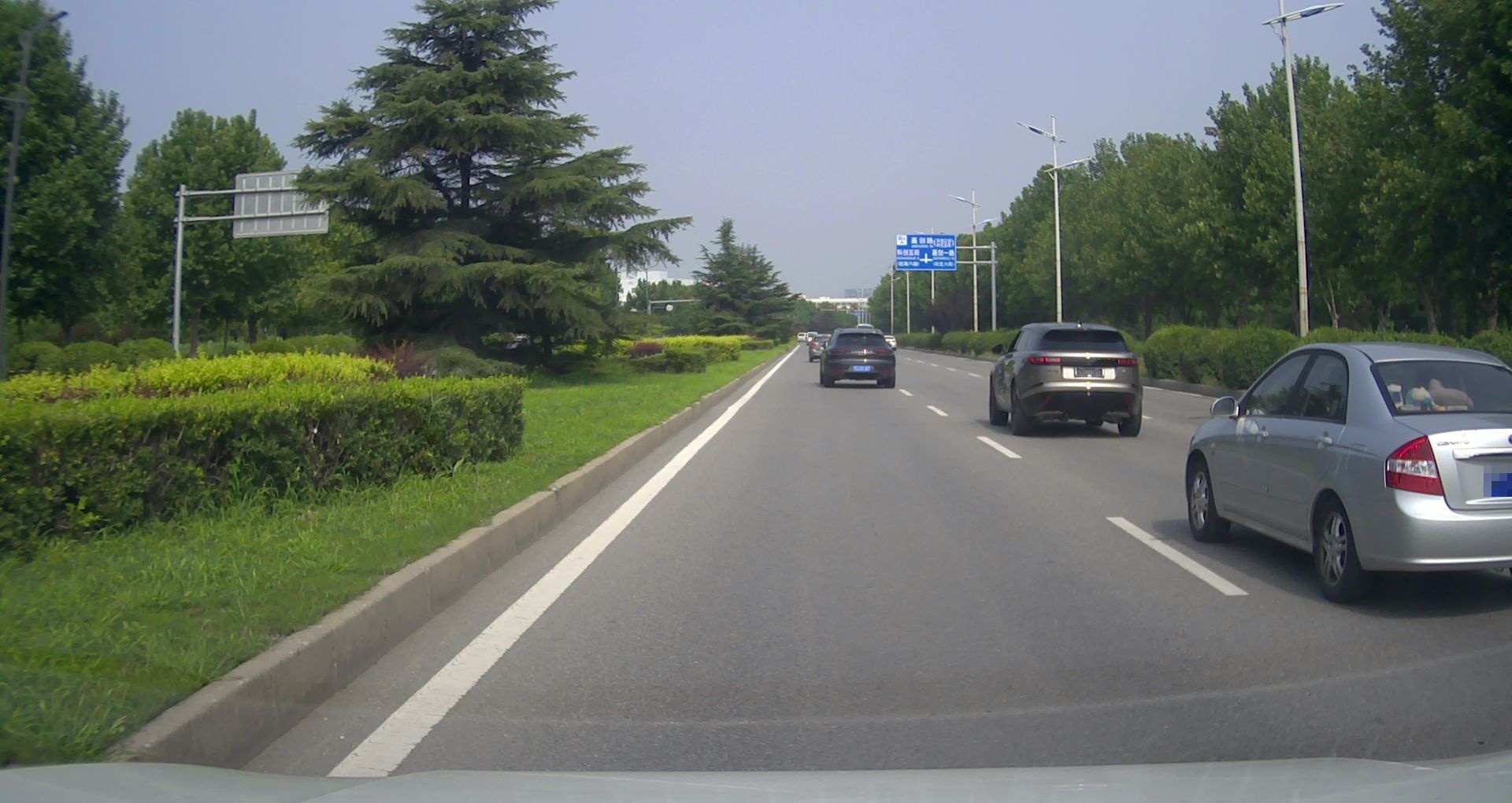

Supplement: S1 Dataset — All collected images were collected together, labeled and summarized one by one, and resulting classification results were roughly classified into three major categories: dry, wet and snowy. (ZIP) [file pone.0310858.s001.zip › weather1_data/dry_road/1628127572686.jpg]

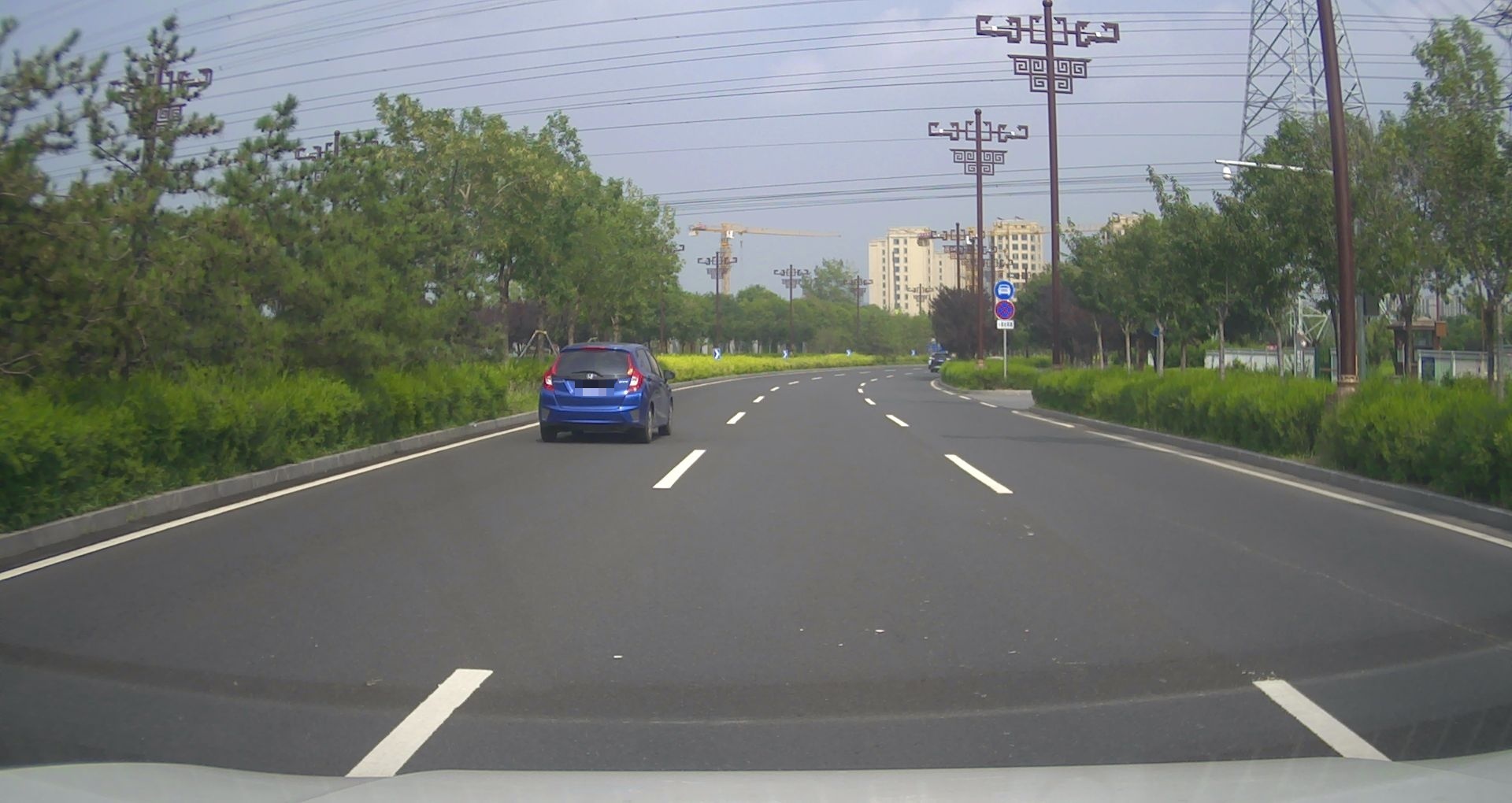

Supplement: S1 Dataset — All collected images were collected together, labeled and summarized one by one, and resulting classification results were roughly classified into three major categories: dry, wet and snowy. (ZIP) [file pone.0310858.s001.zip › weather1_data/dry_road/1628146662786.jpg]

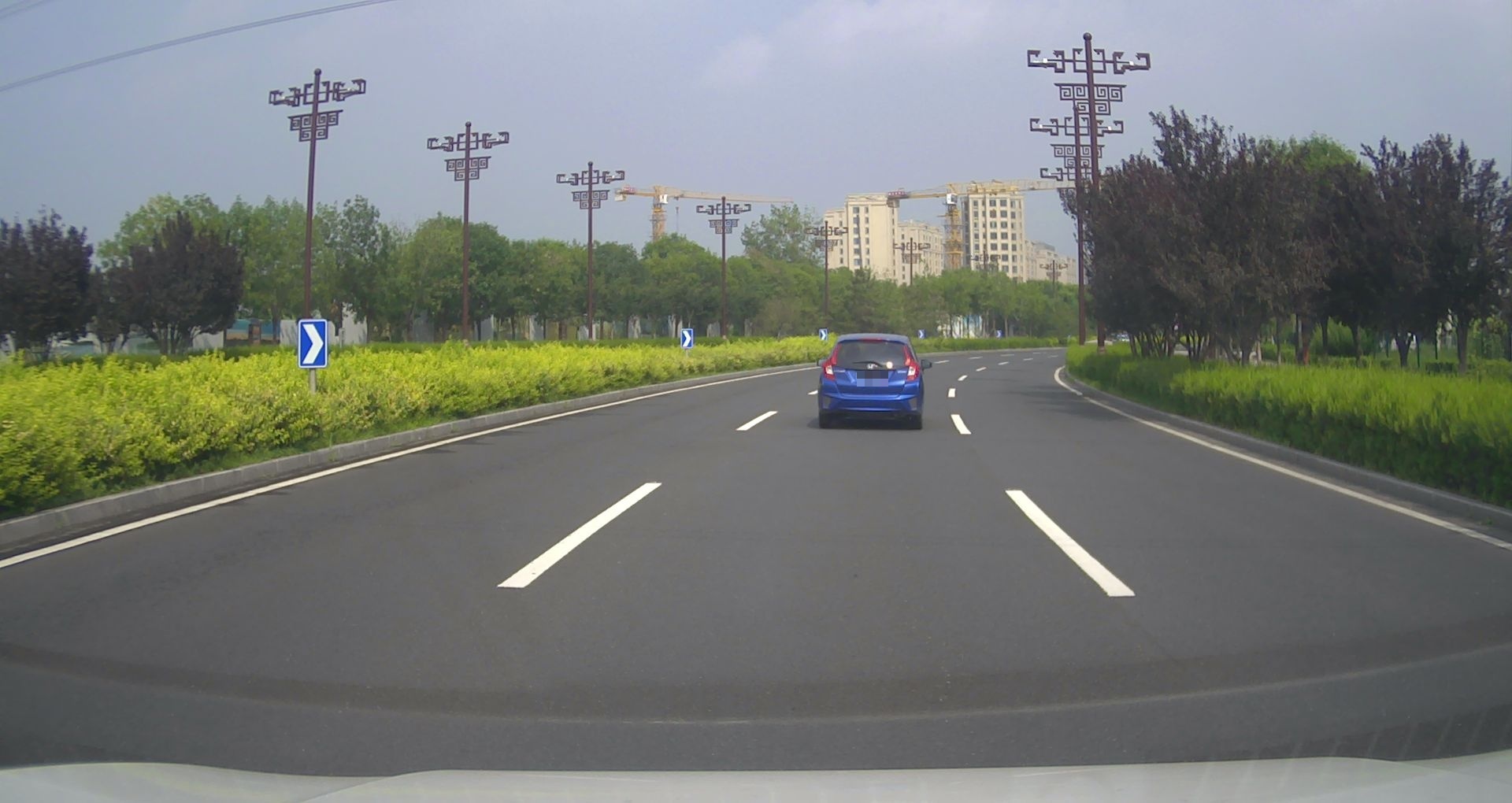

Supplement: S1 Dataset — All collected images were collected together, labeled and summarized one by one, and resulting classification results were roughly classified into three major categories: dry, wet and snowy. (ZIP) [file pone.0310858.s001.zip › weather1_data/dry_road/1628146667527.jpg]

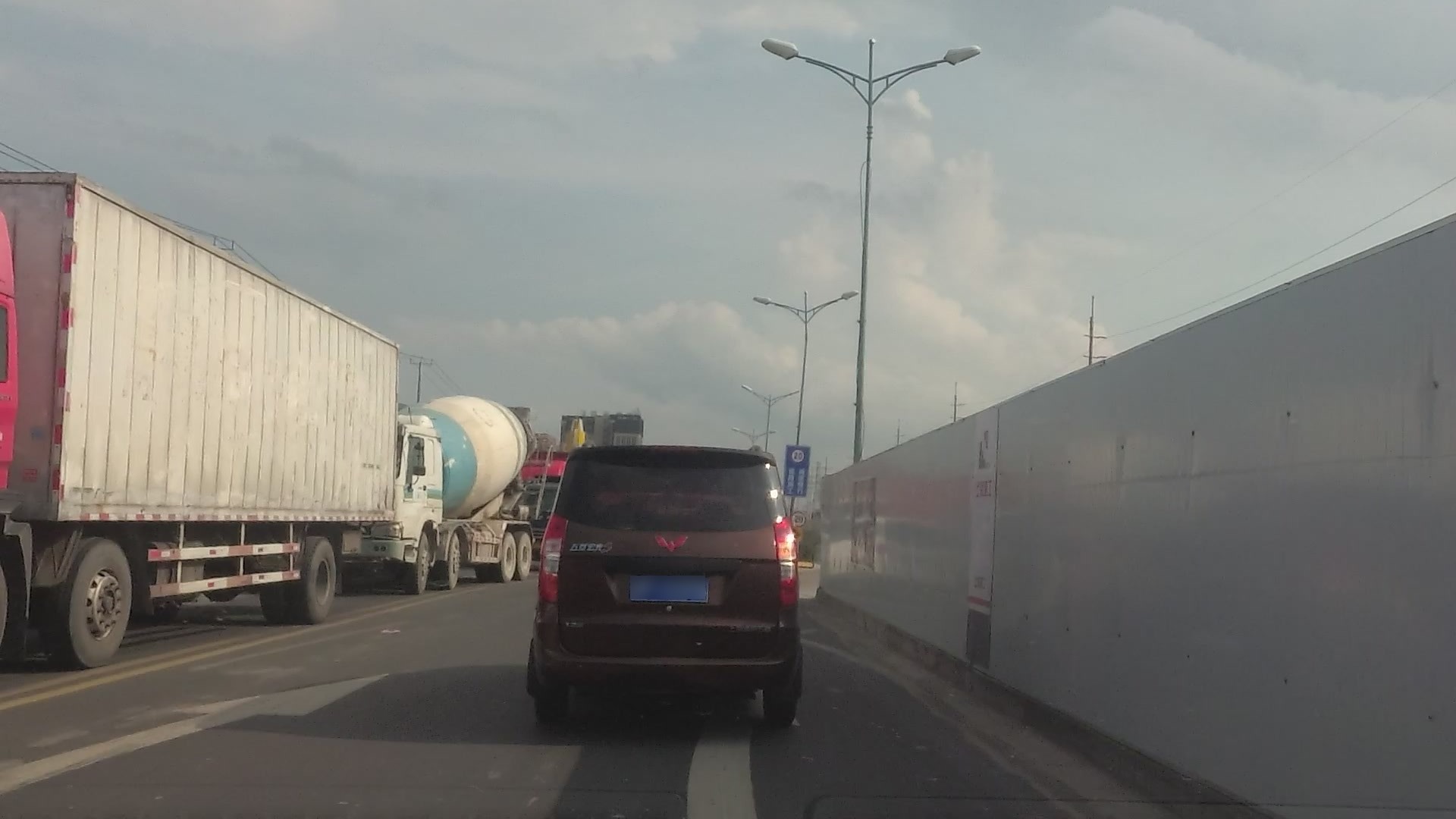

Supplement: S1 Dataset — All collected images were collected together, labeled and summarized one by one, and resulting classification results were roughly classified into three major categories: dry, wet and snowy. (ZIP) [file pone.0310858.s001.zip › weather1_data/dry_road/HT_TRAIN_000009_SH_000.jpg]

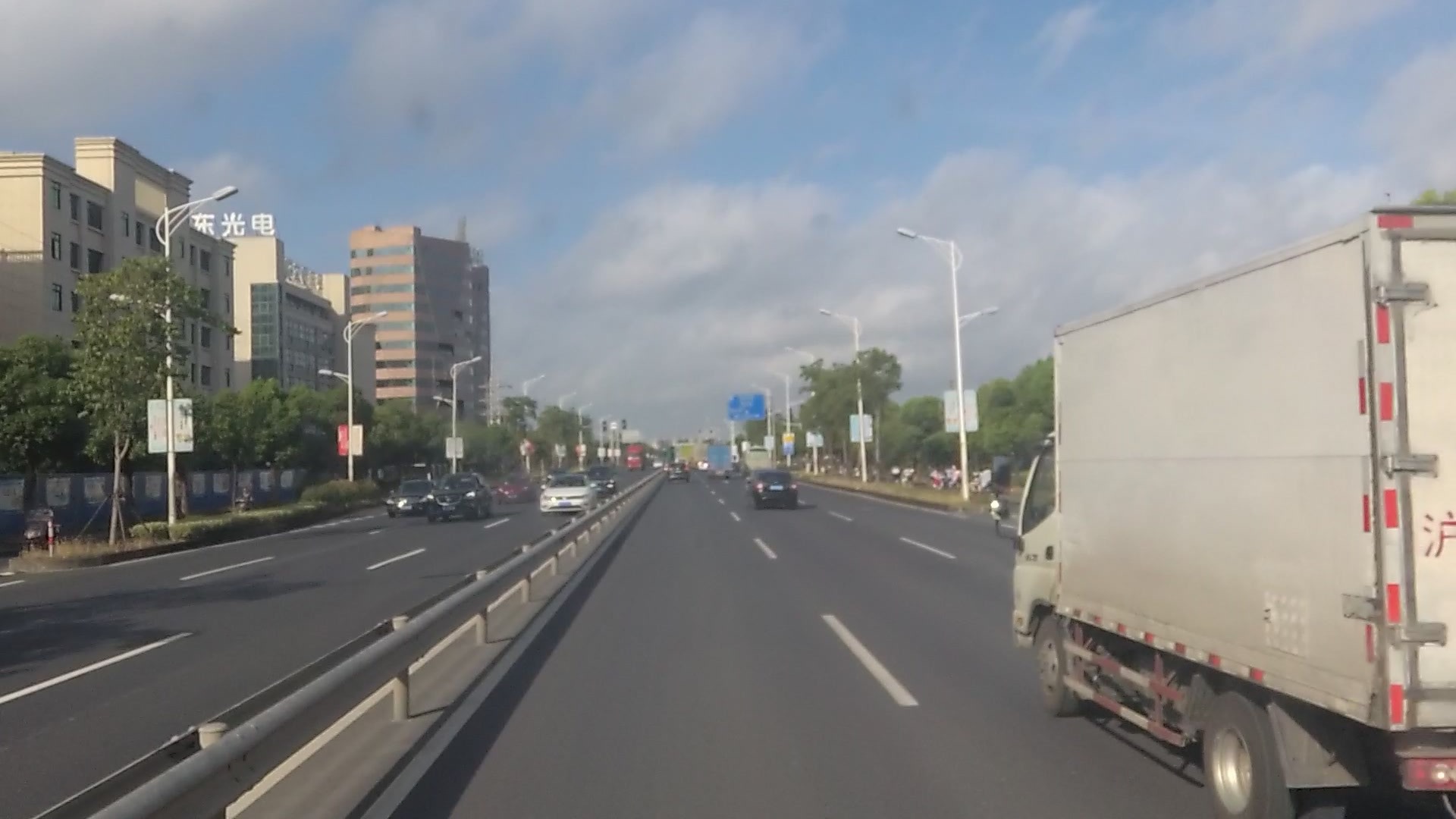

Supplement: S1 Dataset — All collected images were collected together, labeled and summarized one by one, and resulting classification results were roughly classified into three major categories: dry, wet and snowy. (ZIP) [file pone.0310858.s001.zip › weather1_data/dry_road/HT_TRAIN_000016_SH_000.jpg]

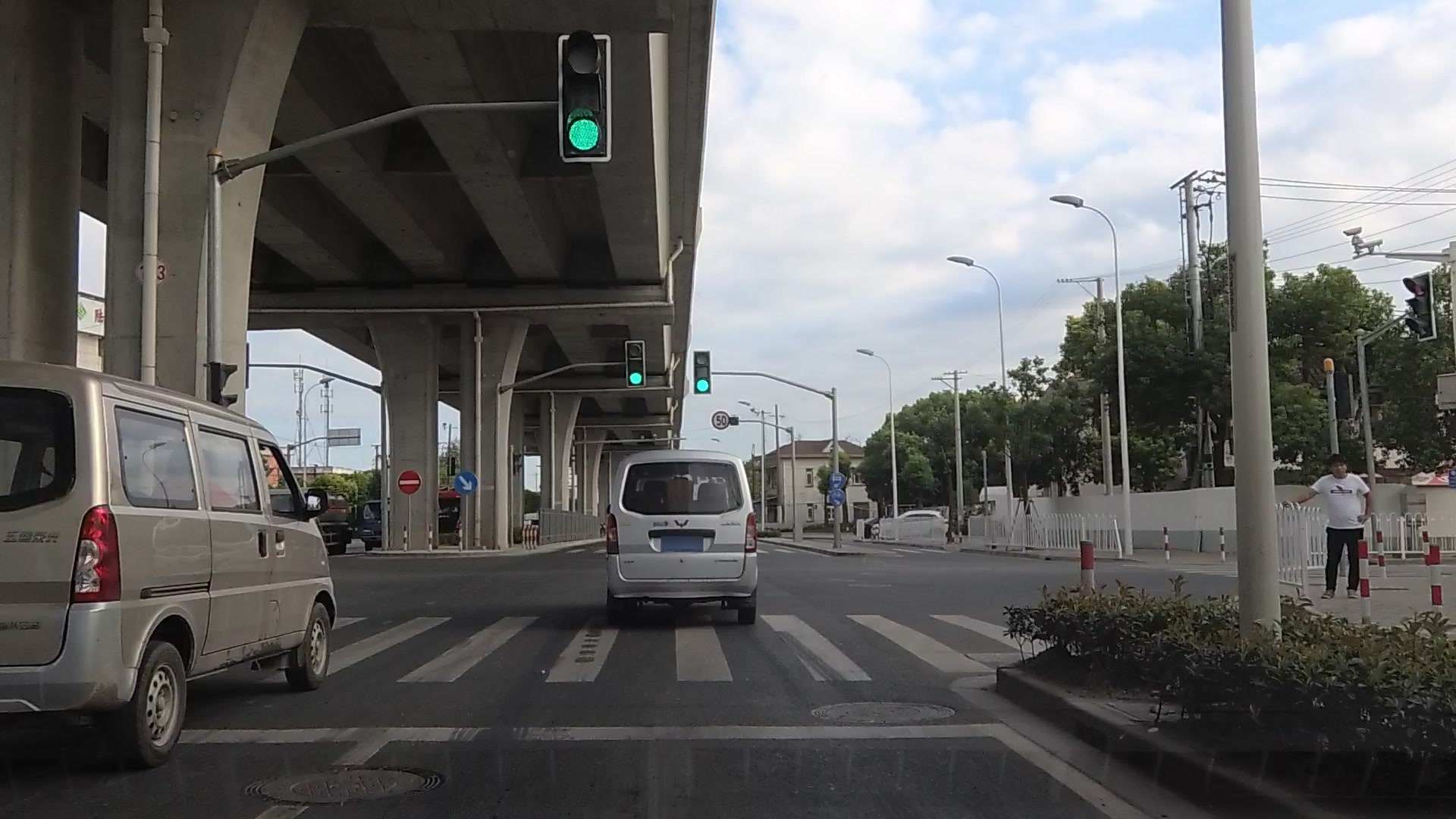

Supplement: S1 Dataset — All collected images were collected together, labeled and summarized one by one, and resulting classification results were roughly classified into three major categories: dry, wet and snowy. (ZIP) [file pone.0310858.s001.zip › weather1_data/dry_road/HT_TRAIN_000021_SH_000.jpg]

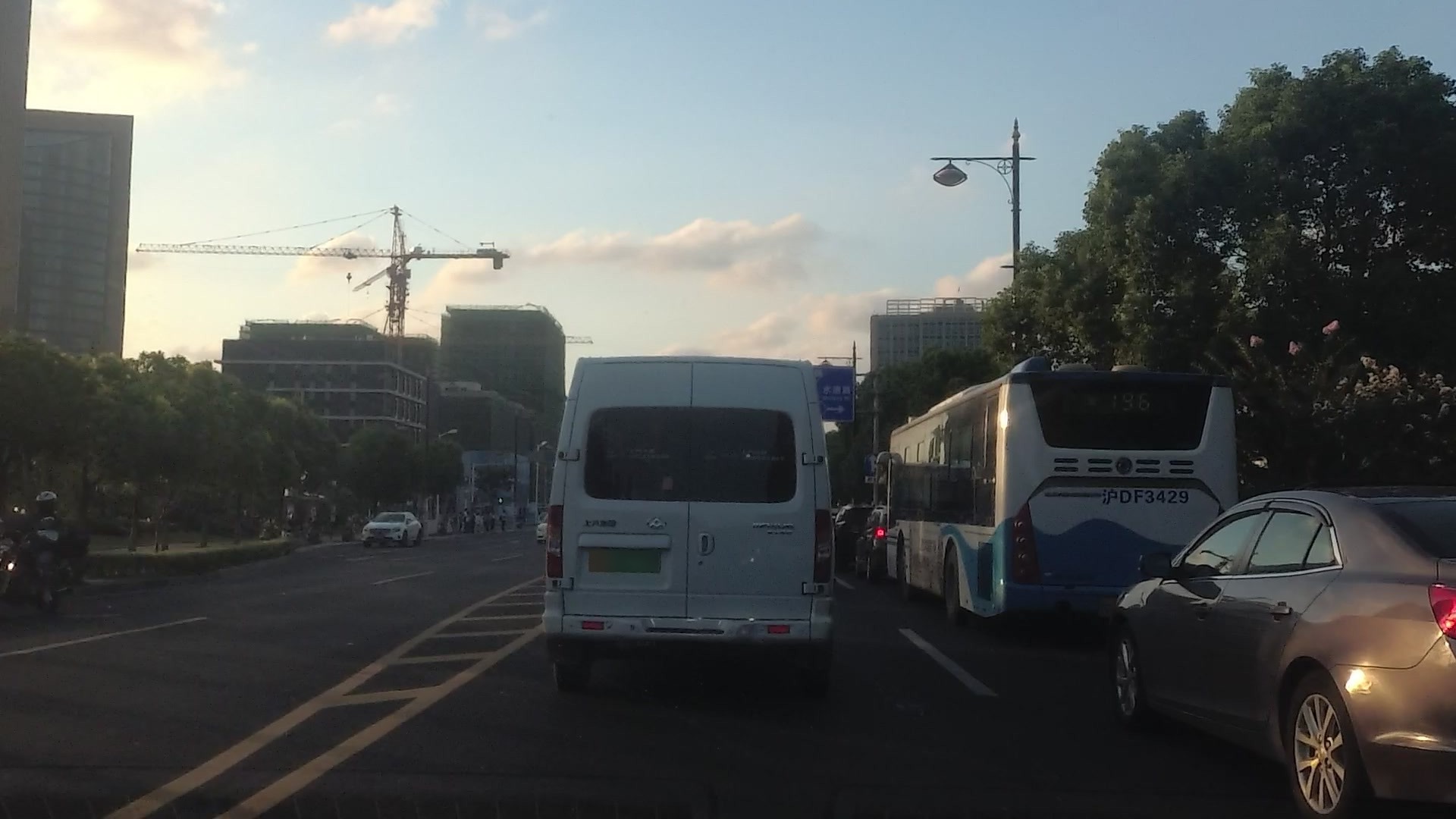

Supplement: S1 Dataset — All collected images were collected together, labeled and summarized one by one, and resulting classification results were roughly classified into three major categories: dry, wet and snowy. (ZIP) [file pone.0310858.s001.zip › weather1_data/dry_road/HT_TRAIN_000023_SH_000.jpg]

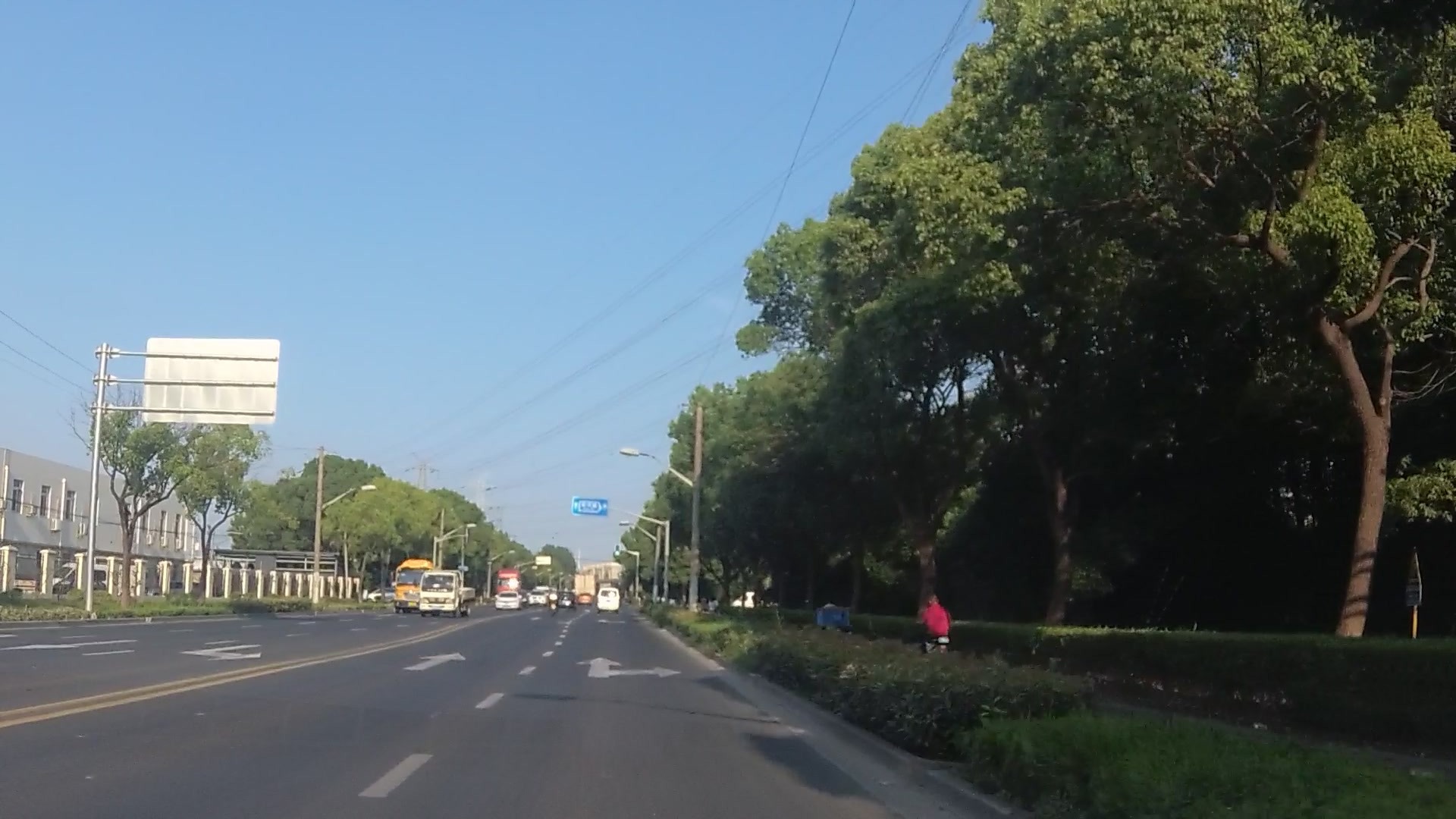

Supplement: S1 Dataset — All collected images were collected together, labeled and summarized one by one, and resulting classification results were roughly classified into three major categories: dry, wet and snowy. (ZIP) [file pone.0310858.s001.zip › weather1_data/dry_road/HT_TRAIN_000024_SH_000.jpg]

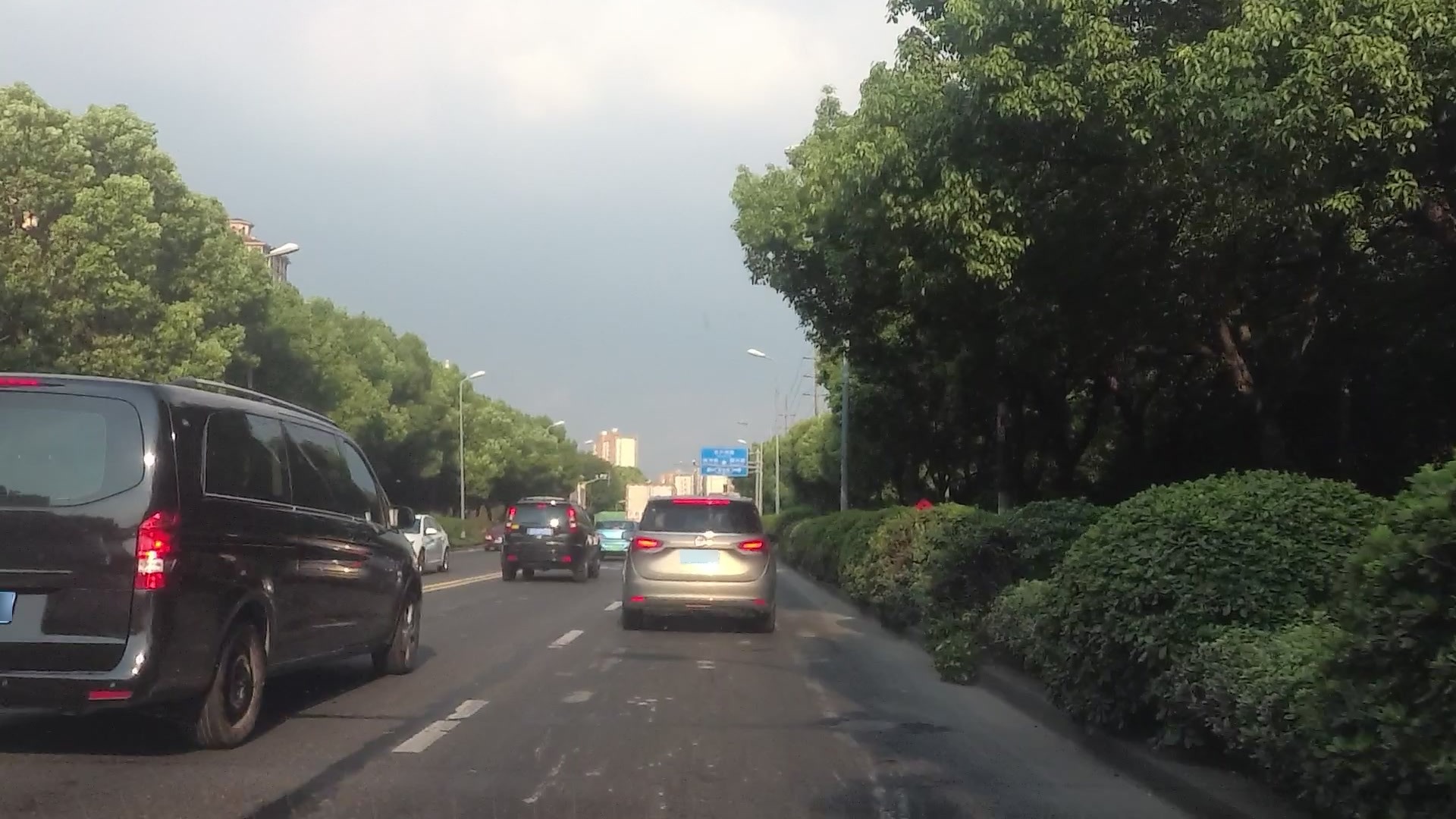

Supplement: S1 Dataset — All collected images were collected together, labeled and summarized one by one, and resulting classification results were roughly classified into three major categories: dry, wet and snowy. (ZIP) [file pone.0310858.s001.zip › weather1_data/dry_road/HT_TRAIN_000025_SH_000.jpg]

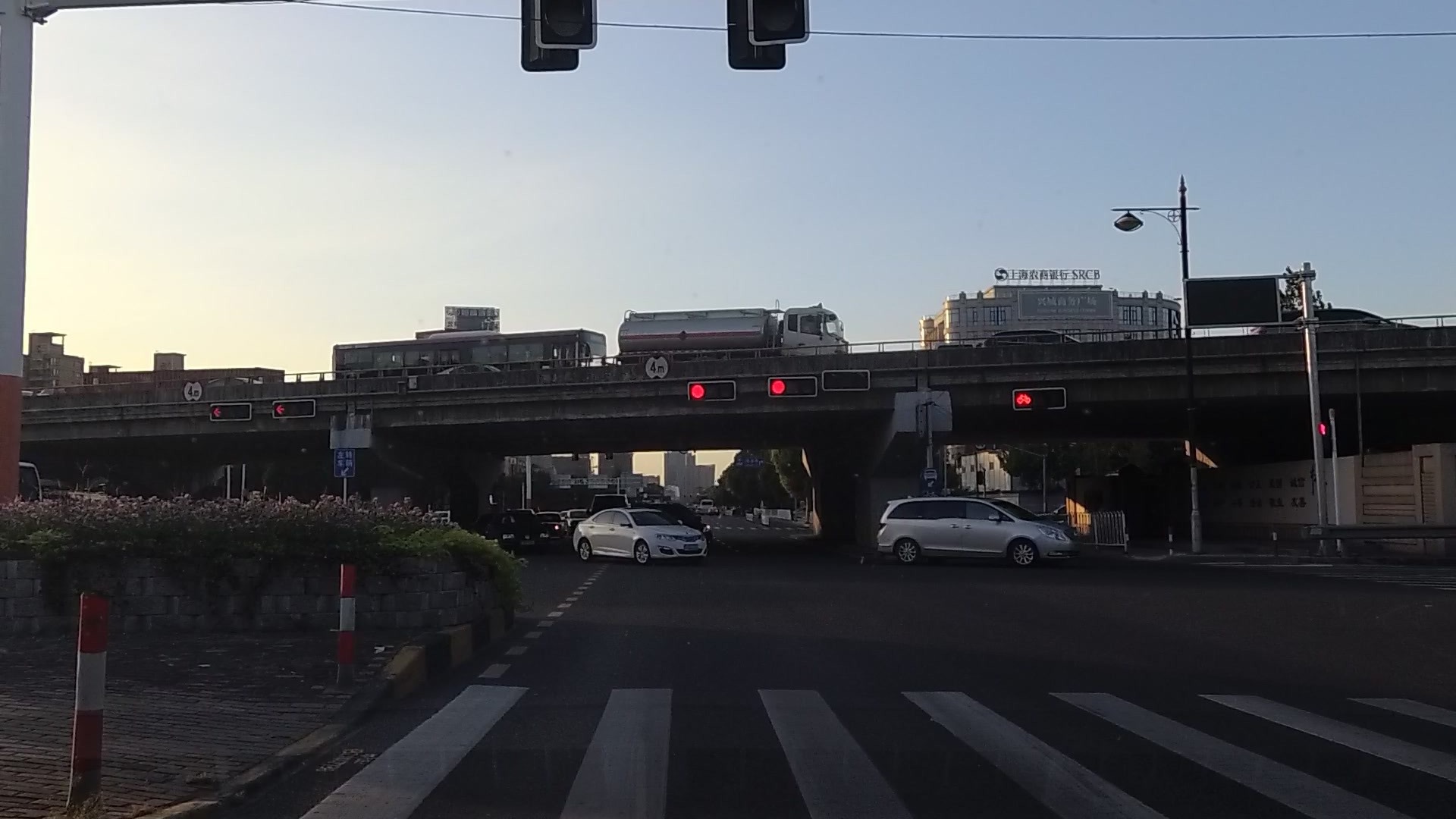

Supplement: S1 Dataset — All collected images were collected together, labeled and summarized one by one, and resulting classification results were roughly classified into three major categories: dry, wet and snowy. (ZIP) [file pone.0310858.s001.zip › weather1_data/dry_road/HT_TRAIN_000036_SH_000.jpg]

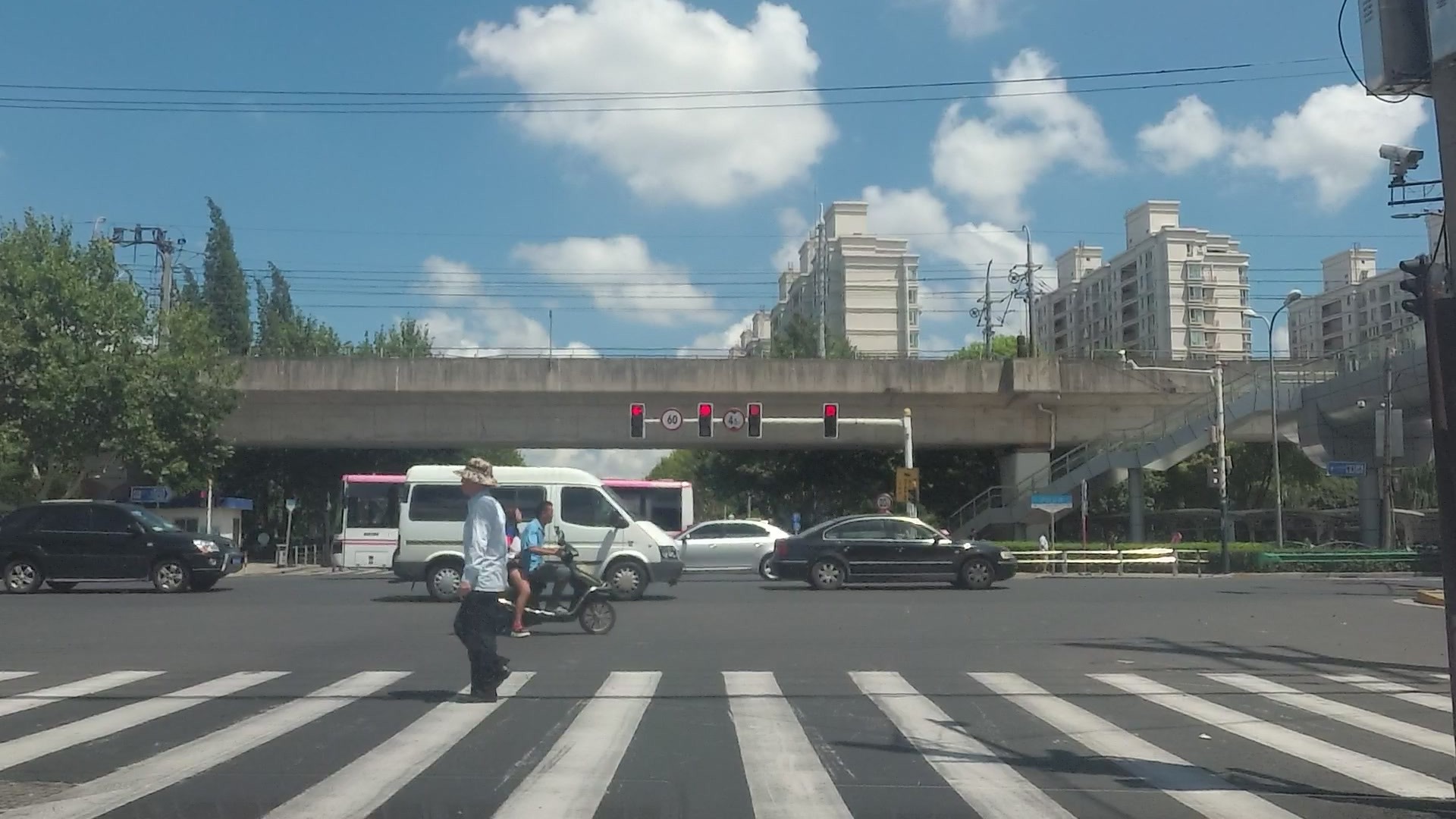

Supplement: S1 Dataset — All collected images were collected together, labeled and summarized one by one, and resulting classification results were roughly classified into three major categories: dry, wet and snowy. (ZIP) [file pone.0310858.s001.zip › weather1_data/dry_road/HT_TRAIN_000048_SH_000.jpg]

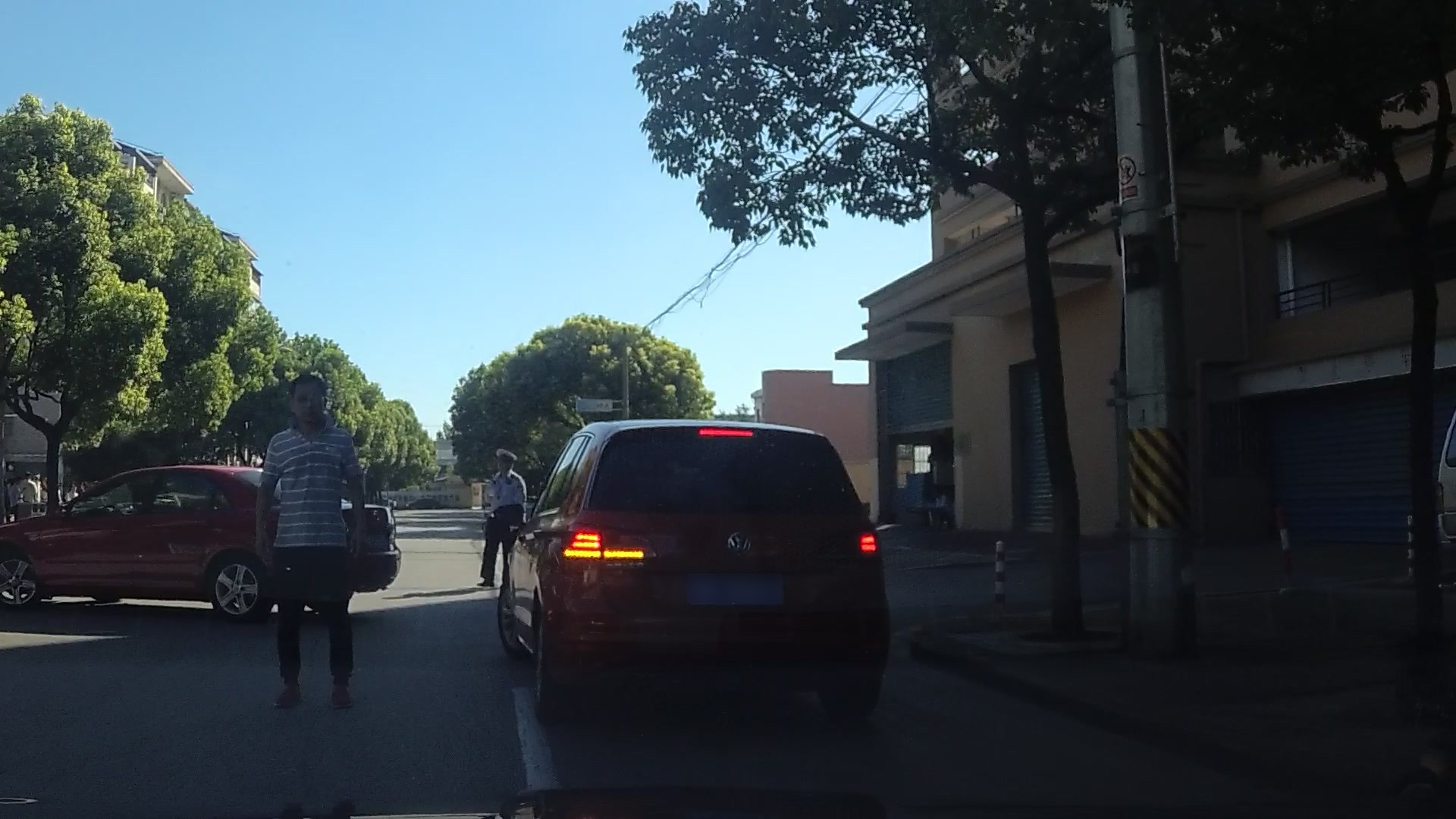

Supplement: S1 Dataset — All collected images were collected together, labeled and summarized one by one, and resulting classification results were roughly classified into three major categories: dry, wet and snowy. (ZIP) [file pone.0310858.s001.zip › weather1_data/dry_road/HT_TRAIN_000054_SH_000.jpg]

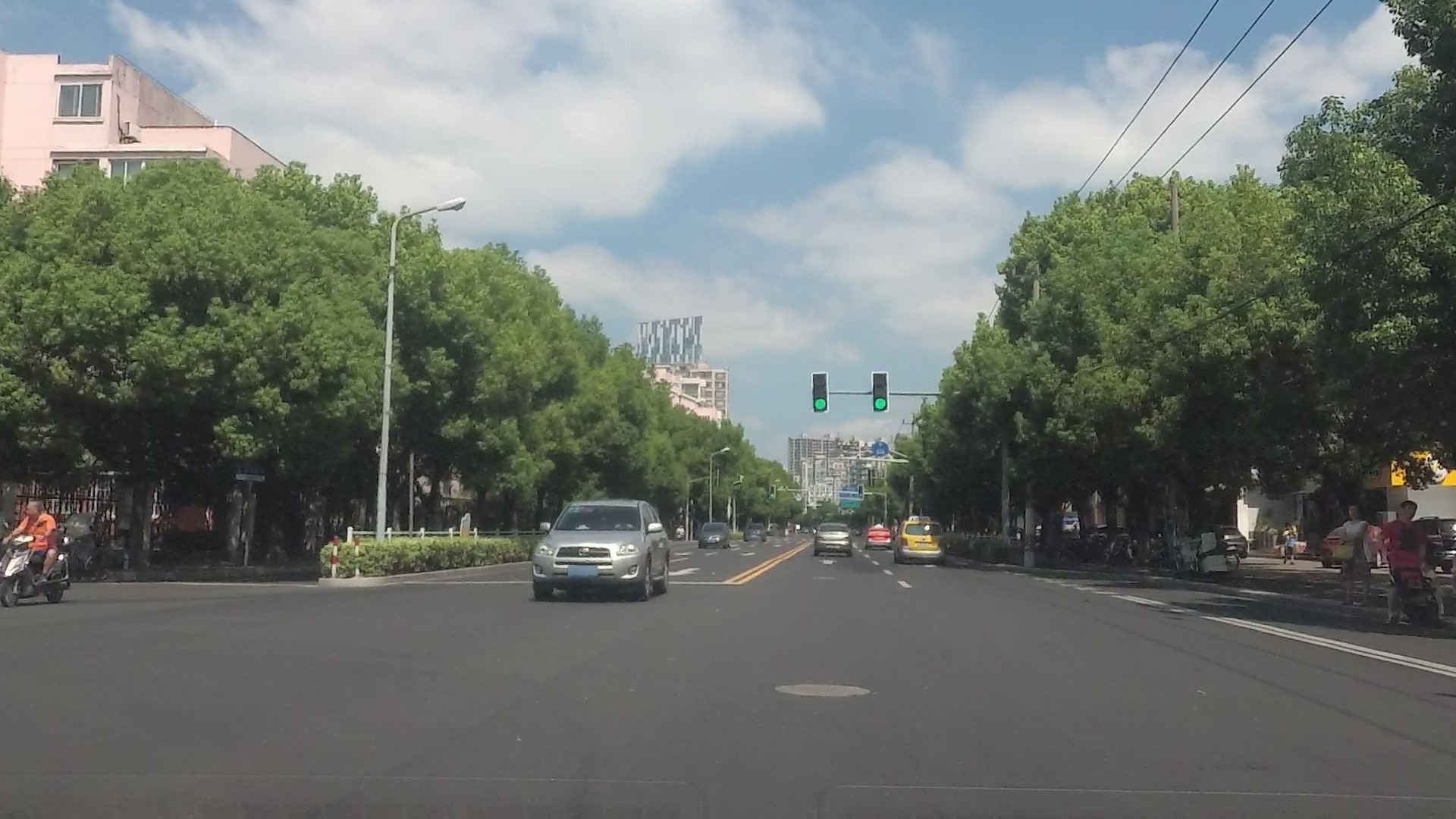

Supplement: S1 Dataset — All collected images were collected together, labeled and summarized one by one, and resulting classification results were roughly classified into three major categories: dry, wet and snowy. (ZIP) [file pone.0310858.s001.zip › weather1_data/dry_road/HT_TRAIN_000062_SH_000.jpg]

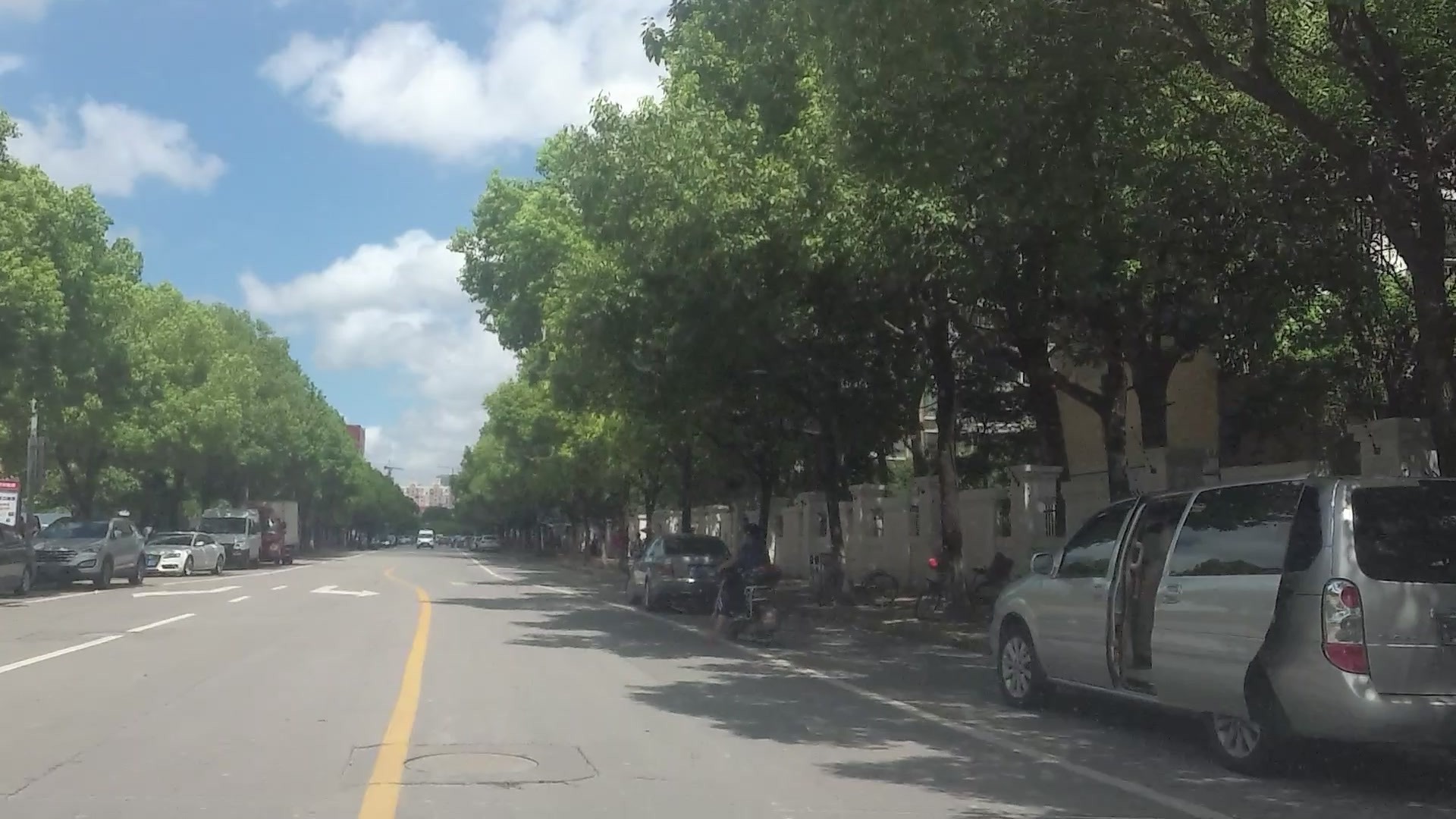

Supplement: S1 Dataset — All collected images were collected together, labeled and summarized one by one, and resulting classification results were roughly classified into three major categories: dry, wet and snowy. (ZIP) [file pone.0310858.s001.zip › weather1_data/dry_road/HT_TRAIN_000072_SH_000.jpg]

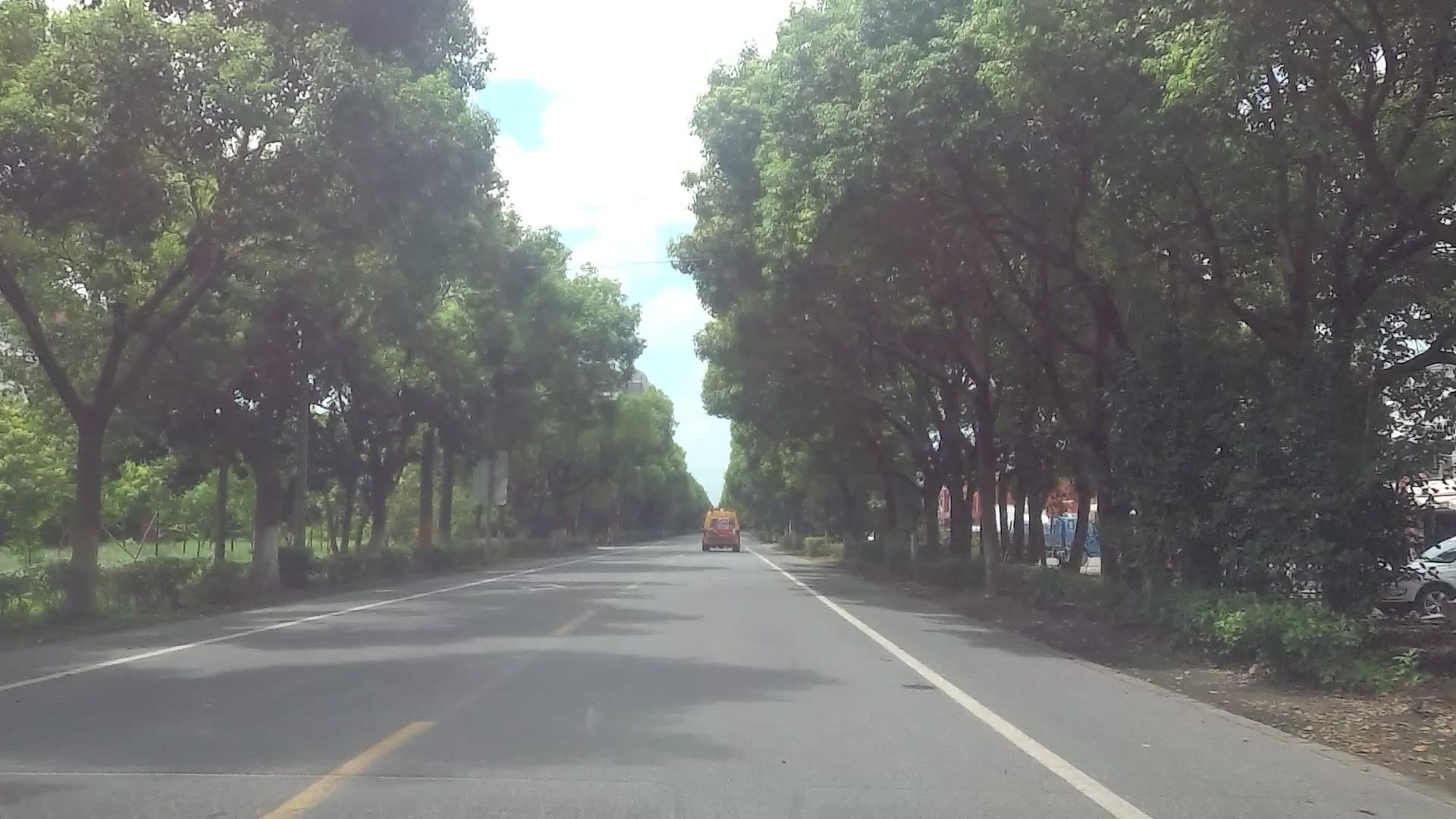

Supplement: S1 Dataset — All collected images were collected together, labeled and summarized one by one, and resulting classification results were roughly classified into three major categories: dry, wet and snowy. (ZIP) [file pone.0310858.s001.zip › weather1_data/dry_road/HT_TRAIN_000073_SH_000.jpg]

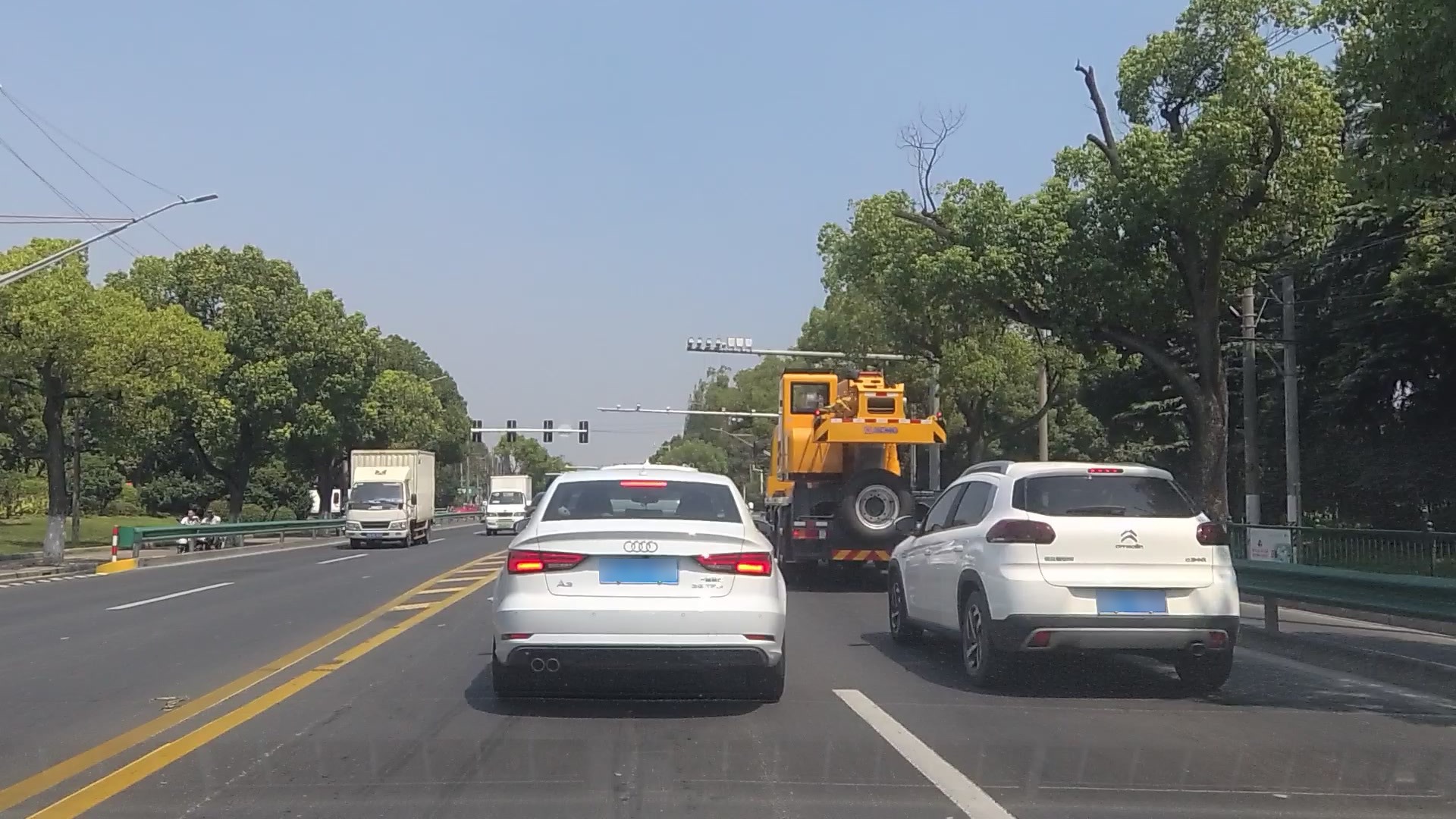

Supplement: S1 Dataset — All collected images were collected together, labeled and summarized one by one, and resulting classification results were roughly classified into three major categories: dry, wet and snowy. (ZIP) [file pone.0310858.s001.zip › weather1_data/dry_road/HT_TRAIN_000078_SH_000.jpg]

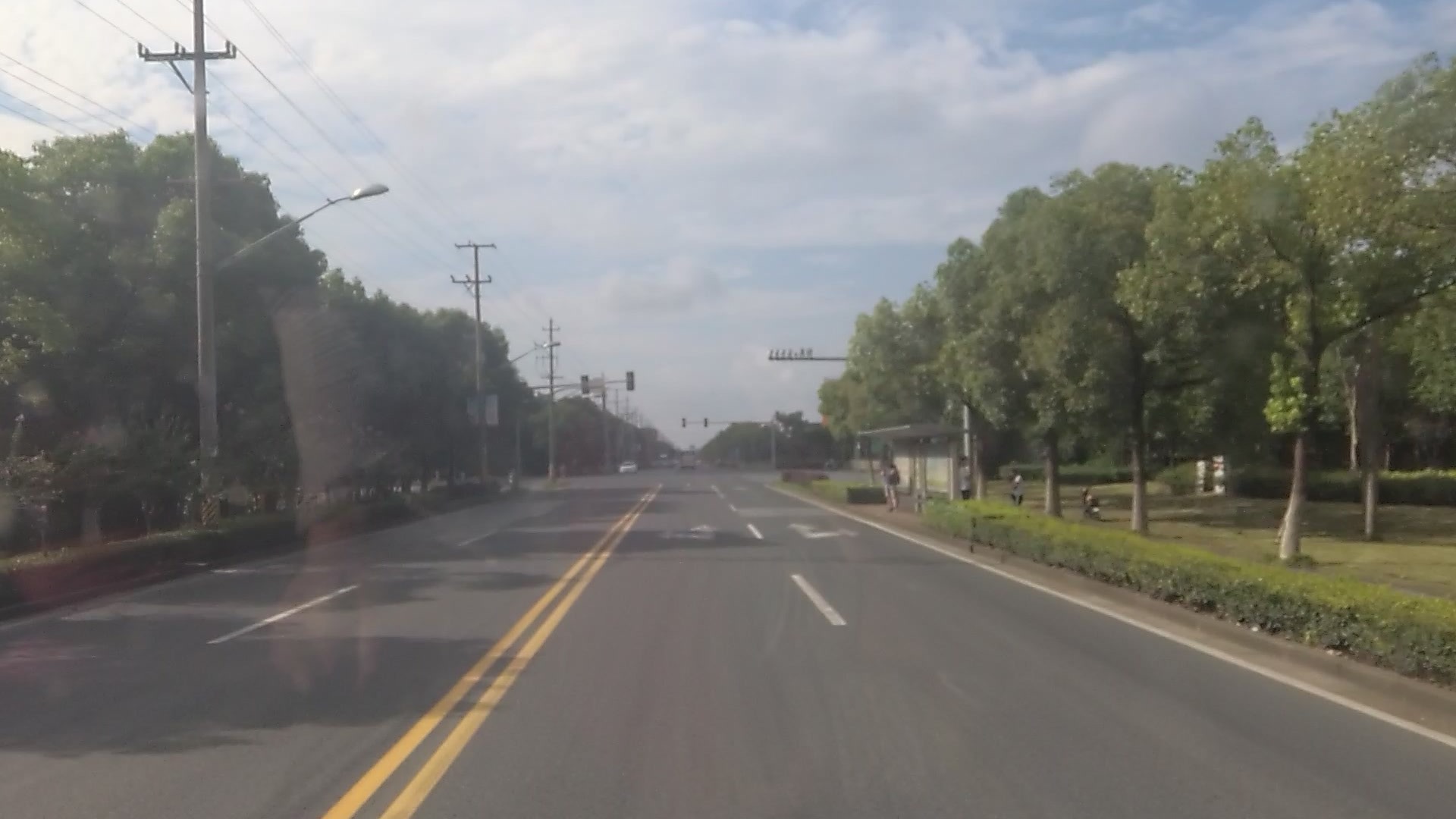

Supplement: S1 Dataset — All collected images were collected together, labeled and summarized one by one, and resulting classification results were roughly classified into three major categories: dry, wet and snowy. (ZIP) [file pone.0310858.s001.zip › weather1_data/dry_road/HT_TRAIN_000082_SH_000.jpg]

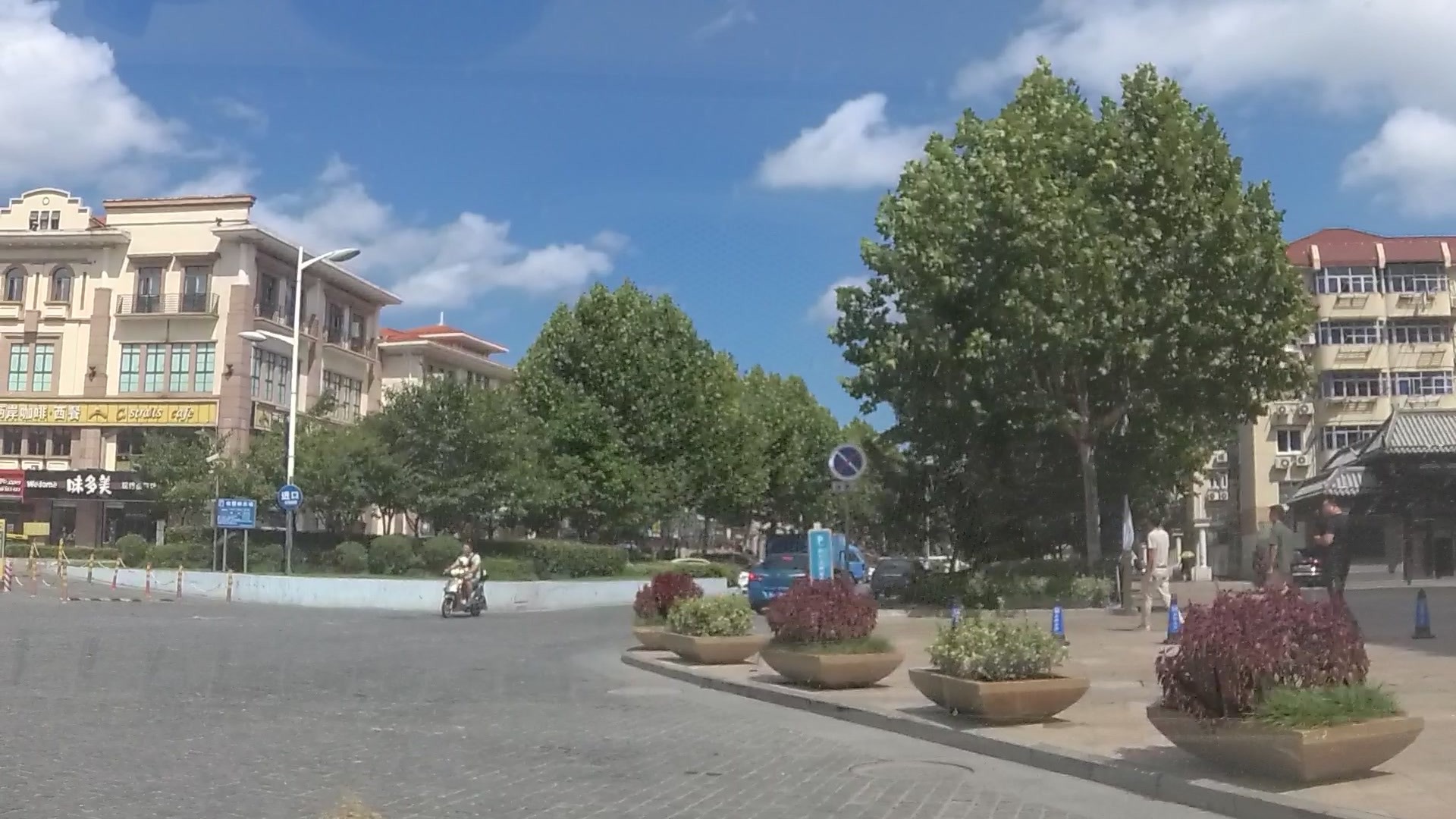

Supplement: S1 Dataset — All collected images were collected together, labeled and summarized one by one, and resulting classification results were roughly classified into three major categories: dry, wet and snowy. (ZIP) [file pone.0310858.s001.zip › weather1_data/dry_road/HT_TRAIN_000168_SH_000.jpg]

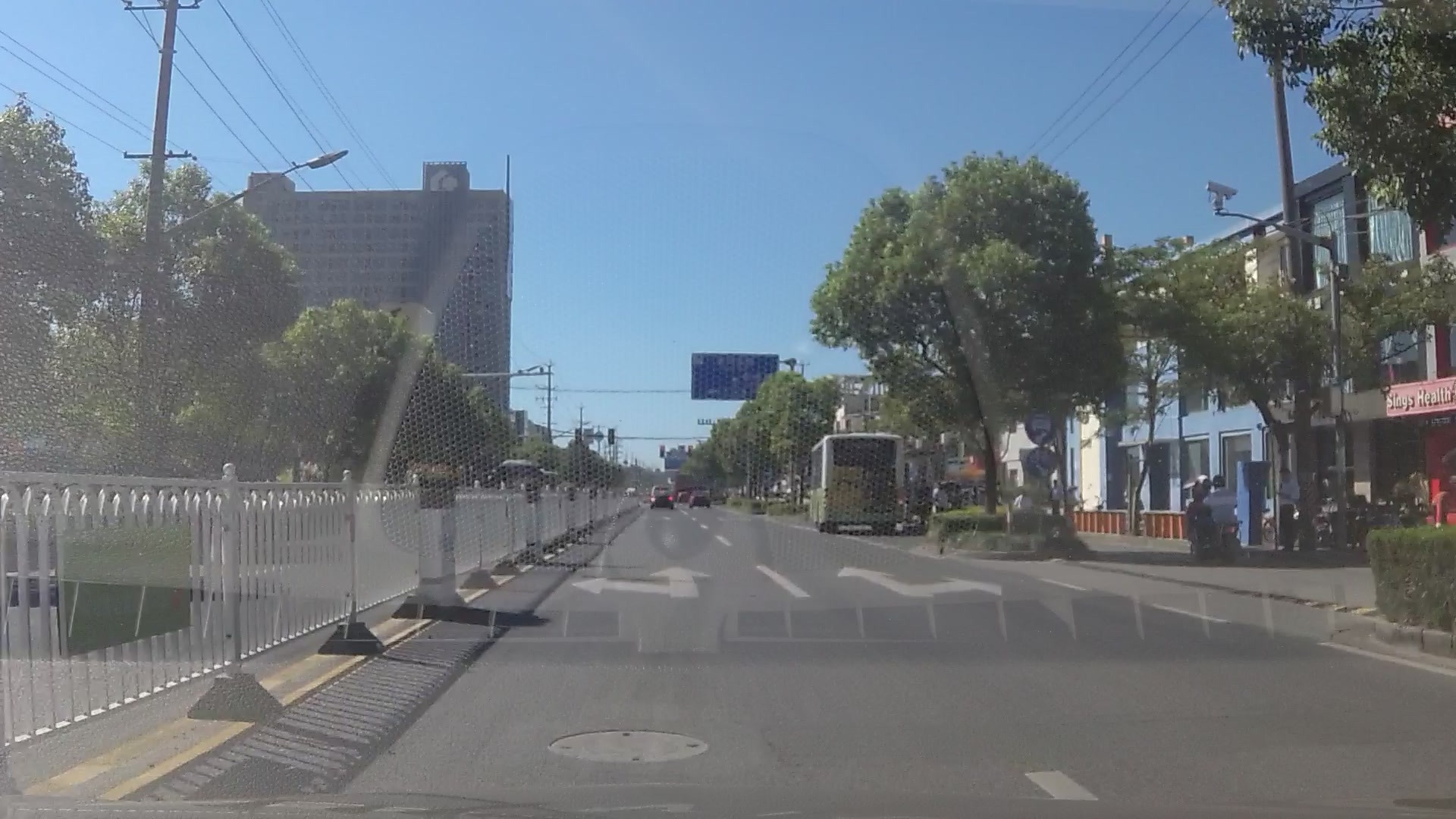

Supplement: S1 Dataset — All collected images were collected together, labeled and summarized one by one, and resulting classification results were roughly classified into three major categories: dry, wet and snowy. (ZIP) [file pone.0310858.s001.zip › weather1_data/dry_road/HT_TRAIN_000173_SH_000.jpg]

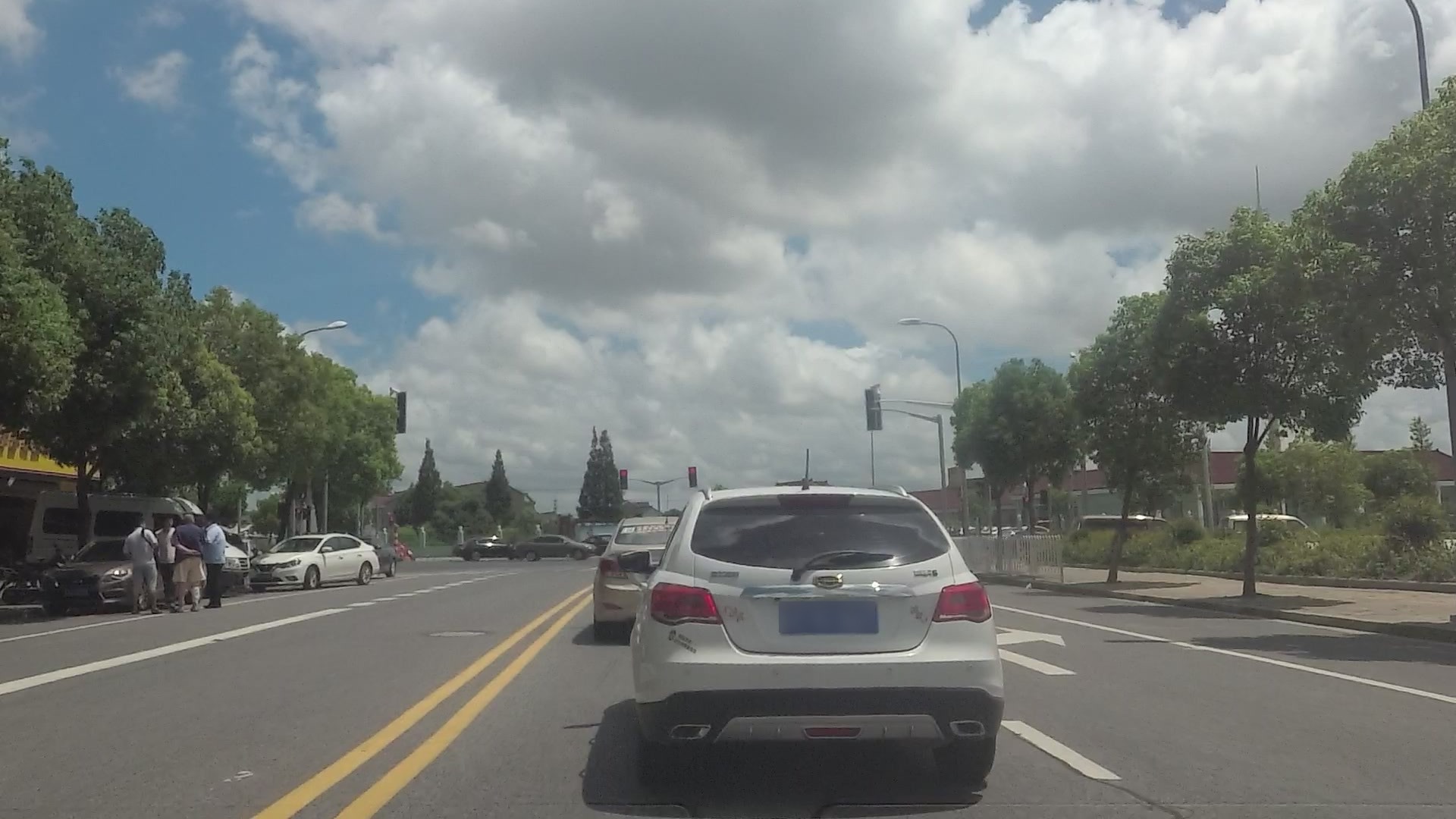

Supplement: S1 Dataset — All collected images were collected together, labeled and summarized one by one, and resulting classification results were roughly classified into three major categories: dry, wet and snowy. (ZIP) [file pone.0310858.s001.zip › weather1_data/dry_road/HT_TRAIN_000175_SH_000.jpg]

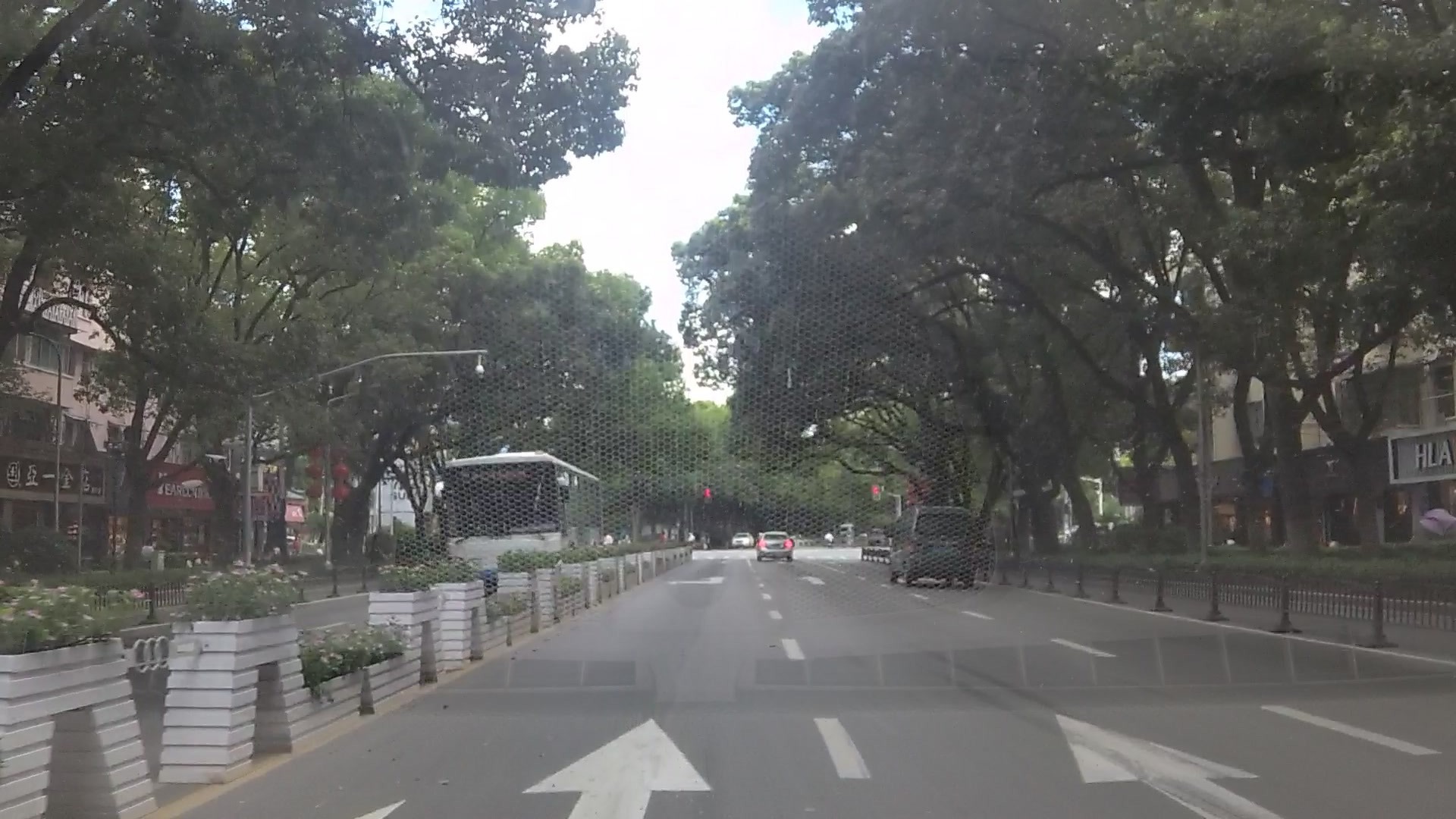

Supplement: S1 Dataset — All collected images were collected together, labeled and summarized one by one, and resulting classification results were roughly classified into three major categories: dry, wet and snowy. (ZIP) [file pone.0310858.s001.zip › weather1_data/dry_road/HT_TRAIN_000226_SH_000.jpg]

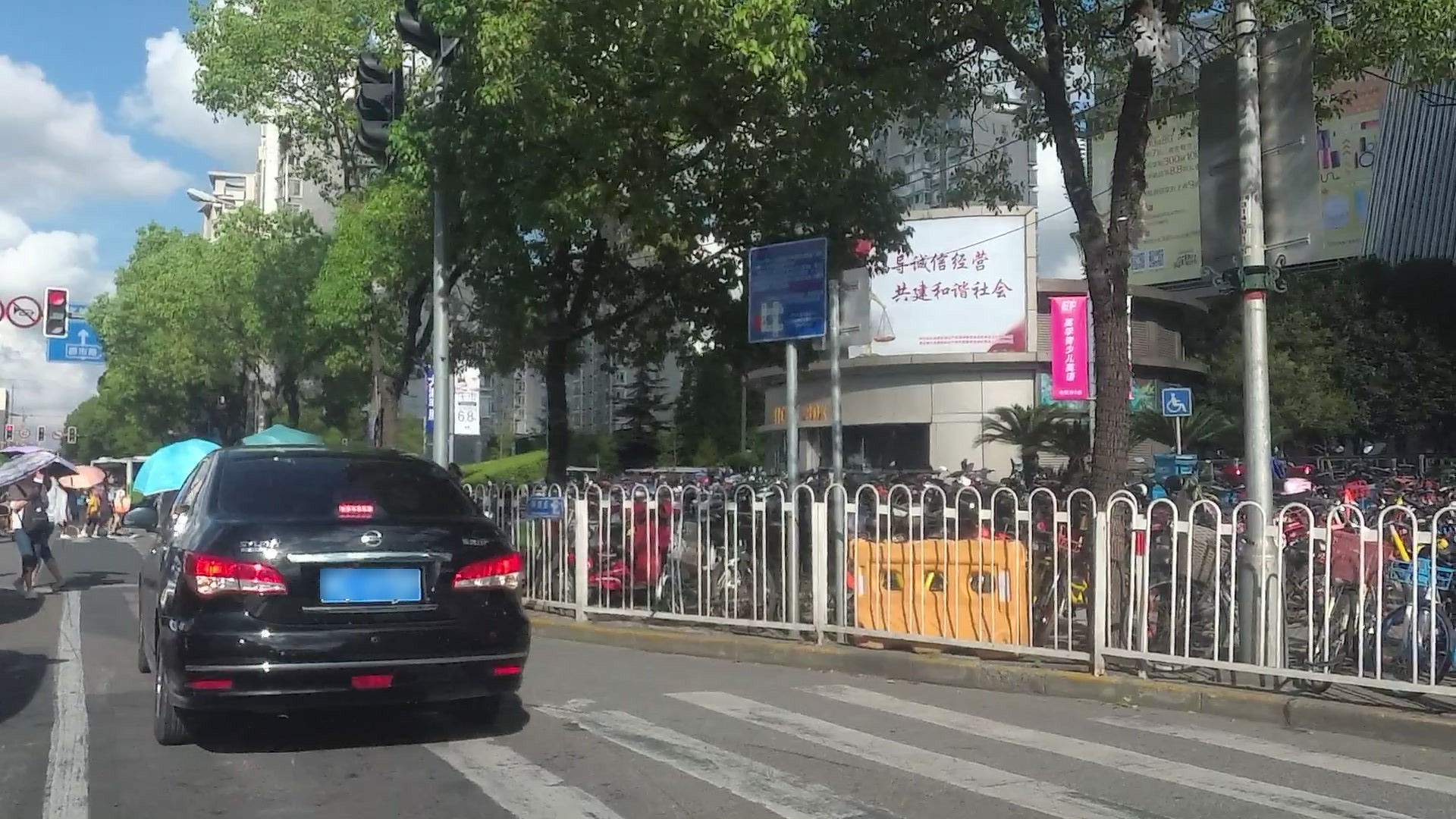

Supplement: S1 Dataset — All collected images were collected together, labeled and summarized one by one, and resulting classification results were roughly classified into three major categories: dry, wet and snowy. (ZIP) [file pone.0310858.s001.zip › weather1_data/dry_road/HT_TRAIN_000243_SH_000.jpg]

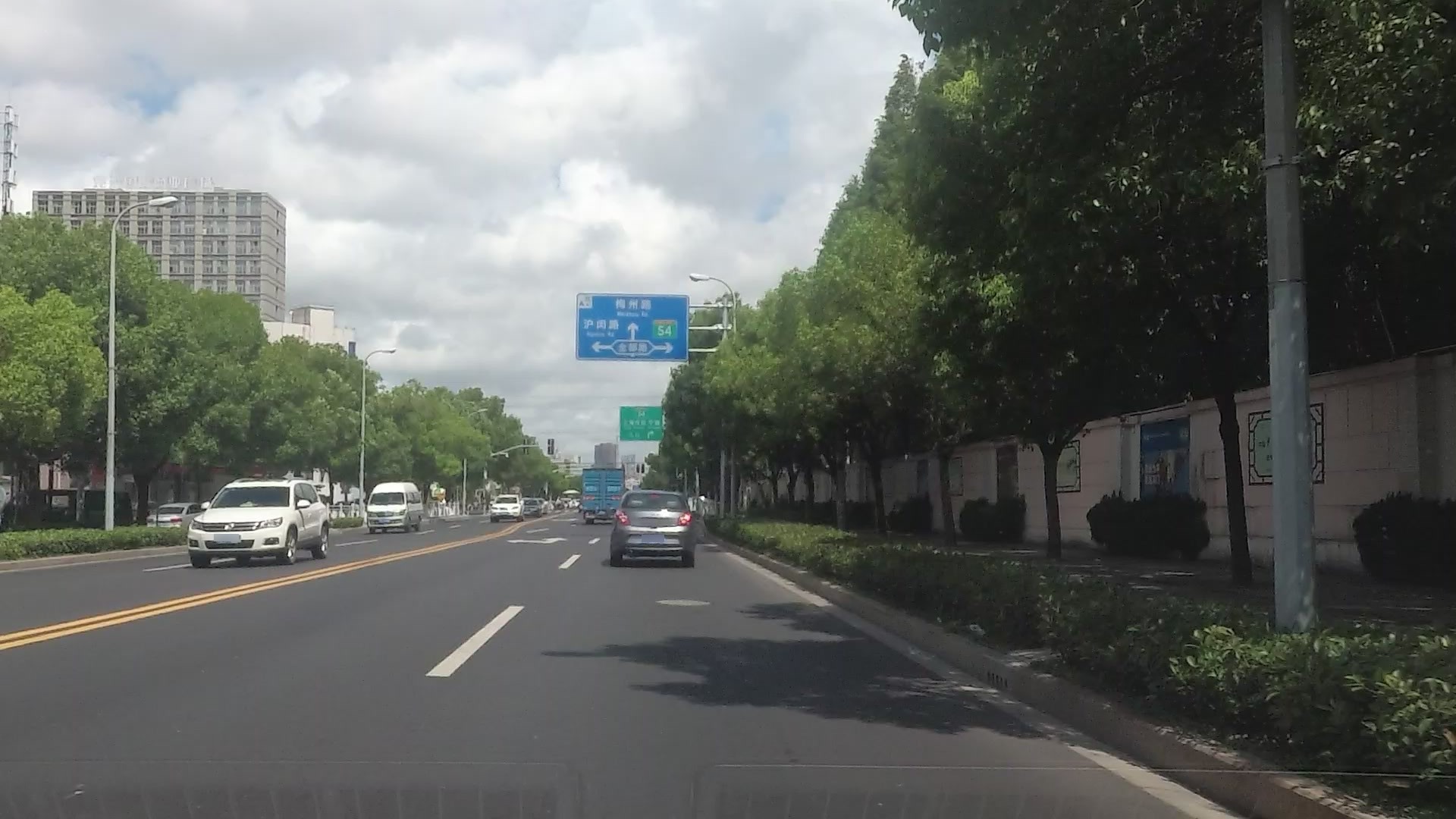

Supplement: S1 Dataset — All collected images were collected together, labeled and summarized one by one, and resulting classification results were roughly classified into three major categories: dry, wet and snowy. (ZIP) [file pone.0310858.s001.zip › weather1_data/dry_road/HT_TRAIN_000254_SH_000.jpg]

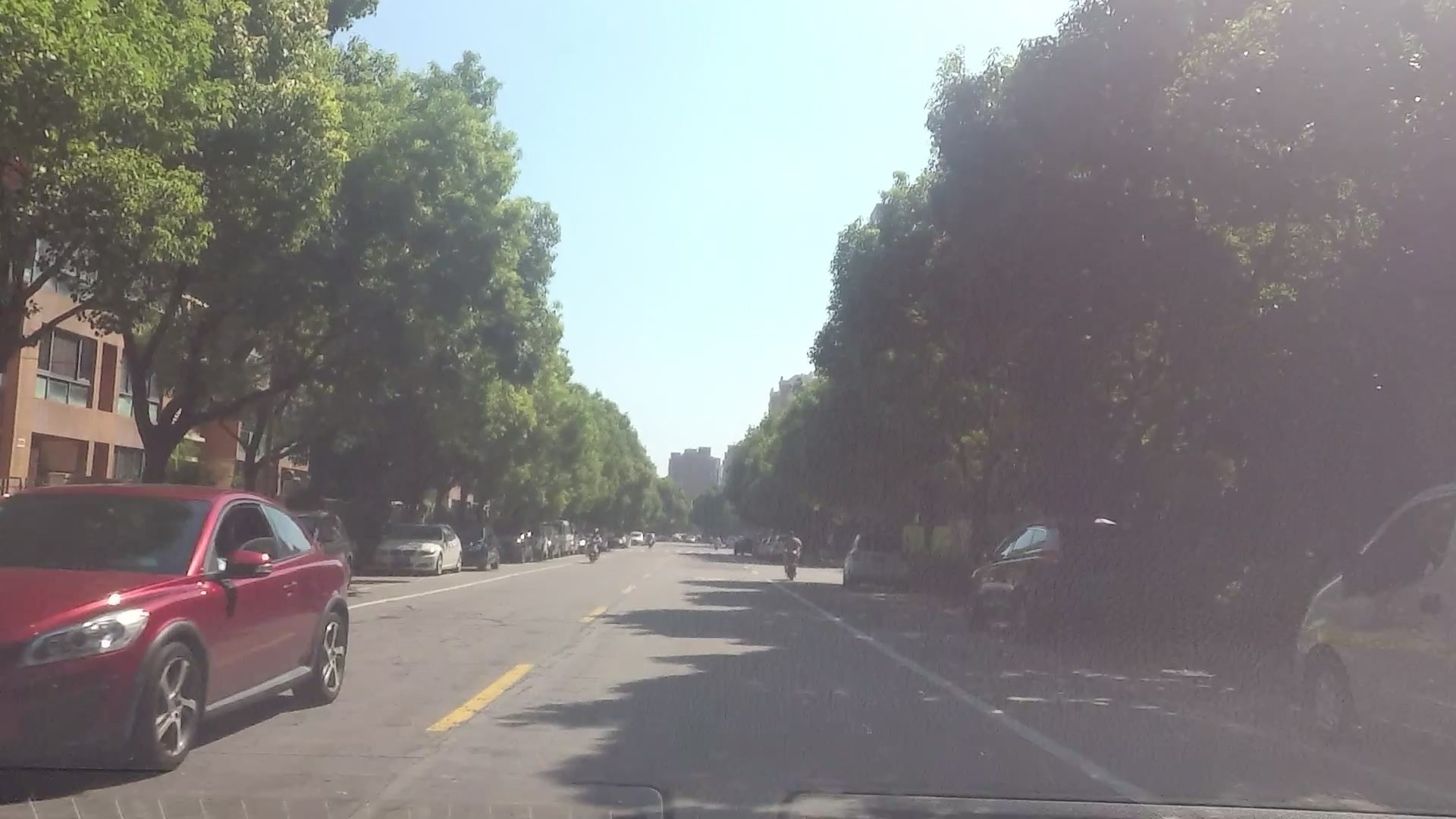

Supplement: S1 Dataset — All collected images were collected together, labeled and summarized one by one, and resulting classification results were roughly classified into three major categories: dry, wet and snowy. (ZIP) [file pone.0310858.s001.zip › weather1_data/dry_road/HT_TRAIN_000259_SH_000.jpg]

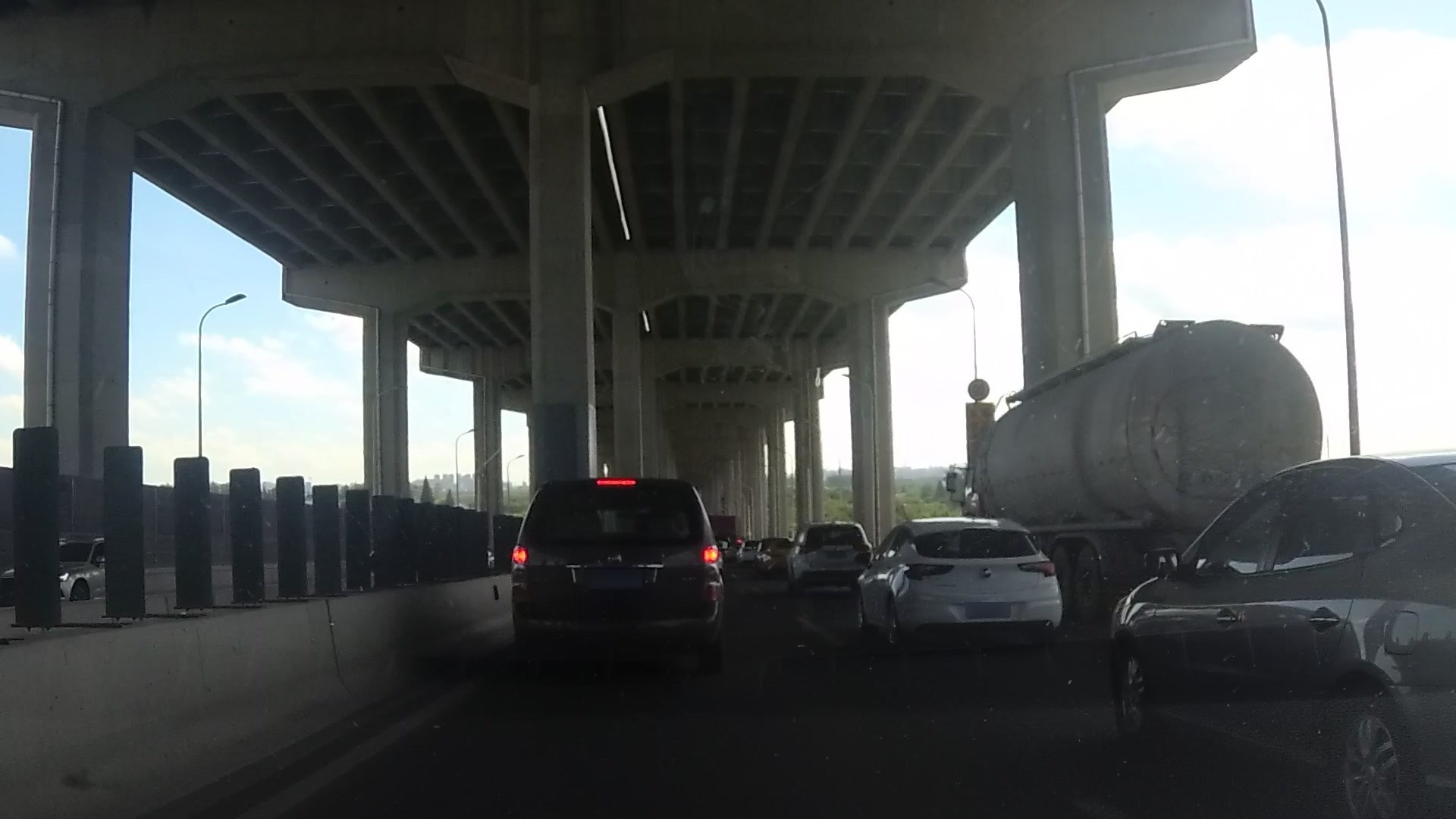

Supplement: S1 Dataset — All collected images were collected together, labeled and summarized one by one, and resulting classification results were roughly classified into three major categories: dry, wet and snowy. (ZIP) [file pone.0310858.s001.zip › weather1_data/dry_road/HT_TRAIN_000260_SH_000.jpg]

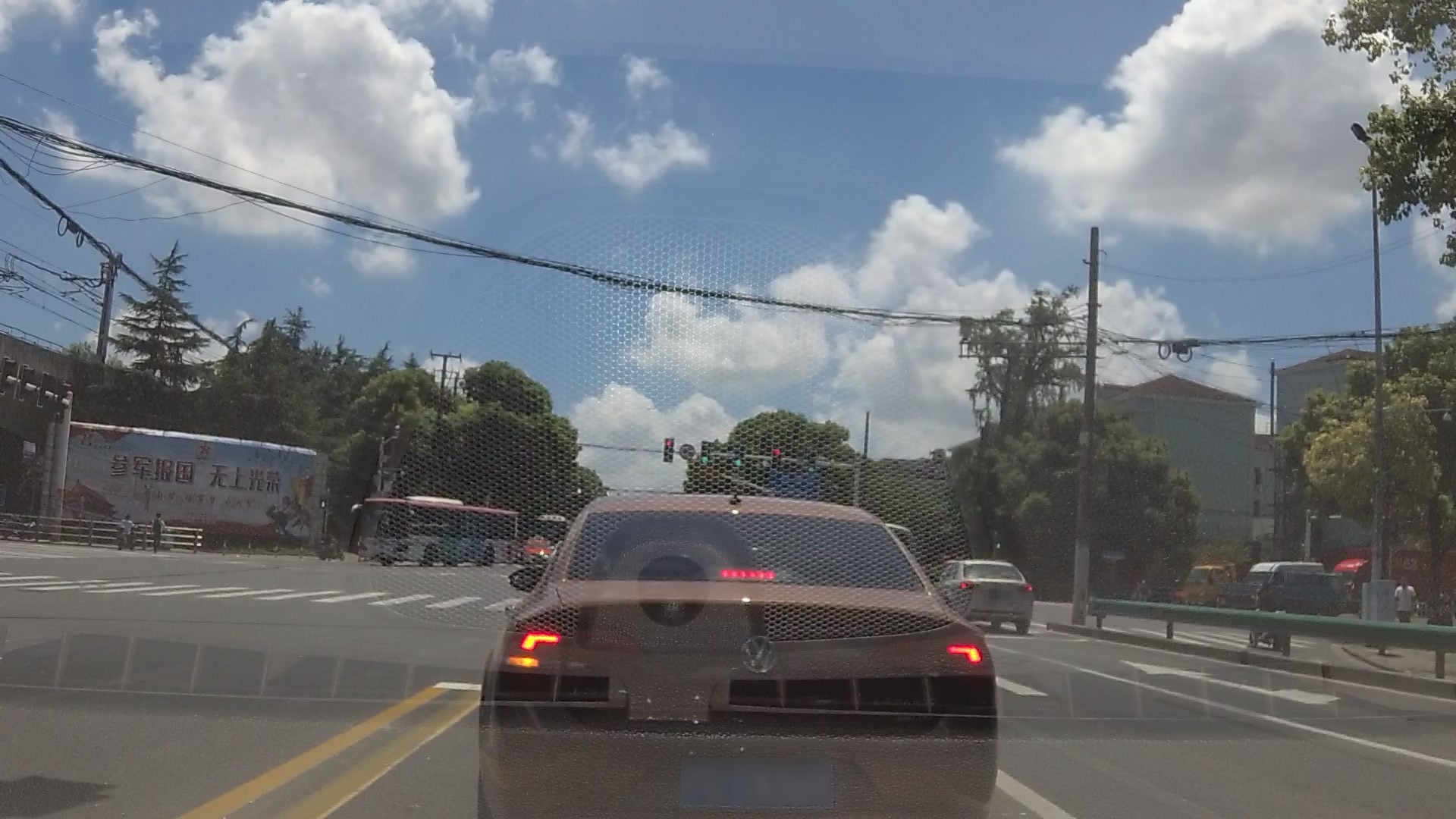

Supplement: S1 Dataset — All collected images were collected together, labeled and summarized one by one, and resulting classification results were roughly classified into three major categories: dry, wet and snowy. (ZIP) [file pone.0310858.s001.zip › weather1_data/dry_road/HT_TRAIN_000261_SH_000.jpg]

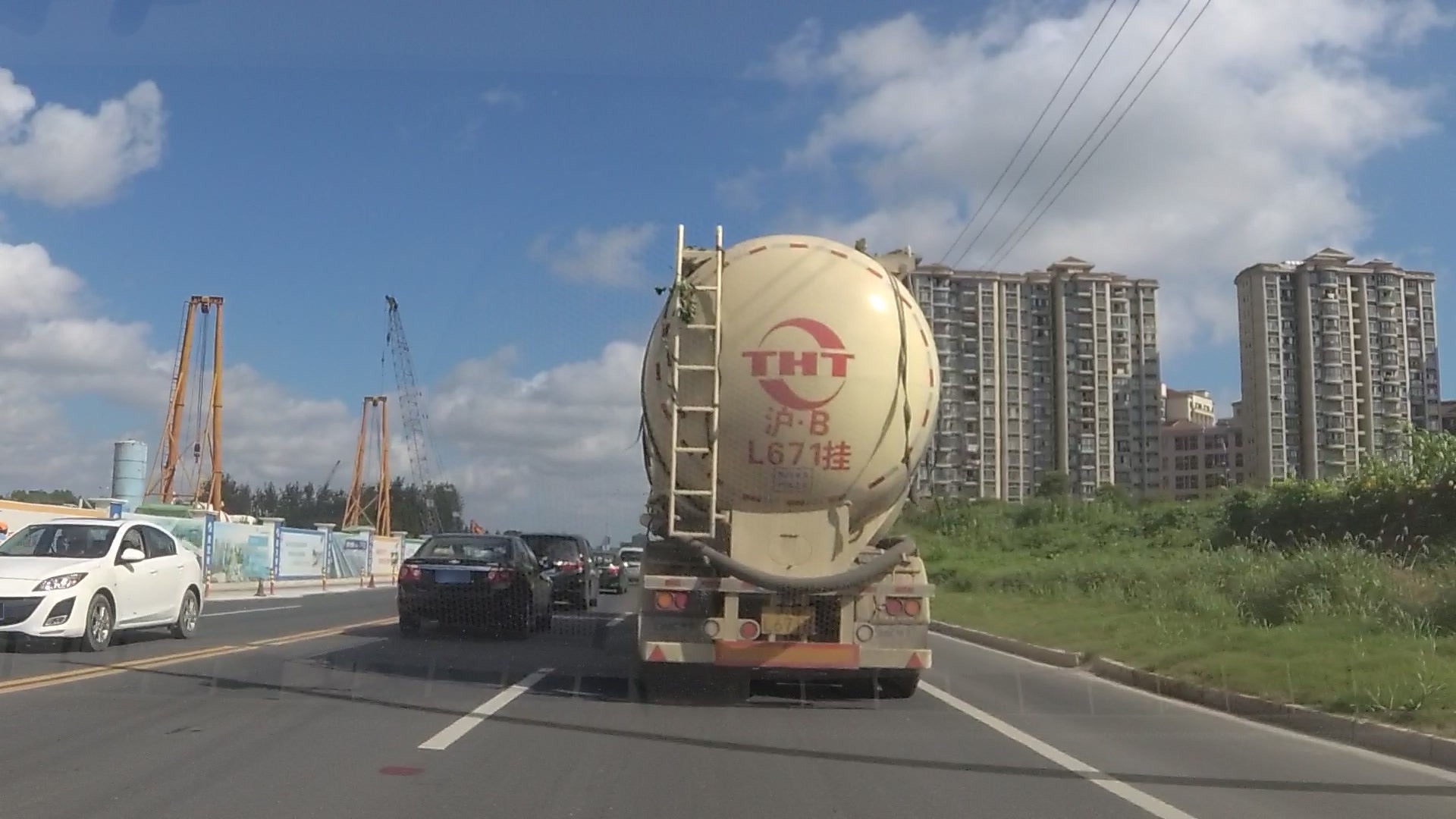

Supplement: S1 Dataset — All collected images were collected together, labeled and summarized one by one, and resulting classification results were roughly classified into three major categories: dry, wet and snowy. (ZIP) [file pone.0310858.s001.zip › weather1_data/dry_road/HT_TRAIN_000262_SH_000.jpg]

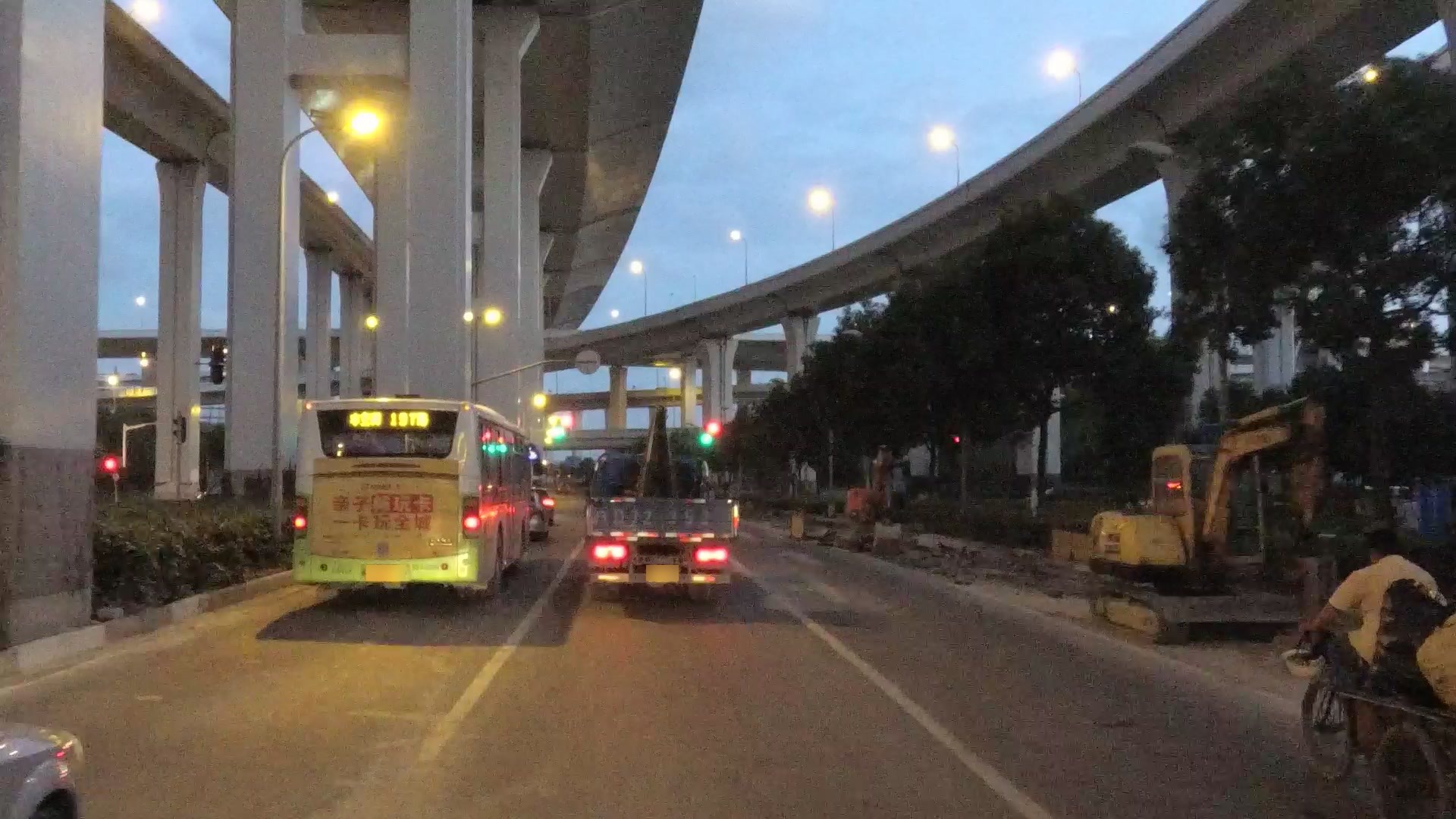

Supplement: S1 Dataset — All collected images were collected together, labeled and summarized one by one, and resulting classification results were roughly classified into three major categories: dry, wet and snowy. (ZIP) [file pone.0310858.s001.zip › weather1_data/dry_road/HT_TRAIN_000263_SH_000.jpg]

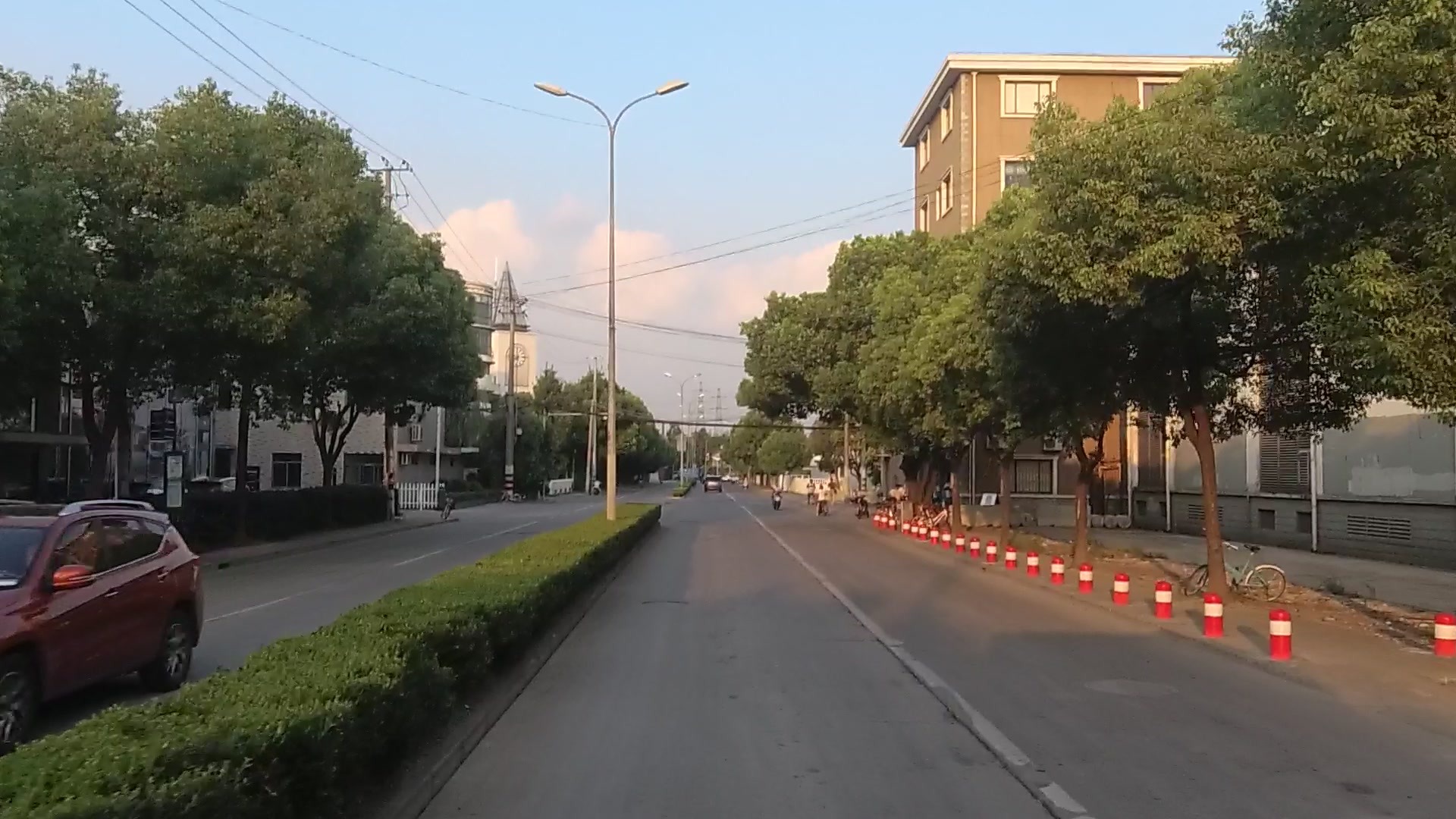

Supplement: S1 Dataset — All collected images were collected together, labeled and summarized one by one, and resulting classification results were roughly classified into three major categories: dry, wet and snowy. (ZIP) [file pone.0310858.s001.zip › weather1_data/dry_road/HT_TRAIN_000265_SH_000.jpg]

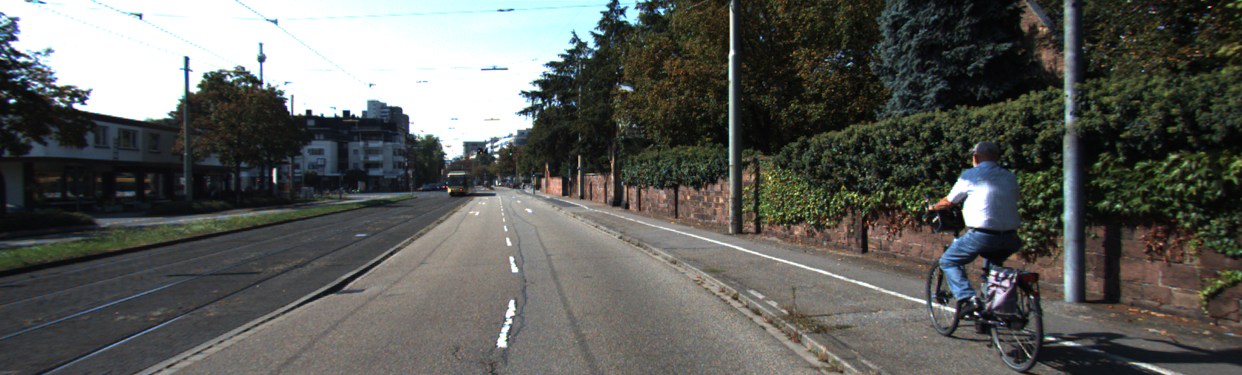

Supplement: S1 Dataset — All collected images were collected together, labeled and summarized one by one, and resulting classification results were roughly classified into three major categories: dry, wet and snowy. (ZIP) [file pone.0310858.s001.zip › weather1_data/dry_road/um_000000.png]

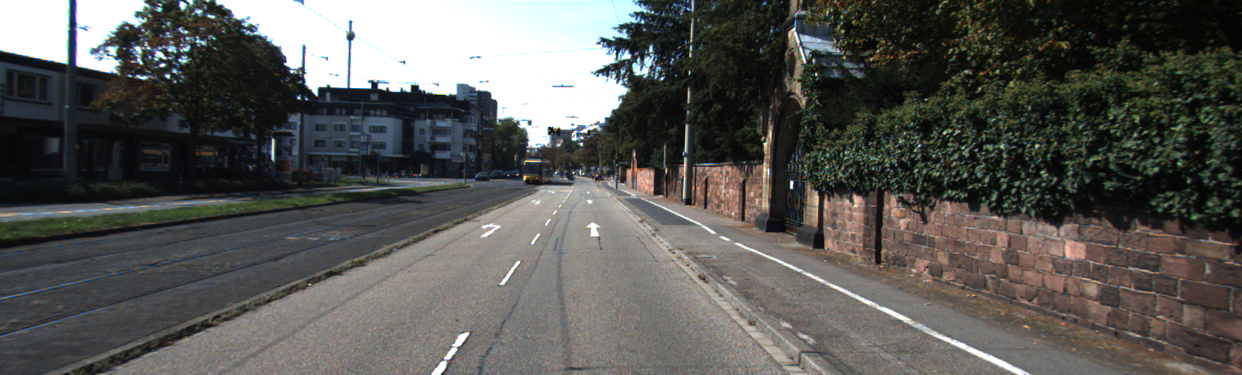

Supplement: S1 Dataset — All collected images were collected together, labeled and summarized one by one, and resulting classification results were roughly classified into three major categories: dry, wet and snowy. (ZIP) [file pone.0310858.s001.zip › weather1_data/dry_road/um_000001.png]

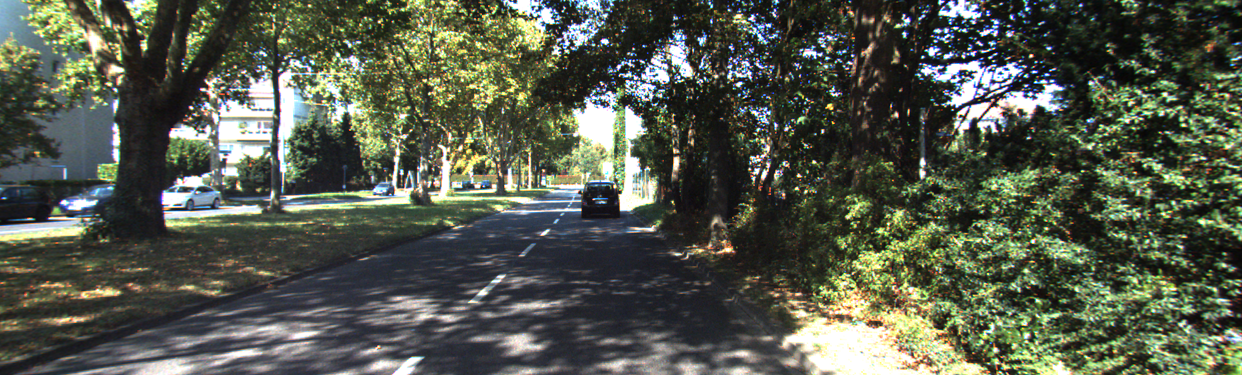

Supplement: S1 Dataset — All collected images were collected together, labeled and summarized one by one, and resulting classification results were roughly classified into three major categories: dry, wet and snowy. (ZIP) [file pone.0310858.s001.zip › weather1_data/dry_road/um_000009.png]

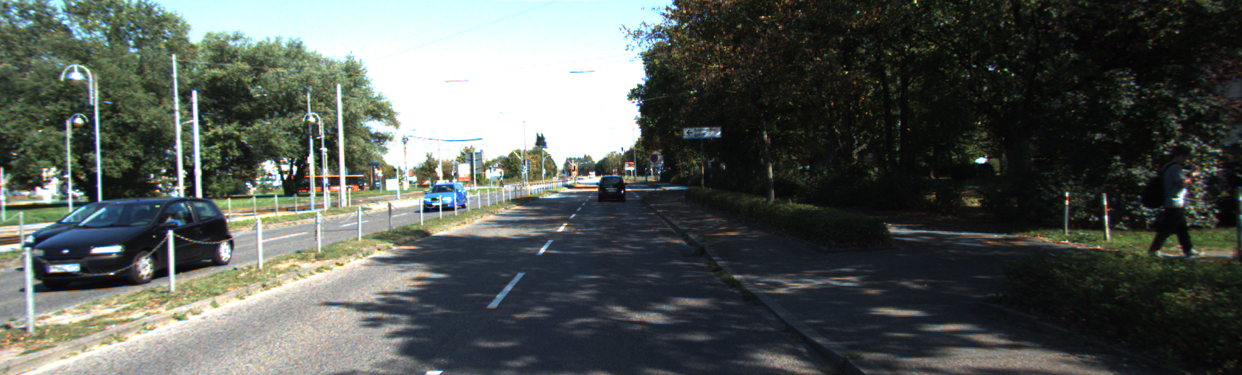

Supplement: S1 Dataset — All collected images were collected together, labeled and summarized one by one, and resulting classification results were roughly classified into three major categories: dry, wet and snowy. (ZIP) [file pone.0310858.s001.zip › weather1_data/dry_road/um_000022.png]

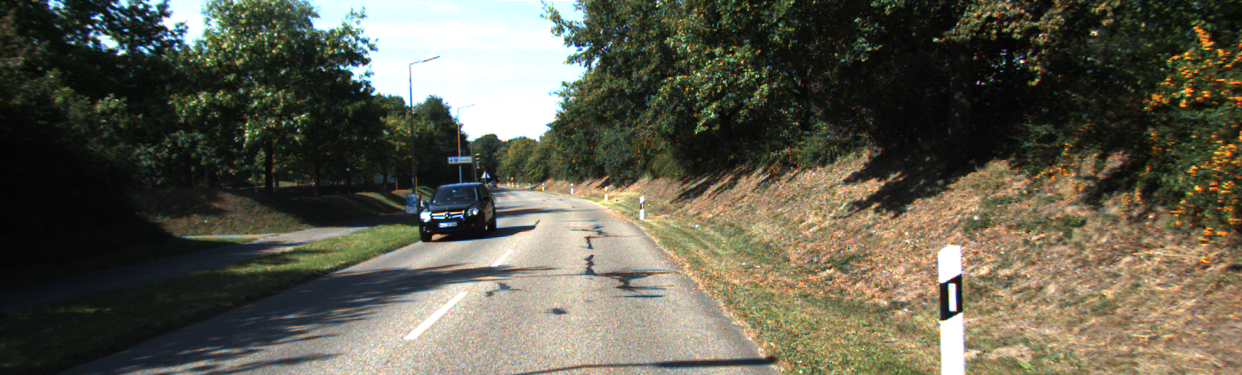

Supplement: S1 Dataset — All collected images were collected together, labeled and summarized one by one, and resulting classification results were roughly classified into three major categories: dry, wet and snowy. (ZIP) [file pone.0310858.s001.zip › weather1_data/dry_road/um_000042.png]

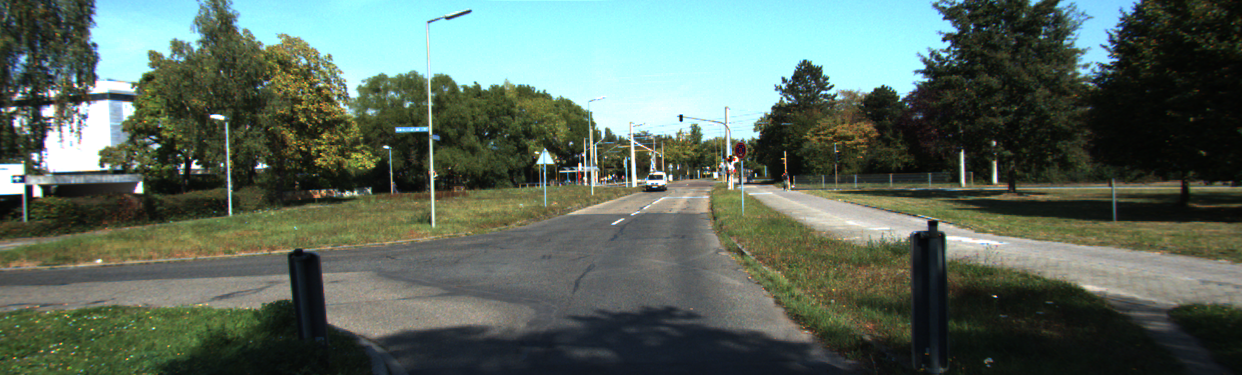

Supplement: S1 Dataset — All collected images were collected together, labeled and summarized one by one, and resulting classification results were roughly classified into three major categories: dry, wet and snowy. (ZIP) [file pone.0310858.s001.zip › weather1_data/dry_road/um_000044.png]

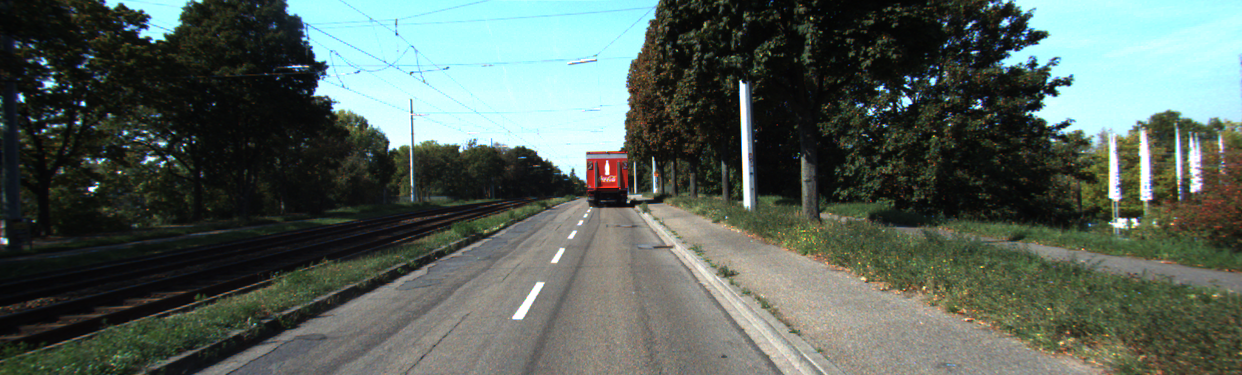

Supplement: S1 Dataset — All collected images were collected together, labeled and summarized one by one, and resulting classification results were roughly classified into three major categories: dry, wet and snowy. (ZIP) [file pone.0310858.s001.zip › weather1_data/dry_road/um_000052.png]

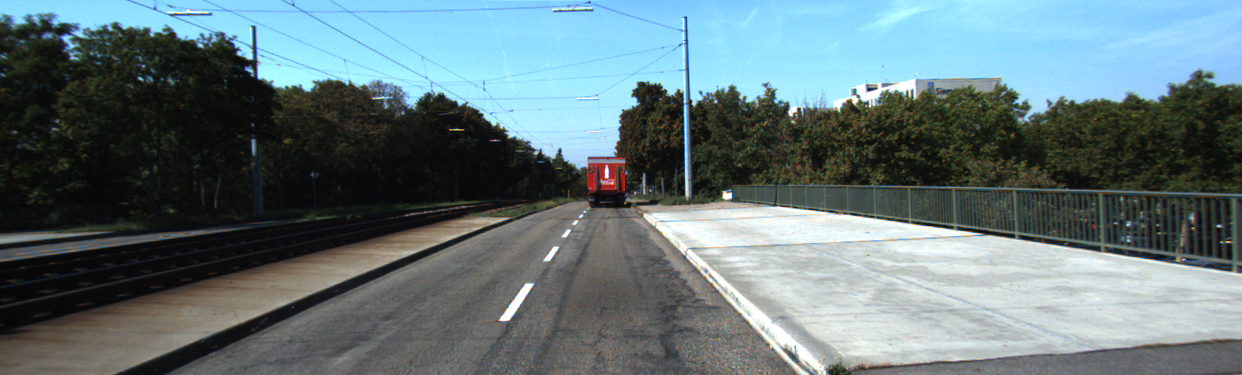

Supplement: S1 Dataset — All collected images were collected together, labeled and summarized one by one, and resulting classification results were roughly classified into three major categories: dry, wet and snowy. (ZIP) [file pone.0310858.s001.zip › weather1_data/dry_road/um_000054.png]

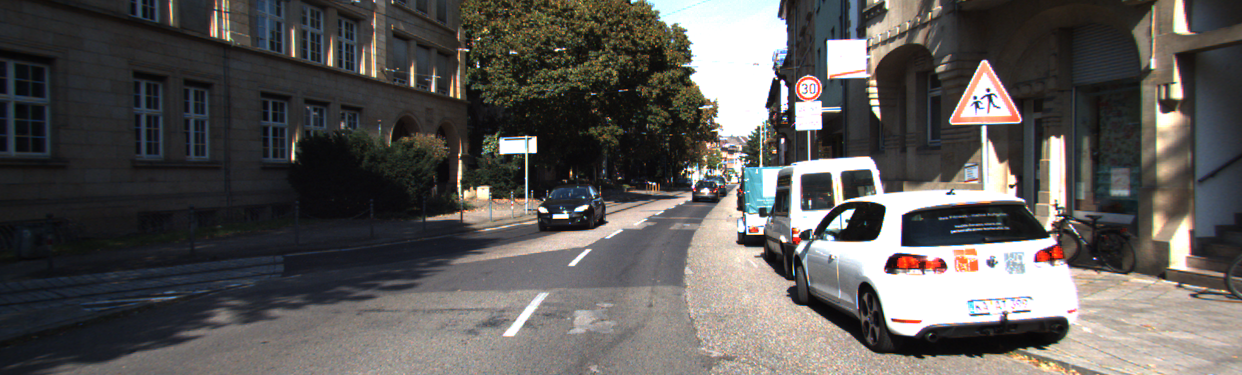

Supplement: S1 Dataset — All collected images were collected together, labeled and summarized one by one, and resulting classification results were roughly classified into three major categories: dry, wet and snowy. (ZIP) [file pone.0310858.s001.zip › weather1_data/dry_road/um_000070.png]

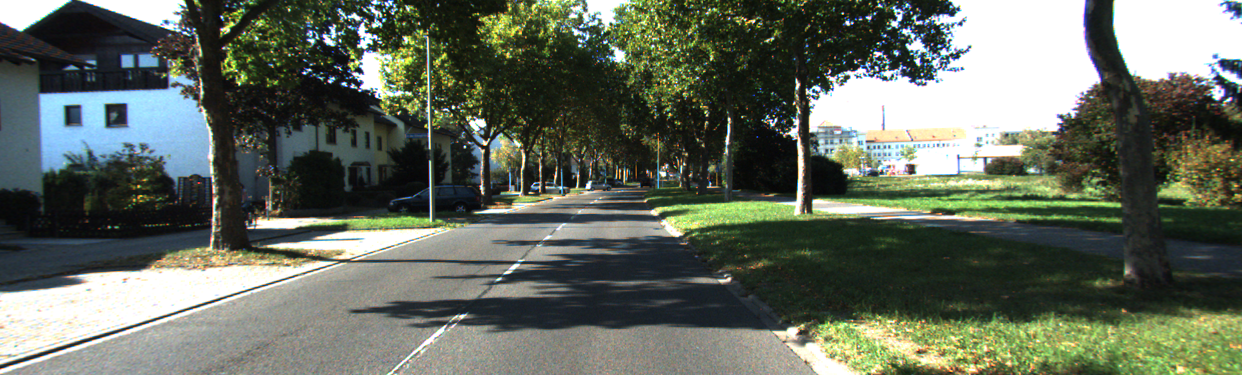

Supplement: S1 Dataset — All collected images were collected together, labeled and summarized one by one, and resulting classification results were roughly classified into three major categories: dry, wet and snowy. (ZIP) [file pone.0310858.s001.zip › weather1_data/dry_road/um_000073.png]

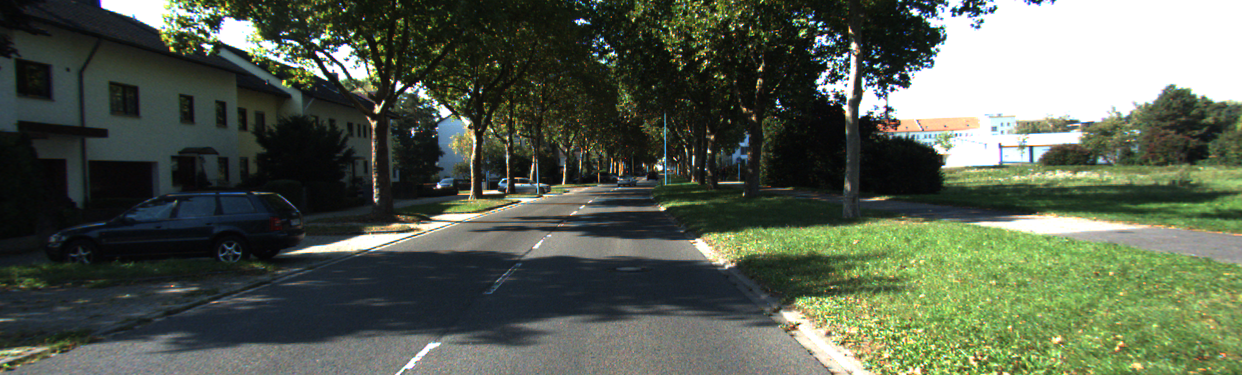

Supplement: S1 Dataset — All collected images were collected together, labeled and summarized one by one, and resulting classification results were roughly classified into three major categories: dry, wet and snowy. (ZIP) [file pone.0310858.s001.zip › weather1_data/dry_road/um_000074.png]

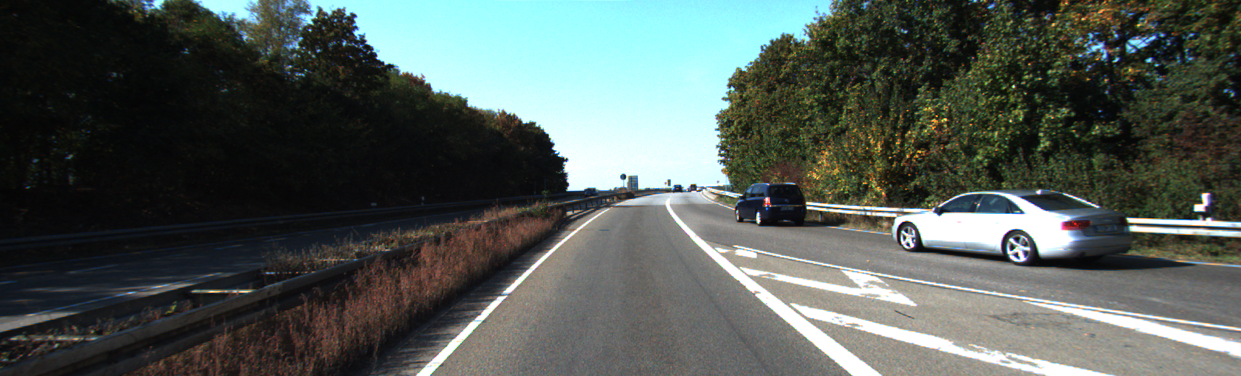

Supplement: S1 Dataset — All collected images were collected together, labeled and summarized one by one, and resulting classification results were roughly classified into three major categories: dry, wet and snowy. (ZIP) [file pone.0310858.s001.zip › weather1_data/dry_road/umm_000095.png]

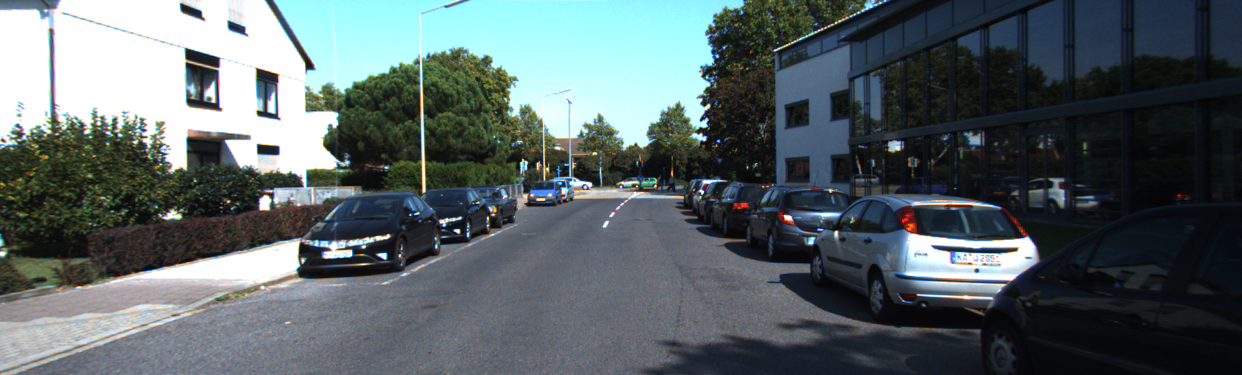

Supplement: S1 Dataset — All collected images were collected together, labeled and summarized one by one, and resulting classification results were roughly classified into three major categories: dry, wet and snowy. (ZIP) [file pone.0310858.s001.zip › weather1_data/dry_road/uu_000009.png]

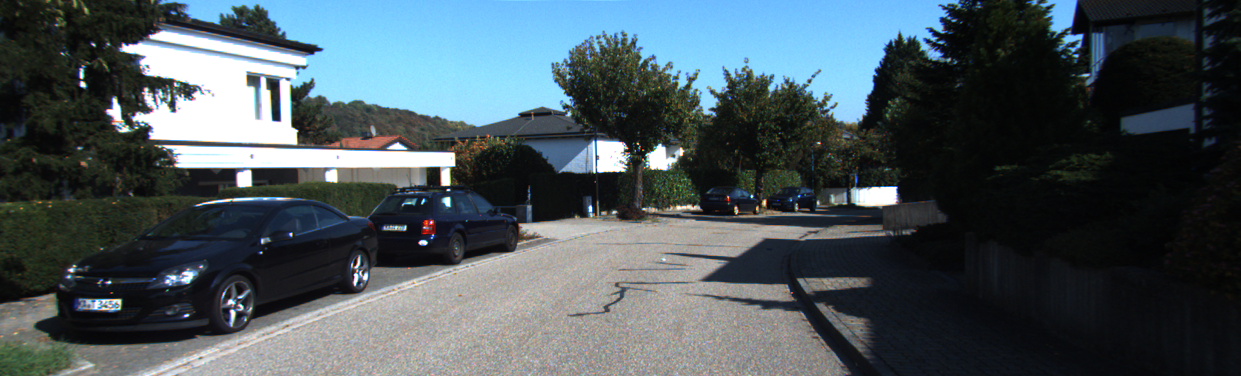

Supplement: S1 Dataset — All collected images were collected together, labeled and summarized one by one, and resulting classification results were roughly classified into three major categories: dry, wet and snowy. (ZIP) [file pone.0310858.s001.zip › weather1_data/dry_road/uu_000088.png]

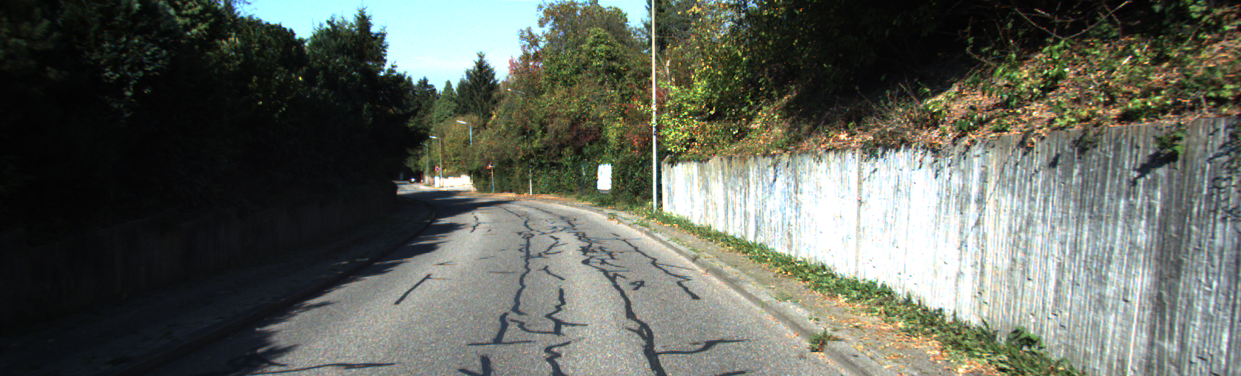

Supplement: S1 Dataset — All collected images were collected together, labeled and summarized one by one, and resulting classification results were roughly classified into three major categories: dry, wet and snowy. (ZIP) [file pone.0310858.s001.zip › weather1_data/dry_road/uu_000090.png]

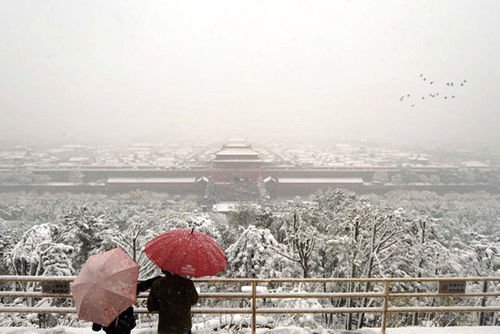

Supplement: S1 Dataset — All collected images were collected together, labeled and summarized one by one, and resulting classification results were roughly classified into three major categories: dry, wet and snowy. (ZIP) [file pone.0310858.s001.zip › weather1_data/snow_road/1271.jpg]

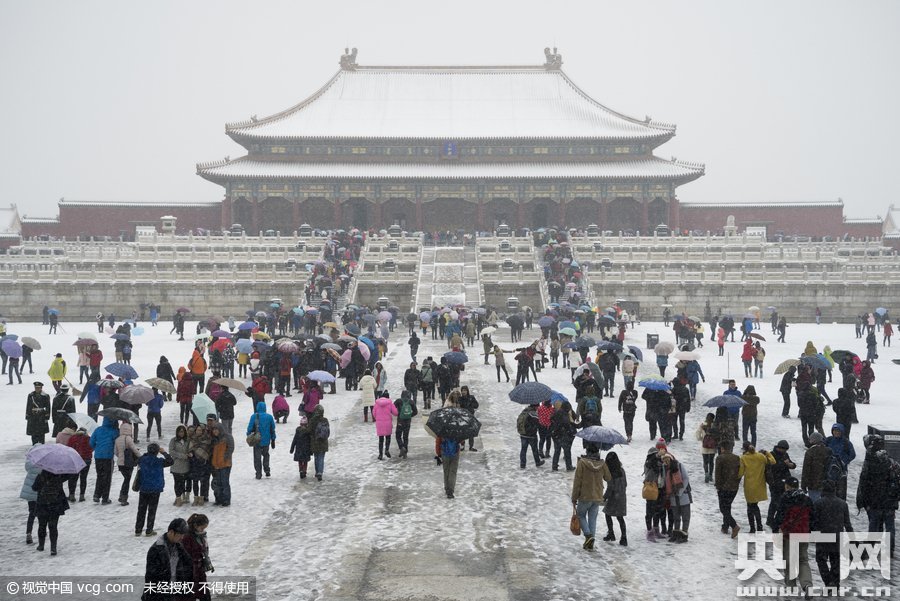

Supplement: S1 Dataset — All collected images were collected together, labeled and summarized one by one, and resulting classification results were roughly classified into three major categories: dry, wet and snowy. (ZIP) [file pone.0310858.s001.zip › weather1_data/snow_road/1273.jpg]

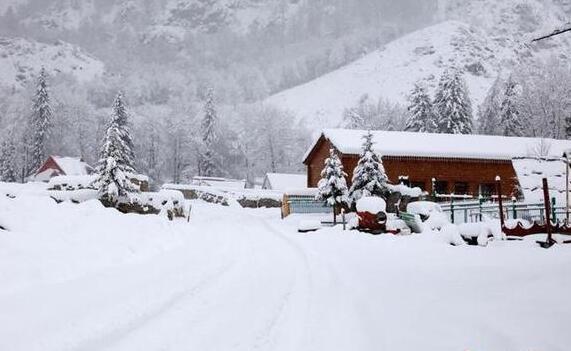

Supplement: S1 Dataset — All collected images were collected together, labeled and summarized one by one, and resulting classification results were roughly classified into three major categories: dry, wet and snowy. (ZIP) [file pone.0310858.s001.zip › weather1_data/snow_road/1275.jpg]

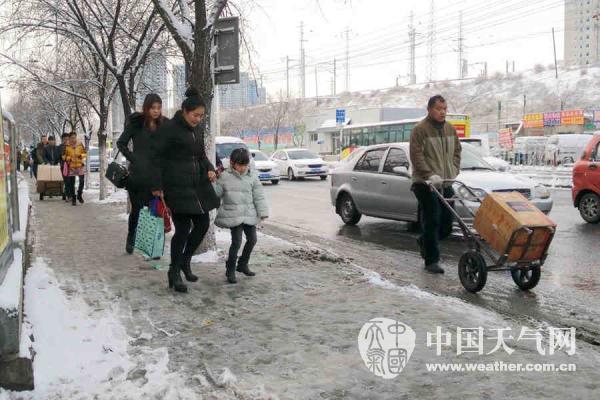

Supplement: S1 Dataset — All collected images were collected together, labeled and summarized one by one, and resulting classification results were roughly classified into three major categories: dry, wet and snowy. (ZIP) [file pone.0310858.s001.zip › weather1_data/snow_road/1276.jpg]

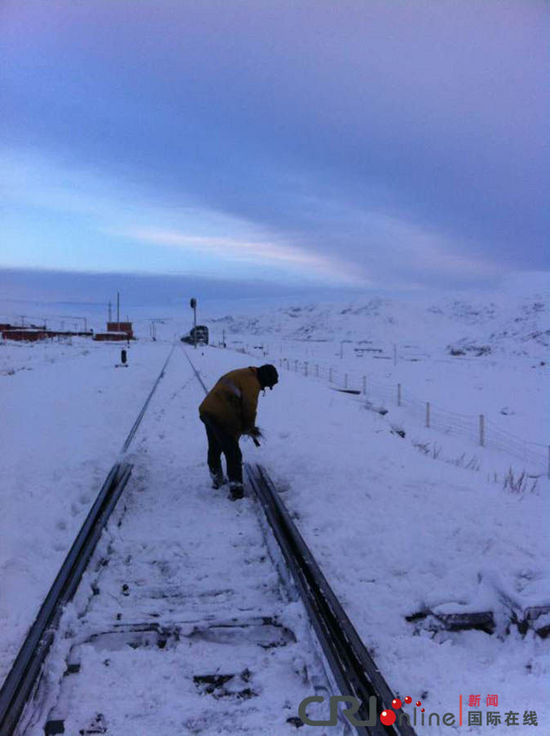

Supplement: S1 Dataset — All collected images were collected together, labeled and summarized one by one, and resulting classification results were roughly classified into three major categories: dry, wet and snowy. (ZIP) [file pone.0310858.s001.zip › weather1_data/snow_road/1278.jpg]

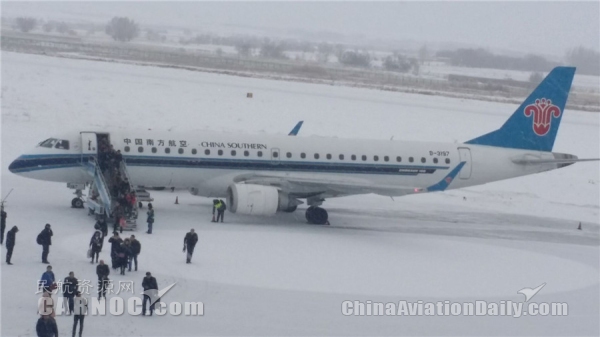

Supplement: S1 Dataset — All collected images were collected together, labeled and summarized one by one, and resulting classification results were roughly classified into three major categories: dry, wet and snowy. (ZIP) [file pone.0310858.s001.zip › weather1_data/snow_road/1287.jpg]

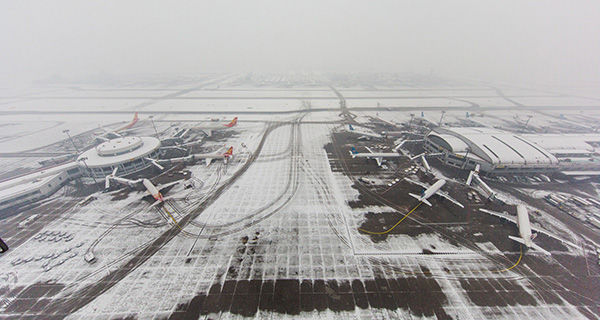

Supplement: S1 Dataset — All collected images were collected together, labeled and summarized one by one, and resulting classification results were roughly classified into three major categories: dry, wet and snowy. (ZIP) [file pone.0310858.s001.zip › weather1_data/snow_road/1291.jpg]

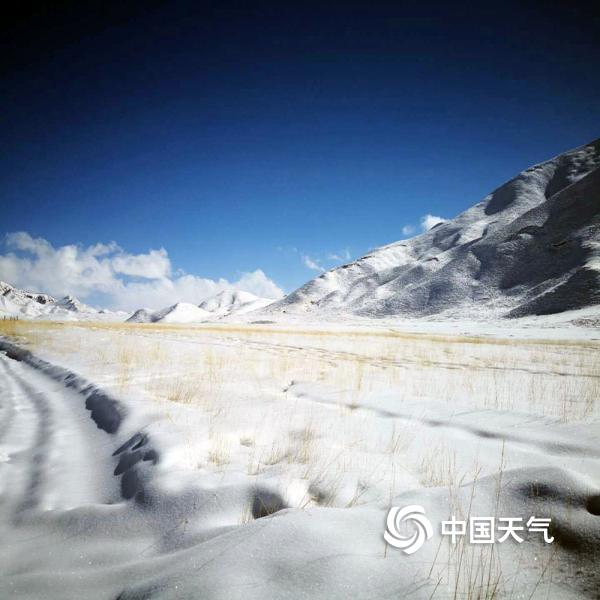

Supplement: S1 Dataset — All collected images were collected together, labeled and summarized one by one, and resulting classification results were roughly classified into three major categories: dry, wet and snowy. (ZIP) [file pone.0310858.s001.zip › weather1_data/snow_road/1293.jpg]

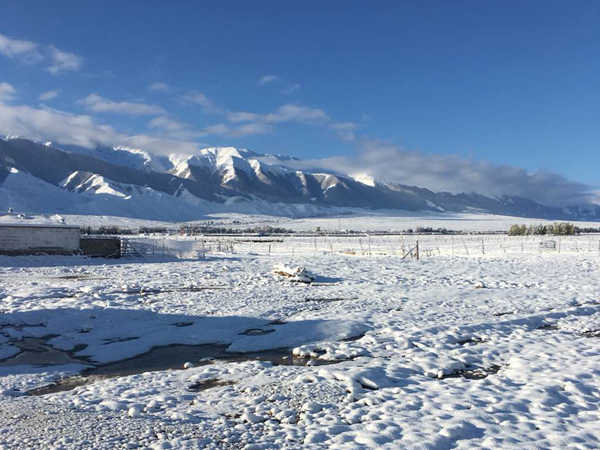

Supplement: S1 Dataset — All collected images were collected together, labeled and summarized one by one, and resulting classification results were roughly classified into three major categories: dry, wet and snowy. (ZIP) [file pone.0310858.s001.zip › weather1_data/snow_road/1299.jpg]

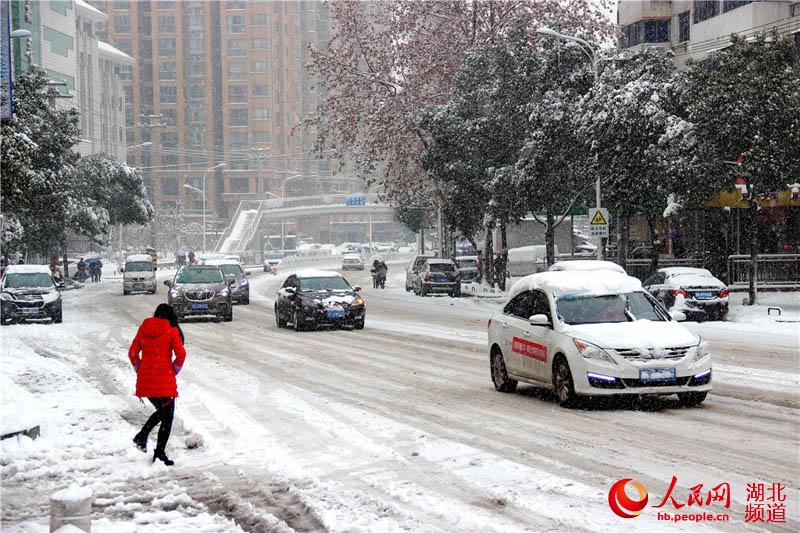

Supplement: S1 Dataset — All collected images were collected together, labeled and summarized one by one, and resulting classification results were roughly classified into three major categories: dry, wet and snowy. (ZIP) [file pone.0310858.s001.zip › weather1_data/snow_road/1316.jpg]

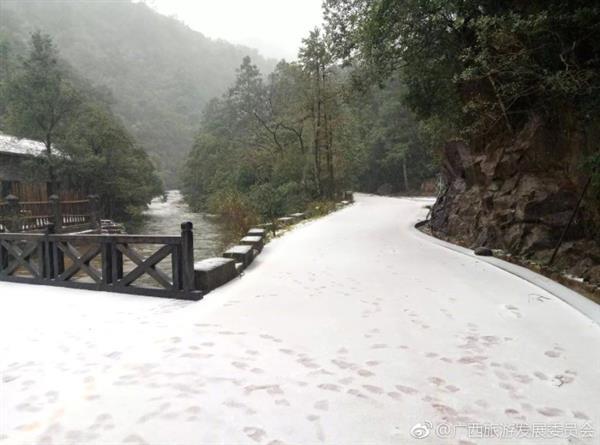

Supplement: S1 Dataset — All collected images were collected together, labeled and summarized one by one, and resulting classification results were roughly classified into three major categories: dry, wet and snowy. (ZIP) [file pone.0310858.s001.zip › weather1_data/snow_road/1317.jpg]
